# Supplementary material for: Optimising paediatric afferent component early warning systems: a hermeneutic systematic literature review and model development
Source: BMJ Open. 2019 Nov 14;9(11):e028796. doi: 10.1136/bmjopen-2018-028796 (PMC6886951; doi:10.1136/bmjopen-2018-028796)
Supplement: Supplementary data [file bmjopen-2018-028796supp001.pdf]

Appendix 1: Data Extraction Template 1

PUMA literature review template

**Study/Paper**	
Authors, title, date, source	
Purpose	
Contribution to review	
Design/Methods	
Participants/Setting	
Data type	
Theoretical framework	
Quality Appraisal Tool	
Quality Assessment	
Intervention type	
Formal study finding	
Recommendations	

**Context**	
History of intervention	
Country	
Organisation type (i.e. DGH, tertiary hospital, primary care)	
PEWS (efferent) wider activity system elements and inter-relationships with TTT [decision algorithms, CCOT, MET, ICU liaison, PICU, HDU]	
Infra-structural context [other related artefacts]	
Related activity systems [QI, audit, governance, referral processes and inter-relationships with TTT]	
Socio-cultural context [including experience of QI, stability of team and division of labour, teaching-non-teaching etc]	
Wider policy/organisational context	
**Intervention (TIDieR)**	
Name	
Rationale, theory or goal of the elements – active ingredients - identified as essential to the intervention [DA: this could be explicit or implicit in the paper]	
What (materials): Describe any physical or informational	

materials used in the intervention, including those provided to participants or used in intervention delivery or in training of intervention providers.<br><br>**TTT Affordances (including but not limited to indicative list)**<br>Paper/electronic<br>What observations/information are recorded?<br>How is data displayed?<br>Single parameter/ Aggregated weighting<br>Response algorithm(s) (including details)<br>Parental/patient involvement<br>Manual or automated monitoring<br>Proactive intermittent/continuous monitoring<br>Linked artefacts (e.g SBAR)<br>Audit/performance surveillance	

What (procedures): describe each of the procedures, activities, and/or processes used in the intervention, including any enabling or supportive activities.	
Who provided: For each category of intervention provider (for example, nursing assistant, nurse, junior doctor) describe their expertise, background and any specific training given and also the roles and relationships within the overall division of labour.	
How: Describe the mechanisms (implicit or explicit) about how the intervention, or intervention components have their effects	
How: Describe the modes of TTT delivery (such as face to face or some other mechanism such as phone or internet), or whether the intervention was provided individually or in a group.	
When and how much: Describe the number of times the intervention was delivered and over what period of time,	

<table>
<tr><td>including the number of sessions, schedule and intensity of dose (universal or selective application)</td><td colspan="4"></td></tr>
<tr><td>Tailoring: If the intervention was planned to be personalised or adapted, then describe what, why, when and how.</td><td colspan="4"></td></tr>
<tr><td>Modifications: If the intervention was modified during the course of the study, describe the changes (What, why, when, and how)</td><td colspan="4"></td></tr>
<tr><td>Assessment of intervention adherence or fidelity, describe how and by whom, and if any strategies were used to maintain or improve fidelity, describe them</td><td colspan="4"></td></tr>
<tr><td>If intervention adherence or fidelity was assessed, describe the extent to which the intervention was delivered as planned</td><td colspan="4"></td></tr>
<tr><td colspan="5">Implementation (NPT) (questions modified from Murray et al. 2010 and kennedy et al 2013)</td></tr>
<tr><td rowspan="2">Coherence [i.e. the meaning attributed to PEWS and whether it makes sense for users e.g. cultural and scientific legitimacy, feasibility]</td><td>Policy</td><td>Organisational</td><td>Professional</td><td>Patient/family</td></tr>
<tr><td></td><td></td><td></td><td></td></tr>
</table>

• Evidence that the intervention was easy to describe<br>• Evidence that it was recognised as different from existing ways of working by stakeholders<br>• Evidence that it has a clear purpose for all relevant actors<br>• Evidence that stakeholders recognise the benefits the intervention will bring and to whom<br>• Evidence that the intervention fitted with the overall goals and activity of the organisation				
Cognitive Participation [i.e. commitment and engagement with the intervention]<br>• Evidence that the target group see the intervention was a good idea<br>• Evidence that they were prepared to invest time and energy and work in it?				

• Evidence that the stakeholders thought their engagement with the intervention was appropriate<br>• Evidence that the stakeholders have taken steps to sustain the use of the intervention				
Collective Action [what are the skills and resources required to enact the intervention?]<br>• How did the intervention affect the user groups?<br>• What work was undertaken to operationalise the intervention?<br>• Did it promote or impede their work?<br>• Did staff require extensive training before they can use it?<br>• How compatible was the intervention with existing work practices?<br>• What impact did it have on the division of				

labour, resources, power and responsibility between different professional groups?<br>• Did it fit with the overall goals and activity of the organisation?				
Reflexive Monitoring [i.e methods for formal and informal evaluation of the effects of the intervention]<br>• Did stakeholders take practical steps to measure the influence of the intervention?<br>• How did users perceive the intervention once it has been in use for a while?<br>• Was the intervention seen as advantageous for patients and staff?<br>• Was it clear what effects the intervention has had (including unintended consequences)?<br>• Did users' contribute feedback on the				

intervention once it is in use?<br>• Was the intervention be adapted/improved on the basis of this experience?				
**Findings (positive, negative and unintended consequences) and useful content**				
**Emergent theories about key ingredients and mechanisms (including summary of supporting evidence)**				
**Emergent theories of the factors necessary for paediatric EWS to be normalised (including summary of supporting evidence)**				
**Additional Insights of note (including references to follow up)**				

Appendix 2: Search Methodology

Search Methods

Database Search

A comprehensive search was conducted across a wide-ranging set *of databases* from 1995 September 2016, which was then extended to May 2018 to identify relevant evidence/studies in English language on

- Paediatric Early Warning Systems (all study types).

A preliminary search strategy was developed using a set of key papers known to the group for Ovid Medline using both text words and Medical subject headings. A further three systematic searches was conducted across a range of databases from 1995 to September 2016 to identify relevant studies in the English language papers reporting on:

- Adult Early Warning Systems (qualitative studies only)
- Interventions to improve situational awareness (all study types)
- Structured communication tools for handover and handoff (all study types)

The Medline search strategy was translated to use across the rest of the databases.

The focus of the search strategy was to achieve high sensitivity and specificity for retrieving studies relevant to the review question. The Medline search strategy was modified according to the indexing systems of the other databases.

Table 1.

**Databases**
British Nursing Index
CINAHL (Cumulative Index of Nursing and Allied Health Literature)
Cochrane Central Register of Controlled Trials
Database of Abstracts of Reviews of Effectiveness
EMBASE
HMIC (Health Management Information Centre)
Medline
Medline in Process
Scopus
Web of Knowledge (Science Citation Indexes)

Additional searches

In addition to the above databases, we searched both published and unpublished literature. To identify supplementary papers, information on studies in progress, unpublished research or research reported in the grey literature was identified through searching a range of relevant websites and trial registers including Clinical Trials.gov. To identify published resources that had not yet been catalogued in the electronic databases, recent editions of key journals were hand-searched.

**Trial Registers**
ClinicalTrials.gov<br>https://clinicaltrials.gov/

UK Clinical Trials Gateway<br>http://www.ukctg.nihr.ac.uk/default.aspx
The WHO trial search portal for studies worldwide:<br>http://apps.who.int/trialsearch
**Journal site**
BMJ<br>http://www.bmj.com/theBMJ
BMJ Quality and safety<br>http://qualitysafety.bmj.com/
Websites and organisations
The Health Foundation

Identify relevant studies

The search results would be imported into reference management database *Endnote.* Duplicate references and clearly irrelevant citations will be removed. All remaining studies will then be downloaded into Dropbox for reviewers to screen for relevance.

Medline Search Strategy: Adult Early Warning Systems

1. ("early warning" adj5 scor*).ab,ti.
2. ("early warning" adj5 system* adj5 (deteriorat* or mortality or death or outcome* or harm* or safety)).ab,ti.
3. "acute illness severity".mp.
4. "early medical intervention"/ and ((prevent* or reduc* or improv*) adj5 (deteriorat* or mortality or death or outcome* or harm* or safety)).ab,ti.
5. ("early medical intervention" adj5 (tool* or scor* or index* or indicator* or indice* or assessment* or guide* or instrument* or criteria or parameter* or deteriorat* or mortality or death or monitor* or outcome* or harm* or safety)).ab,ti.
6. exp Health Status Indicators/ and ((tool* or scor* or index* or indicator* or indice* or assessment* or instrument* or criteria or parameter*) adj3 ((prevent* or reduc* or improv*) adj3 (deteriorat* or mortality or death or outcome* or harm* or safety))).ab,ti.
7. "Severity of Illness Index"/ and ((tool* or scor* or index* or indicator* or indice* or assessment* or instrument* or criteria or parameter*) adj5 ((prevent* or reduc* or improv*) adj5 (deteriorat* or mortality or death or outcome* or harm* or safety))).ab,ti.
8. exp Hospitals/ and ((Detecting or managing) adj3 deterioration).tw.
9. ("medical device" adj3 (prevent* or reduc* or improv*)).mp.
10. ("alert criteria" or "activation criteria" or "calling criteria").tw.
11. Hospital Rapid Response Team/
12. Clinical Alarms/
13. (outreach adj3 emergency).tw.
14. VitalPAC Early Warning Score.tw.
15. medical emergency team.tw.
16. Rapid Response Systems.mp.
17. Rapid Response Team.tw.
18. (((Detecting or managing) adj3 deterioration) and warning).tw.

19. track-and-trigger system.tw.
20. (Track adj trigger).tw.
21. (Track and trigger).tw.
22. trigger tools.tw.
23. SBAR technique*.mp.
24. ("rapid response" adj5 (prevent* or reduc* or improv*)).tw.
25. (score adj3 severity of illness).tw.
26. or/1-25
27. (qualitative or ethnograph* or thematic analysis or grounded theory or audio-recorded or transcribed or verbatim or ethnograph* or content analysis technique).ti,ab.
28. (("semi-structured" or semistructured or unstructured or informal or "in-depth" or indepth or "face-to-face" or structured or guide) adj3 (interview* or discussion* or questionnaire*)).ti,ab.
29. ((field or case) adj (stud* or research)).ti,ab.
30. Focus groups/ or Qualitative research/ or Interviews as topic/ or Interview, Psychological/ or ((focus or discussion) adj group*1).ti,ab.
31. (Questionnaires/ or interviews as topic/ or interview, psychological/) and (experience* or predictor* or determinant* or barrier* or facilitator* or enabler* or factor* associat* or perception* or perceive* or attitude* or view*1 or viewpoint* or standpoint* or encounter* or experience* or story or stories or narrative*1 or theme*1 or opinion* or concerns or motivat* or need*1).ti,ab.
32. (cross-sectional studies/ or cross-sectional survey.ti,ab. or correlation study.ti,ab.) and (predictor* or determinant* or barrier* or facilitator* or enabler* or factor* associat* or perception* or perceive* or attitude* or view*1 or viewpoint* or standpoint* or encounter* or experience* or story or stories or narrative*1 or theme*1 or opinion* or concerns or motivat* or need*).ti,ab.
33. process evaluation/ or process evaluation.ti,ab.
34. mixed method*1.ti,ab.
35. ((assoc* factor*1 or predictor* or determinant* or barrier* or facilitator* or enabler*) adj3 (interview* or survey* or questionnaire* or study)).ti,ab.
36. *motivation/
37. ((perception* or perceive* or attitude* or view*1 or viewpoint* or standpoint* or encounter* or experience* or story or stories or narrative*1 or description* or theme* or opinion* or need*1 or concerns or motivat*) adj3 (interview* or survey* or questionnaire* or study or explor* or evaluate or investigate* or analys* or collect*)).ti,ab.
38. (themes adj3 (identif* or analy* or review or explor* or investigat*)).ti,ab.
39. "attitude of health personnel"/ or *attitude to health/
40. exp emotions/
41. consumer satisfaction/
42. personal satisfaction/
43. exp professional-patient relations/
44. exp interprofessional relations/
45. "Health Services Needs and Demand"/
46. or/27-45
47. 26 and 46
48. limit 47 to (english language and humans and yr="1995 -Current")

Medline Search Strategy: Structured communication tools for handover and handoff

1. Situation Background Assessment Recommendation.tw.
2. SBAR.mp.
3. (ABC and "collaborative care").tw.
4. Patient Handoff/
5. Patient Discharge/ and ABC.mp.
6. exp Patient Transfer/ and checklist.mp.
7. (handoff adj3 communication).tw.
8. (handoff adj5 (tool or approach or technique or method)).tw.
9. ("information transfer" and "emergency care").tw.
10. Patient Discharge/ and information transfer.tw.
11. ("information transfer" and "critical care").tw.
12. ("information transfer" and handoff).tw.
13. written checklist.tw.
14. ("Rapid Syndrome Validation Project" or RSVP).tw.
15. "Communication tools".tw.
16. "Escalation of care".tw.
17. or/1-16
18. limit 17 to (english language and humans and yr="1995 -Current")

Medline Search Strategy: Paediatric Early Warning Systems to include observation and training

1. ("early warning" adj5 scor*).ab,ti.
2. ("early warning" adj5 system* adj5 (deteriorat* or mortality or death or outcome* or harm* or safety)).ab,ti.
3. "acute illness severity".mp.
4. "early medical intervention"/ and ((prevent* or reduc* or improv*) adj5 (deteriorat* or mortality or death or outcome* or harm* or safety)).ab,ti.
5. ("early medical intervention" adj5 (tool* or scor* or index* or indicator* or indice* or assessment* or guide* or instrument* or criteria or parameter* or deteriorat* or mortality or death or monitor* or outcome* or harm* or safety)).ab,ti.
6. exp Health Status Indicators/ and ((tool* or scor* or index* or indicator* or indice* or assessment* or instrument* or criteria or parameter*) adj3 ((prevent* or reduc* or improv*) adj3 (deteriorat* or mortality or death or outcome* or harm* or safety))).ab,ti.
7. "Severity of Illness Index"/ and ((tool* or scor* or index* or indicator* or indice* or assessment* or instrument* or criteria or parameter*) adj5 ((prevent* or reduc* or improv*) adj5 (deteriorat* or mortality or death or outcome* or harm* or safety))).ab,ti.
8. exp Hospitals/ and ((Detecting or managing) adj3 deterioration).tw.
9. ("medical device" adj3 (prevent* or reduc* or improv*)).mp.
10. ("alert criteria" or "activation criteria" or "calling criteria").tw.
11. Hospital Rapid Response Team/
12. Clinical Alarms/
13. (outreach adj3 emergency).tw.
14. VitalPAC Early Warning Score.tw.

15. medical emergency team.tw.
16. Rapid Response Systems.mp.
17. Rapid Response Team.tw.
18. (((Detecting or managing) adj3 deterioration) and warning).tw.
19. track-and-trigger system.tw.
20. (Track adj trigger).tw.
21. (Track and trigger).tw.
22. trigger tools.tw.
23. SBAR technique*.mp.
24. ("Situation Background Assessment Recommendation" or SBAR).tw.
25. (patient* adj3 deteriorat*).tw.
26. (deterioration adj3 hospital).tw.
27. (patient deterioration adj4 hospital).tw.
28. (Patients adj4 adverse event).tw.
29. clinical deterioration.tw.
30. ("rapid response" adj5 (prevent* or reduc* or improv*)).tw.
31. (score adj3 severity of illness).tw.
32. Vital signs.tw.
33. or/1-26
34. P?ediatric Early Warning.mp.
35. p?ediatric alert.tw.
36. p?ediatric early warning systems.mp.
37. p?ediatric risk of mortality.tw.
38. P?ediatric Rapid Response Team.tw.
39. Point-of-Care Systems/ and ((paediatric or pediatric) adj3 (improve or identify or detect* or outcome or early or critical or emergency)).tw.
40. P?ediatric Advanced Warning Score.tw.
41. neonatal early warning.tw.
42. infant early warning.tw.
43. p?ediatric rapid response.tw.
44. Bedside paediatric early warning.tw.
45. Bedside PEWS.tw.
46. or/34-45
47. 33 or 46
48. Health Plan Implementation/
49. (implement* or applicat* or execute).tw.
50. (observ* or monitoring or monitor or education).tw.
51. Risk Assessment/
52. Education/
53. Education, Continuing/
54. "Hospitals, Teaching"/
55. Decision Making/
56. Safety Management/
57. Patient Simulation/
58. Awareness/
59. Knowledge/
60. *"Attitude of Health Personnel"/

61. *"Education, Medical, Continuing"/
62. *"Interdisciplinary Communication"/
63. Communication/
64. Monitoring, Physiologic/
65. Decision Making/
66. Judgment/
67. Needs Assessment/
68. Interprofessional Relations/
69. Interdisciplinary Communication/
70. ((organi#ation* adj2 (structur* or form* or function* or determinant* or factors or environme nt* or process* or culture*)) and (outcome? or perform* or satisf* or efficien* or effective* or equ* or growth or develop* or justice or quality or culture* or manage* or leader*)).tw.
71. exp *psychology, industrial/ or *absenteeism/ or *efficiency/ or *job satisfaction/ or *"task performance and analysis"/ or *"time and motion studies"/ or *work simplification/ or *time management/ or *vocational guidance/
72. Decision Support Systems, Clinical/
73. Workflow/
74. or/48-73
75. Nurses/
76. Physicians/
77. (nurse* or physician* or doctor*).tw.
78. Medical Staff/
79. Medical Staff, Hospital/ or Nursing Staff, Hospital/ or Intensive Care Units/
80. or/75-79
81. 47 and 74 and 80
82. limit 81 to (english language and humans and yr="1995 -Current")

Medline Search Strategy: Interventions to improve situational awareness

1 Patient Safety/ (6742)

2 patient safety.tw. (13735)

3 1 or 2 (18307)

4 "Situation* Awareness".tw. (488)

5 3 and 4 (69)

6 limit 5 to (english language and humans and yr="1995 -Current")

Appendix 3: Data extraction template 2

**Study/Paper**	
Authors, title, date, source	
Purpose	
Design/Methods	
Participants/Setting	
Data type	
Theoretical framework	
Quality Assessment	
Formal study finding	

**Theory Area 1 – Properties of the monitoring system in EWS**
**What are the technical characteristics of the monitoring system?**
**What are the policies/processes associated with the monitoring system?**
**What are roles and responsibilities of the monitoring system?**
**What are the skills and resources needed to enact the monitoring system?**
**How often is the monitoring system applied?**
**How effective is the monitoring system?**
**What is the mechanisms (implicit or explicit) by which the monitoring system has its effects?**
**What actions are necessary to ensure the effectiveness of the monitoring system?**
**What is the relationship of the monitoring system and the context?**
**What is the relationship of the monitoring system to the recording system?**
Is the evidence provided in this theory area good and relevant enough to be included in the synthesis?

**Theory Area 2 – Properties of the recording system in EWS**
**What are the technical characteristics of the recording system?**

**What are the policies/processes associated with the recording system?**
**What are the roles and responsibilities of the recording system?**
**How effective is the recording system?**
**What are the skills and resources needed to enact the recording system?**
**What is the mechanism (implicit or explicit) by which the recording system has its effects?**
**What actions are necessary to ensure the effectiveness of the recording system?**
**What is the relationship between the recording system and the context?**
**What is the relationship between the recording system, the monitoring and detection system?**
Is the evidence provided in this theory area good and relevant enough to be included in the synthesis?

**Theory Area 3 – Properties of the detection/trigger system in EWS**
**What are the technical characteristics of the detection/trigger system?**
**What are the policies/processes associated with the detection/trigger system?**
**What are the roles and responsibilities involved in the detection/trigger system?**
**What are the skills and resources needed to enact the detection system?**
**How effective is the trigger system?**
**What is the mechanism (implicit or explicit) by which the detection/trigger system has its effects?**
**What actions are necessary to ensure the effectiveness of the detection/trigger system?**
**What is the relationship between the trigger system and the context?**
**What is the relationship between the trigger system and the activation system?**
Is the evidence provided in this theory area good and relevant enough to be included in the synthesis?

**Theory Area 4 – Properties of the activation system in EWS**
**What are the technical characteristics of the activation system?**

**What are the policies/processes associated with the activation system?**
**What are the roles and responsibilities involved in the activation system?**
**What are the skills and resources needed to enact the activation system?**
**How effective is the activation system?**
**What is the mechanism (implicit or explicit) by which the activation system has its effects?**
**What actions are necessary to ensure the effectiveness of the activation system?**
**What is the relationship of the activation system and the context?**
**What is the relationship between the activation system and the monitoring, recording and trigger system?**
Is the evidence provided in this theory area good and relevant enough to be included in the synthesis?

**Theory Area 5 – Properties of family involvement in EWS**
**At what stage in the PEWS process are families involved?**
**What are the roles and responsibilities involved in ensuring family involvement?**
**How effective is family involvement in the EWS process?**
**What are the mechanisms by which families are involved in EWS?**
**How effective are mechanisms for involving families in EWS?**
**What are the benefits and dis-benefits of family involvement?**
Is the evidence provided in this theory area good and relevant enough to be included in the synthesis?

**Theory Area 6 – Properties of chart design in EWS**
**What are the optimal properties of chart design to ensure effective monitoring?**
**What are the optimal properties of chart design to ensure effective recording?**
**What are the optimal properties of chart design to ensure effective detection?**

Is the evidence provided in this theory area good and relevant enough to be included in the synthesis?

**Theory Area 7– Properties of implementation and normalisation in EWS**
**What are the barriers to implementation and normalisation of EWS?**
**What actions are necessary to overcome barriers to the implementation and normalisation of EWS?**
**What actions are effective in overcoming barriers to the implementation and normalisation of EWS?**
**What are the mechanisms by which effective actions in overcoming barriers to implementation and normalisation of EWS have their effects?**
**What are the key pillars of an effective reflexive monitoring system?**
Is the evidence provided in this theory area good and relevant enough to be included in the synthesis?
