# Supplementary material for: Optimising paediatric afferent component early warning systems: a hermeneutic systematic literature review and model development
Source: BMJ Open. 2019 Nov 14;9(11):e028796. doi: 10.1136/bmjopen-2018-028796 (PMC6886951; doi:10.1136/bmjopen-2018-028796)
Supplement: Author's manuscript [file bmjopen-2018-028796.draft_revisions.pdf]

BMJ Open is committed to open peer review. As part of this commitment we make the peer review history of every article we publish publicly available.

When an article is published we post the peer reviewers' comments and the authors' responses online. We also post the versions of the paper that were used during peer review. These are the versions that the peer review comments apply to.

The versions of the paper that follow are the versions that were submitted during the peer review process. They are not the versions of record or the final published versions. They should not be cited or distributed as the published version of this manuscript.

BMJ Open is an open access journal and the full, final, typeset and author-corrected version of record of the manuscript is available on our site with no access controls, subscription charges or pay-per-view fees (http://bmjopen.bmj.com).

If you have any questions on BMJ Open's open peer review process please email info.bmjopen@bmj.com

BMJ Open

Optimising Paediatric Afferent Limb early warning Systems: a hermeneutic systematic literature review and model development

Journal:	*BMJ Open*
Manuscript ID	bmjopen-2018-028796
Article Type:	Research
Date Submitted by the Author:	19-Feb-2019

Complete List of Authors:	Jacob, Nina; Cardiff University, Centre for Trials Research<br>Moriarty, Yvonne; Cardiff University, Centre for Trials Research<br>Lloyd, Amy; Cardiff University, Centre for Trials Research<br>Mann, Mala; Cardiff University, University Library Services<br>Tume, Lyvonne N.; University of the West of England Bristol,<br>Sefton, G.; Alder Hey Childrens NHS Fdn Trust,<br>Powell, Colin; Children's Hospital for Wales, Paediatrics<br>Roland, Damian; Paediatric Emergency Medicine Leicester Academic (PEMLA) Group, Emergency Department; University of Leicester Department of Health Sciences, SAPPHIRE group<br>Trubey, Robert ; Cardiff University, Centre for Trials Research<br>Hood, Kerenza; Cardiff University,<br>Allen, Davina; Cardiff University

Keywords:	STATISTICS & RESEARCH METHODS, PAEDIATRICS, QUALITATIVE RESEARCH

SCHOLARONE™
Manuscripts

Optimising Paediatric Afferent Limb Early Warning Systems: A hermeneutic systematic literature review and model development

Authors: Nina Jacob[1†], Yvonne Moriarty[1], Amy Lloyd [1], Mala Mann[2], Lyvonne Tume[3], Gerri Sefton [4], Colin Powell[5, 6], Damian Roland [7], Robert Trubey[1], Kerenza Hood[1], Davina Allen[8]

1 Centre for Trials Research, Cardiff University, Heath Park, Cardiff, UK

2 Specialist Unit for Review Evidence (SURE), Cardiff University, Heath Park, Cardiff, UK

3 Faculty of Health and Applied Sciences, University of the West of England Bristol, Bristol, UK

4 Paediatric Intensive Care Unit, Alder Hey Children's NHS Foundation Trust, Liverpool, UK

5 Department of Child Health, Children's Hospital for Wales, Division of Population Medicine School of Medicine, Cardiff University, Cardiff, UK,

6 Department of Pediatric Emergency Medicine, Sidra Medicine, Doha, Qatar

7 Paediatric Emergency Medicine Leicester Academic (PEMLA) Group, Emergency Department, Infirmary Square, Leicester, UK

8 School of Healthcare Sciences, Cardiff University, Eastgate House, Cardiff

Corresponding author:

Dr. Nina Jacob, Centre for Trials Research, Cardiff University, Heath Park, Cardiff, UK

Keywords: PEWS, track and trigger scores, early warning scores, clinical deterioration, children, systematic review

Word Count: 4431

For peer review only

ABSTRACT

Objective: To identify the core components of successful afferent limb early warning systems in detecting and initiating action in response to clinical deterioration in the in-patient paediatric environment.

Data sources: Studies of paediatric and adult early warning systems and related research on interventions to support the detection of clinical deterioration and escalation of care in hospital contexts.

Synthesis methods: Hermeneutic systematic literature review informed by Translational Mobilisation Theory and Normalisation Process Theory and development of a propositional model of an optimal afferent limb paediatric early warning system. PROSPERO registration number CRD42015015326.

Eligibility criteria: We included papers reporting on the (1) wider socio-material and contextual factors associated with successful or unsuccessful paediatric early warning system or (2) the component of the afferent limb of paediatric early warning system. All study designs were considered.

Results: Detecting and initiating action in response to clinical deterioration involves several challenges and the potential failure points in early warning systems are well documented. Track and Trigger Tools are a common component of early warning systems and have value in supporting key mechanisms of action but depend on certain preconditions for successful integration into practice. A number of supplementary interventions have been proposed to improve the effectiveness of peadiatric early warning systems but there is limited evidence to recommend their wider use. This reflects both the weight and quality of the evidence, the extent to which paediatric systems are conditioned by the local clinical context, and also the need to attend to the

For peer review only

For peer review only - http://bmjopen.bmj.com/site/about/guidelines.xhtml

relationship between system components which work in concert not in isolation. While it was not possible to make empirical recommendations for practice, the review methodology enabled the generation of theoretical inferences about the core components of optimal afferent limb paediatric early warning systems. These are presented as a propositional model conceptualised as three sub-systems: detection, preparation and action.

Conclusions: There is a growing consensus of the need to think beyond Track and Trigger Tools in improving action to detect and respond to clinical deterioration. Clinical teams wishing to improve afferent limb paediatric early warning systems can use the propositional model to consider systematically the constellation of factors necessary to support detection, preparation and action and consider how these arrangements can be implemented in their local context.

STRENGTHS AND WEAKNESSES OF THE STUDY

- The literature in this field is heterogeneous and better at identifying system weakness than it is effective improvement interventions.
- By deploying social theories and a hermeneutic review methodology it was possible to develop a propositional model of the core components of an afferent limb paediatric early warning system.
- The model is derived from logical inferences drawing on the overall evidence synthesis, social theories and clinical expertise, rather than strong empirical evidence of single intervention effectiveness.
- There is a growing consensus of the need to take a whole systems approach to improve the detection and response to deterioration in the in-patient paediatric population and this paper offers an evidence-based frameworks for this purpose.

For peer review only

This study is funded by the National Institute for Health Research (NIHR) Health Services and Delivery Research (HS&DR) programme (12/178/17). The views and opinions expressed in this paper are those of the authors and not necessarily those of the NHS, the NIHR or the Department of Health.

INTRODUCTION

Failure to recognise and act upon signs of clinical deterioration in the hospitalised child is an acknowledged safety concern (1). Track and Trigger Tools (TTT) are a common response to this problem. A TTT consists of sequential recording and monitoring of physiological, clinical and observational data. When a certain score or trigger is reached then a clinical action should occur including, but not limited to, altered frequency of observation, senior review or more appropriate treatment or management. Tools may be paper based or electronic and monitoring can be automated or undertaken manually by staff.

Despite the growing use of TTTs there is limited evidence of their effectiveness as a single intervention in reducing mortality or arrest rates in hospitalised children (2,3). Results from the largest international cluster randomised control trial of a TTT (the Beside PEWS) did not support TTT use to reduce mortality, and highlighted the multifactorial mechanisms involved in detecting and initiating action in response to deterioration (4). These findings lend further weight to a developing consensus about the need to look beyond TTTs to the impact of wider system factors on detecting and responding to deterioration in the in-patient paediatric population (2,5–9). This paper reports on a theoretically informed systematic hermeneutic literature review (10) to identify the core components and mechanisms of action of successful afferent limb early warning systems in paediatric hospitals and is one of three linked reviews

For peer review only - http://bmjopen.bmj.com/site/about/guidelines.xhtml

undertaken as part of a wider UK study commissioned to develop and evaluate an evidence-based paediatric warning system (11). It addressed the following question:

What socio-material and contextual factors are associated with successful or unsuccessful Paediatric Early Warning Systems (with or without TTTs)?

METHOD

DESIGN

We performed a hermeneutic systematic review of the relevant literature. A hermeneutic systematic review is an iterative process, integrating analysis and interpretation of evidence with literature searching and is designed to develop a better understanding of the field (10). The popularity of the method is growing in health services research where it has value in generating insights from heterogeneous literatures that cannot be synthesised through standard review methodology (12) and would otherwise produce inconclusive findings (see (9). The purpose of the review was not exhaustive aggregation of evidence, but to develop an understanding of the social, material and contextual factors associated with successful or unsuccessful paediatric early warning systems.

THEORETICAL FRAMEWORK

Data extraction and interpretation was informed by Translational Mobilisation Theory (TMT) (13,14) and Normalisation Process Theory (NPT) (15,16). TMT is a practice theory which explains how goal-oriented collaborative activity is mobilised in unpredictable environments [Box 1] and how the relevant mechanisms of action are conditioned by the local context. It is well suited for understanding early warning systems which require the organisation of action in evolving conditions, in a variety of

For peer review only

clinical environments, with different teams, skill mixes, resources, structures, and technologies. NPT shares the same domain assumptions as TMT and is concerned with 'how and why things become, or do not become, routine and normal components of everyday work' (15,16), directing attention to the preconditions necessary for successful implementation of interventions. The theoretical framework informed our data extraction strategy, interpretation of the evidence, and the development of a propositional model of an optimal paediatric early warning system.

FOCUS OF THE REVIEW

The literature in this field identifies four integrated components which work together to provide a safety system for at risk patients: (1) the afferent component which detects deterioration and triggers a response, (2) the efferent component which consists of the people and resources providing a response; (3) a process improvement component, which includes system auditing and monitoring and (4) an administrative component focusing on organisational leadership and education required to implement and sustain the system (17). Our focus was limited to the afferent components of the system.

STAGES OF THE REVIEW

STAGE 1: SCOPING THE LITERATURE

Literature was identified through a recent scoping review (7), team members' knowledge of the field, hand searches and snowball sampling techniques. The purpose was to (1) inform our review question and eligibility criteria and (2) identify emerging themes and issues. Whilst we drew on several reviews of the literature (5, 12-14) we always consulted original papers. Data were extracted using Data Extraction Template 1 (Appendix 1) and analysed to produce a provisional conceptual model of the core components of paediatric early warning systems. Additional themes of relevance were

For peer review only - http://bmjopen.bmj.com/site/about/guidelines.xhtml

identified: family involvement, situational awareness (SA), structured handover, observations and monitoring and the impact of electronic systems and new technologies.

STAGE 2: SEARCHING FOR THE EVIDENCE

We undertook systematic searches of the paediatric and adult early warning system literature (the goals and mechanisms of collective action in detection and rescue trajectories are the same). For the adult literature we used the same search strategies but added a qualitative filter to limit the scope to studies most likely to yield the level of socio-material and contextual detail of value to the review. Literature informing additional areas of interest was located through a combination of systematic and hand searches. Systematic searches (Searches 3 and 4) were undertaken in areas where we anticipated locating evidence of the effectiveness of specific interventions to strengthen early warning systems. Theory driven searches reflected the conceptual requirements of the model development.

SYSTEMATIC SEARCHES

Four systematic searches were conducted across a range of databases from 1995 to September 2016 to identify relevant studies in English language papers reporting on:

1. Paediatric Early Warning Systems

2. Adult Early Warning Systems

3. Interventions to improve situational awareness.

4. Structured communication tools for handover and handoff

Detailed information on the search methodology can be found in Appendix 2.

THEORY DRIVEN SEARCHES

For peer review only - http://bmjopen.bmj.com/site/about/guidelines.xhtml

Additional theory-driven searches were conducted in the following areas:

1. Family involvement
2. Observations and monitoring
3. The impact of electronic systems

These were a combination of exploratory, computerised, snowball, and hand searches. As the analysis progressed, we continued to review new literature on early warning systems as this was published.

SCREENING

After removing duplicates 5256 references were identified for screening. A modified PRISMA flow diagram is provided [Table 1]. Papers were screened by title to assess eligibility and then by full text to assess relevance for data extraction. Searches 1 and 2 were screened by two researchers, searches 3 and 4 were screened by the lead reviewer. Grey literature was excluded to keep the scale of the review manageable.

STAGE 3: DATA EXTRACTION AND APPRAISAL

Data Extraction Template 2 [Appendix 3] was applied to all papers included in the review. Evidential fragments and partial lines of inquiry formed the unit of analysis rather than whole papers. Fragments were drawn from papers that were quality assessed according to study type and the contribution made to the developing analysis. Data extraction and quality appraisal were undertaken concurrently and double-checked by a second reviewer.

STAGE 4: DEVELOPING A PROPOSITIONAL MODEL

A propositional model was developed specifying the core ingredients of a paediatric early warning system [Table 2]. It comprises of logical inferences derived from the theoretical framework and evidence synthesis, informed by clinical experts on the team. Iterations of the model were developed in collaboration with clinical colleagues. A

For peer review only - http://bmjopen.bmj.com/site/about/guidelines.xhtml

series of face-to-face meetings were conducted to review structure, wording and applicability to clinical practice.

PATIENT AND PUBLIC INVOLVEMENT

This review was conducted as part of a larger mixed-methods study (ISRCTN94228292), which used a formal, facilitated parental advisory group. The group comprised parents of children who had experienced an unexpected adverse event in a paediatric unit and provided input which helped to shape the broader research questions and outcome measures. The results of the review will be disseminated to parents through this group.

RESULTS

INCLUDED STUDIES

Seventy-three papers were included in the review. Thirty-six papers focussed on TTT implementation and use in paediatric and adult contexts (fourteen from the paediatric search and the remaining twenty-two from the adult focussed search); the remaining thirty-seven papers contributed supplementary data on factors related to the wider warning system. See Table 3 for a detailed breakdown of this process. No studies were located that adopted a whole systems approach to detecting and responding to deterioration.

ANALYSIS

In TMT the primary unit of analysis is the 'project', which defines the social and material actors (people, materials, technologies) and their relationships involved in achieving a particular goal. The goals of the afferent paediatric warning system are first, that the child is identified as at risk and a vital signs monitoring regime instigated;

For peer review only - http://bmjopen.bmj.com/site/about/guidelines.xhtml

second, that evidence of deterioration is identified through monitoring and categorised as such and; third, that timely and appropriate action is initiated in response to deterioration. Our analysis of the literature suggests that three sub-systems within the afferent limb of early warning systems support these processes: detection, preparation and action. In the next section we report on the literature in relation to each subsystem.

DETECTION

The goal of the detection sub-system is to recognise early signs of deterioration so the child becomes the focus of further clinical attention. This requires first, that the child is identified as at risk and a vital sign monitoring regime instigated and second, that the child is identified as showing signs of deterioration.

While the evidence on TTT effectiveness on outcomes is weak, the literature suggests that TTTs have value in supporting process mechanisms in the detection sub-system. Vital signs monitoring is undertaken on all hospital in-patients and, like other high volume routine activity, is often delegated to junior staff (18,19,28–37,20–27) who may not have sufficient skills to interpret results (20,21,36). TTTs have value in mitigating these risks: by specifying physiological thresholds that indicate deterioration they take knowledge to the bedside and act as prompts to action (18,38) which can lead to more frequent monitoring and improved detection of deterioration (39).

TTT's effectiveness in fulfilling these functions depends on certain preconditions. The review highlighted that TTT use was impacted by the availability of appropriate and functioning equipment (21,26,28,33,38,40–44), (in)adequate staffing and night time pressures (21,25,46–50,28,29,36,39–42,45) and an appropriately skilled workforce (25,27,54,55,34,35,41,47,48,51–53). On this latter point, while several papers report

For peer review only

on education packages to improve the detection of deterioration, the evidence is not robust enough to recommend specific programs (22–24,27,29,34,53,56–58).

TTTs are also used differently depending on the experience of the user. For juniors, they provide a methodology and structure for monitoring clinical instability and identifying deterioration, whereas more experienced staff reportedly use TTTs as confirmatory technologies (15-29). The importance of professional intuition in detecting deterioration is extensively reported across the literature (18,19,36–41,43,44,46,48,20,49–51,58–64,21,25,26,28,30,31,35) and several authors recommend the inclusion of 'staff conern' in tool criteria (25,46,49,55). This is important; TTTs may be of less value in patients with chronic conditions because of altered normal physiology or where subtle changes are difficult to detect (60). It is also the case that TTTs are implemented in contexts governed by competing organisational logics which impact on their value and use (41,56,52). For example, Iddrisu et al. (55) show TTTs have limited value immediately after surgery because acceptable vital signs parameters are different in the immediate post-operative period.

There is growing interest in the literature in strategies that facilitate patient and relative involvement in the early detection of deterioration (66,67). Healthcare professionals depend on families to explain their child's normal physiological baseline and identify subtle changes in their child's condition but this information is not always systematically obtained (68,69). Some authors propose family involvement in interdisciplinary rounds[1] (70), but this requires parents to have detailed information about the signs and symptoms they should be attending to (69) and as yet there is little

[1] This is an editorial paper

For peer review only - http://bmjopen.bmj.com/site/about/guidelines.xhtml

evidence on effective strategies for how they might be involved in the detection of deterioration (70).

While much of the literature reports on intermittent manual vital signs monitoring and paper-based recording systems, across the developed world there is a growing use of electronic technologies, which have important implications for the wider detection sub-system. We considered a number of evaluations of new technologies which indicated that electronic vital signs recording is associated with a number of positive outcomes, particularly timeliness and accuracy, when compared with paper-based systems (71). They can provide prompts or alerts for monitoring (72–74), which facilitates better recognition of deterioration and is associated with a reduction in mortality (73,75). These studies tend to evaluate new technologies in isolation, however, and do not engage with the literature highlighting alarm fatigue which is known to mitigate effectiveness over time or concerns about over burdening staff with alerts (76,77). Moreover, the successful implementation of new technologies is conditioned by the local context. For instance, where manual input into an electronic device is required, access to computers is an essential precondition. When computers were not available staff 'batch' the collection of vital signs before data entry, thereby delaying the timely detection of deterioration (26,43,78). In another study where the electronic system was found to be cumbersome and separated the collection and entry of data from the review of vital signs, verbal reports were favoured to ensure timely communication of information (79). See Table 4 for a summary of the evidence reported.

PREPARATION

Detecting and responding to deterioration involves the coordination of action in conditions of uncertainty and competing priorities. The goal of the 'Preparation' sub-

For peer review only - http://bmjopen.bmj.com/site/about/guidelines.xhtml

system is to ensure the clinical team are ready to act in the event of evidence of deterioration and is reflected in the growing interest in in the literature on structures to facilitate team situational awareness, group decisions and planning (59).

A number of studies report on 'huddles' in facilitating situational awareness (31,61,80,81). A huddle is a multidisciplinary event scheduled at pre-determined times where members discuss specific risk factors around deterioration and develop mitigation plans. One study combined the introduction of huddles with a "watchstander", a role fulfilled by a charge nurse or senior resident, whose primary function is to know patients at high risk for deterioration (81). These initiatives were associated with a near fifty per cent reduction in transfers from acute to intensive care determined to be unrecognized situation awareness events. A further strategy identified by Goldenhar et al. describes the use of the 'watcher' category to designate a patient as at risk where staff have a 'gut feeling' deterioration is likely (80).

Handovers are integral to clinical communication and contribute to situational awareness. The extensive literature on handover indicates that information sharing can be of variable quality (45,52,82) and there is growing evidence that structured approaches improve this (29,45,52,59,80,82–86). Ranging from a checklist system (83,85) to a cognitive aid developed through consensus (22,86), most of the published interventions are variations of the SBAR (Situation, Background, Assessment, and Recommendation) tool (52,82–84). While effective handover depends on communicative forms that extend beyond the information transfer that is typically the focus of structured handover tools (82), in the context of early warning systems a lack of standardisation allows greater margin for individualistic practices and difficulties accessing complementary knowledge and establishing shared understandings (45).

For peer review only - http://bmjopen.bmj.com/site/about/guidelines.xhtml

For peer review only

There is also a literature on the use of common information spaces – such as white boards – in facilitating situational awareness in the healthcare team (22,32,45,51,53,56,63). These should be in a visible location and colour-coded to correspond with the TTT score, where relevant (45,53,56). Electronic systems automate this information and allow information to be reviewed remotely (81). However, they disconnect vital signs data from the patient and hence other indicators of clinical status and access to data is contingent upon the availability of computers (26,43,45,78,87).

The literature indicates that situational awareness can be facilitated in different ways in different contexts and it is the relationship between system elements that is important (45). In their study on situational awareness in delivery suites, Mackintosh et al. discuss the three main supports for situational awareness - whiteboard, handover and coordinator role - and illustrate how these interacted in organisations with strong situational awareness compared to those with reduced levels. Crucially this 'interplay' between the different activities was highly context dependent; "the same supports used differently generate different outcomes" pg52 (45). See table 5 for a summary of the preparation evidence.

ACTION

The goal of the "Action" sub-system is to initiate appropriate action in response to evidence of deterioration. The literature suggests that mobilising action across professional boundaries/hierarchies is challenging, with differences in language between doctors and nurses and power dynamics contributory factors (26,39,48,50,55,58). TTTs are in part a response to the challenges of communication in mobilising action in response to deterioration. By transforming a series of discrete observations into a summative indicator of deterioration – such as a score or a trigger –

For peer review only - http://bmjopen.bmj.com/site/about/guidelines.xhtml

TTTs 'translate' and package the patient's status into a form that can be readily communicated enabling individual level clinical data to be synthesised, made sense of and shared (18,19,28–32,38,40,44,46,48,20,49,54,62,21–27). Several studies also report on the use of SBAR in this context. Like TTTs, SBAR translates information into a form that provides structure, consistency and predictability when presenting patient information. SBAR has been shown to help establish common language and expectations, minimising differences in training, experience and hierarchy and facilitating nurse-clinician communication. While several papers advocate combining SBAR with TTTs (22,24,26,29,34,43,48), none specifically evaluated SBAR use. Mackintosh et al. highlight that audit data suggests resistance to SBAR, with others cautioning that overextending SBAR use carries the risk of SBAR fatigue and attenuation of its effects (26).

Structured communication tools like TTTs and SBAR do not solve all the challenges of acting in response to evidence of deterioration. Barriers to action were widely reported in the literature where these tools were in place. These include: a general disinclination to seek help (18,19,37,38,40,46,48,49,54,60,63,20,21,24,26,28,30,35,36), concerns about appearing inadequate in front of colleagues (19,21,35,37,48,63), and failure of staff to invest in the escalation or calling criteria (20,21,47). A number of papers also reported negative attitudes to Rapid Response Team (RRT) or Medical Emergency Team (MET) use in the efferent component of safety systems. METs and RRTs operate outside the immediate medical team and create different issues in paediatric warning systems than when the escalation response is managed by the treating team. These include a reluctance to activate because of the perceived busyness of Paediatric Intensive Care Unit or medical staff (19,28,38,46,48,49), because previous expectations about an

For peer review only - http://bmjopen.bmj.com/site/about/guidelines.xhtml

appropriate response were not met, or a sense that the situation was under control (particularly when the physiological instability is in the area of expertise of the treating team) (21,28,30,37,40,48,50,60).

No literature reported on successful interventions to facilitate RRT use, but several propose strategies to support escalation where there was no designated response team in place in the efferent limb. These includes informal peer-support, where inexperienced staff team up with more experienced staff (20,28,48,60,63); clear structures to support action and a supportive culture that does not penalise individual decision-making, including the use of a 'no false alarms' policy so staff are not deterred from escalating care (21,29,73, 36). Senior leadership is consistently identified as important (8,19,34,45,50,56,62,63,20–22,24,26,29,31,32); lack of support from superiors meant that staff are less likely to escalate and more likely to adhere to hierarchies within the current system (24,39,62). There is some evidence to suggest that any escalation policy should be linked to an administrative arm that reinforces the system, measures outcomes and works to ensure an effective system (26,29).

There is a small literature on family involvement in the Action Subsystem. Several studies report on Condition-Help, a program developed in the United States to support families to directly activate a RRT if they have concerns about their child's condition. Research has evaluated the appropriateness of calls that were made by patients or relatives (32,88–91) but has not considered why calls were *not* made (67). Involving family members in escalation demands vigilance, requiring them to take a proactive and interactive role with staff with potentially some degree of confrontation, particularly if challenging the appropriateness of decisions taken (70). Families need both cognitive and emotional resources to raise concerns that involve negotiating hierarchies and boundaries (34,67). The literature points to a degree of professional

For peer review only - http://bmjopen.bmj.com/site/about/guidelines.xhtml

resistance to family involvement in activation, with reports of physician concern that their role would be undermined, that resources would be stretched with an increase in calls and that it might divert attention away from those in need (68,89,92,93) although these fears are not supported by the evidence (68,92). See Table 6 for a summary of the evidence relating to the action component of the model.

SYNTHESIS & MODEL DEVELOPMENT

The literature in this field is heterogeneous and stronger on the socio-material barriers to successful afferent limb peadiatric early warning systems than it is on solutions. While a number of different single interventions have been proposed and some have been evaluated, there is limited evidence to recommend their use beyond the specific clinical-contexts described in the papers. This reflects both the weight and quality of the evidence, the extent to which paediatric systems are conditioned by the local clinical context, and also the need to attend to the relationship between system components and inteventions which work in concert not in isolation. There is also a growing realisation in the quality improvement field that an intervention that has been successful in one context does not necessarily produce the same results elsewhere which cautions against a 'one size fits all' approach (94,95).

While it is not possible to make empirical recommendations for practice, a hermeneutic review methodology enabled the generation of theoretical inferences about the core components of an optimal paediatric early warning system. These model components are logical inferences derived from an overall synthesis of the evidence, informed by our theoretical framework and clinical expertise. These are presented as a propositional model conceptualised as three sub-systems: detection, preparation and action (see Table 2).

For peer review only - http://bmjopen.bmj.com/site/about/guidelines.xhtml

DISCUSSION

This paper reports on one of three linked reviews undertaken as part of a wider UK study commissioned to develop and evaluate an evidence-based national paediatric early warning system (95). Drawing on TMT and NPT we have synthesised and analysed the findings from the review to develop a propositional model to specify the core components of optimal afferent limb paediatric early warning systems. While there is a growing consensus of the need to think beyond TTTs to consider the whole system, no frameworks exist to support such an approach. Clinical teams wishing to improve rescue trajectories should take a whole systems perspective focused on the constellation of factors necessary to support detection, preparation and action and consider how these relationships can be managed in their local setting. TTTs have value in paediatric early warning systems but they are not the sole solution and depend on certain preconditions for their use. An emerging literature highlights the importance of preparation and indicates that combinations of interventions may facilitate situation awareness. Professional judgement is also important in detecting and acting on deterioration and the evidence points to the importance of a wider organisational culture that is supportive of this. Innovative approaches are needed to support family involvement in all aspects of paediatric early warning systems, which are sensitive to the cognitive and emotional resources this requires. System effectiveness requires attention to the socio-material relationships in the local context, senior support and leadership and continuous monitoring and evaluation. New technologies, such as moving from paper based to electronic TTTs, has important implications for all three sub-systems and critical consideration should be given to their wider impacts and the preconditions for their integration into practice.

CONCLUSION

For peer review only - http://bmjopen.bmj.com/site/about/guidelines.xhtml

Failure to recognise and act upon signs of deterioration is an acknowledged safety concern (1) and TTTs are a common response to this problem. There is, however, a growing recognition of the importance of wider system factors on the effectiveness of responses to deterioration (5) (7). We have reviewed a wide literature and analysed this using social theories to develop a propositional model of an optimal afferent limb paediatric early warning system that can be used as a framework for paediatric units to evaluate their current practices and identify areas for improvement. TTT use should be driven by the extent to which teams think that they will help improve the effectiveness of their system as a whole.

For peer review only - http://bmjopen.bmj.com/site/about/guidelines.xhtml

FOOTNOTES

Contributors

NJ: screening and review of papers, contribution to design of work and model development, preparation of manuscript; YM: screening and review of papers, contribution to model development, review of manuscript; AL: led the model development (with DA), contributed to the drafting of the manuscript; MM: conceived and led the systematic search strategies; LT, GS, CP, DR: screening and review of papers, contributed clinical expertise, contributed to the model development, contributed to the drafting of the manuscript; RT: contributed to literature searches; contributed to model development; contributed to the drafting of the manuscript; KH: contributed to model development; contributed to the drafting of the manuscript; DA: conceived of and designed the review, led the theoretical framing and analysis; screening and review of papers, led the model development (with AL) and led the writing of the manuscript (with NJ)

Acknowledgements

We would like to acknowledge the contribution of Dr Heather Strange to the review.

Competing interests

None declared.

Funding

This study is funded by the National Institute for Health Research (NIHR) Health Services and Delivery Research (HS&DR) programme (12/178/17).

Data sharing statement

For peer review only - http://bmjopen.bmj.com/site/about/guidelines.xhtml

No additional data are available.

For peer review only

For peer review only - http://bmjopen.bmj.com/site/about/guidelines.xhtml

REFERENCES

1. Subbe, C. P. and Welch JR. Failure to rescue: using rapid response systems to improve care of the deteriorating patient in hospital. J Patient Saf Risk Manag. 2013;19(1).

2. Chapman SM, Wray J, Oulton K, Peters MJ. Systematic review of paediatric track and trigger systems for hospitalised children. Resuscitation [Internet]. 2016;109:87–109. Available from: http://dx.doi.org/10.1016/j.resuscitation.2016.07.230

3. Trubey, R. Huang, C. Lugg-Widger, F. Hood, K. Allen, D. Edwards, D. Lacy, D. Lloyd, A. Mann, M. Mason, B. Oliver, A. Roland, D. Sefton, G. Skone, R. Thomas-Jones, E. Tume, L. Powell C. Validity and effectiveness of paediatric early warning systems and track and trigger tools for identifying and reducing clinical deterioration in hospitalised children: a systematic review. BMJ Open.

4. Parshuram CS, Dryden-Palmer K, Farrell C, Gottesman R, Gray M, Hutchison JS, et al. Effect of a pediatric early warning system on all-cause mortality in Hospitalized pediatric patients: The epoch randomized clinical trial. JAMA - J Am Med Assoc. 2018;319(10):1002–12.

5. The Irish Paediatric Early Warning System (PEWS): National Clinical Guideline No. 12. Dep Heal Irel (An Roinn Slainte). 2015;(12).

6. Rihari-Thomas J, Newton PJ, Sibbritt D, Davidson PM. Rapid response systems: where we have come from and where we need to go? J Nurs Manag. 2018;26(1):1–2.

For peer review only - http://bmjopen.bmj.com/site/about/guidelines.xhtml

7. Marie M, Mo TOS. A systematic literature review to support the development of a National Clinical Guideline – Paediatric Early Warning System (PEWS). 2014;

8. Almblad AC, Siltberg P, Engvall G, Målqvist M. Implementation of Pediatric Early Warning Score; Adherence to Guidelines and Influence of Context. J Pediatr Nurs. 2018;38:33–9.

9. Lambert V, Matthews A, MacDonell R, Fitzsimons J. Paediatric early warning systems for detecting and responding to clinical deterioration in children: A systematic review. BMJ Open. 2017;7(3).

10. Boell SK, Cecez-Kecmanovic D. A hermeneutic approach for conducting literature reviews and literature searches. Commun Assoc Inf Syst. 2014;34(12):257–86.

11. Thomas-Jones E, Lloyd A, Roland D, Sefton G, Tume L, Hood K, et al. A prospective, mixed-methods, before and after study to identify the evidence base for the core components of an effective Paediatric Early Warning System and the development of an implementation package containing those core recommendations for use in th. BMC Pediatr [Internet]. 2018;18(1):244. Available from: http://www.biomedcentral.com/bmcpediatr/%0Ahttp://ovidsp.ovid.com/ovidweb.cgi?T=JS&PAGE=reference&D=emexb&NEWS=N&AN=623186190

12. Greenhalgh T, A'Court C, Shaw S. Understanding heart failure; explaining telehealth - A hermeneutic systematic review. BMC Cardiovasc Disord. 2017;17(1):1–16.

For peer review only - http://bmjopen.bmj.com/site/about/guidelines.xhtml

13. Allen D, May C. Organizing Practice and Practicing Organization: An Outline of Translational Mobilization Theory. SAGE Open. 2017;7(2):35–43.

14. Allen D. Translational Mobilisation Theory: A new paradigm for understanding the organisational elements of nursing work. Int J Nurs Stud. 2018;79(April 2017):36–42.

15. May C, Finch T. Implementing, embedding, and integrating practices: An outline of normalization process theory. Sociology. 2009;43(3):535–54.

16. May CR, Johnson M, Finch T. Implementation, context and complexity. Implement Sci. 2016;11(1):1–12.

17. Jagt EW van der. Improving Pediatric Survival from Resuscitation Events: The Role and Organization of Hospital-based Rapid Response Systems and Code Teams. Curr Pediatr Rev [Internet]. 2013;9(2):158–74. Available from: http://www.eurekaselect.com/openurl/content.php?genre=article&issn=1573-3963&volume=9&issue=2&spage=158

18. Andrews T, Waterman H. Packaging: A grounded theory of how to report physiological deterioration effectively. J Adv Nurs. 2005;52(5):473–81.

19. Azzopardi P, Kinney S, Moulden A, Tibballs J. Attitudes and barriers to a Medical Emergency Team system at a tertiary paediatric hospital. Resuscitation. 2011;82(2):167–74.

20. Bonafide CP, Roberts KE, Weirich CM, Paciotti B, Tibbetts KM, Keren R, et al. Beyond statistical prediction: Qualitative evaluation of the mechanisms by which pediatric early warning scores impact patient safety. J Hosp Med. 2013;8(5):248–53.

For peer review only - http://bmjopen.bmj.com/site/about/guidelines.xhtml

21. Braaten JS. Hospital System Barriers to Rapid Response Team Activation: A Cognitive Work Analysis. Am J Nurs. 2015;115(2):22–32.

22. Ennis L. Paediatric early warning scores on a children's ward: a quality improvement initiative. Nurs Child Young People. 2014;26(7):25–31.

23. Kaul M, Snethen J, Kelber ST, Zimmanck K, Maletta K, Meyer M. Implementation of the Bedside Paediatric Early Warning System (BedsidePEWS) for nurse identification of deteriorating patients. J Spec Pediatr Nurs. 2014;19(4):339–49.

24. Lobos AT, Fernandes R, Ramsay T MJ. Patient characteristics and disposition after pediatric medical emergency team (MET) activation: disposition depends on who activates the team. 2014. p. 99–105.

25. McDonnell A, Tod A, Bray K, Bainbridge D, Adsetts D, Walters S. A before and after study assessing the impact of a new model for recognizing and responding to early signs of deterioration in an acute hospital. J Adv Nurs. 2013;69(1):41–52.

26. Mackintosh N, Rainey H, Sandall J. Understanding how rapid response systems may improve safety for the acutely ill patient: learning from the frontline. BMJ Qual Saf. 2012;21(2):135–44.

27. Monaghan A. Detecting and managing deterioration in children. Paediatr Care. 2005;17(1):32–5.

28. Pattison N, Eastham E. Critical care outreach referrals: A mixed-method investigative study of outcomes and experiences. Nurs Crit Care. 2012;17(2):71–82.

For peer review only

29. Pearson G, Duncan H. Early warning systems for identifying sick children. Paediatr Child Health (Oxford). 2011;21(5):230–3.

30. Shearer B, Marshall S, Buist MD, Finnigan M, Kitto S, Hore T, et al. What stops hospital clinical staff from following protocols? An analysis of the incidence and factors behind the failure of bedside clinical staff to activate the rapid response system in a multi-campus Australian metropolitan healthcare service. BMJ Qual Saf. 2012;21(7):569–75.

31. Stewart J, Carman M, Spegman A, Sabol VK. Evaluation of the effect of the modified early warning system on the nurse-led activation of the rapid response system. J Nurs Care Qual. 2014;29(3):223–9.

32. Van Voorhis KT, Willis TS. Implementing a Pediatric Rapid Response System to Improve Quality and Patient Safety. Pediatr Clin North Am. 2009;56(4):919–33.

33. Wheatley I. The nursing practice of taking level 1 patient observations. Intensive Crit Care Nurs. 2006;22(2):115–21.

34. Mccabe A, Duncan H, Heward Y. Paediatric early warning systems: where do we go from here? Paediatr Nurs. 2009;21(1):14–7.

35. Chua WL, Mackey S, Ng EKC, Liaw SY. Front line nurses' experiences with deteriorating ward patients: A qualitative study. Int Nurs Rev. 2013;60(4):501–9.

36. James J, Butler-Williams C, Hunt J, Cox H. Vital signs for vital people: An exploratory study into the role of the Healthcare Assistant in recognising, recording and responding to the acutely ill patient in the general ward setting. J

For peer review only

For peer review only - http://bmjopen.bmj.com/site/about/guidelines.xhtml

Nurs Manag. 2010;18(5):548–55.

37. Mackintosh N, Humphrey C, Sandall J. The habitus of "rescue" and its significance for implementation of rapid response systems in acute health care. Soc Sci Med. 2014;120(July 2015):233–42.

38. Endacott R, Westley M. Managing patients at risk of deterioration in rural hospitals: A qualitative study. Aust J Rural Health. 2006;14(6):275–9.

39. Lydon S, Byrne D, Offiah G, Gleeson L, O'Connor P. A mixed-methods investigation of health professionals' perceptions of a physiological track and trigger system. BMJ Qual Saf. 2016;25(9):688–95.

40. Cioffi J, Salter C, Wilkes L, Vonu-Boriceanu O, Scott J. Clinicians' responses to abnormal vital signs in an emergency department. Aust Crit Care. 2006;19(2):66–72.

41. Endacott R, Kidd T, Chaboyer W, Edington J. Recognition and communication of patient deterioration in a regional hospital: A multi-methods study. Aust Crit Care. 2007;20(3):100–5.

42. Hands C, Reid E, Meredith P, Smith GB, Prytherch DR, Schmidt PE, et al. Patterns in the recording of vital signs and early warning scores: Compliance with a clinical escalation protocol. BMJ Qual Saf. 2013;22(9):719–26.

43. Watson A, Skipper C, Steury R, Walsh H, Levin A. Inpatient nursing care and early warning scores: A workflow mismatch. J Nurs Care Qual. 2014;29(3):215–22.

44. Jensen CS, Nielsen PB, Olesen HV, Kirkegaard H, Aagaard H. Pediatric Early

For peer review only

Warning Score Systems, Nurses Perspective - A Focus Group Study. J Pediatr Nurs. 2018;

45. MacKintosh N, Berridge EJ, Freeth D. Supporting structures for team situation awareness and decision making: Insights from four delivery suites. J Eval Clin Pract. 2009;15(1):46–54.

46. Cioffi J. Nurses' experiences of making decisions to call emergency assistance to their patients. J Adv Nurs. 2000;32(1):108–14.

47. Adelstein B-A, Piza MA, Nayyar V, Mudaliar Y, Klineberg PL, Rubin G. Rapid response systems: A prospective study of response times. J Crit Care. 2011;26(6):635.e11-635.e18.

48. S Astroth K, M Woith W, Stapleton SJ, Degitz RJ, Jenkins SH. Qualitative exploration of nurses' decisions to activate rapid response teams. J Clin Nurs. 2013;22(19–20):2876–82.

49. Cioffi J. Recognition of patients who require emergency assistance: A descriptive study. Hear Lung J Acute Crit Care. 2000;29(4):262–8.

50. Mackintosh N, Watson K, Rance S, Sandall J. Value of a modified early obstetric warning system (MEOWS) in managing maternal complications in the peripartum period: An ethnographic study. BMJ Qual Saf. 2014;23(1):26–34.

51. Davies O, DeVita MA, Ayinla R, Perez X. Barriers to activation of the rapid response system. Resuscitation. 2014;85(11):1557–61.

52. McCrory MC, Aboumatar H, Custer JW, Yang CP, Hunt EA. "aBC-SBAR" training improves simulated critical patient hand-off by pediatric interns. Pediatr

For peer review only - http://bmjopen.bmj.com/site/about/guidelines.xhtml

Emerg Care. 2012;28(6):538–43.

53. Randhawa S, Roberts-Turner R, Woronick K, DuVal J. Implementing and sustaining evidence-based nursing practice to reduce pediatric cardiopulmonary arrest. West J Nurs Res. 2011;33(3):443–56.

54. Salamonson Y, Heere B van, Everett B, Davidson P. Voices from the floor: Nurses' perceptions of the medical emergency team. Intensive Crit Care Nurs. 2006;22(3):138–43.

55. Mohammmed Iddrisu S, Hutchinson AF, Sungkar Y, Considine J. Nurses role in recognising and responding to clinical deterioration. J Clin Nurs. 2018;0–3.

56. Demmel KM, Williams L, Flesch L. Implementation of the pediatric early warning scoring system on a pediatric Hematology/Oncology unit. J Pediatr Oncol Nurs. 2010;27(4):229–40.

57. McKay H, Mitchell IA, Sinn K, Mugridge H, Lafferty T, Van Leuvan C, et al. Effect of a multifaceted intervention on documentation of vital signs and staff communication regarding deteriorating paediatric patients. J Paediatr Child Health. 2013;49(1):48–56.

58. Radeschi G, Urso F, Campagna S, Berchialla P, Borga S, Mina A, et al. Factors affecting attitudes and barriers to a medical emergency team among nurses and medical doctors: A multi-centre survey. Resuscitation. 2015;88:92–8.

59. Brady PW, Goldenhar LM. A qualitative study examining the influences on situation awareness and the identification, mitigation and escalation of recognised patient risk. BMJ Qual Saf. 2014;23(2):153–61.

For peer review only - http://bmjopen.bmj.com/site/about/guidelines.xhtml

60. Donohue LA, Endacott R. Track, trigger and teamwork: Communication of deterioration in acute medical and surgical wards. Intensive Crit Care Nurs. 2010;26(1):10–7.

61. Claussen D, Garner D, Crow B. Early warning signs and the EHR: At the intersection of technology and care. Nurs Manage. 2013;44(11):14–6.

62. Jones D, Baldwin I, McIntyre T, Story D, Mercer I, Miglic A, et al. Nurses' attitudes to a medical emergency team service in a teaching hospital. Qual Saf Heal Care. 2006;15(6):427–32.

63. Massey D, Chaboyer W, Aitken L. Nurses' perceptions of accessing a Medical Emergency Team: A qualitative study. Aust Crit Care. 2014;27(3):133–8.

64. Burns KA, Reber T, Theodore K, Welch B, Roy D, Siedlecki SL. Enhanced Early Warning System Impact on Nursing Practice: A phenomenological study. J Adv Nurs. 2017;(December 2017):1–7.

65. Hope J, Recio-Saucedo A, Fogg C, Griffiths P, Smith GB, Westwood G, et al. A fundamental conflict of care: Nurses' accounts of balancing patients' sleep with taking vital sign observations at night. J Clin Nurs. 2018;(December 2017).

66. Rainey H, Ehrich K, Mackintosh N, Sandall J. The role of patients and their relatives in "speaking up" about their own safety - a qualitative study of acute illness. Heal Expect. 2015;18(3):392–405.

67. Mackintosh, NJ, Davis, RE, Easter, A, Rayment-Jones, H, Sevdalis, N, Wilson, S, Adams, M, Sandall J. Interventions to increase patient and family involvement in escalation of care for acute life-threatening illness in community health and hospital settings (Protocol). Cochrane Database Syst Rev. 2017;(10):CD012829.

For peer review only - http://bmjopen.bmj.com/site/about/guidelines.xhtml

68. Paciotti, B, Roberts, KE, Tibbetts, KM, Weirich Paine, C, Keren, R, Barg, F.K., Holmes, J.H. and Bonafide CP. Physician attitudes toward family-activated medical emergency teams for hospitalized children. Jt Comm J Qual Patient Saf. 2014;40(4):187–92.

69. Graedon J, Graedon T. Enlisting Families as Patient Safety Allies. Clin Pediatr Emerg Med. 2006;7(4):265–7.

70. Entwistle V. Nursing shortages and patient safety problems in hospital care: Is clinical monitoring by families part of the solution? Heal Expect. 2004;7(1):1–5.

71. Mohammed M, Hayton R, Clements G, Smith G, Prytherch D. Improving accuracy and efficiency of early warning scores in acute care. Br J Nurs. 2009;18(1):18–24.

72. Jones S, Mullally M, Ingleby S, Buist M, Bailey M, Eddleston JM. Bedside electronic capture of clinical observations and automated clinical alerts to improve compliance with an Early Warning Score protocol. Crit Care Resusc. 2011;13(2):83–8.

73. Schmidt PE, Meredith P, Prytherch DR, Watson D, Watson V, Killen RM, et al. Impact of introducing an electronic physiological surveillance system on hospital mortality. BMJ Qual Saf. 2015;24(1):10–20.

74. Bellomo R, Ackerman M, Bailey M, Beale R, Clancy G, Danesh V, et al. A controlled trial of electronic automated advisory vital signs monitoring in general hospital wards. Crit Care Med. 2012;40(8):2349–61.

75. Subbe CP, Duller B, Bellomo R. Effect of an automated notification system for

For peer review only

For peer review only - http://bmjopen.bmj.com/site/about/guidelines.xhtml

deteriorating ward patients on clinical outcomes. Crit Care. 2017;21(1):1–9.

76. Bonafide CP, Zander M, Graham CS, Weirich Paine CM, Rock W, Rich A, et al. Video methods for evaluating physiologic monitor alarms and alarm responses. Biomed Instrum Technol. 2014;48(3):220–30.

77. Fagan K, Sabel A, Mehler PS, MacKenzie TD. Vital Sign Abnormalities, Rapid Response, and Adverse Outcomes in Hospitalized Patients. Am J Med Qual. 2012;27(6):480–6.

78. Wager KA, Schaffner MJ, Foulois B, Swanson Kazley A, Parker C, Walo H. Comparison of the quality and timeliness of vital signs data using three different data-entry devices. CIN - Comput Informatics Nurs. 2010;28(4):205–12.

79. Stevenson JE, Nilsson G. Nurses' perceptions of an electronic patient record from a patient safety perspective: A qualitative study. J Adv Nurs. 2012;68(3):667–76.

80. Goldenhar LM, Brady PW, Sutcliffe KM, Muething SE. Huddling for high reliability and situation awareness. BMJ Qual Saf. 2013;22(11):899–906.

81. Brady PW, Muething S, Kotagal U, Ashby M, Gallagher R, Hall D, et al. Improving Situation Awareness to Reduce Unrecognized Clinical Deterioration and Serious Safety Events. Pediatrics. 2013;131(1):e298–308.

82. Pezzolesi C, Manser T, Schifano F, Kostrzewski A, Pickles J, Harriet N, et al. Human factors in clinical handover: Development and testing of a "handover performance tool" for doctors' shift handovers. Int J Qual Heal Care. 2013;25(1):58–65.

For peer review only - http://bmjopen.bmj.com/site/about/guidelines.xhtml

83. Abraham J, Kannampallil T, Patel B, Almoosa K, Patel VL. Ensuring patient safety in care transitions: an empirical evaluation of a Handoff Intervention Tool. AMIA Annu Symp Proc. 2012;2012:17–26.

84. Donahue M, Smith L, Dykes P, Fitzpatrick JJ. Phase 2 of the EMPOWER project: enhancing communication for paraprofessionals. J Contin Educ Nurs. 2010;41(5):197–8.

85. Mullan PC, Macias CG, Hsu D, Alam S, Patel B. A novel briefing checklist at shift handoff in an emergency department improves situational awareness and safety event identification. Pediatr Emerg Care. 2015;31(4):231–8.

86. Weiss MJ, Bhanji F, Fontela PS, Razack SI. A preliminary study of the impact of a handover cognitive aid on clinical reasoning and information transfer. Med Educ. 2013;47(8):832–41.

87. Wong D, Bonnici T, Knight J, Morgan L, Coombes P, Watkinson P. SEND: A system for electronic notification and documentation of vital sign observations. BMC Med Inform Decis Mak. 2015;15(1):1–12.

88. Bobert S, Ferrell C, Rutledge DN. Experience with Family Activation Of Rapid Response Teams. Medsurg Nurs. 2010;19(4):215–22.

89. Dean BS, Decker MJ, Hupp D, Urbach AH, Lewis E, Benes-Stickle J. Condition HELP: a pediatric rapid response team triggered by patients and parents. J Healthc Qual. 2008;30(3):28–31.

90. Greenhouse PK, Kuzminsky B, Martin SC, Merryman T. Calling a condition H(elp). Am J Nurs. 2006;106(11):63–6.

For peer review only

For peer review only - http://bmjopen.bmj.com/site/about/guidelines.xhtml

91. Hueckel RM, Mericle JM, Frush K, Martin PL, Champagne MT. Implementation of condition help: Family teaching and evaluation of family understanding. J Nurs Care Qual. 2012;27(2):176–81.

92. Brady PW, Zix J, Brilli R, Wheeler DS, Griffith K, Giaccone MJ, et al. Developing and evaluating the success of a family activated medical emergency team: A quality improvement report. BMJ Qual Saf. 2015;24(3):203–11.

93. Gerdik C, Vallish RO, Miles K, Godwin SA, Wludyka PS, Panni MK. Successful implementation of a family and patient activated rapid response team in an adult level 1 trauma center. Resuscitation. 2010;81(12):1676–81.

94. Bion J, Richardson A, Hibbert P, Beer J, Abrusci T, McCutcheon M, et al. Matching Michigan: A 2-year stepped interventional programme to minimise central venous catheterblood stream infections in intensive care units in England. BMJ Qual Saf. 2013;22(2):110–23.

95. Dixon-Woods M, Leslie M, Tarrant C, Bion J. Explaining Matching Michigan: An ethnographic study of a patient safety program. Implement Sci. 2013;8(1):1

For peer review only

TABLES

BOX 1: MECHANISMS OF TRANSLATIONAL MOBILISATION AND THEIR APPLICATION TO RESCUE TRAJECTORIES (ALLEN 2018)

Object formation - how people draw on the interpretative resources available to them within a strategic action field to create the objects of their practice. Enrolment into an escalation trajectory requires multiple examples of object formation beginning with construction of an individual as at risk of deterioration and a regime of vital signs monitoring instigated, through recognition that the patient's physiological status is a cause for concern, to the identification of the patient as requiring a specific intervention. How this is achieved is highly dependent on the features of the local strategic action field.

Translation – the processes that enable practice objects to be shared and different understandings accommodated. It points to the actions necessary in order for a patient that is an object of concern for nursing staff to be translated into a clinical priority for the doctor and, if necessary, to be translated into the focus of intervention by the emergency response team.

Articulation - refers to the secondary work processes that align the actions, knowledge and resources necessary for the mobilization of projects of collective action. It is the work that makes the work, work. Responding to deterioration is time critical and articulation work is necessary to ensure the availability of resources and materials to support clinical management. This is not a mundane observation; catastrophic failures in patient safety are often attributed to the lack of functioning

For peer review only - http://bmjopen.bmj.com/site/about/guidelines.xhtml

equipment (Anon 2008) and the absence of monitoring equipment has been identified as a factor undermining the implementation of early warning track and trigger tools (Cioffi 2000a).Attending to articulation in rescue trajectories also underlines the temporal ordering of action and the mechanisms required to achieve this, directing improvement efforts towards the organization's escalation policy, for example.

Reflexive monitoring refers to the processes through which people collectively or individually appraise and review activity. In a distributed field of action, reflexive monitoring is the means through which members accomplish situational awareness (Gilson 1995) of an overall project. The importance of situation awareness in rescue trajectories is well recognised, but achieving this is challenging. Reflexive monitoring is conditioned by the wider institutional context which will include a multiplicity of informal and formal mechanisms designed for this purpose: nursing and medical handovers, the ward round, safety briefings. The form, frequency and effectiveness of these processes in supporting detecting and acting on deterioration would need to be taken into account in any improvement initiative.

Sensemaking - refers to the processes through which agents create order in conditions of complexity. It draws attention to how the material and discursive processes by which members organise their work, account for their actions, and construct the objects of their practice also give meaning and substance to the institutional components of strategic action fields that shape activity and condition future activity.

For peer review only - http://bmjopen.bmj.com/site/about/guidelines.xhtml

TABLE 1 PRISMA DIAGRAM

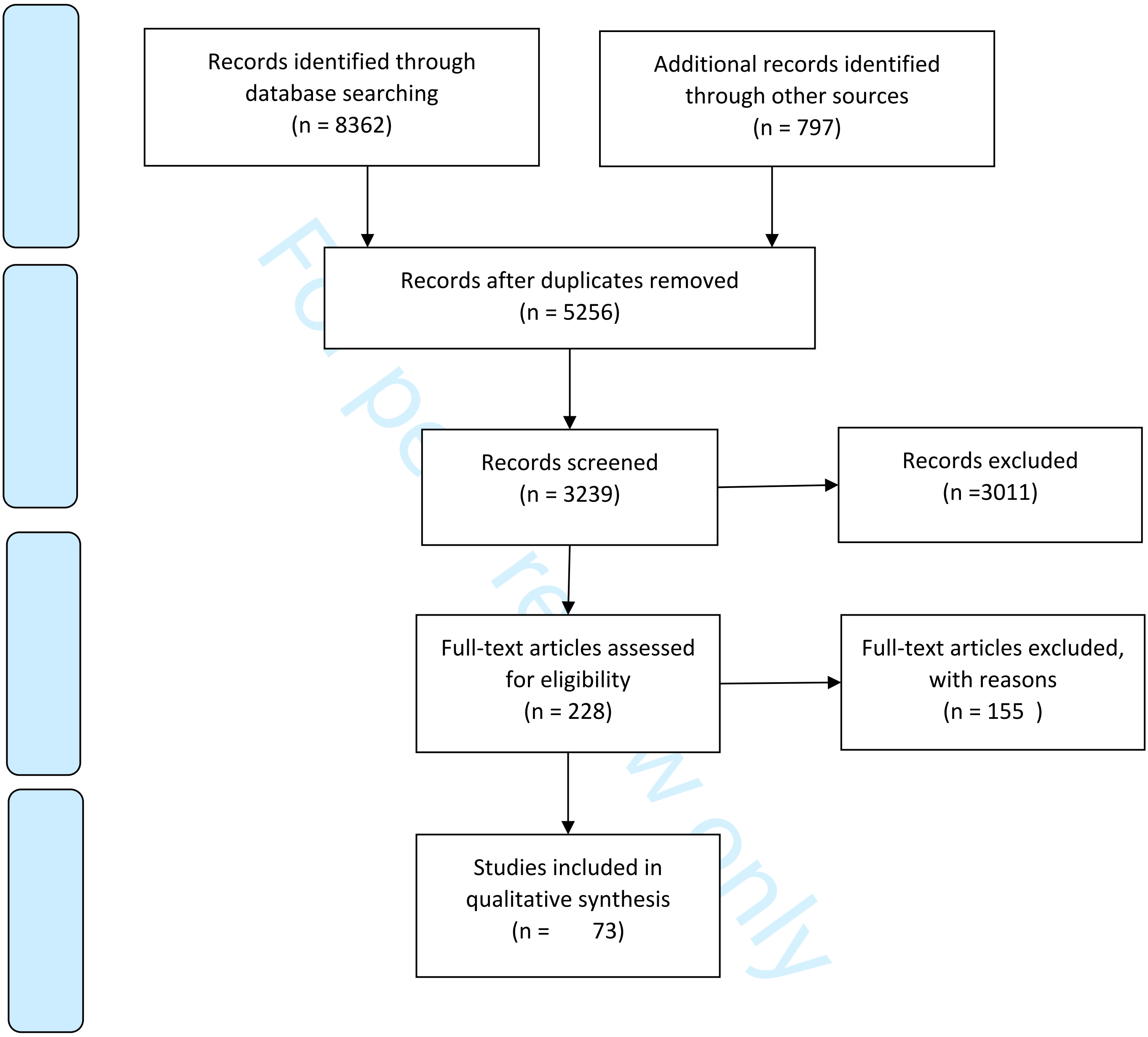

For peer review only - http://bmjopen.bmj.com/site/about/guidelines.xhtml

TABLE 2: PROPOSITIONAL MODEL

	Proposition	Conceptual requirements	Operationalisation	Tool and activities	Proposed effect of interventions
Detect	Detection of deterioration depends on collecting and interpreting information from children, through accurate, timely and appropriate monitoring of vital signs and relevant risk factors.	At a minimum, this requires:<br>• Staff are aware of which core and additional vital signs need to be monitored<br>• Staff are aware of the minimum frequency of observations required for the children in their care<br>• Staff are aware of the need to review the frequency of observations for children in their care<br>• Staff are aware of prior factors that increase children's risk of deterioration (e.g. premature birth)<br>• Staff are aware of additional clinical assessments required for children with prior risk factors<br>• Staff are aware of which additional clinical assessments are appropriate for children in their care	At a minimum, this requires:<br>• Tasks are allocated to staff members with appropriate skills to conduct them<br>• Staff receive guidance about:<br>○ Which core and additional vital signs need to be monitored<br>○ Minimum frequency of observations required for children in their care<br>○ Prior factors that increase children's risk of deterioration<br>○ Which additional clinical assessments are appropriate for children in their care with prior risk factors<br>○ Which members and grades of staff are responsible for monitoring and recording vital signs	• Skills training: accurate, timely and appropriate monitoring<br>• Focused education: recognising signs of deterioration<br>• Induction: staff aware of monitoring processes in a specific ward<br>• Observations policy<br>• Track and Trigger Tool<br>• Regular equipment	• Staff are aware of which core and additional indicators need to be monitored<br>• Staff are aware of the frequency of observations required for the children in their care<br>• Staff have appropriate skills to regularly monitor and accurately record key physiological parameters<br>• Staff are aware of roles and responsibilities<br>• Observations of key physical parameters are structured, accumulated and displayed so that patterns and trends can be readily detected and staff are prompted to make observations at the required frequency.

For peer review only

For peer review only - http://bmjopen.bmj.com/site/about/guidelines.xhtml

		with prior risk factors<br>• Staff have appropriate skills to monitor and accurately record vital signs<br>• Staff have access to appropriate equipment to monitor and accurately record vital signs, and conduct other clinical assessments<br>• Staff are aware of roles and responsibilities<br>• Staff have time to conduct accurate timely and appropriate monitoring of vital signs, alongside other work commitments<br>• Staff use a standardised approach to assessing illness severity	○ How to record and accumulate observations (e.g. use of a device)<br>• Staff discuss and agree appropriate frequency of observations for individual patients<br>• Equipment required to monitor and accurately record vital signs is readily available<br>• Equipment required to monitor and accurately record vital signs is functioning as it should<br>• There are an appropriate number of staff to carry out required tasks<br>• Staff use a device for recording and accumulating key vital signs	checks	

For peer review only - http://bmjopen.bmj.com/site/about/guidelines.xhtml

Detection	Detection of deterioration depends on supporting and enabling staff to detect other signs of deterioration and express concerns	At a minimum this requires:<br>• Teams have appropriate skills to discern patterns and trends of signs and symptoms<br>• Work is organised to enable staff to recognise patterns and trends<br>• Staff are encouraged and supported in raising concerns<br>• Staff are encouraged and supported to share learning	At a minimum this requires:<br>• Work to be organised to facilitate continuity of care<br>• Work to be organised to facilitate communication if continuity of care is breached<br>• Staff concern formally recognised as a valid indicator of deterioration<br>• Staff concerns are positively received and acted upon, irrespective of the outcome<br>• Staff receive guidance on how to raise concerns about patient deterioration not necessarily related to observations	• Routine opportunities for more experienced staff to coach those less experienced (clinical supervision)<br>• Skills training: working as a team<br>• Track and Trigger Tool: staff concern a core parameter	• Less experienced staff learn pattern recognition from more experienced staff<br>• Staff respect and value each other's opinion<br>• Staff recognise concern as a valid indicator of deterioration<br>• Staff concern included as a key parameter in the detection of deterioration

For peer review only

For peer review only - http://bmjopen.bmj.com/site/about/guidelines.xhtml

Detection	Detection of deterioration depends on supporting and enabling families to detect other signs of deterioration and express concerns	At a minimum this requires:<br>• Staff understand the value of family concerns in the detection of deterioration<br>• Families are in a position to discern patterns of signs and symptoms in their child<br>• Families are encouraged and supported in raising concerns<br>• Parents can voice their concerns	At a minimum this requires:<br>• Families are supported to stay with their children<br>• Families are involved with defining normal parameters for their child and tasked with identifying changes Parents are supported to articulate their concerns<br>• Staff keep families informed about developments in care and treatment<br>• Staff receive guidance about how to respond to family concern	• Parent information leaflets and posters<br>• Skills training: family engagement<br>• "Planning care together" forms<br>• "What to expect" script<br>• Track and Trigger Tool: parent concern a core parameter	• Parents are informed that their views matter, and provided guidance about how to share their concerns with staff<br>• Staff respect and value family engagement<br>• Staff recognise family concern as a valid indicator of deterioration<br>• Parents and staff can share, discuss and document parental concerns.<br>• Parents receive guidance about how to articulate their concerns
	**Proposition**	**Conceptual requirements**	**Operationalisation**	**Interventions**	**Proposed effect of interventions**
	Preparation depends on collating and sharing indicators of deterioration for each patient.	At a minimum this requires:<br>• Information from children, staff and families is integrated<br>• Staff are in a position to review and interpret information from children, families and staff Identifying children that need to be watched	At a minimum this requires:<br>• Information from children, staff and families is interpreted together<br>• Regular opportunities for communication between relevant team members	• Skills training: working as a team<br>• "Watcher"	• Staff develop the skills and behaviours required for effective communication, coordination and collaboration and understand their purpose and importance.<br>• A formalised process where the bedside nurse, and clinician, proactively identify

For peer review only - http://bmjopen.bmj.com/site/about/guidelines.xhtml

				• Common information space<br><br>• Structured and standardised handover<br><br>• Huddles	risk, which includes assessment of information from children, families and staff<br><br>• Information on deterioration from children, staff and families are brought together, displayed together, regularly updated and easily accessible<br><br>• Key information from children, staff and families is systematically shared in a structured method, during handovers, between shifts or between staff in the same or different clinical areas.<br><br>• Senior staff allocated responsibility for knowing which patients are at high risk of deterioration meet with those responsible for conducting observations and liaise with families to discuss plans for individual patient management

For peer review only

**Preparation**	Preparation depends on staff being aware at ward level of the status of individual patients and the availability of skills and resources, and being able to plan appropriate response.	At a minimum this requires:<br>• There is a regular mechanism for reviewing the status of all children in the ward<br>• The is a regular mechanism for reviewing staffing levels and skills mix, workload, acuity and admissions<br>• There is a regular mechanism for communicating the review of all children, staffing levels and other resources to the rest of the team<br>• There is a regular mechanism for planning appropriate response<br>• Staff are aware of at-risk children and response plans<br>• Staff have situational awareness appropriate to their seniority (e.g. individual child or ward level)	At a minimum this requires:<br>• Information from children, staff and families is brought together<br>• Information from individual patient assessments are brought together and kept up to date<br>• Senior staff members are allocated responsibility for assessing staff levels and resources<br>• Senior staff members are allocated responsibility for managing demand and resources<br>• Senior staff members are allocated responsibility for communicating response plans	• Skills training: working as a team<br>• Common information space<br>• Structured and standardised handover<br>• Huddles<br>• Watch-stander	• Staff develop the skills and behaviours required for effective communication, coordination and collaboration and understand their purpose and importance.<br>• Information on deterioration (physiological, staff concern, family concern) are brought together, displayed together, regularly updated and easily accessible<br>• Key information about patients, staffing levels and other resources is systematically recorded and shared<br>• Senior staff allocated responsibility for knowing which patients are at high risk of deterioration meet with those who oversee patient flow and staffing to discuss plans for overall patient management
	**Proposition**	**Conceptual requirements**	**Operationalisation**	**Interventions**	**Proposed effect of interventions**
	Escalation depends on	At a minimum this requires:<br>• A trigger or prompt to act	At a minimum this requires:<br>• Staff receive guidance about	• Activation policy	• All staff and parents are aware of escalation and

For peer review only - http://bmjopen.bmj.com/site/about/guidelines.xhtml

	defining escalation and response processes	• Clearly defined graded escalation and response procedures – agreed at organisational level<br>• Staff aware of roles and responsibilities<br>• Staff understand their part in the escalation procedure as activators and responders	graded escalation and response procedures<br>• Staff receive guidance on which members of staff are responsible for activating and responding<br>• Staff receive guidance about how to escalate	• Activation criteria displayed throughout hospital<br>• Staff induction<br>• Track and Trigger Tool	response criteria<br>• All staff and parents are aware of escalation and response procedures and roles and responsibilities<br>• Alerts staff to signs of deterioration and prompts the appropriate response
	Escalation depends on effective communication across professional, disciplinary and hierarchical boundaries.	At a minimum this requires:<br>• Staff understand the significance of information<br>• Staff able to communicate information across professional hierarchies<br>• All staff are able to express concerns	At a minimum this requires:<br>• A structured approach to sharing information<br>• Staff concerns are positively received and acted upon, irrespective of the outcome	• Activation criteria displayed throughout hospital<br>• Staff induction<br>• Track and trigger tool<br>• SBAR	• All staff and parents are aware of escalation and response criteria<br>• All staff and parents are aware of escalation and response procedures and roles and responsibilities<br>• Tool packages legitimises, empowers and informs individuals when attempting to signal deterioration<br>• Time critical information about patients, staffing levels and other resources that requires immediate

For peer review only - http://bmjopen.bmj.com/site/about/guidelines.xhtml

					attention and action is shared in a structured method

For peer review only

For peer review only - http://bmjopen.bmj.com/site/about/guidelines.xhtml

TABLE 3 SCREENING BREAKDOWN

	PEWS	EWS	Structured handover	Situational awareness		Electronic systems	Observations & monitoring	Family involvement	Snowball sample
Database searching	3536	1155	3369	302		/	/	/	/
Additional sources	83	7	150	46		/	/	/	/
Records after duplicates removed	2166	751	2156	199		/	/	/	/
Hand screening/searches	431	/	/	/					
Title screening	62	751	2156	199					
Abstract screening	34	106	n/a	n/a					
Full paper screening	29	65	37	26					
Data extraction	14	26	10	6		26	20	15	6
**Included in Syntheses**	**14**	**22**	**4**	**6**		**10**	**2**	**9**	**6**

For peer review only - http://bmjopen.bmj.com/site/about/guidelines.xhtml

TABLE 4 SUMMARY OF DETECTION EVIDENCE

Author	Country	Methodology	Analysis	Search Area	Evidence Contribution
Andrews, T. and Waterman, H. (2005) Packaging: a grounded theory of how to report physiological deterioration effectively. *Journal of Advanced Nursing* 52, 5, 473–481	U.K	Interviews and observations	Grounded theory	**EWS**	Importance of 'gut feeling' in detecting deterioration<br><br>Vital sign monitoring delegated to junior staff
Astroth, K., Woith, W. M., Stapleton, S. J., Degitz, R. J., & Jenkins, S. H. (2013). Qualitative exploration of nurses' decisions to activate rapid response teams. Journal of Clinical Nursing, 22(19-20), 2876-2882	U.S.A	Semi-structured interviews with nurses	Coding categories were generated from the data, and consensus on final themes was achieved through an iterative process	**EWS**	Staff encouraged to use their intuition when activating the RRT
Azzopardi, P.; Kinney, S.; Moulden, A. & Tibbals, J. Attitudes and barriers to a Medical Emergency Team system at a tertiary paediatric hospital. *Resuscitation*, 82, 167-174	Australia	Survey	Statistical analysis	**PEWS**	Track & Trigger Tools used to confirm or identify deterioration depending on experience. Useful for junior staff<br><br>Vital sign monitoring delegated to junior staff
Bellomo, R., Ackerman, M., Bailey, M., Beale, R. Clancy, G., Danesh, V. et al . (2012) The Vital Signs to Identify, Target, and Assess Level of Care Study (VITAL Care	International: U.S.A; Sweden; U.K; Netherla	Before-and-after multi-centred international controlled trial.	Automated vital signs monitoring and early warning score (EWS) calculated, international study,	**Electronic systems**	The electronic health record (HER) provides a prompt to action

For peer review only - http://bmjopen.bmj.com/site/about/guidelines.xhtml

Author	Country	Methodology	Analysis	Search Area	Evidence Contribution
Study) Investigators.. Crit Care Med Vol. 40, No. 8; 2349–2361	nds; Australia		blinded trial, hospitals retained own EWS and escalation policy		
Bonafide C., Roberts K., Weirich C., Paciotti B., Tibbetts K., Keren R., Barg F. and Holmes J. (2013) Beyond Statistical Prediction: Qualitative Evaluation of the Mechanisms by which Pediatric Early Warning Scores Impact Patient safety. *J Hospital Medicine*, 8, 5, 248-253	U.S.A	Semi-structured interviews	Grounded theory	**PEWS**	Vital sign monitoring delegated to junior workers who may not have the knowledge to interpret results. Track & Trigger Tools used to confirm or identify deterioration depending on experience & particularly useful for junior staff<br><br>Professional intuition important for senior staff to detect deterioration
Bonafide, C. P., et al. (2014). Video Methods for Evaluating Physiologic Monitor Alarms And Alarm Responses. Biomédical Instrumentation & Technology May/June 2014, p220-230	U.S.A	Prospective Feasibility study	Video recording and electronic patient data collected prospectively. Pragmatic observational study of VitalPAC deployment in 2 large hospitals	**Electronic systems**	Alarm fatigue a barrier to escalation
Braaten, J.S. (2015). Hospital System Barriers to Rapid Response Team Activation: A Cognitive Work Analysis. *American Journal of Nursing*, 115(2), 22-32.	U.S.A	Document review and interviews using the principles of cognitive work analysis	Inductive and deductive forms of analysis – cognitive work analysis, framework and directed content analysis	**EWS**	Vital sign monitoring delegated to junior workers who may not have the knowledge to interpret results.<br><br>Track and Trigger Tools used to confirm or identify deterioration depending on experience; Professional intuition important for more senior staff/when clinical change is abrupt<br>Issues around availability of equipment and staffing

For peer review only

Author	Country	Methodology	Analysis	Search Area	Evidence Contribution
Brady P.W. and Goldenhar L.M. (2013a). A qualitative study examining the influences on situation awareness and the identification, mitigation and escalation of recognised patient risk. *BMJ Quality and SafetyI,* 0, 1-9.	U.S.A	Focus groups x7 – held in groups of participants with similar roles	Constant comparison	**Situational awareness**	Paediatric early warning score (PEWS) supplementing gut feeling but these were not standardised
Burns et al. (2018) Enhanced early warning system impact on nursing practice: A phenomenological study. *Journal of Advanced Nursing*, 1-7.	U.S.A.	Semi-structured interviews were used drawing on a descriptive phenomenological methodology	Iterative thematic analysis	**Snowball sample**	Importance of professional intuition is reported
Chua, W. L., Mackey, S., Ng, E. K. C., & Liaw, S. Y. (2013). Front line nurses' experiences with deteriorating ward patients: a qualitative study. *International Nursing Review*, 60, 4., 501-509.	Singapore	A qualitative survey using critical incident technique	Inductive content analysis	**EWS**	Vital sign monitoring is the responsibility of nurses. The regularity of this activity can lead to it being viewed as a mundane activity<br><br>Importance of 'gut feeling' and intuition in detection of deterioration
Cioffi, J. (2000). Nurses' experiences of making decisions to call emergency assistance to their patients. Journal of Advanced Nursing, 32(1), 108-114.	Australia	Unstructured interviews with nurses who had activated the medical emergency team (MET)	Simple code and retrieve	**EWS**	Importance of gut feeling and 'knowing' your patient in the detection of deterioration<br><br>Importance of having staff concern in the MET criterion
Cioffi, J. (2000). Recognition of patients who require emergency	Australia	Unstructured interviews	Simple code and retrieve	**EWS**	Importance of gut feeling and intuition in recognising deterioration<br><br>Importance of having staff concern in a calling criteria

For peer review only

Author	Country	Methodology	Analysis	Search Area	Evidence Contribution
assistance: a descriptive study. Heart & Lung, 29(4), 262-268.					
Cioffi, J., Salter, C., Wilkes, L., Vonu-Boriceanu, O., & Scott, J. (2006). Clinicians' responses to abnormal vital signs in an emergency department. Australian Critical Care, 19(2), 66-72.	Australia	Focus groups with clinicians and nurses exploring their responses to abnormal vital signs	Constant comparison	**EWS**	Intuition important, particularly for more senior staff to detect AVS. Knowing your patient reported as key; knowledge and experience seen as essential.
Claussen, D., Garner, D. and Crow, B. (2013) Early warning signs and the EHR: At the intersection of technology and care. *Nursing Management*, 44, 11, 14-16	U.S.A	Retrospective review of calls to the rapid response team (RRT) and cardiac arrest calls to evaluate impact of evidence based guidelines (pre-intervention). EWS and huddles implemented. EHR available throughout to compare pre and post	Descriptive statistics	**Electronic systems**	Professional intuition used in conjunction with MEWS
Davies, O., DeVita, M.A., Ayinla, A. and Perez, X.. (2014). Barriers to activation of the rapid response system. *Resuscitation*, 85(11), 1557-1561.	U.S.A	Survey looking at barriers to rapid response system (RRS) activation	Statistical analysis	**EWS**	Professional intuition used over RRS activation criteria – barrier to activation
Donohue, L. and Endacott, R. (2010) Track, trigger and teamwork: Communication	U.K	Qualitative design with critical incident technique.	Thematic analysis	**EWS**	Importance of gut feeling and intuition in detecting deterioration

For peer review only - http://bmjopen.bmj.com/site/about/guidelines.xhtml

Author	Country	Methodology	Analysis	Search Area	Evidence Contribution
of deterioration in acute medical and surgical wards. *Intensive and Critical Care Nursing*, 26, 10-17		Semi-structured interviews with nurses and the outreach team			
Endacott et al (2007). Recognition and communication of patient deterioration in a regional hospital: A multi-methods study. Australian Critical care 20, 100—105	U.K.	Mixed methods case study – semi structured interviews and audit of charts	Qualitative content analysis & descriptive statistics	**Obs and monitoring**	Gut feeling important – interestingly particularly for nurses whereas doctors sought additional empirical evidence to back up intuition
Endacott, R., & Westley, M. (2006). Managing patients at risk of deterioration in rural hospitals: a qualitative study. Australian Journal of Rural Health, 14(6), 275-279	Australia	Questionnaire, in-depth interviews and observations	Content analysis and constant comparison	**EWS**	Intuition and anticipatory skills important in detecting deterioration
Entwistle, V. (2004). Nursing shortages and patient safety problems in hospital care: is clinical monitoring by families part of the solution? Health Expectations, 7, pp.1–5 (editorial)	U.S.A	Editorial	N/a	**Family involvement**	Little evidence/no evaluations of policies or practices that encourage and support family involvement in clinical monitoring<br><br>Propose the innovative practice of interdisciplinary rounds where families are invited and communication is directed to the patient and family
Fagan, K.,Sabel, A., Mehler, P. S. and MacKenzie, T. D. (2012). Vital Sign Abnormalities, Rapid Response, and Adverse Outcomes in Hospitalized Patients. American Journal	U.S.A	Observational Cohort comparison study	Descriptive statistics	**Electronic systems**	Concerns about over-burdening staff with automated alerts.<br><br>When considering the patient's baseline, nursing staff are expected to notify the patient's care provider immediately responsible for the patient when the vital signs meet or exceed the clinical trigger

For peer review only - http://bmjopen.bmj.com/site/about/guidelines.xhtml

Author	Country	Methodology	Analysis	Search Area	Evidence Contribution
of Medical Quality 27(6) 480– 486					
Graedon, J. and Graedon, T. (2006) Enlisting Families as Patient Safety Allies. Clinical Paediatric *Emergency Medicine*, 265-267	U.S.A focus	Opinion piece	N/a	**Family involvement**	Parents to explain child's baseline<br><br>Need to have detailed information about signs and symptoms that they should look for that would require alerting the medical team<br><br>Propose the need for structured inclusion of family concern during ward rounds (safety walkrounds)
Hands, C. et al. (2013) Patterns in the recording of vital signs and early warning scores: compliance with a clinical escalation protocol. *Quality and Safety in Health Care*, 1-8.	U.K.	The vital signs (V.S.) and early warning data for all in-patients for one year to investigate patterns of V.S observations collected	Statistical	**Obs and Monitoring**	Staffing levels and availability of equipment impede the collection of V.S and early warning data.<br><br>Night time factors also play a role with a decrease in observations noted
Hope et al. (2017) A fundamental conflict of care: Nurses' accounts of balancing patients' sleep with taking vital sign observations at night. *Journal of Clinical Medicine*	U.K.	Semi-structured interviews with 17 registered nurses	Constant comparative method informed by grounded theory	**Snowball sample**	Wider context of night time care considered; there is some indication that staff are making a choice and prioritising sleep over monitoring
James, J., Butler-Williams, C., Hunt, J. and Cox, H. (2010) Vital signs for vital people: an exploratory study into the role of the Healthcare Assistant in recognising, recording and responding to the acutely ill patient in the general ward	U.K.	Postal survey with Healthcare Assistants (HCAs) utilising closed and open questions	Descriptive statistics & content analysis of qualitative data	**Observations and monitoring**	Factors other than the score used to detect deterioration - intuition/patient's own descriptions<br><br>Vital sign monitoring delegated to junior staff who may not have the skills to interpret results; many HCAs did not "fully understand this neurological assessment and it is implications for the acutely unwell patient thus resulting in the risk of an inaccurate EWS score" p 552.

For peer review only - http://bmjopen.bmj.com/site/about/guidelines.xhtml

Author	Country	Methodology	Analysis	Search Area	Evidence Contribution
setting. *Journal of Nursing Management*, 18, 548-555					
Jensen, C. et al. (2018) Pediatric Early Warning Score Systems, Nurses Perspective – A Focus Group Study. *Journal of Pediatric Nursing*	Denmark	Focus group exploring nurse' experiences with PEWS	Qualitative meaning condensation analysis	**Snowball sample**	Sleeping as a part of care identified<br><br>Professional intuition<br><br>Night-time pressures
Jones, D. et al. (2006). Nurses' attitudes to a medical emergency team service in a teaching hospital. Quality & Safety in Health Care, 15(6), 427-432.	Australia	Questionnaire about understandings and barriers to activating a medical emergency team (MET)	Simple descriptive statistics	**EWS**	Intuition important when detecting deterioration
Jones, S., Mullally, M., Ingleby, S., Buist, M., Bailey, M. and Eddleston, J. M. (2011). Bedside electronic capture of clinical observations and automated clinical alerts to improve compliance with an Early Warning Score protocol. Crit Care Resusc; 13: 83–88	U.K.	Electronic capture of physiological data to see if automated clinical alerts increase compliance with an early warning score (EWS) and improve patient outcomes	Statistical	**Electronic systems**	Electronic systems can provide prompts or alerts for monitoring vital signs<br><br>This system demonstrated significant improvement in clinician attendance to acutely ill patients with an early warning score of 3 and above
Kaul, M., Snethen, J., Kelber, S., Zimmanck, K., Maletta, K. and Meyer, M. (2014) Implementation of the bedside paediatric early warning system (BedsidePEWS) for nurse identification of	U.S.A	Descriptive cross sectional study; nurse and medical staff survey	Descriptive statistics	**PEWS**	Benefits of BedsidePEWS – nurses more likely to identify early signs of deterioration and a greater ability to escalate care. Delegation to junior staff justified.

For peer review only

Author	Country	Methodology	Analysis	Search Area	Evidence Contribution
deteriorating patients'. *Journal for specialists in pediatirc nursing*, 19, 339-349					
Lobos AT, Fernandes R, Ramsay T, McNally JD. (2014) Patient characteristics and disposition after pediatric medical emergency team (MET) activation: disposition depends on who activates the team. Hospital Pediatrics. 4(2):99-105	Canada	Implementation report	Simple descriptive statistics	**PEWS**	Delegation to junior staff<br><br>Education package developed around the RRS with a variety of informal lectures and interactive sessions; "lunch and learns", "education coffee carts" and simulation programs for instance.
Lydon et al. (2016) A mixed-methods investigation of health professionals' perceptions of a physiological track and trigger system. *BMJ Quality and Safety*, 25, 688-695		30 semi-structured interviews based on the theory of planned behaviour were conducted.<br><br>Questionnaire with Likert scale developed from the interview data	Deductive content analysis	**PEWS (second search)**	Professional intuition
Mackintosh, N., Humphrey, C., & Sandall, J. (2014a). The habitus of 'rescue' and its significance for implementation of rapid response systems in acute health care. Social Science & Medicine, 120, 233-242	U.K.	Ethnographic perspective; observations, semi-structured interviews	Data were inductively and deductively coded using NVivo v8 and organised thematically.	**EWS**	Delegation of routine observation and vital sign monitoring to junior staff<br><br>Professional intuition important; 'knowing the patient' important for detecting subtle changes in a patient's condition
Mackintosh, N., Rainey, H. and Sandall, J. (2012a)	U.K.	Comparative case study a rapid	Inductive and deductive coding	**EWS**	Observations and monitoring delegated to junior staff/health care assistants (HCAs) and nurses – early warning system (EWS) legitimised delegation of these tasks.

For peer review only - http://bmjopen.bmj.com/site/about/guidelines.xhtml

Author	Country	Methodology	Analysis	Search Area	Evidence Contribution
Understanding how rapid response systems may improve safety for the acutely ill patient: learning from the frontline. *BMJ Qual Saf*, 21, 135-144		response system (RRS) using ethnographic methods including observations, interviews and documentary review.	facilitated by NVivo. Also utilised theme building and structuring methods from framework analysis whilst also informed by other theoretical frameworks such as 'technology-in-practice'.		Importance of pattern recognition and intuition<br><br>The TTT used to confirm or identify deterioration and are particularly useful for junior staff.
Mackintosh, N., Watson, K., Rance, S., & Sandall, J. (2014b). Value of a modified early obstetric warning system (MEOWS) in managing maternal complications in the peripartum period: an ethnographic study. BMJ Quality & Safety, 23(1), 26-34	U.K.	Ethnographic study using observations (>120 h), semi-structured interviews (n=45) and documentary review	Thematic analysis with data analysed iteratively in addition to a more strategic and policy-focused coding framework	**EWS**	Professional intuition important when detecting deterioration
Massey, D., Chaboyer, W., & Aitken, L. (2014). Nurses' perceptions of accessing a Medical Emergency Team: a qualitative study. Australian Critical Care, 27(3), 133-138	Australia	In depth semi structured interviews	Inductive approach – thematic analysis	**EWS**	Importance of intuition or 'sensing clinical deterioration'
McDonnell, A., Tod, A., Bray, K., Bainbridge, D., Adsetts, D., & Walters, S. (2013). A before and after study assessing the impact of a new model for	U.K.	Single centre, mixed method before and after study including a survey to measure changes in nurses' knowledge after	Statistical analysis and thematic framework analysis	**EWS**	T&Ts used to confirm or identify deterioration depending on the experience of the user. Specific objective information was seen as helping the response arm prioritise work<br><br>Importance of professional intuition or 'gut feeling' in detecting deterioration

For peer review only - http://bmjopen.bmj.com/site/about/guidelines.xhtml

Author	Country	Methodology	Analysis	Search Area	Evidence Contribution
recognizing and responding to early signs of deterioration in an acute hospital. *Journal of Advanced Nursing,* 69(1), 41-52.		implementation of a track and trigger system (T&Ts). Also qualitative interviews			Need for staff concern in T&Ts
McKay, H., Mitchell, I., Sinn, K., Mugridge, H., Lafferty, T., Leuvan,, C. Mamootil, S. and Abdel-Latif, M. (2013) "Effect of a multifaceted intervention on documentation of vital signs and staff communication regarding deteriorating paediatric patients", *Journal of Paediatrics and Child Health,* Volume 49, Issue 1, pages 48–56	Australia	Prospective, controlled before and after intervention trial.	Statistical analysis	**PEWS**	Specific education package developed around the Paediatric Early Warning Score (PEWS) which assists in the recognition of deterioration
Mohammed, Hayton, R., Clements, G., Smith, G. and Prytherch, D. (2009) Improving accuracy and efficiency of early warning scores in acute care. *British Journal of Nursing*, 18, 1, 18-24	U.K.	Three phases; the first two were based in a classroom and asked nurses to calculate an early warning score (EWS) from vignettes using pen and paper followed by a hand-held computer. The third phase followed the previous approach but was based on the ward after	Statistical	**Electronic systems**	Timeliness of electronic vital sign recording when compared with paper systems

For peer review only - http://bmjopen.bmj.com/site/about/guidelines.xhtml

For peer review only

Author	Country	Methodology	Analysis	Search Area	Evidence Contribution
		nurses had been using the device for 4 weeks			
Mohammed Iddrisu, S. et al (2018) Nurses role in recognising and responding to clinical deterioration. *Journal of Clinical Nursing,* 27, 9-10, 1920-1930	Australia	To explore nurse' role in recognising and responding to deteriorating postoperative patients through focus groups.	Thematic analysis	**Snowball sample**	Staff concern in tool criteria useful
Paciotti B, Roberts KE, Tibbets KM, Weirich Paine C, Keren R, Barg FK, Holmes J H and Bonafide CP (2014). Physician attitudes toward family-activated medical emergency teams for hospitalized children. The Joint Commission Journal on Quality and Patient Safety, 40 (4)	U.S.A.	Semi-structured interviews with clinicians	Grounded theory and constant comparison	**Family involvement**	Physicians depend on families to explain a child's base-line
Pattison, N., & Eastham, E. (2012). Critical care outreach referrals: a mixed-method investigative study of outcomes and experiences. *Nursing in Critical Care,* 17(2), 71-82.	U.K.	Mixed method study looking at the impact of a critical care outreach team (CCOT)	Statistical analysis and grounded theory	**EWS**	Track and trigger tool is used to either confirm or identify deterioration depending on the experience of the user<br><br>'Gut feeling'/intuition important – patient appearance often an important sign in detecting deterioration
Radeschi, G., Urso, F., Campagna, S., Berchialla, P., Borga, S., Mina, A., . . . Sandroni, C. (2015). Factors affecting attitudes and barriers to a medical emergency team among	Italy	Multicentre survey to identify the attitudes and barriers to medical emergency team (MET) utilisation	Statistical analysis	**EWS**	Intuition (physical appearance important) in detecting deterioration

For peer review only - http://bmjopen.bmj.com/site/about/guidelines.xhtml

Author	Country	Methodology	Analysis	Search Area	Evidence Contribution
nurses and medical doctors: A multi-centre survey. *Resuscitation*, 88, 92-98.					
Schmidt, P. E., et al (2014). Impact of introducing an electronic physiological surveillance system on hospital mortality. BMJ Quality and safety.	U.K.	Retrospective analysis of data collected routinely. Pragmatic observational study of VitalPAC deployment in 2 large hospitals	Statistical analyses	**Electronic systems**	Electronic systems provide prompts for alerts facilitates better recognition of deterioration and is associated with reduced mortality.
Shearer, B., Marshall, S., Buist, M. D., Finnigan, M., Kitto, S., . . . Ramsay, W. (2012). What stops hospital clinical staff from following protocols? An analysis of the incidence and factors behind the failure of bedside clinical staff to activate the rapid response system in a multi-campus Australian metropolitan healthcare service. BMJ Quality & Safety, 21(7), 569-575	Australia	A mixed-method study	Iterative coding	**EWS**	Importance of professional intuition
Stevenson, J. E. & Nilsson G. (2011). Nurses' perceptions of an electronic patient record from a patient safety perspective: a qualitative study. Journal of	Sweden	Qualitative; focus groups with 21 registered nurses	Content analysis of interviews	**Electronic systems**	Verbal reports were favoured over the electronic system

For peer review only

For peer review only - http://bmjopen.bmj.com/site/about/guidelines.xhtml

Author	Country	Methodology	Analysis	Search Area	Evidence Contribution
Advanced Nursing 68(3), 667–676					
Stewart, J., Carman, M., Spegman, A., & Sabol, V. (2014). Evaluation of the effect of the modified early warning system on the nurse-led activation of the rapid response system. *Journal of Nursing Care Quality*, 29(3), 223-229.	Sweden	Mixed method. Retrospective review of records and nurse led focus groups	Statistical analysis and content analysis	**EWS**	Intuition still plays a part – the 'score' was rarely the single determining factor in activation but rather prompted nurses to gather additional information
Subbe et al. (2018) Effect of an automated notification system for deteriorating ward patients on clinical outcomes. *Critical Care*, 21, 52	U.K.	A before and after study of an electronic automated advisory V.S. monitoring and notification system. Elevated scores were relayed to a RRT.	Statistical analysis	**Snowball sample**	Automated VS monitoring associated with a decrease in mortality
Wager, K. A., Schaffner, M. J., Foulois, B., Swanson Kazley, A., Parker, C. Walo, H. (2010). Comparison of the Quality and Timeliness of Vital Signs Data Using Three Different Data-Entry Devices. Computers, Informatics, Nursing & Vol. 28, No. 4, 205–212	U.S.A	Observational study.	Descriptive statistics	**Electronic systems**	Batching of patient data whereby the care provider handwrites the patient's vital signs and uploads it to the computer at a later time is common, especially as the computers are often busy. Individual tablet PCs seems to eliminate this from occurring.
Watson, A., Skipper, C., Steury, R., Walsh, H. and Levin, A. (2014) Inpatient nursing care and early warning scores: a workflow	U.S.A.	Mixed method, retrospective medical record observations and observations of	Observation analysis, although this is not described, and statistical analysis	**PEWS**	Information relating to transfer to electronic systems – distance the 'eyes' of the nurses from the patient; batching.<br><br>Intuition

For peer review only - http://bmjopen.bmj.com/site/about/guidelines.xhtml

Author	Country	Methodology	Analysis	Search Area	Evidence Contribution
mismatch. *J Nurs Care Qual*, 29, 3, 215-222		nurse interactions in one minute blocks.			
Wheatley (2006). The nursing practice of taking level 1 patient observations. I&CCNurs 22: 115-121	U.K.	Ethnographic approach; participant observation & semi-structured interviews	Thematic & content analysis	**Observations and monitoring**	The regularity of vital signs monitoring may also lead to it becoming viewed as mundane practice that can be delegated to health care assistants.<br><br>Work place pressure/equipment failures affect quality of observations

For peer review only

For peer review only - http://bmjopen.bmj.com/site/about/guidelines.xhtml

TABLE 5 SUMMARY OF PREPARATION EVIDENCE

Author	Country	Methodology	Analysis	Search Area	Evidence Contribution
Abraham J, Kannampallil T, Patel B, Almoosa K, Patel V. (2012) Ensuring patient safety in care transitions: an empirical evaluation of a Handoff *Intervention Tool. AMIA Annual Symposium Proceedings/AMIA Symposium*, 17-26	U.S.A	Pre and post prospective study.	The quality and completeness of the handoff note – both tools – was assessed by a multi-professional round	**Structured handover**	Value of a checklist tool for handover
Brady P.W. and Goldenhar L.M. (2013a). A qualitative study examining the influences on situation awareness and the identification, mitigation and escalation of recognised patient risk. *BMJ Quality and Safetyl*, 0, 1-9.	U.S.A	Focus groups x7 – held in groups of participants with similar roles	Constant comparison	**Situational awareness**	Huddle useful to proactively identify and plan for risk<br><br>Structure to support handover important
Brady P.W., Muething S., Kotagal et al. (2013b). Improving situation awareness to reduce unrecognized clinical deterioration and serious safety events. *Pediatrics,* 131, 1.	U.S.A		Statistical process control charts	**Situational awareness**	Huddles led by a Watchstander charge nurse. When risk is identified the team discussed this and developed a plan to mitigate risk
Claussen, D., Garner, D. and Crow, B. (2013) Early warning signs and the EHR: At the intersection of technology and care. *Nursing Management*, 44, 11, 14-16	U.S.A	Retrospective review of calls to the rapid response team (RRT) and cardiac arrest calls to evaluate impact of evidence based guidelines	Descriptive statistics	**Electronic systems**	The huddle was seen as useful and was called as a patient's status was changing to 'red' so that all team members were informed

For peer review only - http://bmjopen.bmj.com/site/about/guidelines.xhtml

Davies, O., DeVita, M.A., Ayinla, A. and Perez, X.. (2014). Barriers to activation of the rapid response system. *Resuscitation*, 85(11), 1557-1561.	U.S.A	Survey looking at barriers to rapid response system (RRS) activation	Statistical analysis	**EWS**	Activation criteria displayed around the hospital
Demmel KM, Williams L, Flesch L. (2010) Implementation of the Pediatric Early Warning Scoring System on a Pediatric Hematology/Oncology Unit. *Journal of Pediatric Oncology Nursing*, 27(4):229-240	U.S.A	Discussion of the set-up and implementation of a paediatric early warning scoring tool and an associated algorithm	Rapid Plan-Do-Study-Act (PDSA) cycles were implemented using small tests of change. The data from the PDSA cycles were continuously collected, analysed, and reviewed with the multidisciplinary staff and planning team and used to give ongoing direction to the implementation plan.	**PEWS**	Importance of common information spaces & display of activation criteria throughout the hospital
Donahue M, Smith L, Dykes P. (2010) Phase 2 of the EMPOWER project: enhancing communication for paraprofessionals. *Journal of Continuing Education in Nursing*. 0;41(5).	U.S.A	Focus groups evaluation of a training programme which was developed to teach paraprofessionals situation, background, assessment, recommendation (SBAR) communication tool	Not clear	**Structured handover**	Value of a structured approach to handover<br><br>Adaptation of the SBAR tool for handover
Ennis, L (2014) Paediatric early warning scores on a children's ward: a quality improvement initiative. *Nursing children and young people*, 26, 7, 25-31	Ireland	Description of implementation of paediatric early warning score (PEWS) and subsequent audit (prospective cohort observational study)	Simple descriptive statistics of numbers children triggering PEWS and compliance with escalation protocol	**PEWS**	Common information spaces important & display of activation criteria throughout the hospital<br><br>Usefulness of ISBAR as a communication tool
Goldenhar LM, Brady PW, Sutcliffe KM, Muething SE (2013). Huddling for high reliability and situation	U.S.A	Semi structured interviews and focus groups to develop a deeper understanding of a newly implemented huddle systems	Constant comparison	**Situational awareness**	Importance of the huddle - Empowerment and sense of community; Facilitated greater and better information sharing

For peer review only - http://bmjopen.bmj.com/site/about/guidelines.xhtml

awareness. *BMJ Quality & Safety*, 22, 899-906					Each huddle participant was asked to systematically report on patients in their units who they thought would deteriorate in the near future and to label them as 'watchers', asking senior nurses and physician leads to coach charge nurses on how to integrate their perceptions into a informal severity of illness assessment (comprehension) and training the clinicians on how to use the information to facilitate prediction and planning for at risk patients (projection).
Mackintosh N, Berridge E-J, Freeth D. (2009) Supporting structures for team situation awareness and decision making: insights from four delivery suites. *Journal of Evaluation in Clinical Practice.*15(1):46-54.	U.K.	Ethnographic<br>2 stage process of lightly structured observations followed by a more focused period of data generation to test and elaborate the outcomes from stage 1	Initial thematic analysis and search for negative cases.	**Situational awareness**	For teams with a strong level of situation awareness (SA), key supports were all used in a balanced and supportive manner to gather and disseminate information which served to promote a culture of shared ownership and a proactive model of workload management, with temporary disruptions to the model easily accommodated.<br><br>Whiteboard – when used effectively was a vital means for supporting SA. It provides teams with a snapshot of a constantly

For peer review only - http://bmjopen.bmj.com/site/about/guidelines.xhtml

					changing workload, the team's activity levels and resource availability. Workload at times compromised its effectiveness. And irrespective of workloads, location and local traditions had a bearing on its use and usefulness.<br><br>White boards need to be in a visible location<br><br>Importantly, the interplay between these key support for SA will vary depending on the context; the same supports used differently will naturally produce different outcomes<br><br>Handover took many forms; structured and informal; profession specific and inter-professional; participatory or a one way transmission of information. The effectiveness of SA was dependent upon the form that handover took – who was present, contributions made, information relayed. SA was more likely to be compromised when key people were absent.<br><br>Senior staff coordinator important for SA and became compromised if they

For peer review only - http://bmjopen.bmj.com/site/about/guidelines.xhtml

					were largely focussed on providing patient care.
Mackintosh, N., Rainey, H. and Sandall, J. (2012a) Understanding how rapid response systems may improve safety for the acutely ill patient: learning from the frontline. *BMJ Qual Saf*, 21, 135-144	U.K.	Comparative case study a rapid response system (RRS) using ethnographic methods including observations, interviews and documentary review.	Inductive and deductive coding facilitated by NVivo. Also utilised theme building and structuring methods from framework analysis whilst also informed by other theoretical frameworks such as 'technology-in-practice'.	**EWS**	Availability of equipment an issue where the track and trigger tool (TTT) was electronic<br><br>Electronic systems helped HCAs and nursing staff to share understandings, planning and manage their workload
Massey, D., Chaboyer, W., & Aitken, L. (2014). Nurses' perceptions of accessing a Medical Emergency Team: a qualitative study. Australian Critical Care, 27(3), 133-138	Australia	In depth semi structured interviews	Inductive approach – thematic analysis	**EWS**	Common information spaces useful. Display of activation criteria throughout hospital
McCrory et al. (2012) ABC-SBAR Training improves simulated critical patient hand-off by pediatric interns. *Pediatric Emergency Care*. 0;28(6).	U.S.A	Prospective, pre-interventional and post-interventional study to evaluate the educational intervention of teaching ABC-SBAR	Two blinded reviewers assessed 52 video-recorded hand-offs for inclusion, order, and elapsed time to essential hand-off information using a scoring tool	**Structured handover**	Information sharing for handovers are of variable quality<br><br>A more structured approach will improve information sharing and therefore situational awareness – "without a structured hand-of tool, paediatric interns overemphasize background information and leave the reason for the call delayed".<br><br>Adaptation of the SBAR tool to include ABC – usefulness of this
Mullan PCM. A novel briefing checklist at shift handoff in	U.S.A	Descriptive observational study	Checklists were evaluated for rates of use, completion	**Situational awareness**	The value of more structured approach to information

For peer review only - http://bmjopen.bmj.com/site/about/guidelines.xhtml

an emergency department improves situational awareness and safety event identification. *Pediatric Emergency Care*. 2015;31(4):231-8.			and identification of potential safety events		sharing and situational awareness.<br><br>Utilises a checklist handover system for physicians. Checklist items focussed on the status of the patient, ED providers and hospital resources. A "Read-Do" format was designed.
Pearson G & Duncan H. (2011) Early warning systems for identifying sick children Paediatrics and Child Health. 21(5): 232-233	U.K.	Brief review of the evidence base surrounding PEWS	N/a	**PEWS**	Value of a more structured approach to communication - Advocate the use of a shared communication model such as SBAR (Situation, Background, Assessment and Recommendation) to communicate findings to superiors.
Pezzolesi C, Manser T, Schifano F, Kostrzewski A, Pickles J, Harriet N, et al. (2013) Human factors in clinical handover: Development and testing of a 'handover performance tool' for doctors' shift handovers. *International Journal for Quality in Health Care*. 25(1):58-65.	U.K.	Delphi Study for tool development	Descriptive statistics<br><br>Handovers were analysed and rated according to a measure of essential skills	**Situational awareness**	Information on handover – can be of variable quality<br><br>Most handover tools are adaptations of Situation Background Assessment Recommendation (SBAR) communication tool<br><br>Important to remember that handover facilitates teamwork and this hinges on communicative forms that extend beyond the information transfer that is

For peer review only - http://bmjopen.bmj.com/site/about/guidelines.xhtml

					typically the focus of structured handover tools
Randhawa S, Roberts-Turner R, Woronick K, DuVal J. (2011) Implementing and Sustaining Evidence-Based Nursing Practice to Reduce Pediatric Cardiopulmonary Arrest. Western Journal of Nursing Research. 33(3):443-456	U.S.A	Description of the implementation process with cardiopulmonary arrest (CPA) statistics pre and post implementation	Once a cycle from the implementation has been completed this is evaluated and then another cycle begins.	**PEWS**	White boards placed in a central location displayed scores of all patients so that staff could quickly assess which patients were at high risk for deterioration.<br><br>Activation criteria displayed throughout the hospital
Stewart, J., Carman, M., Spegman, A., & Sabol, V. (2014). Evaluation of the effect of the modified early warning system on the nurse-led activation of the rapid response system. *Journal of Nursing Care Quality*, 29(3), 223-229.	Sweden	Mixed method. Retrospective review of records and nurse led focus groups	Statistical analysis and content analysis	**EWS**	Huddle –the score was used during the daily bed 'huddle' with the nursing administrators, to evaluate current unit acuity, determine staffing need & prepare for any transfers
Van Voorhis, K.T. and Scahde Willis, T. (2009) Implementing a pediatric rapid response system to improve quality and patient safety. *Pediatr Clin N Am*, 56, 919-933	U.S.A.	Discussion paper highlighting the process of developing a paediatric rapid response system (RRS).	N/a	**PEWS**	Display of activation criteria throughout hospital on lanyards and use of white boards useful
Wager, K. A., Schaffner, M. J., Foulois, B., Swanson Kazley, A., Parker, C. Walo, H. (2010). Comparison of the Quality and Timeliness of Vital Signs Data Using Three Different Data-Entry Devices. Computers, Informatics, Nursing & Vol. 28, No. 4, 205–212	U.S.A	Observational study.<br><br>Explored the timeliness and quality of vital signs data entered by 3 different recording methods	Descriptive statistics	**Electronic systems**	Batching of patient data whereby the care provider handwrites the patient's vital signs and uploads it to the computer at a later time is common, especially as the computers are often busy.
Watson, A., Skipper, C., Steury, R., Walsh, H. and Levin, A. (2014) Inpatient	U.S.A.	Mixed method, retrospective medical record observations and	Observation analysis, although this is not	**PEWS**	Availability of equipment a factor

For peer review only - http://bmjopen.bmj.com/site/about/guidelines.xhtml

nursing care and early warning scores: a workflow mismatch. *J Nurs Care Qual*, 29, 3, 215-222		observations of nurse interactions	described, and statistical analysis		
Weiss M.J.B., Bhanji, F., Fontela, PS. And Razack, SI.. (2013) A preliminary study of the impact of a handover cognitive aid on clinical reasoning and information transfer. Medical Education. 0;47(8)	Canada	A randomised controlled trial in an academic paediatric intensive care unit (PICU) of 20 handover events	Differences between intervention and control groups were assessed using the Mann-Whitney test and multivariate linear regression.	**Structured handover**	Value of a more structured approach to support information sharing - a cognitive aid to facilitate handover that prompted residents to transmit this information. The handover aid was not linked into hospital information systems – so this had to be completed by hand before handover.<br><br>Handover is an opportunity for learning and professional socialisation
Wong, D., Bonnici, T., Knight, J., Morgan, L., Coombes, P. and Watkinson, P. (2015) SEND: a system for electronic notification and documentation of vital sign observations. *BMC Medical Informatics and Decision Making*, 15, 68.	U.K.	Description of user-focussed design process for use of electronic monitoring & numbers of observations taken using the system. Acceptability questionnaire	Descriptive statistics on the number of observations recorded using the SEND system & the number of active users	**Electronic systems**	Development of a flexible electronic system which enabled staff to have an overview of patients – reflections on disconnection

For peer review only - http://bmjopen.bmj.com/site/about/guidelines.xhtml

TABLE 6 SUMMARY OF ACTION EVIDENCE

Author	Country	Methodology	Analysis	Search Area	Evidence Contribution
Adelstein, B. A., Piza, M. A., Nayyar, V., Mudaliar, Y., Klineberg, P. L., & Rubin, G. (2011). Rapid response systems: A prospective study of response times. Journal of Critical Care, 26, 635	Australia	Prospective comparison of rapid response team (RRT) criteria breaches	Statistical	**EWS**	Day/night differences in activation identified<br><br>Nurses may not understand what is required for activation.<br><br>Staff not investing in calling criteria
Almblad, A.C., Siltberg, P., Engvall, G. and Malqvist, M. (2018) Implementation of Pediatric Early Warning Score; Adherence toGuidelines and Influence of Context. *Journal of Pediatric Nursing,* 38, 33-39	Sweden	retrospective review of EPR and a context assessment of the work environment using the Alberta Context Tool.	Statistical	**Snowball sample**	Senior leadership consistently identified as important
Andrews, T. and Waterman, H. (2005) Packaging: a grounded theory of how to report physiological deterioration effectively. *Journal of Advanced Nursing* 52, 5,473–481	U.K	Interviews and observations	Grounded theory	**EWS**	Track & Trigger Tools (TTT) act as prompts to action<br><br>TTT used to overcome challenges in communication & particularly valuable for junior staff<br><br>Negative attitude towards calling for help
Astroth, K., Woith, W. M., Stapleton, S. J., Degitz, R. J., & Jenkins, S. H. (2013). Qualitative exploration of nurses' decisions to activate rapid response teams. Journal of Clinical Nursing, 22(19-20), 2876-2882	U.S.A	Semi-structured interviews with nurses	Coding categories were generated from the data, and consensus on final themes was achieved through an iterative process	**EWS**	Situation under control – no need to escalate or perceived business of medical staff discouraged staff from RRT activation<br><br>Staff encouraged to use their intuition when activating the RRT<br><br>Concern about feeling inadequate in front of colleagues a barrier to RRT activation<br>Inexperienced staff teaming up – led to staff trusting their own judgement<br><br>Traditional hierarchies a barrier to RRT activation – nurses more likely to call the attending physician rather than activate the RRT

For peer review only - http://bmjopen.bmj.com/site/about/guidelines.xhtml

Author	Country	Methodology	Analysis	Search Area	Evidence Contribution
Azzopardi, P.; Kinney, S.; Moulden, A. & Tibbals, J. Attitudes and barriers to a Medical Emergency Team system at a tertiary paediatric hospital. *Resuscitation*, 82, 167-174	Australia	Survey	Statistical analysis	**PEWS**	Score rarely the determining factor in escalation - would not escalate for a patient who looked well but would escalate for a patient they were worried about even if not triggering<br><br>Negative attitude towards calling for help – feeling inadequate/perceived business of Paediatric Intensive Care Unit had an impact on doctors escalating but not nurses<br><br>Senior leadership is important when implementing a MET
Bogert, S., Ferrell, C. and Rutledge, D. N. (2010). Experience with Family Activation Of Rapid Response Teams. MEDSURG Nursing—July/August 2010—Vol. 19/No. 4	U.S.A	Implementation of Condition Help (ConditionH)	Descriptive statistics	**Family involvement**	Implementation of ConditionH.<br><br>ConditionH being addressed during daily rounds
Bonafide C., Roberts K., Weirich C., Paciotti B., Tibbetts K., Keren R., Barg F. and Holmes J. (2013) Beyond Statistical Prediction: Qualitative Evaluation of the Mechanisms by which Pediatric Early Warning Scores Impact Patient safety. *J Hospital Medicine*, 8, 5, 248-253	U.S.A	Semi-structured interviews	Grounded theory	**PEWS**	Disinclination to seek help and concerns about appearing inadequate in front of colleagues<br><br>Informal peer support<br><br>Senior leadership important
Braaten, J.S. (2015). Hospital System Barriers to Rapid Response Team Activation: A Cognitive Work Analysis. *American Journal of Nursing*, 115(2), 22-32.	U.S.A	Document review and interviews using the principles of cognitive work analysis	Inductive and deductive forms of analysis – cognitive work analysis, framework and directed content analysis	**EWS**	Issues around availability of equipment and staffing<br><br>Negative attitude/delays around calling for help with staff needing to justify escalation. Other factors impact upon this including the perception that the situation is under control/perceived business of physicians/not wanting to appear inadequate
Brady P.W., Muething S., Kotagal et al. (2013b). Improving situation awareness to reduce	U.S.A		Statistical process control charts	**Situational awareness**	Concerns about resources reported

For peer review only - http://bmjopen.bmj.com/site/about/guidelines.xhtml

Author	Country	Methodology	Analysis	Search Area	Evidence Contribution
unrecognized clinical deterioration and serious safety events. *Pediatrics*, 131, 1.					
Brady, P.W. et al. (2014) Developing and evaluating the success of a family activated medical emergency team: a quality improvement report. *BMJ Qual Saf* 2014; 0: 1–9.	U.S.A.	A retrospective cohort study looking at the association between family and clinician activations and transfer to the intensive care unit following a MET call	Quality improvement methods and statistical process control charts were used to assess the rate of family activation of METs	**Family involvement**	Direct mechanism for families to activate a MET<br><br>Concerns from clinicians about a family activated MET over-burdening the system are unfounded
Chua, W. L., Mackey, S., Ng, E. K. C., & Liaw, S. Y. (2013). Front line nurses' experiences with deteriorating ward patients: a qualitative study. *International Nursing Review*, 60, 4., 501-509.	Singapore	A qualitative survey using critical incident technique	Inductive content analysis	**EWS**	Staff felt that they had not been educated to an adequate level – training lacking<br><br>Negative attitude towards calling for help – fears of appearing inadequate
Cioffi, J. (2000). Nurses' experiences of making decisions to call emergency assistance to their patients. Journal of Advanced Nursing, 32(1), 108-114.	Australia	Unstructured interviews with nurses who had activated the medical emergency team (MET)	Simple code and retrieve	**EWS**	Reluctance to activate – doubting ability; fears of appearing inadequate; decisions made based on the perceived availability of resources/business of medical staff/time of day all had an impact on decisions to activate the MET<br><br>Importance of having staff concern in the MET criterion
Cioffi, J. (2000). Recognition of patients who require emergency assistance: a descriptive study. Heart & Lung, 29(4), 262-268.	Australia	Unstructured interviews	Simple code and retrieve	**EWS**	Importance of having staff concern in a calling criteria<br><br>Reluctance to activate – business of ward a factor

For peer review only - http://bmjopen.bmj.com/site/about/guidelines.xhtml

Author	Country	Methodology	Analysis	Search Area	Evidence Contribution
Cioffi, J., Salter, C., Wilkes, L., Vonu-Boriceanu, O., & Scott, J. (2006). Clinicians' responses to abnormal vital signs in an emergency department. Australian Critical Care, 19(2), 66-72.	Australia	Focus groups with clinicians and nurses exploring their responses to abnormal vital signs	Constant comparison	**EWS**	Availability of equipment an issue/staffing pressures; staff unable to carry out routine monitoring that would enable the detection of abnormal vital signs (AVS)/escalation hampered because of difficulty finding the appropriate senior person<br><br>.<br><br>Medical emergency team (MET) criteria used to confirm or identify deterioration depending on experience<br><br>Negative attitude towards asking for help - lack of confidence questioning peers/fear of being reprimanded /feeling the situation was under control
Dean, BS, Decker MJ, Hupp D, Urbach AH, Lewis E, Benes-Stickle J (2008) Condition HELP: a pediatric rapid response team triggered by patients and parents. *J Health Qual*, 30, 3, 28-31	U.S.A	Two year reflection following implementation of Condition Help (ConditionH)	Descriptive statistics	**Family involvement**	ConditionH criteria for activation<br><br>Concern that family activated RRS could divert attention away from resources<br><br>Clinician involvement important<br><br>Daily 'patient rounds' involving patients and families is useful<br><br>Patients and families have access to relevant information and understand the medical information and care plans.
Demmel KM, Williams L, Flesch L. (2010) Implementation of the Pediatric Early Warning Scoring System on a Pediatric Hematology/Oncology Unit. *Journal of Pediatric Oncology Nursing*, 27(4):229-240	U.S.A	Discussion of the set-up and implementation of a paediatric early warning scoring tool and an associated algorithm	Rapid Plan-Do-Study-Act (PDSA) cycles were implemented using small tests of change.	**PEWS**	Education package developed around the history and development of PEWS along with the rationale for and the goals of the initiative. The scoring process was explained and how it would be integrated into routine nursing assessments; normal vital sign parameters were reviewed.<br><br>Importance of common information spaces & display of activation criteria throughout the hospital<br><br>Senior lead commitment & importance of champions integral for implementation
Donohue, L. and Endacott, R. (2010) Track, trigger and teamwork: Communication of deterioration in acute medical and surgical wards. *Intensive and Critical Care Nursing*, 26, 10-17	U.K	Qualitative design with critical incident technique. Semi-structured interviews with nurses and the outreach team	Thematic analysis	**EWS**	Some resistance to escalation – clinicians preferring to deal with patient problems within their own team<br><br>Inexperienced staff teaming up with more experienced staff once patient deterioration was recognised

For peer review only - http://bmjopen.bmj.com/site/about/guidelines.xhtml

Author	Country	Methodology	Analysis	Search Area	Evidence Contribution
Endacott, R., & Westley, M. (2006). Managing patients at risk of deterioration in rural hospitals: a qualitative study. Australian Journal of Rural Health, 14(6), 275-279	Australia	Questionnaire, in-depth interviews and observations	Content analysis and constant comparison	**EWS**	Art of referral important – using the right language and suggesting actions that would be acceptable to the doctor<br><br>Availability of equipment a factor<br><br>Negative attitude towards calling for help; Escalation dependent upon perceived capability of medical staff
Ennis, L (2014) Paediatric early warning scores on a children's ward: a quality improvement initiative. *Nursing children and young people*, 26, 7, 25-31	Ireland	Description of implementation of paediatric early warning score (PEWS) and subsequent audit (prospective cohort observational study)	Simple descriptive statistics of numbers children triggering PEWS and compliance with escalation protocol	**PEWS**	Structured education and training programme on the use of ISBAR (Identify, Situation, Background,<br><br>Assessment and Recommendation) and PEWS was provided and nurse manager/staff nurse in charge should review any educational requirements in completing PEWS, particularly for relief staff<br><br>Common information spaces important & display of activation criteria throughout the hospital<br><br>Usefulness of ISBAR as a communication tool<br><br>Senior lead commitment – PEWS management policy developed/senior staff promote and reinforce use of PEWS
Entwistle, V. (2004). Nursing shortages and patient safety problems in hospital care: is clinical monitoring by families part of the solution? Health Expectations, 7, pp.1–5 (editorial)	U.S.A	Editorial	N/a	**Family involvement**	Little evidence/no evaluations of policies or practices that encourage and support family involvement in clinical monitoring<br><br>Propose the innovative practice of interdisciplinary rounds where families are invited and communication is directed to the patient and family
Gerdick, C., Vallish, R., Miles, K., Godwin, S., Wludyka, P. and Panni, M. (2010) Successful implementation of a family and patient activated rapid response team in an adult level 1 trauma center. *Resuscitation*, 81, 1676-1681	U.S.A	Routine data collection for number of rapid response team (RRT) calls and the result of these activations and patient/family survey relating to RRT activation	Statistical analysis	**Family involvement**	Direct mechanism for families to activate the RRT<br><br>Barriers to family activation highlighted, specifically professional resistance<br><br>Physician and leadership support important to overcome barriers

For peer review only

Author	Country	Methodology	Analysis	Search Area	Evidence Contribution
Greenhouse, P., Kuzminsky, B., Martin, S., and Merryman, T. (2006) Calling a Condition H(elp). *AJN*, 106, 11	U.S.A focus	Discussion about the implementation of Condition Help (ConditionH)	Descriptive statistics	**Family involvement**	Appropriateness of calls is reported rather that why they are made<br><br>Note some scepticism and wariness among nurses and physicians
Hueckel, R. M., Mericle, J. M., Frush, K., Martin, P. L. and Champagne M. T. (2012). Implementation of Condition Help. Family Teaching and Evaluation of Family Understanding. J Nurs Care Qual Vol. 27, No. 2, pp. 176–181	U.S.A.	Scripted family teaching about RRT activation at the time of patient admission from Condition Help (ConditionH).	Descriptive statistics about delivery of educational programme and rapid response team (RRT) call-out; survey testing family understanding	**Family involvement**	Description of ConditionHelp<br>Appropriateness of calls is reported rather that why they are made
James, J., Butler-Williams, C., Hunt, J. and Cox, H. (2010) Vital signs for vital people: an exploratory study into the role of the Healthcare Assistant in recognising, recording and responding to the acutely ill patient in the general ward setting. *Journal of Nursing Management*, 18, 548-555	U.K.	Postal survey with Healthcare Assistants (HCAs) utilising closed and open questions	Descriptive statistics & content analysis of qualitative data	**Observations and monitoring**	Workload and ward distractions a barrier to activation, such as time spent locating equipment.<br><br>Disinclination to seek help from senior staff/clinicians
Kaul, M., Snethen, J., Kelber, S., Zimmanck, K., Maletta, K. and Meyer, M. (2014) Implementation of the bedside paediatric early warning system (BedsidePEWS) for nurse identification of deteriorating patients'. *Journal for specialists in pediatirc nursing*, 19, 339-349	U.S.A	Descriptive cross sectional study; nurse and medical staff survey	Descriptive statistics	**PEWS**	Noted that the score provides a 'universal language' and interdisciplinary communication

For peer review only - http://bmjopen.bmj.com/site/about/guidelines.xhtml

Author	Country	Methodology	Analysis	Search Area	Evidence Contribution
Lobos AT, Fernandes R, Ramsay T, McNally JD. (2014) Patient characteristics and disposition after pediatric medical emergency team (MET) activation: disposition depends on who activates the team. Hospital Pediatrics. 4(2):99-105	Canada	Implementation discussion	Simple descriptive statistics	**PEWS**	Situation Background Assessment Recommendation (SBAR) helps to establish a common language and guide escalated events<br><br>Negative attitude towards calling for help – traditional hierarchies a barrier to activation/concerns about communication between primary and responding team<br><br>No false alarms and de-brief useful<br><br>Importance of champions (utilising a social marketing approach) to encourage 'inter-professional collaboration & advisory group to help establish a sense of ownership<br><br>Lack of support from superiors means less likely to escalate
Mackintosh, N., Rainey, H. and Sandall, J. (2012a) Understanding how rapid response systems may improve safety for the acutely ill patient: learning from the frontline. *BMJ Qual Saf*, 21, 135-144	U.K.	Comparative case study a rapid response system (RRS) using ethnographic methods including observations, interviews and documentary review.	Inductive and deductive coding facilitated by NVivo. Also utilised theme building and structuring methods from framework analysis whilst also informed by other theoretical frameworks such as 'technology-in-practice'.	**EWS**	Availability of equipment an issue where the track and trigger tool (TTT) was electronic<br><br>Gave junior staff license to escalate care.<br>Additionally, 'while standardisation of practice clearly has its benefits, it also comes at a cost that these tools attenuate lower level staff's authority and ability to persuade staff higher up in the organisation of the credibility of their knowledge' p 143.<br><br>Efforts to develop junior staff's communication and clinical understanding need to acknowledge power dynamics at play.<br><br>Usefulness of situation-background-assessment-recommendation' (SBAR) communication tool as part of the escalation policy as reported by staff (not seen in action).<br><br>Negative attitude towards escalation – difficulty in summoning a response<br><br>Senior lead commitment to patient safety was important. Zero tolerance for cardiac arrest was championed by senior staff<br><br>Night time/out of hours pressures identified
Mackintosh, N., Humphrey, C., & Sandall, J. (2014a). The habitus of 'rescue' and its significance for implementation of rapid response systems in acute	U.K.	Ethnographic perspective; observations, semi-structured interviews	Data were inductively and deductively coded and organised thematically.	**EWS**	Negative attitude towards seeking help. Escalating care outside the parameters marked by a track and trigger tool proved difficult; Power struggles identified – junior staff have difficulty persuading more senior staff of the credibility of their knowledge<br><br>Difficulties in activation across professional boundaries

For peer review only - http://bmjopen.bmj.com/site/about/guidelines.xhtml

Author	Country	Methodology	Analysis	Search Area	Evidence Contribution
health care. Social Science & Medicine, 120, 233-242					
Massey, D., Chaboyer, W., & Aitken, L. (2014). Nurses' perceptions of accessing a Medical Emergency Team: a qualitative study. Australian Critical Care, 27(3), 133-138	Australia	In depth semi structured interviews	Inductive approach – thematic analysis	**EWS**	Common information spaces useful. Display of activation criteria throughout hospital<br><br>General negative attitude towards calling for help – appearing inadequate in front of others<br><br>Importance of leadership support<br><br>Peers support – would often consult their colleagues
McCabe, A. and Duncan, H. (2009) "Paediatric early warning systems: where do we go from here?" *Paediatric Nursing*, 21, 1, 14-17	U.K.	Opinion piece about lessons to be learnt from the adult experience of implementing early warning systems	N/a	**PEWS**	Specific education package needed on how to use an early warning system (EWS) and on basic clinical assessment, guidance and standardisation of observation and monitoring. Advocate situational simulated scenario education and e-learning<br><br>Highlight the usefulness of communication tools such as situation, background, assessment, recommendation (SBAR) for establishing roles and responsibilities, engaging them in making an appropriate management plan that can, if necessary, be escalated<br><br>Senior lead commitment key - reflected in resources and education - to improve the safety and quality of care of hospitalised patients<br><br>Families need to be empowered to request a patient review
McDonnell, A., Tod, A., Bray, K., Bainbridge, D., Adsetts, D., & Walters, S. (2013). A before and after study assessing the impact of a new model for recognizing and responding to early signs of deterioration in an acute hospital. *Journal of Advanced Nursing,* 69(1), 41-52.	U.K.	Single centre, mixed method before and after study including a survey to measure changes in nurses' knowledge after implementation of a track and trigger system (T&Ts). Also qualitative interviews	Statistical analysis and thematic framework analysis	**EWS**	Rolling education programme for all nurses on the recognition and response to deteriorating patients & an overview of the T&Ts<br>Work place pressures; nurses concerned that they couldn't always summon a timely response from doctors /night time pressures also identified<br><br>Need for staff concern in T&Ts
Monaghan A. (2005) Detecting and managing deterioration in children.	U.K. focus	Commentary on the development of the Brighton paediatric early	Simple descriptive statistics of all activations, actions and outcomes	**PEWS**	Education based model was developed to assist in recognising deterioration<br><br>Temporary staff/workplace pressures impact upon staffs' ability to detect deterioration

For peer review only - http://bmjopen.bmj.com/site/about/guidelines.xhtml

Author	Country	Methodology	Analysis	Search Area	Evidence Contribution
Paediatric Nursing. 17(1):32-35		warning score and setting up a paediatric critical care outreach team.	during the first three months of implementation		
Paciotti B, Roberts KE, Tibbets KM, Weirich Paine C, Keren R, Barg FK, Holmes J H and Bonafide CP (2014). Physician attitudes toward family-activated medical emergency teams for hospitalized children. The Joint Commission Journal on Quality and Patient Safety, 40 (4)	U.S.A.	Semi-structured interviews with clinicians to explore physicians viewpoints on families facilitating the identification of children with a deteriorating condition.	Grounded theory and constant comparison	**Family involvement**	Concerns that resources would be diverted away with an increase in calls – not supported
Pattison, N., & Eastham, E. (2012). Critical care outreach referrals: a mixed-method investigative study of outcomes and experiences. *Nursing in Critical Care,* 17(2), 71-82.	U.K.	Mixed method study looking at the impact of a critical care outreach team (CCOT)	Statistical analysis and grounded theory	**EWS**	Availability of equipment an issue/workload<br><br>Negative attitude towards calling for help – situation under control/ward business<br><br>Inexperienced staff teaming up/checking with peers before calling the CCOT
Pearson G & Duncan H. (2011) Early warning systems for identifying sick children Paediatrics and Child Health. 21(5): 232-233	U.K.	Brief review of the evidence base surrounding PEWS together with reflections from their own experiences from the Birmingham Children's Hospital	N/a	**PEWS**	Team training & education is important increasing confidence in the use of medical language and empowering bedside carers. 'Although doing observations is fundamental to nursing practice many … have not been taught a structured approach to assessment'. Advocate a simulated environment<br><br>Value of a more structured approach to communication - Advocate the use of a shared communication model such as SBAR (Situation, Background, Assessment and Recommendation) to communicate findings to superiors.<br><br>Need for senior commitment – cultural change may be required to ensure management support (reflected in resources and education) /Importance of champions

For peer review only - http://bmjopen.bmj.com/site/about/guidelines.xhtml

Author	Country	Methodology	Analysis	Search Area	Evidence Contribution
Salamonson, Y., van Heere, B., Everett, B., & Davidson, P. (2006). Voices from the floor: Nurses' perceptions of the medical emergency team. *Intensive & Critical Care Nursing*, 22(3), 138-143.	Australia	Survey with closed and open questions to examine perceptions of and satisfaction with the medical emergency team (MET)	Descriptive statistics and content analysis	**EWS**	Need for more education on deterioration identified<br><br>Negative attitude towards asking for help; attitude of MET team a barrier to activation
Shearer, B., Marshall, S., Buist, M. D., Finnigan, M., Kitto, S., . . . Ramsay, W. (2012). What stops hospital clinical staff from following protocols? An analysis of the incidence and factors behind the failure of bedside clinical staff to activate the rapid response system in a multi-campus Australian metropolitan healthcare service. BMJ Quality & Safety, 21(7), 569-575	Australia	A multi-method study;<br>A point prevalence survey;<br>A prospective audit of all patients experiencing a cardiac arrest, unplanned intensive care (ICU) unit admission or death over an 8 week period.<br>Structured interviews with staff to explore cognitive and sociocultural barriers to activation	Iterative coding	**EWS**	Adequate staffing and a lack of beds on critical care leads to a failure to activate the RRS<br><br>Score rarely the single determining factor in activation despite the fact that staff recognised patients met activation criteria. Data from the point prevalence study confirm this as only one patient had a serious adverse event<br><br>Negative attitude towards calling for help – situation under control; treating team had expertise to treat (particularly when the physiological instability was in the area of expertise of the treating team)<br><br>Traditional (intra-professional clinical) hierarchies a barrier to activation
Stewart, J., Carman, M., Spegman, A., & Sabol, V. (2014). Evaluation of the effect of the modified early warning system on the nurse-led activation of the rapid response system. *Journal of Nursing Care Quality*, 29(3), 223-229.	Sweden	Mixed method. Retrospective review of records and nurse led focus groups	Statistical analysis and content analysis	**EWS**	The RRS was valuable for junior staff escalating care across hierarchical and professional boundaries<br><br>Senior lead commitment – culture of support promoted by nursing administrators

For peer review only - http://bmjopen.bmj.com/site/about/guidelines.xhtml

Author	Country	Methodology	Analysis	Search Area	Evidence Contribution
Van Voorhis, K.T. and Scahde Willis, T. (2009) Implementing a pediatric rapid response system to improve quality and patient safety. *Pediatr Clin N Am*, 56, 919-933	U.S.A.	Discussion paper highlighting the process of developing a paediatric rapid response system (RRS).<br><br>The system was evaluated by prospectively collected data recorded on RRS activation forms and existing performance improvement database information.	N/a	**PEWS**	Display of activation criteria throughout hospital on lanyards and use of white boards useful<br><br>De-briefing following activation and a commitment to no false alarms is encouraged<br><br>Senior lead commitment – administrative arm of the RRS vital<br><br>Utilises ConditionHelp. The appropriateness of calls was facilitated by the 'no false alarms' culture.
Watson, A., Skipper, C., Steury, R., Walsh, H. and Levin, A. (2014) Inpatient nursing care and early warning scores: a workflow mismatch. *J Nurs Care Qual*, 29, 3, 215-222	U.S.A.	Mixed method, retrospective medical record observations and observations of nurse interactions in one minute blocks.	Observation analysis, although this is not described, and statistical analysis	**PEWS**	Availability of equipment a factor<br><br>Score rarely the determining factor in escalation<br><br>SBAR

For peer review only - http://bmjopen.bmj.com/site/about/guidelines.xhtml

For peer review only

For peer review only - http://bmjopen.bmj.com/site/about/guidelines.xhtml

Appendix 1: Data Extraction Template 1

PUMA literature review template

Study/Paper	
Authors, title, date, source	
Purpose	
Contribution to review	
Design/Methods	
Participants/Setting	
Data type	
Theoretical framework	
Quality Appraisal Tool	
Quality Assessment	
Intervention type	
Formal study finding	
Recommendations	

For peer review only

Context	
History of intervention	
Country	
Organisation type (i.e. DGH, tertiary hospital, primary care)	
PEWS (efferent) wider activity system elements and inter-relationships with TTT [decision algorithms, CCOT, MET, ICU liaison, PICU, HDU]	
Infra-structural context [other related artefacts]	
Related activity systems [QI, audit, governance, referral processes and inter-relationships with TTT]	
Socio-cultural context [including experience of QI, stability of team and division of labour, teaching-non-teaching etc]	
Wider policy/organisational context	
**Intervention (TIDieR)**	
Name	
Rationale, theory or goal of the elements – active ingredients - identified as essential to the intervention [DA: this could be explicit or implicit in the paper]	
What (materials): Describe any physical or informational	

For peer review only

materials used in the intervention, including those provided to participants or used in intervention delivery or in training of intervention providers.

**TTT Affordances (including but not limited to indicative list)**	
Paper/electronic	
What observations/information are recorded?	
How is data displayed?	
Single parameter/ Aggregated weighting	
Response algorithm(s) (including details)	
Parental/patient involvement	
Manual or automated monitoring	
Proactive intermittent/continuous monitoring	
Linked artefacts (e.g SBAR)	
Audit/performance surveillance	

For peer review only

What (procedures): describe each of the procedures, activities, and/or processes used in the intervention, including any enabling or supportive activities.	
Who provided: For each category of intervention provider (for example, nursing assistant, nurse, junior doctor) describe their expertise, background and any specific training given and also the roles and relationships within the overall division of labour.	
How: Describe the mechanisms (implicit or explicit) about how the intervention, or intervention components have their effects	
How: Describe the modes of TTT delivery (such as face to face or some other mechanism such as phone or internet), or whether the intervention was provided individually or in a group.	
When and how much: Describe the number of times the intervention was delivered and over what period of time,	

For peer review only

including the number of sessions, schedule and intensity of dose (universal or selective application)				
Tailoring: If the intervention was planned to be personalised or adapted, then describe what, why, when and how.				
Modifications: If the intervention was modified during the course of the study, describe the changes (What, why, when, and how)				
Assessment of intervention adherence or fidelity, describe how and by whom, and if any strategies were used to maintain or improve fidelity, describe them				
If intervention adherence or fidelity was assessed, describe the extent to which the intervention was delivered as planned				
**Implementation (NPT) (questions modified from Murray et al. 2010 and kennedy et al 2013)**				
Coherence [i.e. the meaning attributed to PEWS and whether it makes sense for users e.g. cultural and scientific legitimacy, feasibility]	Policy	Organisational	Professional	Patient/family

For peer review only

For peer review only - http://bmjopen.bmj.com/site/about/guidelines.xhtml

• Evidence that the intervention was easy to describe<br>• Evidence that it was recognised as different from existing ways of working by stakeholders<br>• Evidence that it has a clear purpose for all relevant actors<br>• Evidence that stakeholders recognise the benefits the intervention will bring and to whom<br>• Evidence that the intervention fitted with the overall goals and activity of the organisation				
Cognitive Participation [i.e. commitment and engagement with the intervention]<br>• Evidence that the target group see the intervention was a good idea<br>• Evidence that they were prepared to invest time and energy and work in it?				

For peer review only

• Evidence that the stakeholders thought their engagement with the intervention was appropriate<br>• Evidence that the stakeholders have taken steps to sustain the use of the intervention				
Collective Action [what are the skills and resources required to enact the intervention?]<br>• How did the intervention affect the user groups?<br>• What work was undertaken to operationalise the intervention?<br>• Did it promote or impede their work?<br>• Did staff require extensive training before they can use it?<br>• How compatible was the intervention with existing work practices?<br>• What impact did it have on the division of				

For peer review only

labour, resources, power and responsibility between different professional groups?<br>• Did it fit with the overall goals and activity of the organisation?				
Reflexive Monitoring [i.e methods for formal and informal evaluation of the effects of the intervention]<br>• Did stakeholders take practical steps to measure the influence of the intervention?<br>• How did users perceive the intervention once it has been in use for a while?<br>• Was the intervention seen as advantageous for patients and staff?<br>• Was it clear what effects the intervention has had (including unintended consequences)?<br>• Did users' contribute feedback on the				

For peer review only

intervention once it is in use?<br>• Was the intervention be adapted/improved on the basis of this experience?				

**Findings (positive, negative and unintended consequences) and useful content**
**Emergent theories about key ingredients and mechanisms (including summary of supporting evidence)**
**Emergent theories of the factors necessary for paediatric EWS to be normalised (including summary of supporting evidence)**
**Additional Insights of note (including references to follow up)**

For peer review only

Appendix 2: Search Methodology

Search Methods

Database Search

A comprehensive search was conducted across a wide-ranging set *of databases* from 1995 to April 2015 to identify relevant evidence/studies in English language.

A preliminary search strategy was developed using a set of key papers known to the group for Ovid Medline using both text words and Medical subject headings. A total of four systematic searches was conducted across a range of databases from 1995 to April 2015 to identify relevant studies in the English language papers reporting on:

- Paediatric Early Warning Systems (all study types).
- Adult Early Warning Systems (qualitative studies only)
- Interventions to improve situational awareness (all study types)
- Structured communication tools for handover and handoff (all study types)

The Medline search strategy was translated to use across the rest of the databases.

The focus of the search strategy was to achieve high sensitivity and specificity for retrieving studies relevant to the review question. The Medline search strategy was modified according to the indexing systems of the other databases.

Table 1.

**Databases**
British Nursing Index
CINAHL (Cumulative Index of Nursing and Allied Health Literature)
Cochrane Central Register of Controlled Trials
Database of Abstracts of Reviews of Effectiveness
EMBASE
HMIC (Health Management Information Centre)
Medline
Medline in Process
Scopus
Web of Knowledge (Science Citation Indexes)

Additional searches

In addition to the above databases, we searched both published and unpublished literature. To identify supplementary papers, information on studies in progress, unpublished research or research reported in the grey literature was identified through searching a range of relevant websites and trial registers including Clinical Trials.gov. To identify published resources that had not yet been catalogued in the electronic databases, recent editions of key journals were hand-searched.

**Trial Registers**
ClinicalTrials.gov<br>https://clinicaltrials.gov/
UK Clinical Trials Gateway

For peer review only

http://www.ukctg.nihr.ac.uk/default.aspx
The WHO trial search portal for studies worldwide:<br>http://apps.who.int/trialsearch
**Journal site**
BMJ<br>http://www.bmj.com/theBMJ
BMJ Quality and safety<br>http://qualitysafety.bmj.com/
Websites and organisations
The Health Foundation

Identify relevant studies

The search results would be imported into reference management database *Endnote.* Duplicate references and clearly irrelevant citations will be removed. All remaining studies will then be downloaded into Dropbox for reviewers to screen for relevance.

Medline Search Strategy: Adult Early Warning Systems

1. ("early warning" adj5 scor*).ab,ti.
2. ("early warning" adj5 system* adj5 (deteriorat* or mortality or death or outcome* or harm* or safety)).ab,ti.
3. "acute illness severity".mp.
4. "early medical intervention"/ and ((prevent* or reduc* or improv*) adj5 (deteriorat* or mortality or death or outcome* or harm* or safety)).ab,ti.
5. ("early medical intervention" adj5 (tool* or scor* or index* or indicator* or indice* or assessment* or guide* or instrument* or criteria or parameter* or deteriorat* or mortality or death or monitor* or outcome* or harm* or safety)).ab,ti.
6. exp Health Status Indicators/ and ((tool* or scor* or index* or indicator* or indice* or assessment* or instrument* or criteria or parameter*) adj3 ((prevent* or reduc* or improv*) adj3 (deteriorat* or mortality or death or outcome* or harm* or safety))).ab,ti.
7. "Severity of Illness Index"/ and ((tool* or scor* or index* or indicator* or indice* or assessment* or instrument* or criteria or parameter*) adj5 ((prevent* or reduc* or improv*) adj5 (deteriorat* or mortality or death or outcome* or harm* or safety))).ab,ti.
8. exp Hospitals/ and ((Detecting or managing) adj3 deterioration).tw.
9. ("medical device" adj3 (prevent* or reduc* or improv*)).mp.
10. ("alert criteria" or "activation criteria" or "calling criteria").tw.
11. Hospital Rapid Response Team/
12. Clinical Alarms/
13. (outreach adj3 emergency).tw.
14. VitalPAC Early Warning Score.tw.
15. medical emergency team.tw.
16. Rapid Response Systems.mp.
17. Rapid Response Team.tw.
18. (((Detecting or managing) adj3 deterioration) and warning).tw.
19. track-and-trigger system.tw.

For peer review only

20. (Track adj trigger).tw.
21. (Track and trigger).tw.
22. trigger tools.tw.
23. SBAR technique*.mp.
24. ("rapid response" adj5 (prevent* or reduc* or improv*)).tw.
25. (score adj3 severity of illness).tw.
26. or/1-25
27. (qualitative or ethnograph* or thematic analysis or grounded theory or audio-recorded or transcribed or verbatim or ethnograph* or content analysis technique).ti,ab.
28. (("semi-structured" or semistructured or unstructured or informal or "in-depth" or indepth or "face-to-face" or structured or guide) adj3 (interview* or discussion* or questionnaire*)).ti,ab.
29. ((field or case) adj (stud* or research)).ti,ab.
30. Focus groups/ or Qualitative research/ or Interviews as topic/ or Interview, Psychological/ or ((focus or discussion) adj group*1).ti,ab.
31. (Questionnaires/ or interviews as topic/ or interview, psychological/) and (experience* or predictor* or determinant* or barrier* or facilitator* or enabler* or factor* associat* or perception* or perceive* or attitude* or view*1 or viewpoint* or standpoint* or encounter* or experience* or story or stories or narrative*1 or theme*1 or opinion* or concerns or motivat* or need*1).ti,ab.
32. (cross-sectional studies/ or cross-sectional survey.ti,ab. or correlation study.ti,ab.) and (predictor* or determinant* or barrier* or facilitator* or enabler* or factor* associat* or perception* or perceive* or attitude* or view*1 or viewpoint* or standpoint* or encounter* or experience* or story or stories or narrative*1 or theme*1 or opinion* or concerns or motivat* or need*).ti,ab.
33. process evaluation/ or process evaluation.ti,ab.
34. mixed method*1.ti,ab.
35. ((assoc* factor*1 or predictor* or determinant* or barrier* or facilitator* or enabler*) adj3 (interview* or survey* or questionnaire* or study)).ti,ab.
36. *motivation/
37. ((perception* or perceive* or attitude* or view*1 or viewpoint* or standpoint* or encounter* or experience* or story or stories or narrative*1 or description* or theme* or opinion* or need*1 or concerns or motivat*) adj3 (interview* or survey* or questionnaire* or study or explor* or evaluate or investigate* or analys* or collect*)).ti,ab.
38. (themes adj3 (identif* or analy* or review or explor* or investigat*)).ti,ab.
39. "attitude of health personnel"/ or *attitude to health/
40. exp emotions/
41. consumer satisfaction/
42. personal satisfaction/
43. exp professional-patient relations/
44. exp interprofessional relations/
45. "Health Services Needs and Demand"/
46. or/27-45
47. 26 and 46
48. limit 47 to (english language and humans and yr="1995 -Current")

Medline Search Strategy: Structured communication tools for handover and handoff

For peer review only

1. Situation Background Assessment Recommendation.tw.
2. SBAR.mp.
3. (ABC and "collaborative care").tw.
4. Patient Handoff/
5. Patient Discharge/ and ABC.mp.
6. exp Patient Transfer/ and checklist.mp.
7. (handoff adj3 communication).tw.
8. (handoff adj5 (tool or approach or technique or method)).tw.
9. ("information transfer" and "emergency care").tw.
10. Patient Discharge/ and information transfer.tw.
11. ("information transfer" and "critical care").tw.
12. ("information transfer" and handoff).tw.
13. written checklist.tw.
14. ("Rapid Syndrome Validation Project" or RSVP).tw.
15. "Communication tools".tw.
16. "Escalation of care".tw.
17. or/1-16
18. limit 17 to (english language and humans and yr="1995 -Current")

Medline Search Strategy: Paediatric Early Warning Systems to include observation and training

1. ("early warning" adj5 scor*).ab,ti.
2. ("early warning" adj5 system* adj5 (deteriorat* or mortality or death or outcome* or harm* or safety)).ab,ti.
3. "acute illness severity".mp.
4. "early medical intervention"/ and ((prevent* or reduc* or improv*) adj5 (deteriorat* or mortality or death or outcome* or harm* or safety)).ab,ti.
5. ("early medical intervention" adj5 (tool* or scor* or index* or indicator* or indice* or assessment* or guide* or instrument* or criteria or parameter* or deteriorat* or mortality or death or monitor* or outcome* or harm* or safety)).ab,ti.
6. exp Health Status Indicators/ and ((tool* or scor* or index* or indicator* or indice* or assessment* or instrument* or criteria or parameter*) adj3 ((prevent* or reduc* or improv*) adj3 (deteriorat* or mortality or death or outcome* or harm* or safety))).ab,ti.
7. "Severity of Illness Index"/ and ((tool* or scor* or index* or indicator* or indice* or assessment* or instrument* or criteria or parameter*) adj5 ((prevent* or reduc* or improv*) adj5 (deteriorat* or mortality or death or outcome* or harm* or safety))).ab,ti.
8. exp Hospitals/ and ((Detecting or managing) adj3 deterioration).tw.
9. ("medical device" adj3 (prevent* or reduc* or improv*)).mp.
10. ("alert criteria" or "activation criteria" or "calling criteria").tw.
11. Hospital Rapid Response Team/
12. Clinical Alarms/
13. (outreach adj3 emergency).tw.
14. VitalPAC Early Warning Score.tw.
15. medical emergency team.tw.

For peer review only

16. Rapid Response Systems.mp.
17. Rapid Response Team.tw.
18. (((Detecting or managing) adj3 deterioration) and warning).tw.
19. track-and-trigger system.tw.
20. (Track adj trigger).tw.
21. (Track and trigger).tw.
22. trigger tools.tw.
23. SBAR technique*.mp.
24. ("Situation Background Assessment Recommendation" or SBAR).tw.
25. (patient* adj3 deteriorat*).tw.
26. (deterioration adj3 hospital).tw.
27. (patient deterioration adj4 hospital).tw.
28. (Patients adj4 adverse event).tw.
29. clinical deterioration.tw.
30. ("rapid response" adj5 (prevent* or reduc* or improv*)).tw.
31. (score adj3 severity of illness).tw.
32. Vital signs.tw.
33. or/1-26
34. P?ediatric Early Warning.mp.
35. p?ediatric alert.tw.
36. p?ediatric early warning systems.mp.
37. p?ediatric risk of mortality.tw.
38. P?ediatric Rapid Response Team.tw.
39. Point-of-Care Systems/ and ((paediatric or pediatric) adj3 (improve or identify or detect* or outcome or early or critical or emergency)).tw.
40. P?ediatric Advanced Warning Score.tw.
41. neonatal early warning.tw.
42. infant early warning.tw.
43. p?ediatric rapid response.tw.
44. Bedside paediatric early warning.tw.
45. Bedside PEWS.tw.
46. or/34-45
47. 33 or 46
48. Health Plan Implementation/
49. (implement* or applicat* or execute).tw.
50. (observ* or monitoring or monitor or education).tw.
51. Risk Assessment/
52. Education/
53. Education, Continuing/
54. "Hospitals, Teaching"/
55. Decision Making/
56. Safety Management/
57. Patient Simulation/
58. Awareness/
59. Knowledge/
60. *"Attitude of Health Personnel"/
61. *"Education, Medical, Continuing"/

For peer review only

62. *"Interdisciplinary Communication"/
63. Communication/
64. Monitoring, Physiologic/
65. Decision Making/
66. Judgment/
67. Needs Assessment/
68. Interprofessional Relations/
69. Interdisciplinary Communication/
70. ((organi#ation* adj2 (structur* or form* or function* or determinant* or factors or environme nt* or process* or culture*)) and (outcome? or perform* or satisf* or efficien* or effective* or equ* or growth or develop* or justice or quality or culture* or manage* or leader*)).tw.
71. exp *psychology, industrial/ or *absenteeism/ or *efficiency/ or *job satisfaction/ or *"task performance and analysis"/ or *"time and motion studies"/ or *work simplification/ or *time management/ or *vocational guidance/
72. Decision Support Systems, Clinical/
73. Workflow/
74. or/48-73
75. Nurses/
76. Physicians/
77. (nurse* or physician* or doctor*).tw.
78. Medical Staff/
79. Medical Staff, Hospital/ or Nursing Staff, Hospital/ or Intensive Care Units/
80. or/75-79
81. 47 and 74 and 80
82. limit 81 to (english language and humans and yr="1995 -Current")

Medline Search Strategy: Interventions to improve situational awareness

1 Patient Safety/ (6742)

2 patient safety.tw. (13735)

3 1 or 2 (18307)

4 "Situation* Awareness".tw. (488)

5 3 and 4 (69)

6 limit 5 to (english language and humans and yr="1995 -Current")

For peer review only

Appendix 3; Data extraction template 2

Study/Paper	
Authors, title, date, source	
Purpose	
Design/Methods	
Participants/Setting	
Data type	
Theoretical framework	
Quality Assessment	
Formal study finding	

**Theory Area 1 – Properties of the monitoring system in EWS**
**What are the technical characteristics of the monitoring system?**
**What are the policies/processes associated with the monitoring system?**
**What are roles and responsibilities of the monitoring system?**
**What are the skills and resources needed to enact the monitoring system?**
**How often is the monitoring system applied?**
**How effective is the monitoring system?**
**What is the mechanisms (implicit or explicit) by which the monitoring system has its effects?**
**What actions are necessary to ensure the effectiveness of the monitoring system?**
**What is the relationship of the monitoring system and the context?**
**What is the relationship of the monitoring system to the recording system?**
Is the evidence provided in this theory area good and relevant enough to be included in the synthesis?

**Theory Area 2 – Properties of the recording system in EWS**
**What are the technical characteristics of the recording system?**
**What are the policies/processes associated with the recording system?**

For peer review only

What are the roles and responsibilities of the recording system?
How effective is the recording system?
What are the skills and resources needed to enact the recording system?
What is the mechanism (implicit or explicit) by which the recording system has its effects?
What actions are necessary to ensure the effectiveness of the recording system?
What is the relationship between the recording system and the context?
What is the relationship between the recording system, the monitoring and detection system?
Is the evidence provided in this theory area good and relevant enough to be included in the synthesis?

Theory Area 3 – Properties of the detection/trigger system in EWS
What are the technical characteristics of the detection/trigger system?
What are the policies/processes associated with the detection/trigger system?
What are the roles and responsibilities involved in the detection/trigger system?
What are the skills and resources needed to enact the detection system?
How effective is the trigger system?
What is the mechanism (implicit or explicit) by which the detection/trigger system has its effects?
What actions are necessary to ensure the effectiveness of the detection/trigger system?
What is the relationship between the trigger system and the context?
What is the relationship between the trigger system and the activation system?
Is the evidence provided in this theory area good and relevant enough to be included in the synthesis?

Theory Area 4 – Properties of the activation system in EWS
What are the technical characteristics of the activation system?
What are the policies/processes associated with the activation system?

For peer review only

**What are the roles and responsibilities involved in the activation system?**
**What are the skills and resources needed to enact the activation system?**
**How effective is the activation system?**
**What is the mechanism (implicit or explicit) by which the activation system has its effects?**
**What actions are necessary to ensure the effectiveness of the activation system?**
**What is the relationship of the activation system and the context?**
**What is the relationship between the activation system and the monitoring, recording and trigger system?**
Is the evidence provided in this theory area good and relevant enough to be included in the synthesis?

**Theory Area 5 – Properties of family involvement in EWS**
**At what stage in the PEWS process are families involved?**
**What are the roles and responsibilities involved in ensuring family involvement?**
**How effective is family involvement in the EWS process?**
**What are the mechanisms by which families are involved in EWS?**
**How effective are mechanisms for involving families in EWS?**
**What are the benefits and dis-benefits of family involvement?**
Is the evidence provided in this theory area good and relevant enough to be included in the synthesis?

**Theory Area 6 – Properties of chart design in EWS**
**What are the optimal properties of chart design to ensure effective monitoring?**
**What are the optimal properties of chart design to ensure effective recording?**
**What are the optimal properties of chart design to ensure effective detection?**
Is the evidence provided in this theory area good and relevant enough to be included in the synthesis?

For peer review only

Theory Area 7– Properties of implementation and normalisation in EWS
**What are the barriers to implementation and normalisation of EWS?**
**What actions are necessary to overcome barriers to the implementation and normalisation of EWS?**
**What actions are effective in overcoming barriers to the implementation and normalisation of EWS?**
**What are the mechanisms by which effective actions in overcoming barriers to implementation and normalisation of EWS have their effects?**
**What are the key pillars of an effective reflexive monitoring system?**
Is the evidence provided in this theory area good and relevant enough to be included in the synthesis?

For peer review only

For peer review only

PRISMA 2009 Checklist

Section/topic	#	Checklist item	Reported on page #
**TITLE**			
Title	1	Identify the report as a systematic review, meta-analysis, or both.	1
**ABSTRACT**			
Structured summary	2	Provide a structured summary including, as applicable: background; objectives; data sources; study eligibility criteria, participants, and interventions; study appraisal and synthesis methods; results; limitations; conclusions and implications of key findings; systematic review registration number.	2-3
**INTRODUCTION**			
Rationale	3	Describe the rationale for the review in the context of what is already known.	4
Objectives	4	Provide an explicit statement of questions being addressed with reference to participants, interventions, comparisons, outcomes, and study design (PICOS).	n/a
**METHODS**			
Protocol and registration	5	Indicate if a review protocol exists, if and where it can be accessed (e.g., Web address), and, if available, provide registration information including registration number.	5
Eligibility criteria	6	Specify study characteristics (e.g., PICOS, length of follow-up) and report characteristics (e.g., years considered, language, publication status) used as criteria for eligibility, giving rationale.	n/a
Information sources	7	Describe all information sources (e.g., databases with dates of coverage, contact with study authors to identify additional studies) in the search and date last searched.	7
Search	8	Present full electronic search strategy for at least one database, including any limits used, such that it could be repeated.	7, appendix 2
Study selection	9	State the process for selecting studies (i.e., screening, eligibility, included in systematic review, and, if applicable, included in the meta-analysis).	8
Data collection process	10	Describe method of data extraction from reports (e.g., piloted forms, independently, in duplicate) and any processes for obtaining and confirming data from investigators.	8, appendix 1 & 3
Data items	11	List and define all variables for which data were sought (e.g., PICOS, funding sources) and any assumptions and simplifications made.	n/a
Risk of bias in individual studies	12	Describe methods used for assessing risk of bias of individual studies (including specification of whether this was done at the study or outcome level), and how this information is to be used in any data synthesis.	n/a
Summary measures	13	State the principal summary measures (e.g., risk ratio, difference in means).	n/a
Synthesis of results	14	Describe the methods of handling data and combining results of studies, if done, including measures of consistency (e.g., I^2) for each meta-analysis.	5-6, 8

For peer review only - http://bmjopen.bmj.com/site/about/guidelines.xhtml

PRISMA 2009 Checklist

Section/topic	#	Checklist item	Reported on page #
Risk of bias across studies	15	Specify any assessment of risk of bias that may affect the cumulative evidence (e.g., publication bias, selective reporting within studies).	n/a
Additional analyses	16	Describe methods of additional analyses (e.g., sensitivity or subgroup analyses, meta-regression), if done, indicating which were pre-specified.	8-9, 16-17 table 2
**RESULTS**			
Study selection	17	Give numbers of studies screened, assessed for eligibility, and included in the review, with reasons for exclusions at each stage, ideally with a flow diagram.	9, table 3
Study characteristics	18	For each study, present characteristics for which data were extracted (e.g., study size, PICOS, follow-up period) and provide the citations.	Tables 4, 5 and 6
Risk of bias within studies	19	Present data on risk of bias of each study and, if available, any outcome level assessment (see item 12).	n/a
Results of individual studies	20	For all outcomes considered (benefits or harms), present, for each study: (a) simple summary data for each intervention group (b) effect estimates and confidence intervals, ideally with a forest plot.	n/a
Synthesis of results	21	Present results of each meta-analysis done, including confidence intervals and measures of consistency.	n/a
Risk of bias across studies	22	Present results of any assessment of risk of bias across studies (see Item 15).	n/a
Additional analysis	23	Give results of additional analyses, if done (e.g., sensitivity or subgroup analyses, meta-regression [see Item 16]).	9, box 1
**DISCUSSION**			
Summary of evidence	24	Summarize the main findings including the strength of evidence for each main outcome; consider their relevance to key groups (e.g., healthcare providers, users, and policy makers).	18
Limitations	25	Discuss limitations at study and outcome level (e.g., risk of bias), and at review-level (e.g., incomplete retrieval of identified research, reporting bias).	3
Conclusions	26	Provide a general interpretation of the results in the context of other evidence, and implications for future research.	18-19
**FUNDING**			
Funding	27	Describe sources of funding for the systematic review and other support (e.g., supply of data); role of funders for the systematic review.	4

From: Moher D, Liberati A, Tetzlaff J, Altman DG, The PRISMA Group (2009). Preferred Reporting Items for Systematic Reviews and Meta-Analyses: The PRISMA Statement. PLoS Med 6(7): e1000097. doi:10.1371/journal.pmed1000097

For more information, visit: **www.prisma-statement.org**.

For peer review only - http://bmjopen.bmj.com/site/about/guidelines.xhtml

BMJ Open

Optimising Paediatric Afferent Component Early Warning Systems: A hermeneutic systematic literature review and model development

Journal:	*BMJ Open*
Manuscript ID	bmjopen-2018-028796.R1
Article Type:	Original research
Date Submitted by the Author:	22-Aug-2019

Complete List of Authors:	Jacob, Nina; Cardiff University, Centre for Trials Research<br>Moriarty, Yvonne; Cardiff University, Centre for Trials Research<br>Lloyd, Amy; Cardiff University, Centre for Trials Research<br>Mann, Mala; Cardiff University, University Library Services<br>Tume, Lyvonne N.; University of the West of England Bristol,<br>Sefton, G.; Alder Hey Childrens NHS Fdn Trust,<br>Powell, Colin; Children's Hospital for Wales, Paediatrics<br>Roland, Damian; Paediatric Emergency Medicine Leicester Academic (PEMLA) Group, Emergency Department; University of Leicester Department of Health Sciences, SAPPHIRE group<br>Trubey, Robert ; Cardiff University, Centre for Trials Research<br>Hood, Kerenza; Cardiff University,<br>Allen, Davina; Cardiff University

**Primary Subject Heading**:	Paediatrics
Secondary Subject Heading:	Research methods
Keywords:	STATISTICS & RESEARCH METHODS, PAEDIATRICS, QUALITATIVE RESEARCH

SCHOLARONE™
Manuscripts

Optimising Paediatric Afferent Component Early Warning Systems: A hermeneutic systematic literature review and model development

Authors: Nina Jacob[1†], Yvonne Moriarty[1], Amy Lloyd [1], Mala Mann[2], Lyvonne Tume[3], Gerri Sefton [4], Colin Powell[5, 6], Damian Roland [7,8], Robert Trubey[1], Kerenza Hood[1], Davina Allen[9]

1 Centre for Trials Research, Cardiff University, Heath Park, Cardiff, UK

2 Specialist Unit for Review Evidence (SURE), Cardiff University, Heath Park, Cardiff, UK

3 Faculty of Health and Applied Sciences, University of the West of England Bristol, Bristol, UK

4 Paediatric Intensive Care Unit, Alder Hey Children's NHS Foundation Trust, Liverpool, UK

5 Department of Child Health, Children's Hospital for Wales, Division of Population Medicine School of Medicine, Cardiff University, Cardiff, UK,

6 Department of Pediatric Emergency Medicine, Sidra Medicine, Doha, Qatar

7 Paediatric Emergency Medicine Leicester Academic (PEMLA) Group, Emergency Department, Infirmary Square, Leicester, UK

8 SAPPHIRE Group, Health Sciences, Leicester University, Leicester, UK

9 School of Healthcare Sciences, Cardiff University, Eastgate House, Cardiff, UK

Corresponding author:

†Dr Nina Jacob, Centre for Trials Research, Cardiff University, Heath Park, Cardiff, UK. JacobN@cardiff.ac.uk

Keywords: PEWS, track and trigger scores, early warning scores, clinical deterioration, children, systematic review

Word Count: 4459

For peer review only - http://bmjopen.bmj.com/site/about/guidelines.xhtml

OPTIMISING PAEDIATRIC AFFERENT COMPONENT EARLY WARNING SYSTEMS: A HERMENEUTIC SYSTEMATIC LITERATURE REVIEW AND MODEL DEVELOPMENT

ABSTRACT

Objective: To identify the core components of successful early warning systems for detecting and initiating action in response to clinical deterioration in paediatric in-patients.

Methods: Hermeneutic systematic literature review informed by Translational Mobilisation Theory and Normalisation Process Theory and development of a propositional model of an optimal afferent system. PROSPERO registration number CRD42015015326.

Data sources: Studies of paediatric and adult early warning systems and interventions to support the detection of clinical deterioration and escalation of care.

Results: Detecting deterioration and initiating action in response to clinical deterioration in paediatric in-patients involves several challenges, and the potential failure points in early warning systems are well documented. Track and Trigger Tools (TTTs) are commonly used and have value in supporting key mechanisms of action but depend on certain preconditions for successful integration into practice. Several supplementary interventions have been proposed to improve the effectiveness of early warning systems but there is limited evidence to recommend their wider use, due to the weight and quality of the evidence; the extent to which systems are conditioned by the local clinical context; and the need to attend to system component relationships, which do not work in isolation. While it was not possible to make empirical recommendations for practice, the review methodology generated theoretical inferences about the core components of an optimal system for early warning systems. These are presented as a propositional model conceptualised as three sub-systems: detection, preparation and action.

For peer review only - http://bmjopen.bmj.com/site/about/guidelines.xhtml

Conclusions: There is a growing consensus of the need to think beyond TTTs in improving action to detect and respond to clinical deterioration. Clinical teams wishing to improve early warning systems can use the model to consider systematically the constellation of factors necessary to support detection, preparation and action and consider how these arrangements can be implemented in their local context.

STRENGTHS AND WEAKNESSES OF THE STUDY

- The literature in this field is heterogeneous and better at identifying system weakness than it is effective improvement interventions.
- By deploying social theories and a hermeneutic review methodology it was possible to develop a propositional model of the core components of an afferent component paediatric early warning system.
- The model is derived from logical inferences drawing on the overall evidence synthesis, social theories and clinical expertise, rather than strong empirical evidence of single intervention effectiveness.
- There is a growing consensus of the need to take a whole systems approach to improve the detection and response to deterioration in the in-patient paediatric population and this paper offers an evidence-based framework for this purpose.

This study is funded by the National Institute for Health Research (NIHR) Health Services and Delivery Research (HS&DR) programme (12/178/17). The views and opinions expressed in this paper are those of the authors and not necessarily those of the NHS, the NIHR or the Department of Health.

INTRODUCTION

Failure to recognise and act upon signs of clinical deterioration in the hospitalised child is an acknowledged safety concern (1). Track and Trigger Tools (TTT) are a common response to this problem. A TTT consists of sequential recording and monitoring of physiological, clinical and observational data. When a certain score or trigger is reached then a clinical action should occur including, but not limited to, altered frequency of observation, senior review or more appropriate treatment or

For peer review only - http://bmjopen.bmj.com/site/about/guidelines.xhtml

management. Tools may be paper based or electronic and monitoring can be automated or undertaken manually by staff.

Despite the growing use of TTTs there is limited evidence of their effectiveness as a single intervention in reducing mortality or arrest rates in hospitalised children (2,3). Results from the largest international cluster randomised control trial of a TTT (the Beside PEWS) did not support TTT use to reduce mortality, and highlighted the multifactorial mechanisms involved in detecting and initiating action in response to deterioration (4). These findings lend further weight to a developing consensus about the need to look beyond TTTs to the impact of wider system factors on detecting and responding to deterioration in the in-patient paediatric population (2,5–9). This paper reports on a theoretically informed systematic hermeneutic literature review (10) to identify the core components and mechanisms of action of successful afferent component early warning systems in paediatric hospitals and is one of three linked reviews undertaken as part of a wider UK study commissioned to develop and evaluate an evidence-based paediatric warning system (3,11). It addressed the following question:

What socio-material and contextual factors are associated with successful or unsuccessful Paediatric Early Warning Systems (with or without TTTs)?

METHOD

DESIGN

We performed a hermeneutic systematic review of the relevant literature. A hermeneutic systematic review is an iterative process, integrating analysis and interpretation of evidence with literature searching and is designed to develop a better understanding of the field (10). The popularity of the method is growing in health services research where it has value in generating insights from heterogeneous literatures that cannot be synthesised through standard review methodology (12) and would otherwise produce inconclusive findings (see (9). The purpose of the review was not exhaustive aggregation of evidence, but to develop an understanding of the social, material and contextual factors associated with successful or unsuccessful paediatric early warning systems.

For peer review only - http://bmjopen.bmj.com/site/about/guidelines.xhtml

THEORETICAL FRAMEWORK

Data extraction and interpretation was informed by Translational Mobilisation Theory (TMT) (13,14) and Normalisation Process Theory (NPT) (15,16). TMT is a practice theory which explains how goal-oriented collaborative activity is mobilised in unpredictable environments [Box 1] and how the relevant mechanisms of action are conditioned by the local context. It is well suited for understanding early warning systems which require the organisation of action in evolving conditions, in a variety of clinical environments, with different teams, skill mixes, resources, structures, and technologies. NPT shares the same domain assumptions as TMT and is concerned with 'how and why things become, or do not become, routine and normal components of everyday work' (15,16), directing attention to the preconditions necessary for successful implementation of interventions. The theoretical framework informed our data extraction strategy, interpretation of the evidence, and the development of a propositional model of an optimal paediatric early warning system.

FOCUS OF THE REVIEW

The literature in this field identifies four integrated components which work together to provide a safety system for at risk patients: (1) the afferent component which detects deterioration and triggers timely and appropriate action, (2) the efferent component which consists of the people and resources providing a response; (3) a process improvement component, which includes system auditing and monitoring and (4) an administrative component focusing on organisational leadership and education required to implement and sustain the system (17). Our focus was limited to the afferent components of the system.

STAGES OF THE REVIEW

STAGE 1: SCOPING THE LITERATURE

Literature was identified through a recent scoping review (7), team members' knowledge of the field, hand searches and snowball sampling techniques. The purpose was to (1) inform our review question and eligibility criteria and (2) identify emerging themes and issues. Whilst we drew on several reviews of the literature (5, 12-14) we always consulted original papers. Data were extracted using Data Extraction Template

For peer review only - http://bmjopen.bmj.com/site/about/guidelines.xhtml

1 (Appendix 1) and analysed to produce a provisional conceptual model of the core components of paediatric early warning systems. Additional themes of relevance were identified: family involvement, situational awareness (SA), structured handover, observations and monitoring and the impact of electronic systems and new technologies.

STAGE 2: SEARCHING FOR THE EVIDENCE

We undertook systematic searches of the paediatric and adult early warning system literature (the goals and mechanisms of collective action in detection and rescue trajectories are the same). For the adult literature we used the same search strategies but added a qualitative filter to limit the scope to studies most likely to yield the level of socio-material and contextual detail of value to the review. Literature informing additional areas of interest was located through a combination of systematic and hand searches. Systematic searches (Searches 2 and 3) were undertaken in areas where we anticipated locating evidence of the effectiveness of specific interventions to strengthen early warning systems. Theory driven searches reflected the conceptual requirements of the model development.

SYSTEMATIC SEARCHES

A systematic search was conducted across a range of databases from 1995 to May 2018 to identify relevant studies on the Paediatric Early Warning System literature. An additional three systematic searches were conducted from 1996 to September 2016 to identify supplementary papers to aid in developing understanding on the PEWS literature:

1. Adult Early Warning Systems (EWS)

2. Interventions to improve situational awareness.

3. Structured communication tools for handover and handoff

Detailed information on the search methodology can be found in Appendix 2. Grey literature was excluded in order to keep the review manageable

THEORY DRIVEN SEARCHES

Additional theory-driven searches were conducted in the following areas:

1. Family involvement

For peer review only - http://bmjopen.bmj.com/site/about/guidelines.xhtml

2. Observations and monitoring
3. The impact of electronic systems

These were a combination of exploratory, computerised, snowball, and hand searches. As the analysis progressed, we continued to review new literature on early warning systems as this was published.

SCREENING

After removing duplicates 5284 references were identified for screening. A modified PRISMA flow diagram is provided [Figure 1]. Papers were screened by title to assess eligibility and then by full text to assess relevance for data extraction. The PEWS and adult EWS searches were screened by two researchers, searches 2 and 3 were screened by the lead reviewer.

STAGE 3: DATA EXTRACTION AND APPRAISAL

Data Extraction Template 2 [Appendix 3] was applied to all papers included in the review. As is typical of reviews of this kind, evidential fragments and partial lines of inquiry formed the unit of analysis rather than whole papers (18). These fragments were quality assessed according to the contribution they made to the developing analysis rather than assessing the paper as whole through the use of formal appraisal tools. Data extraction and quality appraisal were undertaken concurrently and double-checked by a second reviewer.

STAGE 4: DEVELOPING A PROPOSITIONAL MODEL

A propositional model was developed specifying the core ingredients of a paediatric early warning system [Table 1]. It comprises of logical inferences derived from the theoretical framework and evidence synthesis, informed by clinical experts on the team. Iterations of the model were developed in collaboration with clinical colleagues. A series of face-to-face meetings were conducted to review structure, wording and applicability to clinical practice.

PATIENT AND PUBLIC INVOLVEMENT

For peer review only - http://bmjopen.bmj.com/site/about/guidelines.xhtml

This review was conducted as part of a larger mixed methods study (ISRCTN94228292), which used a formal, facilitated parental advisory group. The group comprised parents of children who had experienced an unexpected adverse event in a paediatric unit and provided input which helped to shape the broader research questions and wider contextual factors to consider, specifically within the family involvement element of the system. The results of the review will be disseminated to parents through this group.

For peer review only

RESULTS

INCLUDED STUDIES

Eighty-two papers were included in the review. Forty-six papers focussed on TTT implementation and use in paediatric and adult contexts (twenty-four from the paediatric search and the remaining twenty-two from the adult focussed search); the remaining thirty-six papers contributed supplementary data on factors related to the wider warning system. See Table 2 for a detailed breakdown of this process. No studies were located that adopted a whole systems approach to detecting and responding to deterioration.

ANALYSIS

In TMT the primary unit of analysis is the 'project', which defines the social and material actors (people, materials, technologies) and their relationships involved in achieving a particular goal. The goals of the afferent paediatric warning system are first, that the child is identified as at risk and a vital signs monitoring regime instigated; second, that evidence of deterioration is identified through monitoring and categorised as such and; third, that timely and appropriate action is initiated in response to deterioration. Our analysis of the literature suggests that three sub-systems within the afferent component of early warning systems support these processes: the *detection* of signs deterioration; the *preparation* needed to ensure teams are ready to act when deterioration is detected; and the initiation of timely *action*. Whilst we have focussed on the afferent component, it is important to remember that all elements of the overall safety system (efferent component, process improvement

For peer review only - http://bmjopen.bmj.com/site/about/guidelines.xhtml

and administrative arm) need to be working in concert in order to maintain an optimal paediatric early warning system. In the next section we report on the literature in relation to each subsystem.

DETECTION

The goal of the detection sub-system is to recognise early signs of deterioration, so the child becomes the focus of further clinical attention. This requires first, that the child is identified as at risk and a vital sign monitoring regime instigated and second, that the child is identified as showing signs of deterioration.

Despite widespread use the evidence on TTT effectiveness in predicting adverse outcomes in hospitalised children is weak (3). Many TTT have only been validated retrospectively and post predictive value were generally low. Studies reporting significant decreases in cardiac arrest calls or mortality had methodological concerns. The literature does suggest that TTTs have value in supporting process mechanisms in the detection sub-system. Vital signs monitoring is undertaken on all hospital in-patients and, like other high-volume routine activity, is often delegated to junior staff (19-38) who may not have sufficient skills to interpret results (21,22,37). TTTs have value in mitigating these risks: by specifying physiological thresholds that indicate deterioration they take knowledge to the bedside and act as prompts to action (19,39) which can lead to a more systematic and frequent approach to monitoring and improved detection of deterioration (40,41).

TTT's effectiveness in fulfilling these functions depends on certain preconditions. The review highlighted that TTT use was impacted by the availability of appropriate and functioning equipment (22,27,29,34,39,42–46), (in)adequate staffing and night time pressures (22,26,29,30,37,40,42-44,47-52) and an appropriately skilled workforce (26,28,36,43,49,50,53-57). On this latter point, while several papers report on education packages to improve the detection of deterioration, the evidence is not robust enough to recommend specific programs (23–25,28,30,35,55,58–60). There were also times whereby nursing staff prioritised sleep over waking a patient to take vital signs (46,61).

TTTs are also used differently depending on the experience of the user. For juniors, they provide a methodology and structure for monitoring clinical instability and

For peer review only - http://bmjopen.bmj.com/site/about/guidelines.xhtml

identifying deterioration, whereas more experienced staff reportedly use TTTs as confirmatory technologies (19-32). The importance of professional intuition in detecting deterioration is extensively reported across the literature (19-22,26,27,29,31,32,36-40,42,43,45,46,48,50-53,60,62-68) and several authors recommend the inclusion of 'staff concern' in tool criteria (26,48,51,57). This is important; TTTs may be of less value in patients with chronic conditions because of altered normal physiology or where subtle changes are difficult to detect (64). It is also the case that TTTs are implemented in contexts governed by competing organisational logics which impact on their value and use (43,54,58). For example, Iddrisu et al. (57) show TTTs have limited value immediately after surgery because acceptable vital signs parameters are different in the immediate post-operative period.

There is growing interest in the literature in strategies that facilitate patient and relative involvement in the early detection of deterioration (69,70). Healthcare professionals depend on families to explain their child's normal physiological baseline and identify subtle changes in their child's condition but this information is not always systematically obtained (71,72). Some authors propose family involvement in interdisciplinary rounds[1] (73), but this requires parents to have detailed information about the signs and symptoms they should be attending to (72) and as yet there is little evidence on effective strategies for how they might be involved in the detection of deterioration (73).

While much of the literature reports on intermittent manual vital signs monitoring and paper-based recording systems, across the developed world there is a growing use of electronic technologies, which have important implications for the wider detection sub-system (74). We considered a number of evaluations of new technologies which indicated that electronic vital signs recording is associated with a number of positive outcomes, particularly timeliness and accuracy, when compared with paper-based systems (75,76). They can provide prompts or alerts for monitoring (77–79), which facilitates better recognition of deterioration and is associated with a reduction in mortality (78,80). These studies tend to evaluate new technologies in isolation, however, and do not engage with the literature highlighting alarm fatigue which is known to mitigate effectiveness over time or concerns about over burdening staff with

[1] This is an editorial paper

For peer review only - http://bmjopen.bmj.com/site/about/guidelines.xhtml

alerts (81–83). Moreover, the successful implementation of new technologies is conditioned by the local context. For instance, where manual input into an electronic device is required, access to computers is an essential precondition. When computers were not available staff 'batch' the collection of vital signs before data entry, thereby delaying the timely detection of deterioration (27,45,84). In another study where the electronic system was found to be cumbersome and separated the collection and entry of data from the review of vital signs, verbal reports were favoured to ensure timely communication of information (85). See Table 3 for a summary of the evidence reported.

PREPARATION

Detecting and responding to deterioration involves the coordination of action in conditions of uncertainty and competing priorities. The goal of the 'Preparation' sub-system is to ensure the clinical team are ready to act in the event of evidence of deterioration and is reflected in the growing interest in in the literature on structures to facilitate team situational awareness, group decisions and planning (62).

TTTs have been found to support situational awareness. Their use enabled clinicians to have a 'birds-eye' view over all admitted patients on a ward as well as encouraging staff to consider projected acuity levels of the ward (86). A number of studies also report on 'huddles' in facilitating situational awareness (32,65,87,88). A huddle is a multidisciplinary event scheduled at pre-determined times where members discuss specific risk factors around deterioration and develop mitigation plans. One study combined the introduction of huddles with a "watchstander", a role fulfilled by a charge nurse or senior resident, whose primary function is to know patients at high risk for deterioration (88). These initiatives were associated with a near fifty per cent reduction in transfers from acute to intensive care determined to be unrecognized situation awareness events. A further strategy identified by Goldenhar et al. describes the use of the 'watcher' category to designate a patient as at risk where staff have a 'gut feeling' deterioration is likely (87). A recent study used the category of 'watcher' to create a bundle of expectations to standardize communication and contingency planning. Once a patient was labelled 'a watcher' a series of five specific tasks, such as documentation of physician awareness of watcher status and

For peer review only - http://bmjopen.bmj.com/site/about/guidelines.xhtml

that the family had been notified of the change in the patient's status, needed to completed within two hours (89).

Handovers are integral to clinical communication and contribute to situational awareness. The extensive literature on handover indicates that information sharing can be of variable quality (47,54,90) and there is growing evidence that structured approaches improve this (30,47,54,63,87,90–94). Ranging from a checklist system (91,93) to a cognitive aid developed through consensus (23,94), most of the published interventions are variations of the SBAR (Situation, Background, Assessment, and Recommendation) tool (54,90–92). While effective handover depends on communicative forms that extend beyond the information transfer that is typically the focus of structured handover tools (90), in the context of early warning systems a lack of standardisation allows greater margin for individualistic practices and difficulties accessing complementary knowledge and establishing shared understandings (47).

There is also a literature on the use of common information spaces – such as white boards – in facilitating situational awareness in the healthcare team (23,33,47,53,55,58,67). These should be in a visible location and colour-coded to correspond with the TTT score, where relevant (47,55,58). Electronic systems automate this information and allow information to be reviewed remotely. However, they disconnect vital signs data from the patient and hence other indicators of clinical status and access to data is contingent upon the availability of computers (27,45,47,84,95).

The literature indicates that situational awareness can be facilitated in different ways in different contexts and it is the relationship between system elements that is important (47). In their study on situational awareness in delivery suites, Mackintosh et al. discuss the three main supports for situational awareness - whiteboard, handover and coordinator role - and illustrate how these interacted in organisations with strong situational awareness compared to those with reduced levels. Crucially this 'interplay' between the different activities was highly context dependent; "the same supports used differently generate different outcomes" pg52 (47). See table 4 for a summary of the preparation evidence.

ACTION

For peer review only - http://bmjopen.bmj.com/site/about/guidelines.xhtml

The goal of the “Action” sub-system is to initiate appropriate action in response to evidence of deterioration. The literature suggests that mobilising action across professional boundaries/hierarchies is challenging, with differences in language between doctors and nurses and power dynamics contributory factors (27,40,41,50,52,57,60,96). TTTs are in part a response to the challenges of communication in mobilising action in response to deterioration. By transforming a series of discrete observations into a summative indicator of deterioration – such as a score or a trigger – TTTs ‘translate’ and package the patient’s status into a form that can be readily communicated enabling individual level clinical data to be synthesised, made sense of and shared (19-29,33,39,41,42,46,48,50,51,56,62,66,74,86). One study, however, found that TTT were regarded as a nursing tool and were therefore not valued by clinicians. Consequently, nurses encountered difficulties in summoning a response (46).

Several studies also report on the use of SBAR in this context. Like TTTs, SBAR translates information into a form that provides structure, consistency and predictability when presenting patient information. SBAR has been shown to help establish common language and expectations, minimising differences in training, experience and hierarchy and facilitating nurse-clinician communication. While several papers advocate combining SBAR with TTTs (23,25,27,30,35,45,50), none specifically evaluated SBAR use. Mackintosh et al. highlight that audit data suggests resistance to SBAR, with others cautioning that overextending SBAR use carries the risk of SBAR fatigue and attenuation of its effects (27).

Structured communication tools like TTTs and SBAR do not solve all the challenges of acting in response to evidence of deterioration. Barriers to action were widely reported in the literature where these tools were in place. These include: a general disinclination to seek help (19-22,25,27,29,31,36-39,42,48,50,51,56,64,67), concerns about appearing inadequate in front of colleagues (20,22,36,38,50,67), and failure of staff to invest in the escalation or calling criteria (21,22,49). A number of papers also reported negative attitudes to Rapid Response Team (RRT) or Medical Emergency Team (MET) use in the efferent component of safety systems. METs and RRTs operate outside the immediate medical team and create different issues in paediatric warning systems than when the escalation response is managed by the treating team. These include a reluctance to activate because of the perceived busyness of Paediatric

For peer review only - http://bmjopen.bmj.com/site/about/guidelines.xhtml

Intensive Care Unit or medical staff (20,29,39,48,50,51), because previous expectations about an appropriate response were not met, or a sense that the situation was under control (particularly when the physiological instability is in the area of expertise of the treating team) (22,29,31,38,42,50,52,64).

No literature reported on successful interventions to facilitate RRT use, but several propose strategies to support escalation where there was no designated response team in place in the efferent component. These includes informal peer-support, where inexperienced staff team up with more experienced staff (21,29,50,64,67); clear structures to support action and a supportive culture that does not penalise individual decision-making, including the use of a 'no false alarms' policy so staff are not deterred from escalating care (21,29,73, 36). Senior leadership is consistently identified as important (8,20-23,25,27,30,32,33,35,47,52,58,66,67); lack of support from superiors meant that staff are less likely to escalate and more likely to adhere to hierarchies within the current system (25,40,66). There is some evidence to suggest that any escalation policy should be linked to an administrative arm that reinforces the system, measures outcomes and works to ensure an effective system (27,30).

There is a small literature on family involvement in the Action Subsystem. Several studies report on Condition-Help, a program developed in the United States to support families to directly activate a RRT if they have concerns about their child's condition. Families are also becoming increasingly recognised as playing a key role in the activation of RRTs in Australia (97). Research has evaluated the appropriateness of calls that were made by patients or relatives (33,97–101) but has not considered why calls were *not* made (70). Involving family members in escalation demands vigilance, requiring them to take a proactive and interactive role with staff with potentially some degree of confrontation, particularly if challenging the appropriateness of decisions taken (73,97). Families need both cognitive and emotional resources to raise concerns that involve negotiating hierarchies and boundaries (35,70). The literature points to a degree of professional resistance to family involvement in activation, with reports of physician concern that their role would be undermined, that resources would be stretched with an increase in calls and that it might divert attention away from those in need (71,97,99,102,103) although these fears are not supported by the evidence (71,102,104). See Table 5 for a summary of the evidence relating to the action component of the model.

For peer review only - http://bmjopen.bmj.com/site/about/guidelines.xhtml

SYNTHESIS & MODEL DEVELOPMENT

The literature in this field is heterogeneous and stronger on the socio-material barriers to successful afferent component peadiatric early warning systems than it is on solutions. While a number of different single interventions have been proposed and some have been evaluated, there is limited evidence to recommend their use beyond the specific clinical contexts described in the papers. This reflects both the weight and quality of the evidence, the extent to which paediatric systems are conditioned by the local clinical context, and also the need to attend to the relationship between system components and inteventions which work in concert not in isolation. There is also a growing realisation in the quality improvement field that an intervention that has been successful in one context does not necessarily produce the same results elsewhere which cautions against a 'one size fits all' approach (105,106).

While it is not possible to make empirical recommendations for practice, a hermeneutic review methodology enabled the generation of theoretical inferences about the core components of an optimal paediatric early warning system. These model components are logical inferences derived from an overall synthesis of the evidence, informed by our theoretical framework and clinical expertise. These are presented as a propositional model conceptualised as three sub-systems: detection, preparation and action (see Table 1).

DISCUSSION

This paper reports on one of three linked reviews undertaken as part of a wider UK study commissioned to develop and evaluate an evidence-based national paediatric early warning system (3). Drawing on TMT and NPT we have synthesised and analysed the findings from the review to develop a propositional model to specify the core components of optimal afferent component paediatric early warning systems. While there is a growing consensus of the need to think beyond TTTs to consider the whole system, no frameworks exist to support such an approach. Clinical teams wishing to improve rescue trajectories should take a whole systems perspective focused on the constellation of factors necessary to support detection, preparation and action and consider how these relationships can be managed in their local setting. TTTs have value in paediatric early warning systems but they are not the sole solution and depend on certain preconditions for their use. An emerging literature highlights

For peer review only - http://bmjopen.bmj.com/site/about/guidelines.xhtml

the importance of preparation and indicates that combinations of interventions may facilitate situation awareness. Professional judgement is also important in detecting and acting on deterioration and the evidence points to the importance of a wider organisational culture that is supportive of this. Innovative approaches are needed to support family involvement in all aspects of paediatric early warning systems, which are sensitive to the cognitive and emotional resources this requires. System effectiveness requires attention to the socio-material relationships in the local context, senior support and leadership and continuous monitoring and evaluation. New technologies, such as moving from paper based to electronic TTTs, has important implications for all three sub-systems and critical consideration should be given to their wider impacts and the preconditions for their integration into practice.

LIMITATIONS OF THE REVIEW

The literature in this field is heterogeneous and better at identifying system weakness than it is effective improvement interventions. It was only by deploying social theories and a hermeneutic review methodology did it prove possible to develop a propositional model of the core components of an afferent component paediatric early warning system. This model is derived from logical inferences drawing on the overall evidence synthesis, social theories and clinical expertise, rather than strong empirical evidence of single intervention effectiveness. Consequently, there is a growing consensus of the need to take a whole systems approach to improve the detection and response to deterioration in the in-patient paediatric population.

CONCLUSION

Failure to recognise and act upon signs of deterioration is an acknowledged safety concern (1) and TTTs are a common response to this problem. There is, however, a growing recognition of the importance of wider system factors on the effectiveness of responses to deterioration (5) (7). We have reviewed a wide literature and analysed this using social theories to develop a propositional model of an optimal afferent component paediatric early warning system that can be used as a framework for paediatric units to evaluate their current practices and identify areas for improvement. TTT use should be driven by the extent to which teams think that they will help improve the effectiveness of their system as a whole.

For peer review only - http://bmjopen.bmj.com/site/about/guidelines.xhtml

For peer review only

ACKNOWLEDGEMENTS

The authors would like to acknowledge the contribution of Dr Heather Strange to the review. The authors would also like to extend their thanks to the parental advisory group who have helped shape the broader questions of the research study as well as offer guidance on wider contextual factors to consider within the overall paediatric early warning system.

CONTRIBUTORS

NJ: screening and review of papers; led the theoretical synthesis of the literature; contributed to model development; preparation and writing of manuscript (with DA). YM: screening and review of papers; contributed to model development; contributed to the drafting of the manuscript. AL: led the model development (with DA); contributed to the drafting of the manuscript. MM: conceived and led the systematic search strategies; review of manuscript. LT, GS, CP, DR: screening and review of papers; contributed clinical expertise; contributed to model development; contributed to the drafting of the manuscript. RT; screening and review of manuscript. KH: contributed to model development; contributed to the drafting of the manuscript. DA: conceived of and designed the review; led the theoretical framing and analysis; screening and review of papers; led the model development (with AL) and led the writing of the manuscript (with NJ).

For peer review only

COMPETING INTERESTS

None declared.

FUNDING

This study is funded by the National Institute for Health Research (NIHR) Health Services and Delivery Research (HS&DR) programme (12/178/17).

DATA SHARING STATEMENT

For peer review only - http://bmjopen.bmj.com/site/about/guidelines.xhtml

All data relevant to the study are included in the article or uploaded as supplementary information.

For peer review only

For peer review only - http://bmjopen.bmj.com/site/about/guidelines.xhtml

REFERENCES

1. Subbe, C. P. and Welch JR. Failure to rescue: using rapid response systems to improve care of the deteriorating patient in hospital. J Patient Saf Risk Manag. 2013;19(1): 6-11.

2. Chapman SM, Wray J, Oulton K, Peters MJ. Systematic review of paediatric track and trigger systems for hospitalised children. Resuscitation. 2016;109:87–109.

3. Trubey R, Huang C, Lugg-widger F V, Hood K, Allen D, Edwards D, et al. Validity and effectiveness of paediatric early warning systems and track and trigger tools for identifying and reducing clinical deterioration in hospitalised children : a systematic review. BMJ Open. 2019;9:e022105.

4. Parshuram CS, Dryden-Palmer K, Farrell C, Gottesman R, Gray M, Hutchison JS, et al. Effect of a pediatric early warning system on all-cause mortality in Hospitalized pediatric patients: The epoch randomized clinical trial. JAMA - J Am Med Assoc. 2018;319(10):1002–12.

5. The Irish Paediatric Early Warning System (PEWS)(NCEC National Clinical Guideline No. 12). 2015. Department of Health Ireland.

6. Rihari-Thomas J, Newton PJ, Sibbritt D, Davidson PM. Rapid response systems: where we have come from and where we need to go? J Nurs Manag. 2018;26(1):1–2.

7. Lambert V, O'Shea M, Walshe C, Matthews A, Corbally M, O'Mathuna D, et al. A systematic literature review to support the development of a National Clinical Guideline – Paediatric Early Warning System (PEWS). 2014. [last accessed August 2019, http://health.gov.ie/wp-content/uploads/2014/03/PEWS-Sytematic-Literature-Review-Oct-2014.pdf]

8. Almblad AC, Siltberg P, Engvall G, Målqvist M. Implementation of Pediatric Early Warning Score; Adherence to Guidelines and Influence of Context. J Pediatr Nurs. 2018;38:33–9.

9. Lambert V, Matthews A, MacDonell R, Fitzsimons J. Paediatric early warning

For peer review only - http://bmjopen.bmj.com/site/about/guidelines.xhtml

systems for detecting and responding to clinical deterioration in children: A systematic review. BMJ Open. 2017;7:e014497.

10. Boell SK, Cecez-Kecmanovic D. A hermeneutic approach for conducting literature reviews and literature searches. Commun Assoc Inf Syst. 2014;34(12):257–86.

11. Thomas-Jones E, Lloyd A, Roland D, Sefton G, Tume L, Hood K, et al. A prospective, mixed-methods, before and after study to identify the evidence base for the core components of an effective Paediatric Early Warning System and the development of an implementation package containing those core recommendations for use in th. BMC Pediatr. 2018;18:244.

12. Greenhalgh T, A'Court C, Shaw S. Understanding heart failure; explaining telehealth - A hermeneutic systematic review. BMC Cardiovasc Disord. 2017;17(1):1–16.

13. Allen D, May C. Organizing Practice and Practicing Organization: An Outline of Translational Mobilization Theory. SAGE Open. 2017;7(2):35–43.

14. Allen D. Translational Mobilisation Theory: A new paradigm for understanding the organisational elements of nursing work. Int J Nurs Stud. 2018;79:36–42.

15. May C, Finch T. Implementing, embedding, and integrating practices: An outline of normalization process theory. Sociology. 2009;43(3):535–54.

16. May CR, Johnson M, Finch T. Implementation, context and complexity. Implement Sci. 2016;11(1):1–12.

17. Jagt EW van der. Improving Pediatric Survival from Resuscitation Events: The Role and Organization of Hospital-based Rapid Response Systems and Code Teams. Curr Pediatr Rev. 2013;9(2):158–74.

18. Pawson R. Evidence-Based Policy: A Realist Perspective. London: Sage; 2006.

19. Andrews T, Waterman H. Packaging: A grounded theory of how to report physiological deterioration effectively. J Adv Nurs. 2005;52(5):473–81.

20. Azzopardi P, Kinney S, Moulden A, Tibballs J. Attitudes and barriers to a Medical Emergency Team system at a tertiary paediatric hospital.

For peer review only

Resuscitation. 2011;82(2):167–74.

21. Bonafide CP, Roberts KE, Weirich CM, Paciotti B, Tibbetts KM, Keren R, et al. Beyond statistical prediction: Qualitative evaluation of the mechanisms by which pediatric early warning scores impact patient safety. J Hosp Med. 2013;8(5):248–53.

22. Braaten JS. Hospital System Barriers to Rapid Response Team Activation: A Cognitive Work Analysis. Am J Nurs. 2015;115(2):22–32.

23. Ennis L. Paediatric early warning scores on a children's ward: a quality improvement initiative. Nurs Child Young People. 2014;26(7):25–31.

24. Kaul M, Snethen J, Kelber ST, Zimmanck K, Maletta K, Meyer M. Implementation of the Bedside Paediatric Early Warning System (BedsidePEWS) for nurse identification of deteriorating patients. J Spec Pediatr Nurs. 2014;19(4):339–49.

25. Lobos AT, Fernandes R, Ramsay T MJ. Patient characteristics and disposition after pediatric medical emergency team (MET) activation: disposition depends on who activates the team. 2014;4:99–105.

26. McDonnell A, Tod A, Bray K, Bainbridge D, Adsetts D, Walters S. A before and after study assessing the impact of a new model for recognizing and responding to early signs of deterioration in an acute hospital. J Adv Nurs. 2013;69(1):41–52.

27. Mackintosh N, Rainey H, Sandall J. Understanding how rapid response systems may improve safety for the acutely ill patient: learning from the frontline. BMJ Qual Saf. 2012;21(2):135–44.

28. Monaghan A. Detecting and managing deterioration in children. Paediatr Care. 2005;17(1):32–5.

29. Pattison N, Eastham E. Critical care outreach referrals: A mixed-method investigative study of outcomes and experiences. Nurs Crit Care. 2012;17(2):71–82.

30. Pearson G, Duncan H. Early warning systems for identifying sick children. Paediatr Child Health (Oxford). 2011;21(5):230–3.

For peer review only - http://bmjopen.bmj.com/site/about/guidelines.xhtml

31. Shearer B, Marshall S, Buist MD, Finnigan M, Kitto S, Hore T, et al. What stops hospital clinical staff from following protocols? An analysis of the incidence and factors behind the failure of bedside clinical staff to activate the rapid response system in a multi-campus Australian metropolitan healthcare service. BMJ Qual Saf. 2012;21(7):569–75.

32. Stewart J, Carman M, Spegman A, Sabol VK. Evaluation of the effect of the modified early warning system on the nurse-led activation of the rapid response system. J Nurs Care Qual. 2014;29(3):223–9.

33. Van Voorhis KT, Willis TS. Implementing a Pediatric Rapid Response System to Improve Quality and Patient Safety. Pediatr Clin North Am. 2009;56(4):919–33.

34. Wheatley I. The nursing practice of taking level 1 patient observations. Intensive Crit Care Nurs. 2006;22(2):115–21.

35. Mccabe A, Duncan H, Heward Y. Paediatric early warning systems: where do we go from here? Paediatr Nurs. 2009;21(1):14–7.

36. Chua WL, Mackey S, Ng EKC, Liaw SY. Front line nurses' experiences with deteriorating ward patients: A qualitative study. Int Nurs Rev. 2013;60(4):501–9.

37. James J, Butler-Williams C, Hunt J, Cox H. Vital signs for vital people: An exploratory study into the role of the Healthcare Assistant in recognising, recording and responding to the acutely ill patient in the general ward setting. J Nurs Manag. 2010;18(5):548–55.

38. Mackintosh N, Humphrey C, Sandall J. The habitus of "rescue" and its significance for implementation of rapid response systems in acute health care. Soc Sci Med. 2014;120:233–42.

39. Endacott R, Westley M. Managing patients at risk of deterioration in rural hospitals: A qualitative study. Aust J Rural Health. 2006;14(6):275–9.

40. Lydon S, Byrne D, Offiah G, Gleeson L, O'Connor P. A mixed-methods investigation of health professionals' perceptions of a physiological track and trigger system. BMJ Qual Saf. 2016;25(9):688–95.

41. Sønning K, Nyrud C, Ravn IH. A survey of healthcare professionals '

For peer review only - http://bmjopen.bmj.com/site/about/guidelines.xhtml

experiences with the Paediatric Early Warning Score (PEWS). Nor J Clin Nurs / Sykepl Forsk. 2018;12:e64605.

42. Cioffi J, Salter C, Wilkes L, Vonu-Boriceanu O, Scott J. Clinicians' responses to abnormal vital signs in an emergency department. Aust Crit Care. 2006;19(2):66–72.

43. Endacott R, Kidd T, Chaboyer W, Edington J. Recognition and communication of patient deterioration in a regional hospital: A multi-methods study. Aust Crit Care. 2007;20(3):100–5.

44. Hands C, Reid E, Meredith P, Smith GB, Prytherch DR, Schmidt PE, et al. Patterns in the recording of vital signs and early warning scores: Compliance with a clinical escalation protocol. BMJ Qual Saf. 2013;22(9):719–26.

45. Watson A, Skipper C, Steury R, Walsh H, Levin A. Inpatient nursing care and early warning scores: A workflow mismatch. J Nurs Care Qual. 2014;29(3):215–22.

46. Jensen CS, Nielsen PB, Olesen HV, Kirkegaard H, Aagaard H. Pediatric Early Warning Score Systems, Nurses Perspective - A Focus Group Study. J Pediatr Nurs. 2018;41:e16–22.

47. MacKintosh N, Berridge EJ, Freeth D. Supporting structures for team situation awareness and decision making: Insights from four delivery suites. J Eval Clin Pract. 2009;15(1):46–54.

48. Cioffi J. Nurses' experiences of making decisions to call emergency assistance to their patients. J Adv Nurs. 2000;32(1):108–14.

49. Adelstein B-A, Piza MA, Nayyar V, Mudaliar Y, Klineberg PL, Rubin G. Rapid response systems: A prospective study of response times. J Crit Care. 2011;26(635):e11-8.

50. S Astroth K, M Woith W, Stapleton SJ, Degitz RJ, Jenkins SH. Qualitative exploration of nurses' decisions to activate rapid response teams. J Clin Nurs. 2013;22(19–20):2876–82.

51. Cioffi J. Recognition of patients who require emergency assistance: A descriptive study. Hear Lung J Acute Crit Care. 2000;29(4):262–8.

For peer review only - http://bmjopen.bmj.com/site/about/guidelines.xhtml

52. Mackintosh N, Watson K, Rance S, Sandall J. Value of a modified early obstetric warning system (MEOWS) in managing maternal complications in the peripartum period: An ethnographic study. BMJ Qual Saf. 2014;23(1):26–34.

53. Davies O, DeVita MA, Ayinla R, Perez X. Barriers to activation of the rapid response system. Resuscitation. 2014;85(11):1557–61.

54. McCrory MC, Aboumatar H, Custer JW, Yang CP, Hunt EA. "aBC-SBAR" training improves simulated critical patient hand-off by pediatric interns. Pediatr Emerg Care. 2012;28(6):538–43.

55. Randhawa S, Roberts-Turner R, Woronick K, DuVal J. Implementing and sustaining evidence-based nursing practice to reduce pediatric cardiopulmonary arrest. West J Nurs Res. 2011;33(3):443–56.

56. Salamonson Y, Heere B van, Everett B, Davidson P. Voices from the floor: Nurses' perceptions of the medical emergency team. Intensive Crit Care Nurs. 2006;22(3):138–43.

57. Mohammmed Iddrisu S, Hutchinson AF, Sungkar Y, Considine J. Nurses role in recognising and responding to clinical deterioration. J Clin Nurs. 2018;27:1920–30.

58. Demmel KM, Williams L, Flesch L. Implementation of the pediatric early warning scoring system on a pediatric Hematology/Oncology unit. J Pediatr Oncol Nurs. 2010;27(4):229–40.

59. McKay H, Mitchell IA, Sinn K, Mugridge H, Lafferty T, Van Leuvan C, et al. Effect of a multifaceted intervention on documentation of vital signs and staff communication regarding deteriorating paediatric patients. J Paediatr Child Health. 2013;49(1):48–56.

60. Radeschi G, Urso F, Campagna S, Berchialla P, Borga S, Mina A, et al. Factors affecting attitudes and barriers to a medical emergency team among nurses and medical doctors: A multi-centre survey. Resuscitation. 2015;88:92–8.

61. Hope J, Recio-Saucedo A, Fogg C, Griffiths P, Smith GB, Westwood G, et al. A fundamental conflict of care: Nurses' accounts of balancing patients' sleep with taking vital sign observations at night. J Clin Nurs. 2018;27(9–10):1860–

For peer review only - http://bmjopen.bmj.com/site/about/guidelines.xhtml

71.

62. de Groot JF, Damen N, Loos E De, Steeg L Van De, Koopmans L, Rosias P, et al. Implementing paediatric early warning scores systems in the Netherlands : future implications. BMC Pediatr. 2018;18:128.

63. Brady PW, Goldenhar LM. A qualitative study examining the influences on situation awareness and the identification, mitigation and escalation of recognised patient risk. BMJ Qual Saf. 2014;23(2):153–61.

64. Donohue LA, Endacott R. Track, trigger and teamwork: Communication of deterioration in acute medical and surgical wards. Intensive Crit Care Nurs. 2010;26(1):10–7.

65. Claussen D, Garner D, Crow B. Early warning signs and the EHR: At the intersection of technology and care. Nurs Manage. 2013;44(11):14–6.

66. Jones D, Baldwin I, McIntyre T, Story D, Mercer I, Miglic A, et al. Nurses' attitudes to a medical emergency team service in a teaching hospital. Qual Saf Heal Care. 2006;15(6):427–32.

67. Massey D, Chaboyer W, Aitken L. Nurses' perceptions of accessing a Medical Emergency Team: A qualitative study. Aust Crit Care. 2014;27(3):133–8.

68. Burns KA, Reber T, Theodore K, Welch B, Roy D, Siedlecki SL. Enhanced Early Warning System Impact on Nursing Practice: A phenomenological study. J Adv Nurs. 2018;74(5):1150–6.

69. Rainey H, Ehrich K, Mackintosh N, Sandall J. The role of patients and their relatives in "speaking up" about their own safety - a qualitative study of acute illness. Heal Expect. 2015;18(3):392–405.

70. Mackintosh, NJ, Davis, RE, Easter, A, Rayment-Jones, H, Sevdalis, N, Wilson, S, Adams, M, Sandall J. Interventions to increase patient and family involvement in escalation of care for acute life-threatening illness in community health and hospital settings (Protocol). Cochrane Database Syst Rev. 2017;(10):CD012829.

71. Paciotti, B, Roberts, KE, Tibbetts, KM, Weirich Paine, C, Keren, R, Barg, F.K., Holmes, J.H. and Bonafide CP. Physician attitudes toward family-activated medical emergency teams for hospitalized children. Jt Comm J Qual

For peer review only

Patient Saf. 2014;40(4):187–92.

72. Graedon J, Graedon T. Enlisting Families as Patient Safety Allies. Clin Pediatr Emerg Med. 2006;7(4):265–7.

73. Entwistle V. Nursing shortages and patient safety problems in hospital care: Is clinical monitoring by families part of the solution? Heal Expect. 2004;7(1):1–5.

74. Downey CL, Tahir W, Randell R, Brown JM, Jayne DG. Strengths and limitations of early warning scores : A systematic review and narrative synthesis. Int J Nurs Stud. 2017;76:106–19.

75. Mohammed M, Hayton R, Clements G, Smith G, Prytherch D. Improving accuracy and efficiency of early warning scores in acute care. Br J Nurs. 2009;18(1):18–24.

76. Sefton G, Lane S, Killen R, Black S, Lyon M, Ampah P, et al. Accuracy and Efficiency of Recording Pediatric Early Warning Scores Using an Electronic Physiological Surveillance System Compared With Traditional Paper-Based Documentation. Comput Inf Nurs. 2017;35(5):228–36.

77. Jones S, Mullally M, Ingleby S, Buist M, Bailey M, Eddleston JM. Bedside electronic capture of clinical observations and automated clinical alerts to improve compliance with an Early Warning Score protocol. Crit Care Resusc. 2011;13(2):83–8.

78. Schmidt PE, Meredith P, Prytherch DR, Watson D, Watson V, Killen RM, et al. Impact of introducing an electronic physiological surveillance system on hospital mortality. BMJ Qual Saf. 2015;24(1):10–20.

79. Bellomo R, Ackerman M, Bailey M, Beale R, Clancy G, Danesh V, et al. A controlled trial of electronic automated advisory vital signs monitoring in general hospital wards. Crit Care Med. 2012;40(8):2349–61.

80. Subbe CP, Duller B, Bellomo R. Effect of an automated notification system for deteriorating ward patients on clinical outcomes. Crit Care. 2017;21(1):1–9.

81. Bonafide CP, Zander M, Graham CS, Weirich Paine CM, Rock W, Rich A, et al. Video methods for evaluating physiologic monitor alarms and alarm responses. Biomed Instrum Technol. 2014;48(3):220–30.

For peer review only - http://bmjopen.bmj.com/site/about/guidelines.xhtml

For peer review only

82. Fagan K, Sabel A, Mehler PS, MacKenzie TD. Vital Sign Abnormalities, Rapid Response, and Adverse Outcomes in Hospitalized Patients. Am J Med Qual. 2012;27(6):480–6.

83. Bonafide CP, Localio AR, Holmes JH, Nadkarni VM, Stemler S, Macmurchy M, et al. Video Analysis of Factors Associated With Response Time to Physiologic Monitor Alarms in a Children's Hospital. 2019;171(6):524–31.

84. Wager KA, Schaffner MJ, Foulois B, Swanson Kazley A, Parker C, Walo H. Comparison of the quality and timeliness of vital signs data using three different data-entry devices. CIN - Comput Informatics Nurs. 2010;28(4):205–12.

85. Stevenson JE, Nilsson G. Nurses' perceptions of an electronic patient record from a patient safety perspective: A qualitative study. J Adv Nurs. 2012;68(3):667–76.

86. Vries A De, Draaisma JMT, Fuijkschot J. Clinician Perceptions of an Early Warning System on Patient Safety. Hosp Pediatr. 2017;7(10):579–86.

87. Goldenhar LM, Brady PW, Sutcliffe KM, Muething SE. Huddling for high reliability and situation awareness. BMJ Qual Saf. 2013;22(11):899–906.

88. Brady PW, Muething S, Kotagal U, Ashby M, Gallagher R, Hall D, et al. Improving Situation Awareness to Reduce Unrecognized Clinical Deterioration and Serious Safety Events. Pediatrics. 2013;131(1):e298–308.

89. Parker MW, Carroll M, Bolser B, Ballinger J, Brewington J, Campanella S. Implementation of a Communication Bundle for High-Risk Patients. Hosp Pediatr. 2017;7(9):523–9.

90. Pezzolesi C, Manser T, Schifano F, Kostrzewski A, Pickles J, Harriet N, et al. Human factors in clinical handover: Development and testing of a "handover performance tool" for doctors' shift handovers. Int J Qual Heal Care. 2013;25(1):58–65.

91. Abraham J, Kannampallil T, Patel B, Almoosa K, Patel VL. Ensuring patient safety in care transitions: an empirical evaluation of a Handoff Intervention Tool. AMIA Annu Symp Proc. 2012;2012:17–26.

92. Donahue M, Smith L, Dykes P, Fitzpatrick JJ. Phase 2 of the EMPOWER

For peer review only

project: enhancing communication for paraprofessionals. J Contin Educ Nurs. 2010;41(5):197–8.

93. Mullan PC, Macias CG, Hsu D, Alam S, Patel B. A novel briefing checklist at shift handoff in an emergency department improves situational awareness and safety event identification. Pediatr Emerg Care. 2015;31(4):231–8.

94. Weiss MJ, Bhanji F, Fontela PS, Razack SI. A preliminary study of the impact of a handover cognitive aid on clinical reasoning and information transfer. Med Educ. 2013;47(8):832–41.

95. Wong D, Bonnici T, Knight J, Morgan L, Coombes P, Watkinson P. SEND: A system for electronic notification and documentation of vital sign observations. BMC Med Inform Decis Mak. 2015;15:68.

96. Preece MHW, Hill A, Horswill MS, Karamatic R, Watson MO. Designing observation charts to optimize the detection of patient deterioriation: Reliance on the subjective preferences of healthcare professionals is not enough. Aust Crit Care. 2012;25(4):238–52.

97. Gill, F.N., Leslie, G.D., Marshall AP. Australian Critical Care Family initiated escalation of care for the deteriorating patient in hospital : Family centred care or just " box ticking ." Aust Crit Care. 2016;29(4):195–200.

98. Bogert S, Ferrell C, Rutledge DN. Experience with Family Activation Of Rapid Response Teams. Medsurg Nurs. 2010;19(4):215–22.

99. Dean BS, Decker MJ, Hupp D, Urbach AH, Lewis E, Benes-Stickle J. Condition HELP: a pediatric rapid response team triggered by patients and parents. J Healthc Qual. 2008;30(3):28–31.

100. Greenhouse PK, Kuzminsky B, Martin SC, Merryman T. Calling a condition H(elp). Am J Nurs. 2006;106(11):63–6.

101. Hueckel RM, Mericle JM, Frush K, Martin PL, Champagne MT. Implementation of condition help: Family teaching and evaluation of family understanding. J Nurs Care Qual. 2012;27(2):176–81.

102. Brady PW, Zix J, Brilli R, Wheeler DS, Griffith K, Giaccone MJ, et al. Developing and evaluating the success of a family activated medical emergency team: A quality improvement report. BMJ Qual Saf.

For peer review only - http://bmjopen.bmj.com/site/about/guidelines.xhtml

2015;24(3):203–11.

103. Gerdik C, Vallish RO, Miles K, Godwin SA, Wludyka PS, Panni MK. Successful implementation of a family and patient activated rapid response team in an adult level 1 trauma center. Resuscitation. 2010;81(12):1676–81.

104. Bavare, A.C Thomas, J.K Elliott, E.P Morgan A.C. Graf J. Family-Initiated Pediatric Rapid Response: Characteristics, Impetus, and Outcomes. J Healthc Qual. 2018;40(2):103–9.

105. Bion J, Richardson A, Hibbert P, Beer J, Abrusci T, McCutcheon M, et al. Matching Michigan: A 2-year stepped interventional programme to minimise central venous catheterblood stream infections in intensive care units in England. BMJ Qual Saf. 2013;22(2):110–23.

106. Dixon-Woods M, Leslie M, Tarrant C, Bion J. Explaining Matching Michigan: An ethnographic study of a patient safety program. Implement Sci. 2013; 8:70.

107. National Patient Safety Agency. Recognising and responding appropriately to early signs of deterioration in hospitalised patients London: NPSA; 2008.

108. Gilson RD. Situation awareness — special issue preface. Hum Factors J Hum Factors Ergon Soc. 1995;37(1):3–4.

TABLES

Box 1: Mechanisms of Translational Mobilisation and their Application to Rescue Trajectories (14)

Object formation - how people draw on the interpretative resources available to them within a strategic action field to create the objects of their practice. Enrolment into an escalation trajectory requires multiple examples of object formation beginning with construction of an individual as at risk of deterioration and a regime of vital signs monitoring instigated, through recognition that the patient's

For peer review only - http://bmjopen.bmj.com/site/about/guidelines.xhtml

physiological status is a cause for concern, to the identification of the patient as requiring a specific intervention. How this is achieved is highly dependent on the features of the local strategic action field.

Translation – the processes that enable practice objects to be shared and different understandings accommodated. It points to the actions necessary in order for a patient that is an object of concern for nursing staff to be translated into a clinical priority for the doctor and, if necessary, to be translated into the focus of intervention by the emergency response team.

Articulation - refers to the secondary work processes that align the actions, knowledge and resources necessary for the mobilization of projects of collective action. It is the work that makes the work, work. Responding to deterioration is time critical and articulation work is necessary to ensure the availability of resources and materials to support clinical management. This is not a mundane observation; catastrophic failures in patient safety are often attributed to the lack of functioning equipment (107) and the absence of monitoring equipment has been identified as a factor undermining the implementation of early warning track and trigger tools (48).Attending to articulation in rescue trajectories also underlines the temporal ordering of action and the mechanisms required to achieve this, directing improvement efforts towards the organization's escalation policy, for example.

Reflexive monitoring refers to the processes through which people collectively or individually appraise and review activity. In a distributed field of action, reflexive monitoring is the means through which members accomplish situational awareness (108) of an overall project. The importance of situation awareness in rescue trajectories is well recognised, but achieving this is challenging. Reflexive monitoring is conditioned by the wider institutional context which will include a multiplicity of informal and formal mechanisms designed for this purpose: nursing and medical handovers, the ward round, safety briefings. The form, frequency and effectiveness of these processes in supporting detecting and acting on deterioration would need to be taken into account in any improvement initiative.

For peer review only - http://bmjopen.bmj.com/site/about/guidelines.xhtml

**Sensemaking -** refers to the processes through which agents create order in conditions of complexity. It draws attention to how the material and discursive processes by which members organise their work, account for their actions, and construct the objects of their practice also give meaning and substance to the institutional components of strategic action fields that shape activity and condition future activity.

For peer review only

For peer review only - http://bmjopen.bmj.com/site/about/guidelines.xhtml

For peer review only

For peer review only - http://bmjopen.bmj.com/site/about/guidelines.xhtml

TABLE 1: PROPOSITIONAL MODEL

	Proposition	Conceptual requirements	Operationalisation	Tool and activities	Proposed effect of interventions
DETECTION	Detection of deterioration depends on collecting and interpreting information from children, through accurate, timely and appropriate monitoring of vital signs and relevant risk factors.	At a minimum, this requires:<br>• Staff are aware of which core and additional vital signs need to be monitored<br>• Staff are aware of the minimum frequency of observations required for the children in their care<br>• Staff are aware of the need to review the frequency of observations for children in their care<br>• Staff are aware of prior factors that increase children's risk of	At a minimum, this requires:<br>• Tasks are allocated to staff members with appropriate skills to conduct them<br>• Staff receive guidance about:<br>- Which core and additional vital signs need to be monitored<br>- Minimum frequency of observations required for children in their care<br>- Prior factors that increase children's risk of deterioration<br>- Which additional	• Skills training: accurate, timely and appropriate monitoring<br>• Focused education: recognising signs of deterioration<br>• Induction: staff aware of monitoring processes in a specific ward<br>• Observations policy<br>• Track and Trigger Tool<br>• Regular equipment checks	• Staff are aware of which core and additional indicators need to be monitored<br>• Staff are aware of the frequency of observations required for the children in their care<br>• Staff have appropriate skills to regularly monitor and accurately record key physiological parameters<br>• Staff are aware of roles and responsibilities

For peer review only - http://bmjopen.bmj.com/site/about/guidelines.xhtml

		deterioration (e.g. premature birth)<br>• Staff are aware of additional clinical assessments required for children with prior risk factors<br>• Staff are aware of which additional clinical assessments are appropriate for children in their care with prior risk factors<br>• Staff have appropriate skills to monitor and accurately record vital signs<br>• Staff have access to appropriate equipment to monitor and accurately record vital signs, and	clinical assessments are appropriate for children in their care with prior risk factors<br>- Which members and grades of staff are responsible for monitoring and recording vital signs<br>- How to record and accumulate observations (e.g. use of a device)<br>• Staff discuss and agree appropriate frequency of observations for individual patients<br>• Equipment required to monitor and accurately record vital signs is readily available		• Observations of key physical parameters are structured, accumulated and displayed so that patterns and trends can be readily detected, and staff are prompted to make observations at the required frequency.

For peer review only - http://bmjopen.bmj.com/site/about/guidelines.xhtml

		conduct other clinical assessments<br>• Staff are aware of roles and responsibilities<br>• Staff have time to conduct accurate timely and appropriate monitoring of vital signs, alongside other work commitments<br>• Staff use a standardised approach to assessing illness severity	• Equipment required to monitor and accurately record vital signs is functioning as it should<br>• There are an appropriate number of staff to carry out required tasks<br>• Staff use a device for recording and accumulating key vital signs		

For peer review only

DETECTION	Detection of deterioration depends on supporting and enabling staff to detect other signs of deterioration and express concerns	At a minimum this requires:<br>• Teams have appropriate skills to discern patterns and trends of signs and symptoms<br>• Work is organised to enable staff to recognise patterns and trends<br>• Staff are encouraged and supported in raising concerns<br>• Staff are encouraged and supported to share learning	At a minimum this requires:<br>• Work to be organised to facilitate continuity of care<br>• Work to be organised to facilitate communication if continuity of care is breached<br>• Staff concern formally recognised as a valid indicator of deterioration<br>• Staff concerns are positively received and acted upon, irrespective of the outcome<br>• Staff receive guidance on how to raise concerns about patient deterioration not necessarily related to observations	• Routine opportunities for more experienced staff to coach those less experienced (clinical supervision)<br>• Skills training: working as a team<br>• Track and Trigger Tool: staff concern a core parameter	• Less experienced staff learn pattern recognition from more experienced staff<br>• Staff respect and value each other's opinion<br>• Staff recognise concern as a valid indicator of deterioration<br>• Staff concern included as a key parameter in the detection of deterioration

For peer review only

DETECTION	Detection of deterioration depends on supporting and enabling families to detect other signs of deterioration and express concerns	At a minimum this requires:<br>• Staff understand the value of family concerns in the detection of deterioration<br>• Families are in a position to discern patterns of signs and symptoms in their child<br>• Families are encouraged and supported in raising concerns<br>• Parents can voice their concerns	At a minimum this requires:<br>• Families are supported to stay with their children<br>• Families are involved with defining normal parameters for their child and tasked with identifying changes Parents are supported to articulate their concerns<br>• Staff keep families informed about developments in care and treatment<br>• Staff receive guidance about how to respond to family concern	• Parent information leaflets and posters<br>• Skills training: family engagement<br>• "Planning care together" forms<br>• "What to expect" script<br>• Track and Trigger Tool: parent concern a core parameter	• Parents are informed that their views matter, and provided guidance about how to share their concerns with staff<br>• Staff respect and value family engagement<br>• Staff recognise family concern as a valid indicator of deterioration<br>• Parents and staff can share, discuss and document parental concerns.<br>• Parents receive guidance about how to articulate their concerns
	Proposition	Conceptual requirements	Operationalisation	Interventions	Proposed effect of

For peer review only - http://bmjopen.bmj.com/site/about/guidelines.xhtml

					interventions
PREPARATION	Preparation depends on collating and sharing indicators of deterioration for each patient.	At a minimum this requires:<br>• Information from children, staff and families is integrated<br>• Staff are in a position to review and interpret information from children, families and staff Identifying children that need to be watched	At a minimum this requires:<br>• Information from children, staff and families is interpreted together<br>• Regular opportunities for communication between relevant team members	• Skills training: working as a team<br>• “Watcher”<br>• Common information space<br>• Structured and standardised handover<br>• Huddles	• Staff develop the skills and behaviours required for effective communication, coordination and collaboration and understand their purpose and importance.<br>• A formalised process where the bedside nurse, and clinician, proactively identify risk, which includes assessment of information from children, families and staff<br>• Information on deterioration from

For peer review only

					children, staff and families are brought together, displayed together, regularly updated and easily accessible<br>• Key information from children, staff and families is systematically shared in a structured method, during handovers, between shifts or between staff in the same or different clinical areas.<br>• Senior staff allocated responsibility for knowing which patients are at high risk of deterioration meet with those responsible for

For peer review only

For peer review only - http://bmjopen.bmj.com/site/about/guidelines.xhtml

					conducting observations and liaise with families to discuss plans for individual patient management

For peer review only

For peer review only - http://bmjopen.bmj.com/site/about/guidelines.xhtml

P R E P A R A T I O N	Preparation depends on staff being aware at ward level of the status of individual patients and the availability of skills and resources and being able to plan appropriate response.	At a minimum this requires:<br>• There is a regular mechanism for reviewing the status of all children in the ward<br>• The is a regular mechanism for reviewing staffing levels and skills mix, workload, acuity and admissions<br>• There is a regular mechanism for communicating the review of all children, staffing levels and other resources to the rest of the team<br>• There is a regular mechanism for planning appropriate response<br>• Staff are aware of at-risk children and response plans<br>• Staff have situational awareness appropriate to their seniority (e.g.	At a minimum this requires:<br>• Information from children, staff and families is brought together<br>• Information from individual patient assessments are brought together and kept up to date<br>• Senior staff members are allocated responsibility for assessing staff levels and resources<br>• Senior staff members are allocated responsibility for managing demand and resources<br>• Senior staff members are allocated responsibility for communicating response plans	• Skills training: working as a team<br>• Common information space<br>• Structured and standardised handover<br>• Huddles<br>• Watch-stander	• Staff develop the skills and behaviours required for effective communication, coordination and collaboration and understand their purpose and importance.<br>• Information on deterioration (physiological, staff concern, family concern) are brought together, displayed together, regularly updated and easily accessible<br>• Key information about patients, staffing levels and other resources is systematically recorded and shared<br>• Senior staff allocated responsibility for knowing which patients are at high risk of

For peer review only

For peer review only - http://bmjopen.bmj.com/site/about/guidelines.xhtml

	Proposition	Conceptual requirements	Operationalisation	Interventions	Proposed effect of interventions
ACTION	Escalation depends on effective communication across professional, disciplinary and hierarchical boundaries.	At a minimum this requires:<br>• A trigger or prompt to act<br>• Clearly defined graded escalation and response procedures – agreed at organisational level<br>• Staff aware of roles and responsibilities<br>• Staff understand their part in the escalation procedure as activators and responders	At a minimum this requires:<br>• Staff receive guidance about graded escalation and response procedures<br>• Staff receive guidance on which members of staff are responsible for activating and responding<br>• Staff receive guidance about how to escalate	• Activation policy<br>• Activation criteria displayed throughout hospital<br>• Staff induction<br>• Track and Trigger Tool	• All staff and parents are aware of escalation and response criteria<br>• All staff and parents are aware of escalation and response procedures and roles and responsibilities<br>• Alerts staff to signs of deterioration and prompts the appropriate response
ACTION	Escalation depends on effective communication across professional, disciplinary and hierarchical boundaries	At a minimum this requires:<br>• Staff understand the significance of information<br>• Staff able to communicate information	At a minimum this requires:<br>• A structured approach to sharing information<br>• Staff concerns are positively received and acted upon, irrespective of	• Activation criteria displayed throughout hospital<br>• Staff induction<br>• Track and trigger tool<br>• SBAR	• All staff and parents are aware of escalation and response criteria<br>• All staff and parents are aware of escalation and response procedures

For peer review only - http://bmjopen.bmj.com/site/about/guidelines.xhtml

		across professional hierarchies<br>• All staff are able to express concerns	the outcome		and roles and responsibilities<br>• Tool packages legitimises, empowers and informs individuals when attempting to signal deterioration<br>• Time critical information about patients, staffing levels and other resources that requires immediate attention and action is shared in a structured method

For peer review only

For peer review only - http://bmjopen.bmj.com/site/about/guidelines.xhtml

TABLE 2 SCREENING BREAKDOWN

	PEWS	EWS	Structured handover	Situational awareness		Electronic systems	Observations & monitoring	Family involvement	Snowball sample
Database searching	3564	1155	3369	302		/	/	/	/
Additional sources	83	7	150	46		/	/	/	/
Records after duplicates removed	2194	751	2156	199		/	/	/	/
Hand searches	431	/	/	/		26	20	15	5
Title screening	90	751	2156	199		26	20	15	5
Abstract screening	62	106	n/a	n/a		26	20	15	5
Full paper screening	39	65	37	26		26	20	15	5
**Included in Syntheses**	**24**	**22**	**4**	**6**		**10**	**2**	**9**	**5**

For peer review only

TABLE 3 SUMMARY OF DETECTION EVIDENCE

Author	Country	Methodology	Analysis	Search Area	Evidence Contribution
Andrews, T. and Waterman, H. (2005) Packaging: a grounded theory of how to report physiological deterioration effectively. *Journal of Advanced Nursing* 52, 5, 473–481	U.K.	Interviews and observations	Grounded theory	EWS	Importance of 'gut feeling' in detecting deterioration<br><br>Vital sign monitoring delegated to junior staff
Astroth, K., Woith, W. M., Stapleton, S. J., Degitz, R. J., & Jenkins, S. H. (2013). Qualitative exploration of nurses' decisions to activate rapid response teams. Journal of Clinical Nursing, 22(19-20), 2876-2882	U.S.A.	Semi-structured interviews with nurses	Coding categories were generated from the data, and consensus on final themes was achieved through an iterative process	EWS	Staff encouraged to use their intuition when activating the RRT

For peer review only - http://bmjopen.bmj.com/site/about/guidelines.xhtml

Author	Country	Methodology	Analysis	Search Area	Evidence Contribution
Azzopardi, P.; Kinney, S.; Moulden, A. & Tibbals, J. Attitudes and barriers to a Medical Emergency Team system at a tertiary paediatric hospital. *Resuscitation*, 82, 167-174	Australia	Survey	Statistical analysis	PEWS	Track & Trigger Tools used to confirm or identify deterioration depending on experience. Useful for junior staff<br><br>Vital sign monitoring delegated to junior staff
Bellomo, R., Ackerman, M., Bailey, M., Beale, R. Clancy, G., Danesh, V. et al . (2012) The Vital Signs to Identify, Target, and Assess Level of Care Study (VITAL Care Study) Investigators.. Crit Care Med Vol. 40, No. 8; 2349–2361	International: U.S.A; Sweden; U.K; Netherlands; Australia	Before-and-after multi-centred international controlled trial.	Automated vital signs monitoring and early warning score (EWS) calculated, international study, blinded trial, hospitals retained own EWS and escalation policy	Electronic systems	The electronic health record (HER) provides a prompt to action
Bonafide C., Roberts K., Weirich C., Paciotti B., Tibbetts K., Keren R.,	U.S.A.	Semi-structured interviews	Grounded theory	PEWS	Vital sign monitoring delegated to junior workers who may not have the knowledge to interpret results. Track & Trigger Tools used to confirm or identify deterioration depending on experience & particularly useful for junior

For peer review only - http://bmjopen.bmj.com/site/about/guidelines.xhtml

Author	Country	Methodology	Analysis	Search Area	Evidence Contribution
Barg F. and Holmes J. (2013) Beyond Statistical Prediction: Qualitative Evaluation of the Mechanisms by which Pediatric Early Warning Scores Impact Patient safety. *J Hospital Medicine*, 8, 5, 248-253					staff<br><br>Professional intuition important for senior staff to detect deterioration
Bonafide, C. P., et al. (2014). Video Methods for Evaluating Physiologic Monitor Alarms and Alarm Responses. Biomédical Instrumentation & Technology May/June 2014, p220-230	U.S.A.	Prospective Feasibility study	Video recording and electronic patient data collected prospectively. Pragmatic observational study of VitalPAC deployment in 2 large hospitals	Electronic systems	Alarm fatigue a barrier to escalation
Bonafide, C.P. et al. (2017) Video Analysis of Factors Associated With Response Time to Physiologic Monitor	U.S.A.	Video review and response time outcome	Statistical analysis	PEWS	Alarm fatigue a barrier to escalation

For peer review only

Author	Country	Methodology	Analysis	Search Area	Evidence Contribution
Alarms in a Children's Hospital. *JAMA Pediatrics*, 171, 6, 524-531					
Braaten, J.S. (2015). Hospital System Barriers to Rapid Response Team Activation: A Cognitive Work Analysis. *American Journal of Nursing*, 115(2), 22-32.	U.S.A.	Document review and interviews using the principles of cognitive work analysis	Inductive and deductive forms of analysis – cognitive work analysis, framework and directed content analysis	EWS	Vital sign monitoring delegated to junior workers who may not have the knowledge to interpret results.<br><br>Track and Trigger Tools used to confirm or identify deterioration depending on experience; Professional intuition important for more senior staff/when clinical change is abrupt<br>Issues around availability of equipment and staffing
Brady P.W. and Goldenhar L.M. (2013a). A qualitative study examining the influences on situation awareness and the identification, mitigation and escalation of recognised patient risk. *BMJ Quality and Safetyl,* 0, 1-9.	U.S.A.	Focus groups x7 – held in groups of participants with similar roles	Constant comparison	Situational awareness	Paediatric early warning score (PEWS) supplementing gut feeling but these were not standardised
Burns et al. (2018)	U.S.A.	Semi-structured	Iterative thematic	Snowball	Importance of professional intuition is reported

For peer review only - http://bmjopen.bmj.com/site/about/guidelines.xhtml

Author	Country	Methodology	Analysis	Search Area	Evidence Contribution
Enhanced early warning system impact on nursing practice: A phenomenological study. *Journal of Advanced Nursing*, 1-7.		interviews were used drawing on a descriptive phenomenologica l methodology	analysis	sample	
Chua, W. L., Mackey, S., Ng, E. K. C., & Liaw, S. Y. (2013). Front line nurses' experiences with deteriorating ward patients: a qualitative study. *International Nursing Review*, 60, 4., 501-509.	Singapor e	A qualitative survey using critical incident technique	Inductive content analysis	EWS	Vital sign monitoring is the responsibility of nurses. The regularity of this activity can lead to it being viewed as a mundane activity<br><br>Importance of 'gut feeling' and intuition in detection of deterioration
Cioffi, J. (2000). Nurses' experiences of making decisions to call emergency assistance to their patients. Journal of Advanced Nursing, 32(1), 108-114.	Australia	Unstructured interviews with nurses who had activated the medical emergency team (MET)	Simple code and retrieve	EWS	Importance of gut feeling and 'knowing' your patient in the detection of deterioration<br><br>Importance of having staff concern in the MET criterion
Cioffi, J. (2000). Recognition of patients	Australia	Unstructured interviews	Simple code and retrieve	EWS	Importance of gut feeling and intuition in recognising deterioration

For peer review only - http://bmjopen.bmj.com/site/about/guidelines.xhtml

Author	Country	Methodology	Analysis	Search Area	Evidence Contribution
who require emergency assistance: a descriptive study. Heart & Lung, 29(4), 262-268.					Importance of having staff concern in a calling criteria
Cioffi, J., Salter, C., Wilkes, L., Vonu-Boriceanu, O., & Scott, J. (2006). Clinicians' responses to abnormal vital signs in an emergency department. Australian Critical Care, 19(2), 66-72.	Australia	Focus groups with clinicians and nurses exploring their responses to abnormal vital signs	Constant comparison	EWS	Intuition important, particularly for more senior staff to detect AVS. Knowing your patient reported as key; knowledge and experience seen as essential.
Claussen, D., Garner, D. and Crow, B. (2013) Early warning signs and the EHR: At the intersection of technology and care. *Nursing Management*, 44, 11, 14-16	U.S.A.	Retrospective review of calls to the rapid response team (RRT) and cardiac arrest calls to evaluate impact of evidence based guidelines (pre-intervention).	Descriptive statistics	Electronic systems	Professional intuition used in conjunction with MEWS

For peer review only - http://bmjopen.bmj.com/site/about/guidelines.xhtml

Author	Country	Methodology	Analysis	Search Area	Evidence Contribution
		EWS and huddles implemented. EHR available throughout to compare pre and post			
Davies, O., DeVita, M.A., Ayinla, A. and Perez, X.. (2014). Barriers to activation of the rapid response system. *Resuscitation*, 85(11), 1557-1561.	U.S.A.	Survey looking at barriers to rapid response system (RRS) activation	Statistical analysis	EWS	Professional intuition used over RRS activation criteria – barrier to activation
de Groot, J F. et al (2018) “Implementing Paediatric Early Warning Scores Systems in the Netherlands : Future Implications.. *BMC Pediatrics,* 18: 128	Netherlan ds	Retrospective patient review & semi structured interviews with professionals	Descriptive statistics and grounded theory	PEWS	Importance of professional intuition is reported
Donohue, L. and Endacott, R. (2010) Track, trigger and	U.K.	Qualitative design with critical incident	Thematic analysis	EWS	Importance of gut feeling and intuition in detecting deterioration

For peer review only

Author	Country	Methodology	Analysis	Search Area	Evidence Contribution
teamwork: Communication of deterioration in acute medical and surgical wards. *Intensive and Critical Care Nursing*, 26, 10-17		technique. Semi-structured interviews with nurses and the outreach team			
Downey, C.L., Tahir, W., Randell, R., Brown, J.M. and Jayne, D.G. (2017) Strengths and limitations of early warning scores: A systematic review and narrative synthesis. *International Journal of Nursing Studies*, 76, 106- 119	U.K.	Narrative review	"Patterns were identified and translated to themes, which were further refined using an iterative process".	PEWS	Drive towards automated alerts
Endacott et al (2007). Recognition and communication of patient deterioration in a regional hospital: A multi-methods study. Australian Critical care	U.K.	Mixed methods case study – semi structured interviews and audit of charts	Qualitative content analysis & descriptive statistics	Obs and monitoring	Gut feeling important – interestingly particularly for nurses whereas doctors sought additional empirical evidence to back up intuition

For peer review only - http://bmjopen.bmj.com/site/about/guidelines.xhtml

Author	Country	Methodology	Analysis	Search Area	Evidence Contribution
20, 100—105					
Endacott, R., & Westley, M. (2006). Managing patients at risk of deterioration in rural hospitals: a qualitative study. Australian Journal of Rural Health, 14(6), 275-279	Australia	Questionnaire, in-depth interviews and observations	Content analysis and constant comparison	EWS	Intuition and anticipatory skills important in detecting deterioration
Entwistle, V. (2004). Nursing shortages and patient safety problems in hospital care: is clinical monitoring by families part of the solution? Health Expectations, 7, pp.1–5 (editorial)	U.S.A.	Editorial	N/a	Family involvement	Little evidence/no evaluations of policies or practices that encourage and support family involvement in clinical monitoring<br><br>Propose the innovative practice of interdisciplinary rounds where families are invited, and communication is directed to the patient and family
Fagan, K.,Sabel, A., Mehler, P. S. and MacKenzie, T. D. (2012). Vital Sign Abnormalities, Rapid Response, and Adverse Outcomes in	U.S.A.	Observational Cohort comparison study	Descriptive statistics	Electronic systems	Concerns about over-burdening staff with automated alerts.<br><br>When considering the patient's baseline, nursing staff are expected to notify the patient's care provider immediately responsible for the patient when the vital signs meet or exceed the clinical trigger

For peer review only

Author	Country	Methodology	Analysis	Search Area	Evidence Contribution
Hospitalized Patients. American Journal of Medical Quality 27(6) 480– 486					
Graedon, J. and Graedon, T. (2006) Enlisting Families as Patient Safety Allies. Clinical Paediatric *Emergency Medicine*, 265-267	U.S.A focus	Opinion piece	N/a	Family involvement	Parents to explain child's baseline<br><br>Need to have detailed information about signs and symptoms that they should look for that would require alerting the medical team<br><br>Propose the need for structured inclusion of family concern during ward rounds (safety walkrounds)
Hands, C. et al. (2013) Patterns in the recording of vital signs and early warning scores: compliance with a clinical escalation protocol. *Quality and Safety in Health Care*, 1-8.	U.K.	The vital signs (V.S.) and early warning data for all in-patients for one year to investigate patterns of V.S observations collected	Statistical	Obs and Monitoring	Staffing levels and availability of equipment impede the collection of vital signs and early warning data.<br><br>Night-time factors also play a role with a decrease in observations noted
Hope et al. (2017) A fundamental conflict of care: Nurses' accounts	U.K.	Semi-structured interviews with 17 registered nurses	Constant comparative method informed	Snowball sample	Wider context of night-time care considered; there is some indication that staff are making a choice and prioritising sleep over monitoring

For peer review only

Author	Country	Methodology	Analysis	Search Area	Evidence Contribution
of balancing patients' sleep with taking vital sign observations at night. *Journal of Clinical Medicine*			by grounded theory		
James, J., Butler-Williams, C., Hunt, J. and Cox, H. (2010) Vital signs for vital people: an exploratory study into the role of the Healthcare Assistant in recognising, recording and responding to the acutely ill patient in the general ward setting. *Journal of Nursing Management*, 18, 548-555	U.K.	Postal survey with Healthcare Assistants (HCAs) utilising closed and open questions	Descriptive statistics & content analysis of qualitative data	Observations and monitoring	Factors other than the score used to detect deterioration - intuition/patient's own descriptions<br><br>Vital sign monitoring delegated to junior staff who may not have the skills to interpret results; many HCAs did not "fully understand this neurological assessment and it is implications for the acutely unwell patient thus resulting in the risk of an inaccurate EWS score" p 552.
Jensen, C. et al. (2018) Pediatric Early Warning Score Systems, Nurses Perspective – A Focus Group Study. *Journal of Pediatric Nursing*	Denmark	Focus group exploring nurse' experiences with PEWS	Qualitative meaning condensation analysis	PEWS	Sleeping as a part of care identified<br><br>Professional intuition<br><br>Night-time pressures

For peer review only - http://bmjopen.bmj.com/site/about/guidelines.xhtml

Author	Country	Methodology	Analysis	Search Area	Evidence Contribution
Jones, D. et al. (2006). Nurses' attitudes to a medical emergency team service in a teaching hospital. Quality & Safety in Health Care, 15(6), 427-432.	Australia	Questionnaire about understandings and barriers to activating a medical emergency team (MET)	Simple descriptive statistics	EWS	Intuition important when detecting deterioration
Jones, S., Mullally, M., Ingleby, S., Buist, M., Bailey, M. and Eddleston, J. M. (2011). Bedside electronic capture of clinical observations and automated clinical alerts to improve compliance with an Early Warning Score protocol. Crit Care Resusc; 13: 83–88	U.K.	Electronic capture of physiological data to see if automated clinical alerts increase compliance with an early warning score (EWS) and improve patient outcomes	Statistical	Electronic systems	Electronic systems can provide prompts or alerts for monitoring vital signs<br><br>This system demonstrated significant improvement in clinician attendance to acutely ill patients with an early warning score of 3 and above
Kaul, M., Snethen, J., Kelber, S., Zimmanck, K., Maletta, K. and	U.S.A.	Descriptive cross sectional study; nurse and	Descriptive statistics	PEWS	Benefits of BedsidePEWS – nurses more likely to identify early signs of deterioration and a greater ability to escalate care. Delegation to junior staff justified.

For peer review only - http://bmjopen.bmj.com/site/about/guidelines.xhtml

Author	Country	Methodology	Analysis	Search Area	Evidence Contribution
Meyer, M. (2014) Implementation of the bedside paediatric early warning system (BedsidePEWS) for nurse identification of deteriorating patients'. *Journal for specialists in pediatirc nursing*, 19, 339-349		medical staff survey			
Lobos AT, Fernandes R, Ramsay T, McNally JD. (2014) Patient characteristics and disposition after pediatric medical emergency team (MET) activation: disposition depends on who activates the team. Hospital Pediatrics. 4(2):99-105	Canada	Implementation report	Simple descriptive statistics	PEWS	Delegation to junior staff<br><br>Education package developed around the RRS with a variety of informal lectures and interactive sessions; "lunch and learns", "education coffee carts" and simulation programs for instance.
Lydon et al. (2016) A mixed-methods investigation of health		30 semi-structured interviews based	Deductive content analysis	PEWS	Professional intuition

For peer review only

Author	Country	Methodology	Analysis	Search Area	Evidence Contribution
professionals' perceptions of a physiological track and trigger system. *BMJ Quality and Safety*, 25, 688-695		on the theory of planned behaviour were conducted.<br><br>Questionnaire with Likert scale developed from the interview data			
Mackintosh, N., Humphrey, C., & Sandall, J. (2014a). The habitus of 'rescue' and its significance for implementation of rapid response systems in acute health care. Social Science & Medicine, 120, 233-242	U.K.	Ethnographic perspective ; observations, semi-structured interviews	Data were inductively and deductively coded using NVivo v8 and organised thematically.	EWS	Delegation of routine observation and vital sign monitoring to junior staff<br><br>Professional intuition important; 'knowing the patient' important for detecting subtle changes in a patient's condition
Mackintosh, N., Rainey, H. and Sandall, J. (2012a) Understanding how rapid response systems may improve	U.K.	Comparative case study a rapid response system (RRS) using	Inductive and deductive coding facilitated by NVivo. Also utilised theme	EWS	Observations and monitoring delegated to junior staff/health care assistants (HCAs) and nurses – early warning system (EWS) legitimised delegation of these tasks.<br>Importance of pattern recognition and intuition

For peer review only - http://bmjopen.bmj.com/site/about/guidelines.xhtml

Author	Country	Methodology	Analysis	Search Area	Evidence Contribution
safety for the acutely ill patient: learning from the frontline. *BMJ Qual Saf*, 21, 135-144		ethnographic methods including observations, interviews and documentary review.	building and structuring methods from framework analysis whilst also informed by other theoretical frameworks such as 'technology-in-practice'.		The TTT used to confirm or identify deterioration and are particularly useful for junior staff.
Mackintosh, N., Watson, K., Rance, S., & Sandall, J. (2014b). Value of a modified early obstetric warning system (MEOWS) in managing maternal complications in the peripartum period: an ethnographic study. BMJ Quality & Safety, 23(1), 26-34	U.K.	Ethnographic study using observations (>120 h), semi-structured interviews (n=45) and documentary review	Thematic analysis with data analysed iteratively in addition to a more strategic and policy-focused coding framework	EWS	Professional intuition important when detecting deterioration
Massey, D., Chaboyer, W., & Aitken, L. (2014). Nurses' perceptions of	Australia	In depth semi structured interviews	Inductive approach – thematic analysis	EWS	Importance of intuition or 'sensing clinical deterioration'

For peer review only - http://bmjopen.bmj.com/site/about/guidelines.xhtml

Author	Country	Methodology	Analysis	Search Area	Evidence Contribution
accessing a Medical Emergency Team: a qualitative study. Australian Critical Care, 27(3), 133-138					
McDonnell, A., Tod, A., Bray, K., Bainbridge, D., Adsetts, D., & Walters, S. (2013). A before and after study assessing the impact of a new model for recognizing and responding to early signs of deterioration in an acute hospital. *Journal of Advanced Nursing,* 69(1), 41-52.	U.K.	Single centre, mixed method before and after study including a survey to measure changes in nurses' knowledge after implementation of a track and trigger system (T&Ts). Also, qualitative interviews	Statistical analysis and thematic framework analysis	EWS	T&Ts used to confirm or identify deterioration depending on the experience of the user. Specific objective information was seen as helping the response arm prioritise work<br><br>Importance of professional intuition or 'gut feeling' in detecting deterioration<br><br>Need for staff concern in T&Ts
McKay, H., Mitchell, I., Sinn, K., Mugridge, H., Lafferty, T., Leuvan,, C. Mamootil, S. and Abdel-Latif, M. (2013) "Effect of	Australia	Prospective, controlled before and after intervention trial.	Statistical analysis	PEWS	Specific education package developed around the Paediatric Early Warning Score (PEWS) which assists in the recognition of deterioration

For peer review only - http://bmjopen.bmj.com/site/about/guidelines.xhtml

Author	Country	Methodology	Analysis	Search Area	Evidence Contribution
a multifaceted intervention on documentation of vital signs and staff communication regarding deteriorating paediatric patients", *Journal of Paediatrics and Child Health,* Volume 49, Issue 1, pages 48–56					
Mohammed, Hayton, R., Clements, G., Smith, G. and Prytherch, D. (2009) Improving accuracy and efficiency of early warning scores in acute care. *British Journal of Nursing*, 18, 1, 18-24	U.K.	Three phases; the first two were based in a classroom and asked nurses to calculate an early warning score (EWS) from vignettes using pen and paper followed by a hand-held computer. The	Statistical	Electronic systems	Timeliness of electronic vital sign recording when compared with paper systems

For peer review only

Author	Country	Methodology	Analysis	Search Area	Evidence Contribution
		third phase followed the previous approach but was based on the ward after nurses had been using the device for 4 weeks			
Mohammed Iddrisu, S. et al (2018) Nurses role in recognising and responding to clinical deterioration. *Journal of Clinical Nursing,* 27, 9-10, 1920-1930	Australia	To explore nurse' role in recognising and responding to deteriorating postoperative patients through focus groups.	Thematic analysis	Snowball sample	Staff concern in tool criteria useful
Paciotti B, Roberts KE, Tibbets KM, Weirich Paine C, Keren R, Barg FK, Holmes J H and Bonafide CP (2014). Physician attitudes toward family-activated	U.S.A.	Semi-structured interviews with clinicians	Grounded theory and constant comparison	Family involvement	Physicians depend on families to explain a child's base-line

For peer review only - http://bmjopen.bmj.com/site/about/guidelines.xhtml

Author	Country	Methodology	Analysis	Search Area	Evidence Contribution
medical emergency teams for hospitalized children. The Joint Commission Journal on Quality and Patient Safety, 40 (4)					
Pattison, N., & Eastham, E. (2012). Critical care outreach referrals: a mixed-method investigative study of outcomes and experiences. *Nursing in Critical Care,* 17(2), 71-82.	U.K.	Mixed method study looking at the impact of a critical care outreach team (CCOT)	Statistical analysis and grounded theory	EWS	Track and trigger tool is used to either confirm or identify deterioration depending on the experience of the user<br><br>'Gut feeling'/intuition important – patient appearance often an important sign in detecting deterioration
Radeschi, G., Urso, F., Campagna, S., Berchialla, P., Borga, S., Mina, A., . . . Sandroni, C. (2015). Factors affecting attitudes and barriers to a medical emergency team among nurses and medical	Italy	Multicentre survey to identify the attitudes and barriers to medical emergency team (MET) utilisation	Statistical analysis	EWS	Intuition (physical appearance important) in detecting deterioration

For peer review only - http://bmjopen.bmj.com/site/about/guidelines.xhtml

Author	Country	Methodology	Analysis	Search Area	Evidence Contribution
doctors: A multi-centre survey. *Resuscitation*, 88, 92-98.					
Schmidt, P. E., et al (2014). Impact of introducing an electronic physiological surveillance system on hospital mortality. BMJ Quality and safety.	U.K.	Retrospective analysis of data collected routinely. Pragmatic observational study of VitalPAC deployment in 2 large hospitals	Statistical analyses	Electronic systems	Electronic systems provide prompts for alerts facilitates better recognition of deterioration and is associated with reduced mortality.
Sefton, G., Lane, S., Killen, R. et al. (2017) Accuracy and Efficiency of Recording Pediatric Early Warning Scores Using an Electronic Physiological Surveillance System Compared With Traditional Paper-Based	U.K.	Controlled study of vital sign documentation & PEWS calculation and a survey of acceptability	Descriptive statistics	PEWS	Errors in paper-based documentation were found; incorrect age-specific chart used; inaccurate documentation of values and plotting of trends; incorrect PEWS calculation. In comparison the error rate using the electronic PEWS was low

For peer review only - http://bmjopen.bmj.com/site/about/guidelines.xhtml

Author	Country	Methodology	Analysis	Search Area	Evidence Contribution
Documentation. *Computers, Informatics, Nursing,* 35, 5, 228 – 236					
Shearer, B., Marshall, S., Buist, M. D., Finnigan, M., Kitto, S., . . . Ramsay, W. (2012). What stops hospital clinical staff from following protocols? An analysis of the incidence and factors behind the failure of bedside clinical staff to activate the rapid response system in a multi-campus Australian metropolitan healthcare service. BMJ Quality & Safety, 21(7), 569-575	Australia	A mixed-method study	Iterative coding	EWS	Importance of professional intuition
Sonning, K, et al. (2018) "A Survey of Healthcare Professionals' Experiences with the Paediatric Early Warning	Norway	Questionnaire of a sample of staff who use PEWS	Descriptive statistics	PEWS	Appreciate that PEWS encourages a systematic approach to monitoring.

For peer review only

Author	Country	Methodology	Analysis	Search Area	Evidence Contribution
Score (PEWS).” *Norwegian Journal of Clinical Nursing* 12(64605).					
Stevenson, J. E. & Nilsson G. (2011). Nurses’ perceptions of an electronic patient record from a patient safety perspective: a qualitative study. Journal of Advanced Nursing 68(3), 667–676	Sweden	Qualitative; focus groups with 21 registered nurses	Content analysis of interviews	Electronic systems	Verbal reports were favoured over the electronic system
Stewart, J., Carman, M., Spegman, A., & Sabol, V. (2014). Evaluation of the effect of the modified early warning system on the nurse-led activation of the rapid response system. *Journal of Nursing Care Quality*, 29(3), 223-229.	Sweden	Mixed method. Retrospective review of records and nurse led focus groups	Statistical analysis and content analysis	EWS	Intuition still plays a part – the ‘score’ was rarely the single determining factor in activation but rather prompted nurses to gather additional information
Subbe et al. (2018)	U.K.	A before and	Statistical analysis	Snowball	Automated VS monitoring associated with a decrease in mortality

For peer review only - http://bmjopen.bmj.com/site/about/guidelines.xhtml

Author	Country	Methodology	Analysis	Search Area	Evidence Contribution
Effect of an automated notification system for deteriorating ward patients on clinical outcomes. *Critical Care*, 21, 52		after study of an electronic automated advisory V.S. monitoring and notification system. Elevated scores were relayed to a RRT.		sample	
Wager, K. A., Schaffner, M. J., Foulois, B., Swanson Kazley, A., Parker, C. Walo, H. (2010). Comparison of the Quality and Timeliness of Vital Signs Data Using Three Different Data-Entry Devices. Computers, Informatics, Nursing & Vol. 28, No. 4, 205–212	U.S.A.	Observational study.	Descriptive statistics	Electronic systems	Batching of patient data whereby the care provider handwrites the patient's vital signs and uploads it to the computer at a later time is common, especially as the computers are often busy. Individual tablet PCs seems to eliminate this from occurring.
Watson, A., Skipper, C., Steury, R., Walsh, H. and Levin, A. (2014)	U.S.A.	Mixed method, retrospective medical record	Observation analysis, although this is not	PEWS	Information relating to transfer to electronic systems – distance the 'eyes' of the nurses from the patient; batching.

For peer review only

Author	Country	Methodology	Analysis	Search Area	Evidence Contribution
Inpatient nursing care and early warning scores: a workflow mismatch. *J Nurs Care Qual*, 29, 3, 215-222		observations and observations of nurse interactions in one minute blocks.	described, and statistical analysis		Intuition
Wheatley (2006). The nursing practice of taking level 1 patient observations. I&CCNurs 22: 115-121	U.K.	Ethnographic approach; participant observation & semi-structured interviews	Thematic & content analysis	Observations and monitoring	The regularity of vital signs monitoring may also lead to it becoming viewed as mundane practice that can be delegated to health care assistants.<br><br>Workplace pressure/equipment failures affect quality of observations

For peer review only

For peer review only - http://bmjopen.bmj.com/site/about/guidelines.xhtml

TABLE 4 SUMMARY OF PREPARATION EVIDENCE

Author	Country	Methodology	Analysis	Search Area	Evidence Contribution
Abraham J, Kannampallil T, Patel B, Almoosa K, Patel V. (2012) Ensuring patient safety in care transitions: an empirical evaluation of a Handoff *Intervention Tool. AMIA Annual Symposium Proceedings/AMIA Symposium*, 17-26	U.S.A.	Pre and post prospective study.	The quality and completeness of the handoff note – both tools – was assessed by a multi-professional round	Structured handover	Value of a checklist tool for handover
Brady P.W. and Goldenhar L.M. (2013a). A qualitative study examining the influences on situation awareness and the identification, mitigation and escalation of recognised patient risk.	U.S.A.	Focus groups x7 – held in groups of participants with similar roles	Constant comparison	Situational awareness	Huddle useful to proactively identify and plan for risk<br><br>Structure to support handover important

For peer review only - http://bmjopen.bmj.com/site/about/guidelines.xhtml

Author	Country	Methodology	Analysis	Search Area	Evidence Contribution
BMJ Quality and Safetyl, 0, 1-9.					
Brady P.W., Muething S., Kotagal et al. (2013b). Improving situation awareness to reduce unrecognized clinical deterioration and serious safety events. *Pediatrics*, 131, 1.	U.S.A.		Statistical process control charts	Situational awareness	Huddles led by a Watchstander charge nurse. When risk is identified the team discussed this and developed a plan to mitigate risk
Claussen, D., Garner, D. and Crow, B. (2013) Early warning signs and the EHR: At the intersection of technology and care. *Nursing Management*, 44, 11, 14-16	U.S.A.	Retrospective review of calls to the rapid response team (RRT) and cardiac arrest calls to evaluate impact of evidence-based guidelines	Descriptive statistics	Electronic systems	The huddle was seen as useful and was called as a patient's status was changing to 'red' so that all team members were informed
Davies, O., DeVita, M.A., Ayinla, A. and Perez, X.. (2014). Barriers to activation of the rapid response system.	U.S.A.	Survey looking at barriers to rapid response system (RRS) activation	Statistical analysis	EWS	Activation criteria displayed around the hospital

For peer review only - http://bmjopen.bmj.com/site/about/guidelines.xhtml

Author	Country	Methodology	Analysis	Search Area	Evidence Contribution
Resuscitation, 85(11), 1557-1561.					
Demmel KM, Williams L, Flesch L. (2010) Implementation of the Pediatric Early Warning Scoring System on a Pediatric Hematology/Oncology Unit. *Journal of Pediatric Oncology Nursing*, 27(4):229-240	U.S.A.	Discussion of the set-up and implementation of a paediatric early warning scoring tool and an associated algorithm	Rapid Plan-Do-Study-Act (PDSA) cycles were implemented using small tests of change. The data from the PDSA cycles were continuously collected, analysed, and reviewed with the multidisciplinary staff and planning team and used to give ongoing direction to the implementation plan.	PEWS	Importance of common information spaces & display of activation criteria throughout the hospital
Donahue M, Smith L, Dykes P. (2010) Phase 2 of the EMPOWER project: enhancing communication for paraprofessionals. *Journal of Continuing Education in Nursing*. 0;41(5).	U.S.A.	Focus groups evaluation of a training programme which was developed to teach paraprofessionals situation, background,	Not clear	Structured handover	Value of a structured approach to handover<br><br>Adaptation of the SBAR tool for handover

For peer review only - http://bmjopen.bmj.com/site/about/guidelines.xhtml

Author	Country	Methodology	Analysis	Search Area	Evidence Contribution
		assessment, recommendation (SBAR) communication tool			
Ennis, L (2014) Paediatric early warning scores on a children's ward: a quality improvement initiative. *Nursing children and young people*, 26, 7, 25-31	Ireland	Description of implementation of paediatric early warning score (PEWS) and subsequent audit (prospective cohort observational study)	Simple descriptive statistics of numbers children triggering PEWS and compliance with escalation protocol	PEWS	Common information spaces important & display of activation criteria throughout the hospital<br><br>Usefulness of ISBAR as a communication tool
Goldenhar LM, Brady PW, Sutcliffe KM, Muething SE (2013). Huddling for high reliability and situation awareness. *BMJ Quality & Safety*, 22, 899-906	U.S.A.	Semi structured interviews and focus groups to develop a deeper understanding of a newly implemented huddle systems	Constant comparison	Situational awareness	Importance of the huddle - Empowerment and sense of community; Facilitated greater and better information sharing<br><br>Each huddle participant was asked to systematically report on patients in their units who they thought would deteriorate in the near future and to label them as 'watchers', asking senior nurses and physician leads to coach charge nurses on how to integrate their perceptions into a informal severity of illness assessment (comprehension) and training the clinicians on how

For peer review only

Author	Country	Methodology	Analysis	Search Area	Evidence Contribution
					to use the information to facilitate prediction and planning for at risk patients (projection).
Mackintosh N, Berridge E-J, Freeth D. (2009) Supporting structures for team situation awareness and decision making: insights from four delivery suites. *Journal of Evaluation in Clinical Practice*.15(1):46-54.	U.K.	Ethnographic<br>2 stage process of lightly structured observations followed by a more focused period of data generation to test and elaborate the outcomes from stage 1	Initial thematic analysis and search for negative cases.	Situational awareness	For teams with a strong level of situation awareness (SA), key supports were all used in a balanced and supportive manner to gather and disseminate information which served to promote a culture of shared ownership and a proactive model of workload management, with temporary disruptions to the model easily accommodated.<br><br>Whiteboard – when used effectively was a vital means for supporting SA. It provides teams with a snapshot of a constantly changing workload, the team's activity levels and resource availability. Workload at times compromised its effectiveness. And irrespective of workloads, location and local traditions had a bearing on its use and usefulness.<br><br>White boards need to be in a visible location<br><br>Importantly, the interplay between these key support for SA will vary depending on the context; the same supports used differently will naturally produce different outcomes<br><br>Handover took many forms; structured and informal; profession

For peer review only

Author	Country	Methodology	Analysis	Search Area	Evidence Contribution
					specific and inter-professional; participatory or a one-way transmission of information. The effectiveness of SA was dependent upon the form that handover took – who was present, contributions made, information relayed. SA was more likely to be compromised when key people were absent.<br><br>Senior staff coordinator important for SA and became compromised if they were largely focussed on providing patient care.
Mackintosh, N., Rainey, H. and Sandall, J. (2012a) Understanding how rapid response systems may improve safety for the acutely ill patient: learning from the frontline. *BMJ Qual Saf*, 21, 135-144	U.K.	Comparative case study a rapid response system (RRS) using ethnographic methods including observations, interviews and documentary review.	Inductive and deductive coding facilitated by NVivo. Also utilised theme building and structuring methods from framework analysis whilst also informed by other theoretical frameworks such as 'technology-in-practice'.	EWS	Availability of equipment an issue where the track and trigger tool (TTT) was electronic<br><br>Electronic systems helped HCAs and nursing staff to share understandings, planning and manage their workload
Massey, D., Chaboyer, W., & Aitken, L. (2014). Nurses' perceptions of accessing a Medical Emergency Team: a	Australia	In depth semi structured interviews	Inductive approach – thematic analysis	EWS	Common information spaces useful. Display of activation criteria throughout hospital

For peer review only

Author	Country	Methodology	Analysis	Search Area	Evidence Contribution
qualitative study. Australian Critical Care, 27(3), 133-138					
McCrory et al. (2012) ABC-SBAR Training improves simulated critical patient hand-off by pediatric interns. *Pediatric Emergency Care*. 0;28(6).	U.S.A.	Prospective, pre-interventional and post-interventional study to evaluate the educational intervention of teaching ABC-SBAR	Two blinded reviewers assessed 52 video-recorded hand-offs for inclusion, order, and elapsed time to essential hand-off information using a scoring tool	Structured handover	Information sharing for handovers are of variable quality<br><br>A more structured approach will improve information sharing and therefore situational awareness – "without a structured hand-of tool, paediatric interns overemphasize background information and leave the reason for the call delayed".<br><br>Adaptation of the SBAR tool to include ABC – usefulness of this
Mullan PCM. A novel briefing checklist at shift handoff in an emergency department improves situational awareness and safety event identification. *Pediatric Emergency Care*. 2015;31(4):231-8.	U.S.A.	Descriptive observational study	Checklists were evaluated for rates of use, completion and identification of potential safety events	Situational awareness	The value of more structured approach to information sharing and situational awareness.<br><br>Utilises a checklist handover system for physicians. Checklist items focussed on the status of the patient, ED providers and hospital resources. A "Read-Do" format was designed.
Parker, M.W, Carroll, M., Bolser, B. et al. (2017) Implementation of a Communication Bundle	U.S.A.	Manual review of all eligible patient records	Descriptive statistics	PEWS	Example of a bundle around the "watcher" category with 5 component that needed to be completed within two hours of a patient being designated as such.

For peer review only - http://bmjopen.bmj.com/site/about/guidelines.xhtml

Author	Country	Methodology	Analysis	Search Area	Evidence Contribution
for High-Risk Patients. *Hospital Pediatrics,* 7, 9, 523-529					
Pearson G & Duncan H. (2011) Early warning systems for identifying sick children Paediatrics and Child Health. 21(5): 232-233	U.K.	Brief review of the evidence base surrounding PEWS	N/a	PEWS	Value of a more structured approach to communication - Advocate the use of a shared communication model such as SBAR (Situation, Background, Assessment and Recommendation) to communicate findings to superiors.
Pezzolesi C, Manser T, Schifano F, Kostrzewski A, Pickles J, Harriet N, et al. (2013) Human factors in clinical handover: Development and testing of a 'handover performance tool' for doctors' shift handovers. *International Journal for Quality in Health Care.* 25(1):58-65.	U.K.	Delphi Study for tool development	Descriptive statistics<br><br>Handovers were analysed and rated according to a measure of essential skills	Situational awareness	Information on handover – can be of variable quality<br><br>Most handover tools are adaptations of Situation Background Assessment Recommendation (SBAR) communication tool<br><br>Important to remember that handover facilitates teamwork and this hinges on communicative forms that extend beyond the information transfer that is typically the focus of structured handover tools
Randhawa S, Roberts-	U.S.A.	Description of the	Once a cycle from the	PEWS	White boards placed in a central location displayed scores of all

For peer review only - http://bmjopen.bmj.com/site/about/guidelines.xhtml

Author	Country	Methodology	Analysis	Search Area	Evidence Contribution
Turner R, Woronick K, DuVal J. (2011) Implementing and Sustaining Evidence-Based Nursing Practice to Reduce Pediatric Cardiopulmonary Arrest. Western Journal of Nursing Research. 33(3):443-456		implementation process with cardiopulmonary arrest (CPA) statistics pre and post implementation	implementation has been completed this is evaluated and then another cycle begins.		patients so that staff could quickly assess which patients were at high risk for deterioration.<br><br>Activation criteria displayed throughout the hospital
Stewart, J., Carman, M., Spegman, A., & Sabol, V. (2014). Evaluation of the effect of the modified early warning system on the nurse-led activation of the rapid response system. *Journal of Nursing Care Quality*, 29(3), 223-229.	Sweden	Mixed method. Retrospective review of records and nurse led focus groups	Statistical analysis and content analysis	EWS	Huddle –the score was used during the daily bed 'huddle' with the nursing administrators, to evaluate current unit acuity, determine staffing need & prepare for any transfers
Van Voorhis, K.T. and Scahde Willis, T. (2009) Implementing a pediatric rapid response system to	U.S.A.	Discussion paper highlighting the process of developing a	N/a	PEWS	Display of activation criteria throughout hospital on lanyards and use of white boards useful

For peer review only - http://bmjopen.bmj.com/site/about/guidelines.xhtml

Author	Country	Methodology	Analysis	Search Area	Evidence Contribution
improve quality and patient safety. *Pediatr Clin N Am*, 56, 919-933		paediatric rapid response system (RRS).			
Vries, A., Draaisma, J. and Fuijkschot, J. (2017) Clinician Perceptions of an Early Warning System on Patient Safety. *Hospital Pediatrics*, 7, 10, 579-586	Netherlands	Semi-structured interview	Qualitative content analysis	PEWS	PEWS/TTT is used to support situational awareness. Use of PEWS enables clinicians to have a 'birds-eye' view over admitted patients
Wager, K. A., Schaffner, M. J., Foulois, B., Swanson Kazley, A., Parker, C. Walo, H. (2010). Comparison of the Quality and Timeliness of Vital Signs Data Using Three Different Data-Entry Devices. Computers, Informatics, Nursing & Vol. 28, No. 4, 205–212	U.S.A.	Observational study.<br><br>Explored the timeliness and quality of vital signs data entered by 3 different recording methods	Descriptive statistics	Electronic systems	Batching of patient data whereby the care provider handwrites the patient's vital signs and uploads it to the computer at a later time is common, especially as the computers are often busy.

For peer review only - http://bmjopen.bmj.com/site/about/guidelines.xhtml

Author	Country	Methodology	Analysis	Search Area	Evidence Contribution
Watson, A., Skipper, C., Steury, R., Walsh, H. and Levin, A. (2014) Inpatient nursing care and early warning scores: a workflow mismatch. *J Nurs Care Qual*, 29, 3, 215-222	U.S.A.	Mixed method, retrospective medical record observations and observations of nurse interactions	Observation analysis, although this is not described, and statistical analysis	PEWS	Availability of equipment a factor
Weiss M.J.B., Bhanji, F., Fontela, PS. And Razack, SI.. (2013) A preliminary study of the impact of a handover cognitive aid on clinical reasoning and information transfer. Medical Education. 0;47(8)	Canada	A randomised controlled trial in an academic paediatric intensive care unit (PICU) of 20 handovers events	Differences between intervention and control groups were assessed using the Mann-Whitney test and multivariate linear regression.	Structured handover	Value of a more structured approach to support information sharing - a cognitive aid to facilitate handover that prompted residents to transmit this information. The handover aid was not linked into hospital information systems – so this had to be completed by hand before handover.<br><br>Handover is an opportunity for learning and professional socialisation
Wong, D., Bonnici, T., Knight, J., Morgan, L., Coombes, P. and Watkinson, P. (2015) SEND: a system for electronic notification and documentation of vital	U.K.	Description of user-focussed design process for use of electronic monitoring & numbers of observations	Descriptive statistics on the number of observations recorded using the SEND system & the number of active users	Electronic systems	Development of a flexible electronic system which enabled staff to have an overview of patients – reflections on disconnection

For peer review only - http://bmjopen.bmj.com/site/about/guidelines.xhtml

Author	Country	Methodology	Analysis	Search Area	Evidence Contribution
sign observations. *BMC Medical Informatics and Decision Making*, 15, 68.		taken using the system. Acceptability questionnaire			

For peer review only

For peer review only - http://bmjopen.bmj.com/site/about/guidelines.xhtml

TABLE 5 SUMMARY OF ACTION EVIDENCE

Author	Country	Methodology	Analysis	Search Area	Evidence Contribution
Adelstein, B. A., Piza, M. A., Nayyar, V., Mudaliar, Y., Klineberg, P. L., & Rubin, G. (2011). Rapid response systems: A prospective study of response times. Journal of Critical Care, 26, 635	Australia	Prospective comparison of rapid response team (RRT) criteria breaches	Statistical	EWS	Day/night differences in activation identified<br><br>Nurses may not understand what is required for activation.<br><br>Staff not investing in calling criteria
Almblad, A.C., Siltberg, P., Engvall, G. and Malqvist, M. (2018) Implementation of Pediatric Early Warning Score; Adherence toGuidelines and Influence of Context. *Journal of Pediatric Nursing,* 38, 33-39	Sweden	retrospective review of EPR and a context assessment of the work environment using the Alberta Context Tool.	Statistical	Snowball sample	Senior leadership consistently identified as important

For peer review only - http://bmjopen.bmj.com/site/about/guidelines.xhtml

Author	Country	Methodology	Analysis	Search Area	Evidence Contribution
Andrews, T. and Waterman, H. (2005) Packaging: a grounded theory of how to report physiological deterioration effectively. *Journal of Advanced Nursing* 52, 5,473–481	U.K.	Interviews and observations	Grounded theory	EWS	Track & Trigger Tools (TTT) act as prompts to action<br><br>TTT used to overcome challenges in communication & particularly valuable for junior staff<br><br>Negative attitude towards calling for help
Astroth, K., Woith, W. M., Stapleton, S. J., Degitz, R. J., & Jenkins, S. H. (2013). Qualitative exploration of nurses' decisions to activate rapid response teams. Journal of Clinical Nursing, 22(19-20), 2876-2882	U.S.A.	Semi-structured interviews with nurses	Coding categories were generated from the data, and consensus on final themes was achieved through an iterative process	EWS	Situation under control – no need to escalate or perceived business of medical staff discouraged staff from RRT activation<br><br>Staff encouraged to use their intuition when activating the RRT<br><br>Concern about feeling inadequate in front of colleagues a barrier to RRT activation<br>Inexperienced staff teaming up – led to staff trusting their own judgement<br><br>Traditional hierarchies a barrier to RRT activation – nurses more likely to call the attending physician rather than activate the RRT
Azzopardi, P.; Kinney, S.; Moulden, A. & Tibbals, J. Attitudes and barriers to a Medical Emergency Team	Australia	Survey	Statistical analysis	PEWS	Score rarely the determining factor in escalation - would not escalate for a patient who looked well but would escalate for a patient they were worried about even if not triggering<br><br>Negative attitude towards calling for help – feeling inadequate/perceived

For peer review only - http://bmjopen.bmj.com/site/about/guidelines.xhtml

Author	Country	Methodology	Analysis	Search Area	Evidence Contribution
system at a tertiary paediatric hospital. *Resuscitation*, 82, 167-174					business of Paediatric Intensive Care Unit had an impact on doctors escalating but not nurses<br><br>Senior leadership is important when implementing a MET
Bavare, A.C., Thomas, J.K., Elliott, E.P., Morgan, A.C. and Graf, J. (2017) Family-initiated pediatric rapid response: characteristics, impetus and outcomes, *Journal for healthcare Quality,* 40, 2, 103 – 109	U.S.A.	Retrospective observational study of rapid response events	Descriptive statistics	PEWS	All family activated RRT had appropriate clinical triggers with the most common being uncontrolled pain. More than half of FIRRs had a vital sign change that should have qualified Clinician-RRT activation. Seventy-six percent FIRRs needed at least one or more interventions. Twenty-seven percent of family initiated RRTs needed transfer to intensive care unit compared with 60% transfer rate for Clinician RRTs.
Bogert, S., Ferrell, C. and Rutledge, D. N. (2010). Experience with Family Activation Of Rapid Response Teams. MEDSURG Nursing—July/August 2010—Vol. 19/No. 4	U.S.A	Implementation of Condition Help (ConditionH)	Descriptive statistics	Family involvement	Implementation of ConditionH.<br><br>ConditionH being addressed during daily rounds
Bonafide C., Roberts K., Weirich C., Paciotti B., Tibbetts K., Keren R.,	U.S.A.	Semi-structured interviews	Grounded theory	PEWS	Disinclination to seek help and concerns about appearing inadequate in front of colleagues

For peer review only - http://bmjopen.bmj.com/site/about/guidelines.xhtml

Author	Country	Methodology	Analysis	Search Area	Evidence Contribution
Barg F. and Holmes J. (2013) Beyond Statistical Prediction: Qualitative Evaluation of the Mechanisms by which Pediatric Early Warning Scores Impact Patient safety. *J Hospital Medicine*, 8, 5, 248-253					Informal peer support<br><br>Senior leadership important
Braaten, J.S. (2015). Hospital System Barriers to Rapid Response Team Activation: A Cognitive Work Analysis. *American Journal of Nursing*, 115(2), 22-32.	U.S.A.	Document review and interviews using the principles of cognitive work analysis	Inductive and deductive forms of analysis – cognitive work analysis, framework and directed content analysis	EWS	Issues around availability of equipment and staffing<br><br>Negative attitude/delays around calling for help with staff needing to justify escalation. Other factors impact upon this including the perception that the situation is under control/perceived business of physicians/not wanting to appear inadequate
Brady P.W., Muething S., Kotagal et al. (2013b). Improving situation awareness to reduce unrecognized clinical deterioration and serious safety events.	U.S.A.		Statistical process control charts	Situational awareness	Concerns about resources reported

For peer review only - http://bmjopen.bmj.com/site/about/guidelines.xhtml

Author	Country	Methodology	Analysis	Search Area	Evidence Contribution
Pediatrics, 131, 1.					
Brady, P.W. et al. (2014) Developing and evaluating the success of a family activated medical emergency team: a quality improvement report. *BMJ Qual Saf* 2014; 0: 1–9.	U.S.A.	A retrospective cohort study looking at the association between family and clinician activations and transfer to the intensive care unit following a MET call	Quality improvement methods and statistical process control charts were used to assess the rate of family activation of METs	Family involvement	Direct mechanism for families to activate a MET<br><br>Concerns from clinicians about a family activated MET over-burdening the system are unfounded
Chua, W. L., Mackey, S., Ng, E. K. C., & Liaw, S. Y. (2013). Front line nurses' experiences with deteriorating ward patients: a qualitative study. *International Nursing Review*, 60, 4., 501-509.	Singapor e	A qualitative survey using critical incident technique	Inductive content analysis	EWS	Staff felt that they had not been educated to an adequate level – training lacking<br><br>Negative attitude towards calling for help – fears of appearing inadequate
Cioffi, J. (2000). Nurses' experiences of making decisions to call	Australia	Unstructured interviews with nurses who had	Simple code and retrieve	EWS	Reluctance to activate – doubting ability; fears of appearing inadequate; decisions made based on the perceived availability of resources/business of medical staff/time of day all had an impact on decisions to activate the MET

For peer review only - http://bmjopen.bmj.com/site/about/guidelines.xhtml

Author	Country	Methodology	Analysis	Search Area	Evidence Contribution
emergency assistance to their patients. Journal of Advanced Nursing, 32(1), 108-114.		activated the medical emergency team (MET)			Importance of having staff concern in the MET criterion
Cioffi, J. (2000). Recognition of patients who require emergency assistance: a descriptive study. Heart & Lung, 29(4), 262-268.	Australia	Unstructured interviews	Simple code and retrieve	EWS	Importance of having staff concern in a calling criteria<br><br>Reluctance to activate – business of ward a factor
Cioffi, J., Salter, C., Wilkes, L., Vonu-Boriceanu, O., & Scott, J. (2006). Clinicians' responses to abnormal vital signs in an emergency department. Australian Critical Care, 19(2), 66-72.	Australia	Focus groups with clinicians and nurses exploring their responses to abnormal vital signs	Constant comparison	EWS	Availability of equipment an issue/staffing pressures; staff unable to carry out routine monitoring that would enable the detection of abnormal vital signs (AVS)/escalation hampered because of difficulty finding the appropriate senior person<br><br>.<br><br>Medical emergency team (MET) criteria used to confirm or identify deterioration depending on experience<br><br>Negative attitude towards asking for help - lack of confidence questioning peers/fear of being reprimanded /feeling the situation was under control
de Groot, J F. et al (2018) "Implementing	Netherlands	Retrospective patient review &	Descriptive statistics and	PEWS	Easily approachable nurses and physicians, as well as good communication, were considered to be vital for timely intervention in cases of clinical

For peer review only - http://bmjopen.bmj.com/site/about/guidelines.xhtml

Author	Country	Methodology	Analysis	Search Area	Evidence Contribution
Paediatric Early Warning Scores Systems in the Netherlands : Future Implications.. *BMC Pediatrics,* 18: 128		semi structured interviews with professionals	grounded theory		deterioration in paediatric patients.<br><br>Facilitators for the implementation of registration of PEWS included the integration of PEWS scores into the electronic patient records
Dean, BS, Decker MJ, Hupp D, Urbach AH, Lewis E, Benes-Stickle J (2008) Condition HELP: a pediatric rapid response team triggered by patients and parents. *J Health Qual*, 30, 3, 28-31	U.S.A.	Two-year reflection following implementation of Condition Help (ConditionH)	Descriptive statistics	Family involvement	ConditionH criteria for activation<br><br>Concern that family activated RRS could divert attention away from resources<br><br>Clinician involvement important<br><br>Daily 'patient rounds' involving patients and families is useful<br><br>Patients and families have access to relevant information and understand the medical information and care plans.
Demmel KM, Williams L, Flesch L. (2010) Implementation of the Pediatric Early Warning Scoring System on a Pediatric Hematology/Oncology Unit. *Journal of Pediatric Oncology Nursing*,	U.S.A.	Discussion of the set-up and implementation of a paediatric early warning scoring tool and an associated algorithm	Rapid Plan-Do-Study-Act (PDSA) cycles were implemented using small tests of change.	PEWS	Education package developed around the history and development of PEWS along with the rationale for and the goals of the initiative. The scoring process was explained and how it would be integrated into routine nursing assessments; normal vital sign parameters were reviewed.<br><br>Importance of common information spaces & display of activation criteria throughout the hospital<br><br>Senior lead commitment & importance of champions integral for implementation

For peer review only - http://bmjopen.bmj.com/site/about/guidelines.xhtml

Author	Country	Methodology	Analysis	Search Area	Evidence Contribution
27(4):229-240					
Donohue, L. and Endacott, R. (2010) Track, trigger and teamwork: Communication of deterioration in acute medical and surgical wards. *Intensive and Critical Care Nursing*, 26, 10-17	U.K.	Qualitative design with critical incident technique. Semi-structured interviews with nurses and the outreach team	Thematic analysis	EWS	Some resistance to escalation – clinicians preferring to deal with patient problems within their own team<br><br>Inexperienced staff teaming up with more experienced staff once patient deterioration was recognised
Downey, C.L., Tahir, W., Randell, R., Brown, J.M. and Jayne, D.G. (2017) Strengths and limitations of early warning scores: A systematic review and narrative synthesis. *International Journal of Nursing Studies*, 76, 106- 119	U.K.	Narrative review	"Patterns were identified and translated to themes, which were further refined using an iterative process".	PEWS	Impact on communication – packaging information. Facilitates communication across hierarchies.
Endacott, R., & Westley, M. (2006). Managing patients at risk of	Australia	Questionnaire, in-depth interviews and	Content analysis and constant comparison	EWS	Art of referral important – using the right language and suggesting actions that would be acceptable to the doctor

For peer review only

For peer review only - http://bmjopen.bmj.com/site/about/guidelines.xhtml

Author	Country	Methodology	Analysis	Search Area	Evidence Contribution
deterioration in rural hospitals: a qualitative study. Australian Journal of Rural Health, 14(6), 275-279		observations			Availability of equipment a factor<br><br>Negative attitude towards calling for help; Escalation dependent upon perceived capability of medical staff
Ennis, L (2014) Paediatric early warning scores on a children's ward: a quality improvement initiative. *Nursing children and young people*, 26, 7, 25-31	Ireland	Description of implementation of paediatric early warning score (PEWS) and subsequent audit (prospective cohort observational study)	Simple descriptive statistics of numbers children triggering PEWS and compliance with escalation protocol	PEWS	Structured education and training programme on the use of ISBAR (Identify, Situation, Background,<br><br>Assessment and Recommendation) and PEWS was provided and nurse manager/staff nurse in charge should review any educational requirements in completing PEWS, particularly for relief staff<br><br>Common information spaces important & display of activation criteria throughout the hospital<br><br>Usefulness of ISBAR as a communication tool<br><br>Senior lead commitment – PEWS management policy developed/senior staff promote and reinforce use of PEWS
Entwistle, V. (2004). Nursing shortages and patient safety problems in hospital care: is clinical monitoring by	U.S.A.	Editorial	N/a	Family involvement	Little evidence/no evaluations of policies or practices that encourage and support family involvement in clinical monitoring<br><br>Propose the innovative practice of interdisciplinary rounds where families are invited, and communication is directed to the patient and family

For peer review only - http://bmjopen.bmj.com/site/about/guidelines.xhtml

Author	Country	Methodology	Analysis	Search Area	Evidence Contribution
families part of the solution? Health Expectations, 7, pp.1–5 (editorial)					
Gerdick, C., Vallish, R., Miles, K., Godwin, S., Wludyka, P. and Panni, M. (2010) Successful implementation of a family and patient activated rapid response team in an adult level 1 trauma center. *Resuscitation*, 81, 1676-1681	U.S.A.	Routine data collection for number of rapid response team (RRT) calls and the result of these activations and patient/family survey relating to RRT activation	Statistical analysis	Family involvement	Direct mechanism for families to activate the RRT<br><br>Barriers to family activation highlighted, specifically professional resistance<br><br>Physician and leadership support important to overcome barriers
Gill, F.J., Leslie, G.D. and Marshall, A.P. (2016) Family initiated escalation of care for the deteriorating patient in hospital: Family centred care or just "box ticking". *Australian Critical Care*, 29, 195-200	Australia	Commentary drawing together family centred care concepts, the NSQHS Standards and the development of family-initiated care in Australia	N/a	PEWS	Family activated RRTs now increasingly common in Australia. In the first instance, families need to be aware of the policy.<br><br>Stress the importance of understanding the number and nature of the call.<br><br>Reports on health professional's resistance to it.<br><br>Families need vigilance to escalate care. Need resources in order to negotiate hierarchies and boundaries

For peer review only - http://bmjopen.bmj.com/site/about/guidelines.xhtml
For peer review only

Author	Country	Methodology	Analysis	Search Area	Evidence Contribution
Greenhouse, P., Kuzminsky, B., Martin, S., and Merryman, T. (2006) Calling a Condition H(elp). *AJN*, 106, 11	U.S.A focus	Discussion about the implementation of Condition Help (ConditionH)	Descriptive statistics	Family involvement	Appropriateness of calls is reported rather that why they are made<br><br>Note some scepticism and wariness among nurses and physicians
Hueckel, R. M., Mericle, J. M., Frush, K., Martin, P. L. and Champagne M. T. (2012). Implementation of Condition Help. Family Teaching and Evaluation of Family Understanding. J Nurs Care Qual Vol. 27, No. 2, pp. 176–181	U.S.A.	Scripted family teaching about RRT activation at the time of patient admission from Condition Help (ConditionH).	Descriptive statistics about delivery of educational programme and rapid response team (RRT) call-out; survey testing family understanding	Family involvement	Description of ConditionHelp<br>Appropriateness of calls is reported rather that why they are made
James, J., Butler-Williams, C., Hunt, J. and Cox, H. (2010) Vital signs for vital people: an exploratory study into the role of the Healthcare Assistant in recognising, recording and	U.K.	Postal survey with Healthcare Assistants (HCAs) utilising closed and open questions	Descriptive statistics & content analysis of qualitative data	Observations and monitoring	Workload and ward distractions a barrier to activation, such as time spent locating equipment.<br><br>Disinclination to seek help from senior staff/clinicians

For peer review only - http://bmjopen.bmj.com/site/about/guidelines.xhtml

Author	Country	Methodology	Analysis	Search Area	Evidence Contribution
responding to the acutely ill patient in the general ward setting. *Journal of Nursing Management*, 18, 548-555					
Jensen, C. et al. (2018) Pediatric Early Warning Score Systems, Nurses Perspective – A Focus Group Study. *Journal of Pediatric Nursing*, 14, e16–e22	Denmark	Focus group exploring nurse' experiences with PEWS	Qualitative meaning condensation analysis	PEWS	PEWS as a nursing tool and therefore not valued by medic - no universal language because of this; "when you call and saythat they have a PEWS score of 5, then they don't know what 5 means" (FG2 P1).
Kaul, M., Snethen, J., Kelber, S., Zimmanck, K., Maletta, K. and Meyer, M. (2014) Implementation of the bedside paediatric early warning system (BedsidePEWS) for nurse identification of deteriorating patients. *Journal for specialists in pediatirc nursing*, 19,	U.S.A	Descriptive cross-sectional study; nurse and medical staff survey	Descriptive statistics	PEWS	Noted that the score provides a 'universal language' and interdisciplinary communication

For peer review only - http://bmjopen.bmj.com/site/about/guidelines.xhtml

Author	Country	Methodology	Analysis	Search Area	Evidence Contribution
339-349					
Lobos AT, Fernandes R, Ramsay T, McNally JD. (2014) Patient characteristics and disposition after pediatric medical emergency team (MET) activation: disposition depends on who activates the team. Hospital Pediatrics. 4(2):99-105	Canada	Implementation discussion	Simple descriptive statistics	PEWS	Situation Background Assessment Recommendation (SBAR) helps to establish a common language and guide escalated events<br><br>Negative attitude towards calling for help – traditional hierarchies a barrier to activation/concerns about communication between primary and responding team<br><br>No false alarms and de-brief useful<br><br>Importance of champions (utilising a social marketing approach) to encourage 'inter-professional collaboration & advisory group to help establish a sense of ownership<br><br>Lack of support from superiors means less likely to escalate
Mackintosh, N., Rainey, H. and Sandall, J. (2012a) Understanding how rapid response systems may improve safety for the acutely ill patient: learning from the frontline. *BMJ Qual Saf*, 21, 135-144	U.K.	Comparative case study a rapid response system (RRS) using ethnographic methods including observations,	Inductive and deductive coding facilitated by NVivo. Also utilised theme building and structuring methods from framework	EWS	Availability of equipment an issue where the track and trigger tool (TTT) was electronic<br><br>Gave junior staff license to escalate care.<br>Additionally, 'while standardisation of practice clearly has its benefits, it also comes at a cost that these tools attenuate lower level staff's authority and ability to persuade staff higher up in the organisation of the credibility of their knowledge' p 143.

For peer review only - http://bmjopen.bmj.com/site/about/guidelines.xhtml

Author	Country	Methodology	Analysis	Search Area	Evidence Contribution
		interviews and documentary review.	analysis whilst also informed by other theoretical frameworks such as 'technology-in-practice'.		Efforts to develop junior staff's communication and clinical understanding need to acknowledge power dynamics at play.<br><br>Usefulness of situation-background-assessment-recommendation' (SBAR) communication tool as part of the escalation policy as reported by staff (not seen in action).<br><br>Negative attitude towards escalation – difficulty in summoning a response<br><br>Senior lead commitment to patient safety was important. Zero tolerance for cardiac arrest was championed by senior staff<br><br>Night-time/out of hours pressures identified
Mackintosh, N., Humphrey, C., & Sandall, J. (2014a). The habitus of 'rescue' and its significance for implementation of rapid response systems in acute health care. Social Science & Medicine, 120, 233-242	U.K.	Ethnographic perspective; observations, semi-structured interviews	Data were inductively and deductively coded and organised thematically.	EWS	Negative attitude towards seeking help. Escalating care outside the parameters marked by a track and trigger tool proved difficult; Power struggles identified – junior staff have difficulty persuading more senior staff of the credibility of their knowledge<br><br>Difficulties in activation across professional boundaries
Massey, D., Chaboyer,	Australia	In depth semi	Inductive	EWS	Common information spaces useful. Display of activation criteria throughout

For peer review only - http://bmjopen.bmj.com/site/about/guidelines.xhtml

Author	Country	Methodology	Analysis	Search Area	Evidence Contribution
W., & Aitken, L. (2014). Nurses' perceptions of accessing a Medical Emergency Team: a qualitative study. Australian Critical Care, 27(3), 133-138		structured interviews	approach – thematic analysis		hospital<br><br>General negative attitude towards calling for help – appearing inadequate in front of others<br><br>Importance of leadership support<br><br>Peers support – would often consult their colleagues
McCabe, A. and Duncan, H. (2009) "Paediatric early warning systems: where do we go from here?" *Paediatric Nursing*, 21, 1, 14-17	U.K.	Opinion piece about lessons to be learnt from the adult experience of implementing early warning systems	N/a	PEWS	Specific education package needed on how to use an early warning system (EWS) and on basic clinical assessment, guidance and standardisation of observation and monitoring. Advocate situational simulated scenario education and e-learning<br><br>Highlight the usefulness of communication tools such as situation, background, assessment, recommendation (SBAR) for establishing roles and responsibilities, engaging them in making an appropriate management plan that can, if necessary, be escalated<br><br>Senior lead commitment key - reflected in resources and education - to improve the safety and quality of care of hospitalised patients<br><br>Families need to be empowered to request a patient review
McDonnell, A., Tod, A., Bray, K., Bainbridge, D.,	U.K.	Single centre, mixed method	Statistical analysis and thematic	EWS	Rolling education programme for all nurses on the recognition and response to deteriorating patients & an overview of the T&Ts

For peer review only - http://bmjopen.bmj.com/site/about/guidelines.xhtml

Author	Country	Methodology	Analysis	Search Area	Evidence Contribution
Adsetts, D., & Walters, S. (2013). A before and after study assessing the impact of a new model for recognizing and responding to early signs of deterioration in an acute hospital. *Journal of Advanced Nursing,* 69(1), 41-52.		before and after study including a survey to measure changes in nurses' knowledge after implementation of a track and trigger system (T&Ts). Also, qualitative interviews	framework analysis		Workplace pressures; nurses concerned that they couldn't always summon a timely response from doctors /night time pressures also identified<br><br>Need for staff concern in T&Ts
Monaghan A. (2005) Detecting and managing deterioration in children. *Paediatric Nursing.* 17(1):32-35	U.K. focus	Commentary on the development of the Brighton paediatric early warning score and setting up a paediatric critical care outreach team.	Simple descriptive statistics of all activations, actions and outcomes during the first three months of implementation	PEWS	Education based model was developed to assist in recognising deterioration<br><br>Temporary staff/workplace pressures impact upon staffs' ability to detect deterioration

For peer review only - http://bmjopen.bmj.com/site/about/guidelines.xhtml

Author	Country	Methodology	Analysis	Search Area	Evidence Contribution
Paciotti B, Roberts KE, Tibbets KM, Weirich Paine C, Keren R, Barg FK, Holmes J H and Bonafide CP (2014). Physician attitudes toward family-activated medical emergency teams for hospitalized children. The Joint Commission Journal on Quality and Patient Safety, 40 (4)	U.S.A.	Semi-structured interviews with clinicians to explore physicians viewpoints on families facilitating the identification of children with a deteriorating condition.	Grounded theory and constant comparison	Family involvement	Concerns that resources would be diverted away with an increase in calls – not supported
Pattison, N., & Eastham, E. (2012). Critical care outreach referrals: a mixed-method investigative study of outcomes and experiences. *Nursing in Critical Care,* 17(2), 71-82.	U.K.	Mixed method study looking at the impact of a critical care outreach team (CCOT)	Statistical analysis and grounded theory	EWS	Availability of equipment an issue/workload<br><br>Negative attitude towards calling for help – situation under control/ward business<br><br>Inexperienced staff teaming up/checking with peers before calling the CCOT
Pearson G & Duncan H. (2011) Early warning	U.K.	Brief review of the evidence	N/a	PEWS	Team training & education is important increasing confidence in the use of medical language and empowering bedside carers. 'Although doing

For peer review only - http://bmjopen.bmj.com/site/about/guidelines.xhtml

Author	Country	Methodology	Analysis	Search Area	Evidence Contribution
systems for identifying sick children Paediatrics and Child Health. 21(5): 232-233		base surrounding PEWS together with reflections from their own experiences from the Birmingham Children's Hospital			observations is fundamental to nursing practice many … have not been taught a structured approach to assessment'. Advocate a simulated environment<br><br>Value of a more structured approach to communication - Advocate the use of a shared communication model such as SBAR (Situation, Background, Assessment and Recommendation) to communicate findings to superiors.<br><br>Need for senior commitment – cultural change may be required to ensure management support (reflected in resources and education) /Importance of champions
Salamonson, Y., van Heere, B., Everett, B., & Davidson, P. (2006). Voices from the floor: Nurses' perceptions of the medical emergency team. *Intensive & Critical Care Nursing*, 22(3), 138-143.	Australia	Survey with closed and open questions to examine perceptions of and satisfaction with the medical emergency team (MET)	Descriptive statistics and content analysis	EWS	Need for more education on deterioration identified<br><br>Negative attitude towards asking for help; attitude of MET team a barrier to activation
Shearer, B., Marshall, S., Buist, M. D., Finnigan, M., Kitto, S., . . . Ramsay, W. (2012).	Australia	A multi-method study;<br>A point prevalence	Iterative coding	EWS	Adequate staffing and a lack of beds on critical care leads to a failure to activate the RRS<br><br>Score rarely the single determining factor in activation despite the fact that staff

For peer review only - http://bmjopen.bmj.com/site/about/guidelines.xhtml

Author	Country	Methodology	Analysis	Search Area	Evidence Contribution
What stops hospital clinical staff from following protocols? An analysis of the incidence and factors behind the failure of bedside clinical staff to activate the rapid response system in a multi-campus Australian metropolitan healthcare service. BMJ Quality & Safety, 21(7), 569-575		survey; A prospective audit of all patients experiencing a cardiac arrest, unplanned intensive care (ICU) unit admission or death over an 8-week period. Structured interviews with staff to explore cognitive and sociocultural barriers to activation			recognised patients met activation criteria. Data from the point prevalence study confirm this as only one patient had a serious adverse event<br><br>Negative attitude towards calling for help – situation under control; treating team had expertise to treat (particularly when the physiological instability was in the area of expertise of the treating team)<br><br>Traditional (intra-professional clinical) hierarchies a barrier to activation
Sonning, K, et al. (2018) "A Survey of Healthcare Professionals' Experiences with the Paediatric Early Warning	Norway	Questionnaire of a sample of staff who use PEWS	Descriptive statistics	PEWS	Nurses gain self-confidence. More effective communication

For peer review only - http://bmjopen.bmj.com/site/about/guidelines.xhtml

Author	Country	Methodology	Analysis	Search Area	Evidence Contribution
Score (PEWS)." *Norwegian Journal of Clinical Nursing* 12(64605).					
Stewart, J., Carman, M., Spegman, A., & Sabol, V. (2014). Evaluation of the effect of the modified early warning system on the nurse-led activation of the rapid response system. *Journal of Nursing Care Quality*, 29(3), 223-229.	Sweden	Mixed method. Retrospective review of records and nurse led focus groups	Statistical analysis and content analysis	EWS	The RRS was valuable for junior staff escalating care across hierarchical and professional boundaries<br><br>Senior lead commitment – culture of support promoted by nursing administrators
Van Voorhis, K.T. and Scahde Willis, T. (2009) Implementing a pediatric rapid response system to improve quality and patient safety. *Pediatr Clin N Am*, 56, 919-933	U.S.A.	Discussion paper highlighting the process of developing a paediatric rapid response system (RRS).<br><br>The system was evaluated by	N/a	PEWS	Display of activation criteria throughout hospital on lanyards and use of white boards useful<br><br>De-briefing following activation and a commitment to no false alarms is encouraged<br><br>Senior lead commitment – administrative arm of the RRS vital<br><br>Utilises ConditionHelp. The appropriateness of calls was facilitated by the 'no false alarms' culture.

For peer review only - http://bmjopen.bmj.com/site/about/guidelines.xhtml

Author	Country	Methodology	Analysis	Search Area	Evidence Contribution
		prospectively collected data recorded on RRS activation forms and existing performance improvement database information.			
Vries, A., Draaisma, J. and Fuijkschot, J. (2017) Clinician Perceptions of an Early Warning System on Patient Safety. *Hospital Pediatrics*, 7, 10, 579-586	Netherlands	Semi-structured interview	Qualitative content analysis	PEWS	PEWS facilitated communication across hierarchies
Watson, A., Skipper, C., Steury, R., Walsh, H. and Levin, A. (2014) Inpatient nursing care and early warning scores: a workflow mismatch. *J Nurs Care*	U.S.A.	Mixed method, retrospective medical record observations and observations of nurse interactions in	Observation analysis, although this is not described, and statistical analysis	PEWS	Availability of equipment a factor<br><br>Score rarely the determining factor in escalation<br><br>SBAR

For peer review only - http://bmjopen.bmj.com/site/about/guidelines.xhtml

Author	Country	Methodology	Analysis	Search Area	Evidence Contribution
Qual, 29, 3, 215-222		one-minute blocks.			

FIGURE 1 PRISMA DIAGRAM

For peer review only

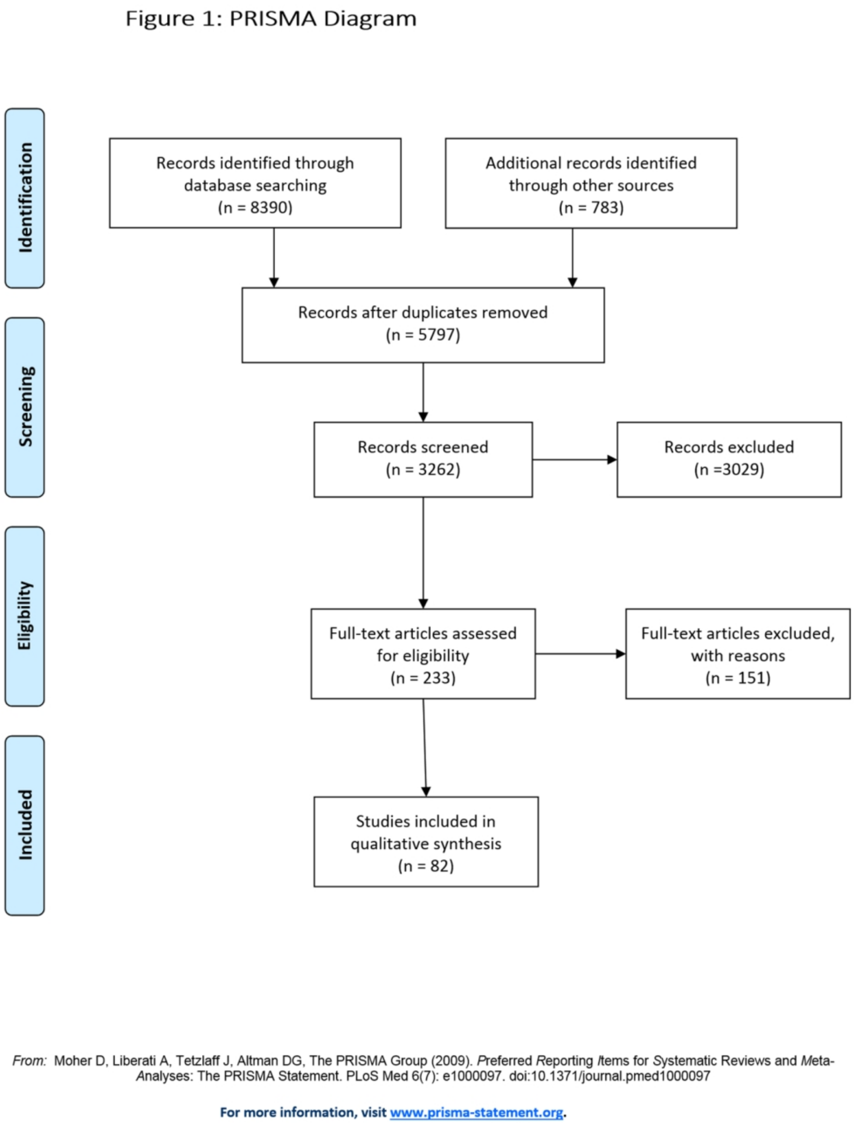

Figure 1: PRISMA Diagram

Appendix 1: Data Extraction Template 1

PUMA literature review template

Study/Paper	
Authors, title, date, source	
Purpose	
Contribution to review	
Design/Methods	
Participants/Setting	
Data type	
Theoretical framework	
Quality Appraisal Tool	
Quality Assessment	
Intervention type	
Formal study finding	
Recommendations	

For peer review only

Context	
History of intervention	
Country	
Organisation type (i.e. DGH, tertiary hospital, primary care)	
PEWS (efferent) wider activity system elements and inter-relationships with TTT [decision algorithms, CCOT, MET, ICU liaison, PICU, HDU]	
Infra-structural context [other related artefacts]	
Related activity systems [QI, audit, governance, referral processes and inter-relationships with TTT]	
Socio-cultural context [including experience of QI, stability of team and division of labour, teaching-non-teaching etc]	
Wider policy/organisational context	
**Intervention (TIDieR)**	
Name	
Rationale, theory or goal of the elements – active ingredients - identified as essential to the intervention [DA: this could be explicit or implicit in the paper]	
What (materials): Describe any physical or informational	

For peer review only

materials used in the intervention, including those provided to participants or used in intervention delivery or in training of intervention providers.	

**TTT Affordances (including but not limited to indicative list)**	
Paper/electronic	
What observations/information are recorded?	
How is data displayed?	
Single parameter/ Aggregated weighting	
Response algorithm(s) (including details)	
Parental/patient involvement	
Manual or automated monitoring	
Proactive intermittent/continuous monitoring	
Linked artefacts (e.g SBAR)	
Audit/performance surveillance	

For peer review only

What (procedures): describe each of the procedures, activities, and/or processes used in the intervention, including any enabling or supportive activities.	
Who provided: For each category of intervention provider (for example, nursing assistant, nurse, junior doctor) describe their expertise, background and any specific training given and also the roles and relationships within the overall division of labour.	
How: Describe the mechanisms (implicit or explicit) about how the intervention, or intervention components have their effects	
How: Describe the modes of TTT delivery (such as face to face or some other mechanism such as phone or internet), or whether the intervention was provided individually or in a group.	
When and how much: Describe the number of times the intervention was delivered and over what period of time,	

For peer review only

including the number of sessions, schedule and intensity of dose (universal or selective application)				
Tailoring: If the intervention was planned to be personalised or adapted, then describe what, why, when and how.				
Modifications: If the intervention was modified during the course of the study, describe the changes (What, why, when, and how)				
Assessment of intervention adherence or fidelity, describe how and by whom, and if any strategies were used to maintain or improve fidelity, describe them				
If intervention adherence or fidelity was assessed, describe the extent to which the intervention was delivered as planned				
**Implementation (NPT) (questions modified from Murray et al. 2010 and kennedy et al 2013)**				
Coherence [i.e. the meaning attributed to PEWS and whether it makes sense for users e.g. cultural and scientific legitimacy, feasibility]	Policy	Organisational	Professional	Patient/family

For peer review only

• Evidence that the intervention was easy to describe<br>• Evidence that it was recognised as different from existing ways of working by stakeholders<br>• Evidence that it has a clear purpose for all relevant actors<br>• Evidence that stakeholders recognise the benefits the intervention will bring and to whom<br>• Evidence that the intervention fitted with the overall goals and activity of the organisation				
Cognitive Participation [i.e. commitment and engagement with the intervention]<br>• Evidence that the target group see the intervention was a good idea<br>• Evidence that they were prepared to invest time and energy and work in it?				

For peer review only

• Evidence that the stakeholders thought their engagement with the intervention was appropriate<br>• Evidence that the stakeholders have taken steps to sustain the use of the intervention				
Collective Action [what are the skills and resources required to enact the intervention?]<br>• How did the intervention affect the user groups?<br>• What work was undertaken to operationalise the intervention?<br>• Did it promote or impede their work?<br>• Did staff require extensive training before they can use it?<br>• How compatible was the intervention with existing work practices?<br>• What impact did it have on the division of				

For peer review only

labour, resources, power and responsibility between different professional groups?<br>• Did it fit with the overall goals and activity of the organisation?				
Reflexive Monitoring [i.e methods for formal and informal evaluation of the effects of the intervention]<br>• Did stakeholders take practical steps to measure the influence of the intervention?<br>• How did users perceive the intervention once it has been in use for a while?<br>• Was the intervention seen as advantageous for patients and staff?<br>• Was it clear what effects the intervention has had (including unintended consequences)?<br>• Did users' contribute feedback on the				

For peer review only

intervention once it is in use?<br>• Was the intervention be adapted/improved on the basis of this experience?				
**Findings (positive, negative and unintended consequences) and useful content**				
**Emergent theories about key ingredients and mechanisms (including summary of supporting evidence)**				
**Emergent theories of the factors necessary for paediatric EWS to be normalised (including summary of supporting evidence)**				
**Additional Insights of note (including references to follow up)**				

For peer review only

Appendix 2: Search Methodology

Search Methods

Database Search

A comprehensive search was conducted across a wide-ranging set *of databases* from 1995 to May 2018 to identify relevant evidence/studies in English language on

- Paediatric Early Warning Systems (all study types).

A preliminary search strategy was developed using a set of key papers known to the group for Ovid Medline using both text words and Medical subject headings. A further three systematic searches was conducted across a range of databases from 1995 to September 2016 to identify relevant studies in the English language papers reporting on:

- Adult Early Warning Systems (qualitative studies only)
- Interventions to improve situational awareness (all study types)
- Structured communication tools for handover and handoff (all study types)

The Medline search strategy was translated to use across the rest of the databases.

The focus of the search strategy was to achieve high sensitivity and specificity for retrieving studies relevant to the review question. The Medline search strategy was modified according to the indexing systems of the other databases.

Table 1.

**Databases**
British Nursing Index
CINAHL (Cumulative Index of Nursing and Allied Health Literature)
Cochrane Central Register of Controlled Trials
Database of Abstracts of Reviews of Effectiveness
EMBASE
HMIC (Health Management Information Centre)
Medline
Medline in Process
Scopus
Web of Knowledge (Science Citation Indexes)

Additional searches

In addition to the above databases, we searched both published and unpublished literature. To identify supplementary papers, information on studies in progress, unpublished research or research reported in the grey literature was identified through searching a range of relevant websites and trial registers including Clinical Trials.gov. To identify published resources that had not yet been catalogued in the electronic databases, recent editions of key journals were hand-searched.

**Trial Registers**
ClinicalTrials.gov<br>https://clinicaltrials.gov/
UK Clinical Trials Gateway

For peer review only

http://www.ukctg.nihr.ac.uk/default.aspx
The WHO trial search portal for studies worldwide: http://apps.who.int/trialsearch
**Journal site**
BMJ http://www.bmj.com/theBMJ
BMJ Quality and safety http://qualitysafety.bmj.com/
Websites and organisations
The Health Foundation

Identify relevant studies

The search results would be imported into reference management database *Endnote.* Duplicate references and clearly irrelevant citations will be removed. All remaining studies will then be downloaded into Dropbox for reviewers to screen for relevance.

Medline Search Strategy: Adult Early Warning Systems

1. ("early warning" adj5 scor*).ab,ti.
2. ("early warning" adj5 system* adj5 (deteriorat* or mortality or death or outcome* or harm* or safety)).ab,ti.
3. "acute illness severity".mp.
4. "early medical intervention"/ and ((prevent* or reduc* or improv*) adj5 (deteriorat* or mortality or death or outcome* or harm* or safety)).ab,ti.
5. ("early medical intervention" adj5 (tool* or scor* or index* or indicator* or indice* or assessment* or guide* or instrument* or criteria or parameter* or deteriorat* or mortality or death or monitor* or outcome* or harm* or safety)).ab,ti.
6. exp Health Status Indicators/ and ((tool* or scor* or index* or indicator* or indice* or assessment* or instrument* or criteria or parameter*) adj3 ((prevent* or reduc* or improv*) adj3 (deteriorat* or mortality or death or outcome* or harm* or safety))).ab,ti.
7. "Severity of Illness Index"/ and ((tool* or scor* or index* or indicator* or indice* or assessment* or instrument* or criteria or parameter*) adj5 ((prevent* or reduc* or improv*) adj5 (deteriorat* or mortality or death or outcome* or harm* or safety))).ab,ti.
8. exp Hospitals/ and ((Detecting or managing) adj3 deterioration).tw.
9. ("medical device" adj3 (prevent* or reduc* or improv*)).mp.
10. ("alert criteria" or "activation criteria" or "calling criteria").tw.
11. Hospital Rapid Response Team/
12. Clinical Alarms/
13. (outreach adj3 emergency).tw.
14. VitalPAC Early Warning Score.tw.
15. medical emergency team.tw.
16. Rapid Response Systems.mp.
17. Rapid Response Team.tw.
18. (((Detecting or managing) adj3 deterioration) and warning).tw.
19. track-and-trigger system.tw.

For peer review only

20. (Track adj trigger).tw.
21. (Track and trigger).tw.
22. trigger tools.tw.
23. SBAR technique*.mp.
24. ("rapid response" adj5 (prevent* or reduc* or improv*)).tw.
25. (score adj3 severity of illness).tw.
26. or/1-25
27. (qualitative or ethnograph* or thematic analysis or grounded theory or audio-recorded or transcribed or verbatim or ethnograph* or content analysis technique).ti,ab.
28. (("semi-structured" or semistructured or unstructured or informal or "in-depth" or indepth or "face-to-face" or structured or guide) adj3 (interview* or discussion* or questionnaire*)).ti,ab.
29. ((field or case) adj (stud* or research)).ti,ab.
30. Focus groups/ or Qualitative research/ or Interviews as topic/ or Interview, Psychological/ or ((focus or discussion) adj group*1).ti,ab.
31. (Questionnaires/ or interviews as topic/ or interview, psychological/) and (experience* or predictor* or determinant* or barrier* or facilitator* or enabler* or factor* associat* or perception* or perceive* or attitude* or view*1 or viewpoint* or standpoint* or encounter* or experience* or story or stories or narrative*1 or theme*1 or opinion* or concerns or motivat* or need*1).ti,ab.
32. (cross-sectional studies/ or cross-sectional survey.ti,ab. or correlation study.ti,ab.) and (predictor* or determinant* or barrier* or facilitator* or enabler* or factor* associat* or perception* or perceive* or attitude* or view*1 or viewpoint* or standpoint* or encounter* or experience* or story or stories or narrative*1 or theme*1 or opinion* or concerns or motivat* or need*).ti,ab.
33. process evaluation/ or process evaluation.ti,ab.
34. mixed method*1.ti,ab.
35. ((assoc* factor*1 or predictor* or determinant* or barrier* or facilitator* or enabler*) adj3 (interview* or survey* or questionnaire* or study)).ti,ab.
36. *motivation/
37. ((perception* or perceive* or attitude* or view*1 or viewpoint* or standpoint* or encounter* or experience* or story or stories or narrative*1 or description* or theme* or opinion* or need*1 or concerns or motivat*) adj3 (interview* or survey* or questionnaire* or study or explor* or evaluate or investigate* or analys* or collect*)).ti,ab.
38. (themes adj3 (identif* or analy* or review or explor* or investigat*)).ti,ab.
39. "attitude of health personnel"/ or *attitude to health/
40. exp emotions/
41. consumer satisfaction/
42. personal satisfaction/
43. exp professional-patient relations/
44. exp interprofessional relations/
45. "Health Services Needs and Demand"/
46. or/27-45
47. 26 and 46
48. limit 47 to (english language and humans and yr="1995 -Current")

Medline Search Strategy: Structured communication tools for handover and handoff

For peer review only

1. Situation Background Assessment Recommendation.tw.
2. SBAR.mp.
3. (ABC and "collaborative care").tw.
4. Patient Handoff/
5. Patient Discharge/ and ABC.mp.
6. exp Patient Transfer/ and checklist.mp.
7. (handoff adj3 communication).tw.
8. (handoff adj5 (tool or approach or technique or method)).tw.
9. ("information transfer" and "emergency care").tw.
10. Patient Discharge/ and information transfer.tw.
11. ("information transfer" and "critical care").tw.
12. ("information transfer" and handoff).tw.
13. written checklist.tw.
14. ("Rapid Syndrome Validation Project" or RSVP).tw.
15. "Communication tools".tw.
16. "Escalation of care".tw.
17. or/1-16
18. limit 17 to (english language and humans and yr="1995 -Current")

Medline Search Strategy: Paediatric Early Warning Systems to include observation and training

1. ("early warning" adj5 scor*).ab,ti.
2. ("early warning" adj5 system* adj5 (deteriorat* or mortality or death or outcome* or harm* or safety)).ab,ti.
3. "acute illness severity".mp.
4. "early medical intervention"/ and ((prevent* or reduc* or improv*) adj5 (deteriorat* or mortality or death or outcome* or harm* or safety)).ab,ti.
5. ("early medical intervention" adj5 (tool* or scor* or index* or indicator* or indice* or assessment* or guide* or instrument* or criteria or parameter* or deteriorat* or mortality or death or monitor* or outcome* or harm* or safety)).ab,ti.
6. exp Health Status Indicators/ and ((tool* or scor* or index* or indicator* or indice* or assessment* or instrument* or criteria or parameter*) adj3 ((prevent* or reduc* or improv*) adj3 (deteriorat* or mortality or death or outcome* or harm* or safety))).ab,ti.
7. "Severity of Illness Index"/ and ((tool* or scor* or index* or indicator* or indice* or assessment* or instrument* or criteria or parameter*) adj5 ((prevent* or reduc* or improv*) adj5 (deteriorat* or mortality or death or outcome* or harm* or safety))).ab,ti.
8. exp Hospitals/ and ((Detecting or managing) adj3 deterioration).tw.
9. ("medical device" adj3 (prevent* or reduc* or improv*)).mp.
10. ("alert criteria" or "activation criteria" or "calling criteria").tw.
11. Hospital Rapid Response Team/
12. Clinical Alarms/
13. (outreach adj3 emergency).tw.
14. VitalPAC Early Warning Score.tw.
15. medical emergency team.tw.

For peer review only

16. Rapid Response Systems.mp.
17. Rapid Response Team.tw.
18. (((Detecting or managing) adj3 deterioration) and warning).tw.
19. track-and-trigger system.tw.
20. (Track adj trigger).tw.
21. (Track and trigger).tw.
22. trigger tools.tw.
23. SBAR technique*.mp.
24. ("Situation Background Assessment Recommendation" or SBAR).tw.
25. (patient* adj3 deteriorat*).tw.
26. (deterioration adj3 hospital).tw.
27. (patient deterioration adj4 hospital).tw.
28. (Patients adj4 adverse event).tw.
29. clinical deterioration.tw.
30. ("rapid response" adj5 (prevent* or reduc* or improv*)).tw.
31. (score adj3 severity of illness).tw.
32. Vital signs.tw.
33. or/1-26
34. P?ediatric Early Warning.mp.
35. p?ediatric alert.tw.
36. p?ediatric early warning systems.mp.
37. p?ediatric risk of mortality.tw.
38. P?ediatric Rapid Response Team.tw.
39. Point-of-Care Systems/ and ((paediatric or pediatric) adj3 (improve or identify or detect* or outcome or early or critical or emergency)).tw.
40. P?ediatric Advanced Warning Score.tw.
41. neonatal early warning.tw.
42. infant early warning.tw.
43. p?ediatric rapid response.tw.
44. Bedside paediatric early warning.tw.
45. Bedside PEWS.tw.
46. or/34-45
47. 33 or 46
48. Health Plan Implementation/
49. (implement* or applicat* or execute).tw.
50. (observ* or monitoring or monitor or education).tw.
51. Risk Assessment/
52. Education/
53. Education, Continuing/
54. "Hospitals, Teaching"/
55. Decision Making/
56. Safety Management/
57. Patient Simulation/
58. Awareness/
59. Knowledge/
60. *"Attitude of Health Personnel"/
61. *"Education, Medical, Continuing"/

For peer review only

62. *"Interdisciplinary Communication"/
63. Communication/
64. Monitoring, Physiologic/
65. Decision Making/
66. Judgment/
67. Needs Assessment/
68. Interprofessional Relations/
69. Interdisciplinary Communication/
70. ((organi#ation* adj2 (structur* or form* or function* or determinant* or factors or environme nt* or process* or culture*)) and (outcome? or perform* or satisf* or efficien* or effective* or equ* or growth or develop* or justice or quality or culture* or manage* or leader*)).tw.
71. exp *psychology, industrial/ or *absenteeism/ or *efficiency/ or *job satisfaction/ or *"task performance and analysis"/ or *"time and motion studies"/ or *work simplification/ or *time management/ or *vocational guidance/
72. Decision Support Systems, Clinical/
73. Workflow/
74. or/48-73
75. Nurses/
76. Physicians/
77. (nurse* or physician* or doctor*).tw.
78. Medical Staff/
79. Medical Staff, Hospital/ or Nursing Staff, Hospital/ or Intensive Care Units/
80. or/75-79
81. 47 and 74 and 80
82. limit 81 to (english language and humans and yr="1995 -Current")

Medline Search Strategy: Interventions to improve situational awareness

1 Patient Safety/ (6742)

2 patient safety.tw. (13735)

3 1 or 2 (18307)

4 "Situation* Awareness".tw. (488)

5 3 and 4 (69)

6 limit 5 to (english language and humans and yr="1995 -Current")

For peer review only

Appendix 3: Data extraction template 2

Study/Paper	
Authors, title, date, source	
Purpose	
Design/Methods	
Participants/Setting	
Data type	
Theoretical framework	
Quality Assessment	
Formal study finding	

**Theory Area 1 – Properties of the monitoring system in EWS**
**What are the technical characteristics of the monitoring system?**
**What are the policies/processes associated with the monitoring system?**
**What are roles and responsibilities of the monitoring system?**
**What are the skills and resources needed to enact the monitoring system?**
**How often is the monitoring system applied?**
**How effective is the monitoring system?**
**What is the mechanisms (implicit or explicit) by which the monitoring system has its effects?**
**What actions are necessary to ensure the effectiveness of the monitoring system?**
**What is the relationship of the monitoring system and the context?**
**What is the relationship of the monitoring system to the recording system?**
Is the evidence provided in this theory area good and relevant enough to be included in the synthesis?

**Theory Area 2 – Properties of the recording system in EWS**
**What are the technical characteristics of the recording system?**

For peer review only

**What are the policies/processes associated with the recording system?**
**What are the roles and responsibilities of the recording system?**
**How effective is the recording system?**
**What are the skills and resources needed to enact the recording system?**
**What is the mechanism (implicit or explicit) by which the recording system has its effects?**
**What actions are necessary to ensure the effectiveness of the recording system?**
**What is the relationship between the recording system and the context?**
**What is the relationship between the recording system, the monitoring and detection system?**
Is the evidence provided in this theory area good and relevant enough to be included in the synthesis?

**Theory Area 3 – Properties of the detection/trigger system in EWS**
**What are the technical characteristics of the detection/trigger system?**
**What are the policies/processes associated with the detection/trigger system?**
**What are the roles and responsibilities involved in the detection/trigger system?**
**What are the skills and resources needed to enact the detection system?**
**How effective is the trigger system?**
**What is the mechanism (implicit or explicit) by which the detection/trigger system has its effects?**
**What actions are necessary to ensure the effectiveness of the detection/trigger system?**
**What is the relationship between the trigger system and the context?**
**What is the relationship between the trigger system and the activation system?**
Is the evidence provided in this theory area good and relevant enough to be included in the synthesis?

**Theory Area 4 – Properties of the activation system in EWS**
**What are the technical characteristics of the activation system?**

For peer review only

**What are the policies/processes associated with the activation system?**
**What are the roles and responsibilities involved in the activation system?**
**What are the skills and resources needed to enact the activation system?**
**How effective is the activation system?**
**What is the mechanism (implicit or explicit) by which the activation system has its effects?**
**What actions are necessary to ensure the effectiveness of the activation system?**
**What is the relationship of the activation system and the context?**
**What is the relationship between the activation system and the monitoring, recording and trigger system?**
Is the evidence provided in this theory area good and relevant enough to be included in the synthesis?

**Theory Area 5 – Properties of family involvement in EWS**
**At what stage in the PEWS process are families involved?**
**What are the roles and responsibilities involved in ensuring family involvement?**
**How effective is family involvement in the EWS process?**
**What are the mechanisms by which families are involved in EWS?**
**How effective are mechanisms for involving families in EWS?**
**What are the benefits and dis-benefits of family involvement?**
Is the evidence provided in this theory area good and relevant enough to be included in the synthesis?

**Theory Area 6 – Properties of chart design in EWS**
**What are the optimal properties of chart design to ensure effective monitoring?**
**What are the optimal properties of chart design to ensure effective recording?**
**What are the optimal properties of chart design to ensure effective detection?**

For peer review only

Is the evidence provided in this theory area good and relevant enough to be included in the synthesis?

**Theory Area 7– Properties of implementation and normalisation in EWS**
**What are the barriers to implementation and normalisation of EWS?**
**What actions are necessary to overcome barriers to the implementation and normalisation of EWS?**
**What actions are effective in overcoming barriers to the implementation and normalisation of EWS?**
**What are the mechanisms by which effective actions in overcoming barriers to implementation and normalisation of EWS have their effects?**
**What are the key pillars of an effective reflexive monitoring system?**
Is the evidence provided in this theory area good and relevant enough to be included in the synthesis?

For peer review only

For peer review only

PRISMA 2009 Checklist

Section/topic	#	Checklist item	Reported on page #
**TITLE**			
Title	1	Identify the report as a systematic review, meta-analysis, or both.	1
**ABSTRACT**			
Structured summary	2	Provide a structured summary including, as applicable: background; objectives; data sources; study eligibility criteria, participants, and interventions; study appraisal and synthesis methods; results; limitations; conclusions and implications of key findings; systematic review registration number.	2-3
**INTRODUCTION**			
Rationale	3	Describe the rationale for the review in the context of what is already known.	3-4
Objectives	4	Provide an explicit statement of questions being addressed with reference to participants, interventions, comparisons, outcomes, and study design (PICOS).	n/a
**METHODS**			
Protocol and registration	5	Indicate if a review protocol exists, if and where it can be accessed (e.g., Web address), and, if available, provide registration information including registration number.	2
Eligibility criteria	6	Specify study characteristics (e.g., PICOS, length of follow-up) and report characteristics (e.g., years considered, language, publication status) used as criteria for eligibility, giving rationale.	n/a
Information sources	7	Describe all information sources (e.g., databases with dates of coverage, contact with study authors to identify additional studies) in the search and date last searched.	6
Search	8	Present full electronic search strategy for at least one database, including any limits used, such that it could be repeated.	7, appendix 2
Study selection	9	State the process for selecting studies (i.e., screening, eligibility, included in systematic review, and, if applicable, included in the meta-analysis).	8
Data collection process	10	Describe method of data extraction from reports (e.g., piloted forms, independently, in duplicate) and any processes for obtaining and confirming data from investigators.	8, appendix 1& 3
Data items	11	List and define all variables for which data were sought (e.g., PICOS, funding sources) and any assumptions and simplifications made.	n/a
Risk of bias in individual studies	12	Describe methods used for assessing risk of bias of individual studies (including specification of whether this was done at the study or outcome level), and how this information is to be used in any data synthesis.	n/a
Summary measures	13	State the principal summary measures (e.g., risk ratio, difference in means).	n/a
Synthesis of results	14	Describe the methods of handling data and combining results of studies, if done, including measures of consistency (e.g., I^2) for each meta-analysis.	5-6, 8

For peer review only - http://bmjopen.bmj.com/site/about/guidelines.xhtml

PRISMA 2009 Checklist

Section/topic	#	Checklist item	Reported on page #
Risk of bias across studies	15	Specify any assessment of risk of bias that may affect the cumulative evidence (e.g., publication bias, selective reporting within studies).	n/a
Additional analyses	16	Describe methods of additional analyses (e.g., sensitivity or subgroup analyses, meta-regression), if done, indicating which were pre-specified.	8-9, table 1
**RESULTS**			
Study selection	17	Give numbers of studies screened, assessed for eligibility, and included in the review, with reasons for exclusions at each stage, ideally with a flow diagram.	8, table 2
Study characteristics	18	For each study, present characteristics for which data were extracted (e.g., study size, PICOS, follow-up period) and provide the citations.	Table 3, 4 and 5
Risk of bias within studies	19	Present data on risk of bias of each study and, if available, any outcome level assessment (see item 12).	n/a
Results of individual studies	20	For all outcomes considered (benefits or harms), present, for each study: (a) simple summary data for each intervention group (b) effect estimates and confidence intervals, ideally with a forest plot.	n/a
Synthesis of results	21	Present results of each meta-analysis done, including confidence intervals and measures of consistency.	n/a
Risk of bias across studies	22	Present results of any assessment of risk of bias across studies (see Item 15).	n/a
Additional analysis	23	Give results of additional analyses, if done (e.g., sensitivity or subgroup analyses, meta-regression [see Item 16]).	8-9, box 1
**DISCUSSION**			
Summary of evidence	24	Summarize the main findings including the strength of evidence for each main outcome; consider their relevance to key groups (e.g., healthcare providers, users, and policy makers).	15-16
Limitations	25	Discuss limitations at study and outcome level (e.g., risk of bias), and at review-level (e.g., incomplete retrieval of identified research, reporting bias).	3
Conclusions	26	Provide a general interpretation of the results in the context of other evidence, and implications for future research.	15-16
**FUNDING**			
Funding	27	Describe sources of funding for the systematic review and other support (e.g., supply of data); role of funders for the systematic review.	3

From: Moher D, Liberati A, Tetzlaff J, Altman DG, The PRISMA Group (2009). Preferred Reporting Items for Systematic Reviews and Meta-Analyses: The PRISMA Statement. PLoS Med 6(7): e1000097. doi:10.1371/journal.pmed1000097

For more information, visit: **www.prisma-statement.org**.

For peer review only - http://bmjopen.bmj.com/site/about/guidelines.xhtml

BMJ Open

Optimising Paediatric Afferent Component Early Warning Systems: A hermeneutic systematic literature review and model development

Journal:	*BMJ Open*
Manuscript ID	bmjopen-2018-028796.R2
Article Type:	Original research
Date Submitted by the Author:	11-Oct-2019
Complete List of Authors:	Jacob, Nina; Cardiff University, Centre for Trials Research<br>Moriarty, Yvonne; Cardiff University, Centre for Trials Research<br>Lloyd, Amy; Cardiff University, Centre for Trials Research<br>Mann, Mala; Cardiff University, University Library Services<br>Tume, Lyvonne N.; University of the West of England Bristol,<br>Sefton, G.; Alder Hey Childrens NHS Fdn Trust,<br>Powell, Colin; Children's Hospital for Wales, Paediatrics<br>Roland, Damian; Paediatric Emergency Medicine Leicester Academic (PEMLA) Group, Emergency Department; University of Leicester Department of Health Sciences, SAPPHIRE group<br>Trubey, Robert ; Cardiff University, Centre for Trials Research<br>Hood, Kerenza; Cardiff University,<br>Allen, Davina; Cardiff University
**Primary Subject Heading**:	Paediatrics
Secondary Subject Heading:	Research methods
Keywords:	STATISTICS & RESEARCH METHODS, PAEDIATRICS, QUALITATIVE RESEARCH

SCHOLARONE™
Manuscripts

Optimising Paediatric Afferent Component Early Warning Systems: A hermeneutic systematic literature review and model development

Authors: Nina Jacob[1†], Yvonne Moriarty[1], Amy Lloyd [1], Mala Mann[2], Lyvonne Tume[3], Gerri Sefton [4], Colin Powell[5, 6], Damian Roland [7,8], Robert Trubey[1], Kerenza Hood[1], Davina Allen[9]

1 Centre for Trials Research, Cardiff University, Heath Park, Cardiff, UK

2 Specialist Unit for Review Evidence (SURE), Cardiff University, Heath Park, Cardiff, UK

3 Faculty of Health and Applied Sciences, University of the West of England Bristol, Bristol, UK

4 Paediatric Intensive Care Unit, Alder Hey Children's NHS Foundation Trust, Liverpool, UK

5 Department of Child Health, Children's Hospital for Wales, Division of Population Medicine School of Medicine, Cardiff University, Cardiff, UK,

6 Department of Pediatric Emergency Medicine, Sidra Medicine, Doha, Qatar

7 Paediatric Emergency Medicine Leicester Academic (PEMLA) Group, Emergency Department, Infirmary Square, Leicester, UK

8 SAPPHIRE Group, Health Sciences, Leicester University, Leicester, UK

9 School of Healthcare Sciences, Cardiff University, Eastgate House, Cardiff, UK

Corresponding author:

[†]Dr Nina Jacob, Centre for Trials Research, Cardiff University, Heath Park, Cardiff, UK. JacobN@cardiff.ac.uk

Keywords: PEWS, track and trigger scores, early warning scores, clinical deterioration, children, systematic review

Word Count: 4459

For peer review only

OPTIMISING PAEDIATRIC AFFERENT COMPONENT EARLY WARNING SYSTEMS: A HERMENEUTIC SYSTEMATIC LITERATURE REVIEW AND MODEL DEVELOPMENT

ABSTRACT

Objective: To identify the core components of successful early warning systems for detecting and initiating action in response to clinical deterioration in paediatric in-patients.

Methods: A hermeneutic systematic literature review informed by Translational Mobilisation Theory and Normalisation Process Theory was used to synthesise 82 studies of paediatric and adult early warning systems and interventions to support the detection of clinical deterioration and escalation of care. This method, which is designed to develop understanding, enabled the development of a propositional model of an optimal afferent component early warning system.

Results: Detecting deterioration and initiating action in response to clinical deterioration in paediatric in-patients involves several challenges, and the potential failure points in early warning systems are well documented. Track and Trigger Tools (TTTs) are commonly used and have value in supporting key mechanisms of action but depend on certain preconditions for successful integration into practice. Several supplementary interventions have been proposed to improve the effectiveness of early warning systems but there is limited evidence to recommend their wider use, due to the weight and quality of the evidence; the extent to which systems are conditioned by the local clinical context; and the need to attend to system component relationships, which do not work in isolation. While it was not possible to make empirical recommendations for practice, the review methodology generated theoretical inferences about the core components of an optimal system for early warning systems. These are presented as a propositional model conceptualised as three sub-systems: detection, preparation and action.

Conclusions: There is a growing consensus of the need to think beyond TTTs in improving action to detect and respond to clinical deterioration. Clinical teams

For peer review only - http://bmjopen.bmj.com/site/about/guidelines.xhtml

wishing to improve early warning systems can use the model to consider systematically the constellation of factors necessary to support detection, preparation and action and consider how these arrangements can be implemented in their local context.

STRENGTHS AND WEAKNESSES OF THE STUDY

- The literature in this field is heterogeneous and better at identifying system weakness than it is effective improvement interventions. By deploying social theories and a hermeneutic review methodology it was possible to develop a propositional model of the core components of an afferent component paediatric early warning system.
- The model is derived from logical inferences drawing on the overall evidence synthesis, social theories and clinical expertise, rather than strong empirical evidence of single intervention effectiveness.
- There is a growing consensus of the need to take a whole systems approach to improve the detection and response to deterioration in the in-patient paediatric population and this paper offers an evidence-based framework for this purpose.

PROSPERO registration number CRD42015015326.

INTRODUCTION

Failure to recognise and act upon signs of clinical deterioration in the hospitalised child is an acknowledged safety concern (1). Track and Trigger Tools (TTT) are a common response to this problem. A TTT consists of sequential recording and monitoring of physiological, clinical and observational data. When a certain score or trigger is reached then a clinical action should occur including, but not limited to, altered frequency of observation, senior review or more appropriate treatment or management. Tools may be paper based or electronic and monitoring can be automated or undertaken manually by staff.

Despite the growing use of TTTs there is limited evidence of their effectiveness as a single intervention in reducing mortality or arrest rates in hospitalised children (2,3).

For peer review only - http://bmjopen.bmj.com/site/about/guidelines.xhtml

Results from the largest international cluster randomised control trial of a TTT (the Beside PEWS) did not support TTT use to reduce mortality, and highlighted the multifactorial mechanisms involved in detecting and initiating action in response to deterioration (4). These findings lend further weight to a developing consensus about the need to look beyond TTTs to the impact of wider system factors on detecting and responding to deterioration in the in-patient paediatric population (2,5–9). This paper reports on a theoretically informed systematic hermeneutic literature review (10) to identify the core components and mechanisms of action of successful afferent component early warning systems in paediatric hospitals and is one of three linked reviews undertaken as part of a wider UK study commissioned to develop and evaluate an evidence-based paediatric warning system (3,11). It addressed the following question:

What socio-material and contextual factors are associated with successful or unsuccessful Paediatric Early Warning Systems (with or without TTTs)?

METHOD

DESIGN

We performed a hermeneutic systematic review of the relevant literature. A hermeneutic systematic review is an iterative process, integrating analysis and interpretation of evidence with literature searching and is designed to develop a better understanding of the field (10). The popularity of the method is growing in health services research where it has value in generating insights from heterogeneous literatures that cannot be synthesised through standard review methodology (12) and would otherwise produce inconclusive findings (see (9). The purpose of the review was not exhaustive aggregation of evidence, but to develop an understanding of the social, material and contextual factors associated with successful or unsuccessful paediatric early warning systems.

THEORETICAL FRAMEWORK

Data extraction and interpretation was informed by Translational Mobilisation Theory (TMT) (13,14) and Normalisation Process Theory (NPT) (15,16). TMT is a practice theory which explains how goal-oriented collaborative activity is mobilised in

For peer review only - http://bmjopen.bmj.com/site/about/guidelines.xhtml

For peer review only

unpredictable environments [Box 1] and how the relevant mechanisms of action are conditioned by the local context. It is well suited for understanding early warning systems which require the organisation of action in evolving conditions, in a variety of clinical environments, with different teams, skill mixes, resources, structures, and technologies. NPT shares the same domain assumptions as TMT and is concerned with 'how and why things become, or do not become, routine and normal components of everyday work' (15,16), directing attention to the preconditions necessary for successful implementation of interventions. The theoretical framework informed our data extraction strategy, interpretation of the evidence, and the development of a propositional model of an optimal paediatric early warning system.

FOCUS OF THE REVIEW

The literature in this field identifies four integrated components which work together to provide a safety system for at risk patients: (1) the afferent component which detects deterioration and triggers timely and appropriate action, (2) the efferent component which consists of the people and resources providing a response; (3) a process improvement component, which includes system auditing and monitoring and (4) an administrative component focusing on organisational leadership and education required to implement and sustain the system (17). Our focus was limited to the afferent components of the system.

STAGES OF THE REVIEW

STAGE 1: SCOPING THE LITERATURE

Literature was identified through a recent scoping review (7), team members' knowledge of the field, hand searches and snowball sampling techniques. The purpose was to (1) inform our review question and eligibility criteria and (2) identify emerging themes and issues. Whilst we drew on several reviews of the literature (5, 12-14) we always consulted original papers. Data were extracted using Data Extraction Template 1 (Appendix 1) and analysed to produce a provisional conceptual model of the core components of paediatric early warning systems. Additional themes of relevance were identified: family involvement, situational awareness (SA), structured handover, observations and monitoring and the impact of electronic systems and new technologies.

For peer review only - http://bmjopen.bmj.com/site/about/guidelines.xhtml

STAGE 2: SEARCHING FOR THE EVIDENCE

We undertook systematic searches of the paediatric and adult early warning system literature (the goals and mechanisms of collective action in detection and rescue trajectories are the same). For the adult literature we used the same search strategies but added a qualitative filter to limit the scope to studies most likely to yield the level of socio-material and contextual detail of value to the review. Literature informing additional areas of interest was located through a combination of systematic and hand searches. Systematic searches (Searches 2 and 3) were undertaken in areas where we anticipated locating evidence of the effectiveness of specific interventions to strengthen early warning systems. Theory driven searches reflected the conceptual requirements of the model development.

SYSTEMATIC SEARCHES

A systematic search was initially conducted across a range of databases from 1995 to September 2016 to identify relevant studies on the Paediatric Early Warning System literature. This search was updated to cover literature from September 2016 to May 2018. An additional three systematic searches were conducted from 1995 to September 2016 to identify supplementary papers to aid in developing understanding on the PEWS literature:

1. Adult Early Warning Systems (EWS)
2. Interventions to improve situational awareness.
3. Structured communication tools for handover and handoff

Detailed information on the search methodology can be found in Appendix 2. Grey literature was excluded in order to keep the review manageable

THEORY DRIVEN SEARCHES

Additional theory-driven searches were conducted in the following areas:

1. Family involvement
2. Observations and monitoring
3. The impact of electronic systems

For peer review only - http://bmjopen.bmj.com/site/about/guidelines.xhtml

These were a combination of exploratory, computerised, snowball, and hand searches. As the analysis progressed, we continued to review new literature on early warning systems as this was published.

SCREENING

After removing duplicates 5284 references were identified for screening. A modified PRISMA flow diagram is provided [Figure 1]. Papers were screened by title to assess eligibility and then by full text to assess relevance for data extraction. The PEWS and adult EWS searches were screened by two researchers, searches 2 and 3 were screened by the lead reviewer.

STAGE 3: DATA EXTRACTION AND APPRAISAL

Data Extraction Template 2 [Appendix 3] was applied to all papers included in the review. As is typical of reviews of this kind, evidential fragments and partial lines of inquiry formed the unit of analysis rather than whole papers (18). These fragments were quality assessed according to the contribution they made to the developing analysis rather than assessing the paper as whole through the use of formal appraisal tools. Data extraction and quality appraisal were undertaken concurrently and double-checked by a second reviewer.

STAGE 4: DEVELOPING A PROPOSITIONAL MODEL

A propositional model was developed specifying the core ingredients of a paediatric early warning system [Table 1]. It comprises of logical inferences derived from the theoretical framework and evidence synthesis, informed by clinical experts on the team. Iterations of the model were developed in collaboration with clinical colleagues. A series of face-to-face meetings were conducted to review structure, wording and applicability to clinical practice.

PATIENT AND PUBLIC INVOLVEMENT

This review was conducted as part of a larger mixed methods study (ISRCTN94228292), which used a formal, facilitated parental advisory group. The

For peer review only

For peer review only - http://bmjopen.bmj.com/site/about/guidelines.xhtml

group comprised parents of children who had experienced an unexpected adverse event in a paediatric unit and provided input which helped to shape the broader research questions and wider contextual factors to consider, specifically within the family involvement element of the system. The results of the review will be disseminated to parents through this group.

RESULTS

INCLUDED STUDIES

Eighty-two papers were included in the review. Forty-six papers focussed on TTT implementation and use in paediatric and adult contexts (twenty-four from the paediatric search and the remaining twenty-two from the adult focussed search); the remaining thirty-six papers contributed supplementary data on factors related to the wider warning system. See Table 2 for a detailed breakdown of this process. No studies were located that adopted a whole systems approach to detecting and responding to deterioration.

ANALYSIS

In TMT the primary unit of analysis is the 'project', which defines the social and material actors (people, materials, technologies) and their relationships involved in achieving a particular goal. The goals of the afferent paediatric warning system are first, that the child is identified as at risk and a vital signs monitoring regime instigated; second, that evidence of deterioration is identified through monitoring and categorised as such and; third, that timely and appropriate action is initiated in response to deterioration. Our analysis of the literature suggests that three sub-systems within the afferent component of early warning systems support these processes: the *detection* of signs deterioration; the *preparation* needed to ensure teams are ready to act when deterioration is detected; and the initiation of timely *action*. Whilst we have focussed on the afferent component, it is important to remember that all elements of the overall safety system (efferent component, process improvement and administrative arm) need to be working in concert in order to maintain an optimal

For peer review only - http://bmjopen.bmj.com/site/about/guidelines.xhtml

For peer review only

paediatric early warning system. In the next section we report on the literature in relation to each subsystem.

DETECTION

The goal of the detection sub-system is to recognise early signs of deterioration, so the child becomes the focus of further clinical attention. This requires first, that the child is identified as at risk and a vital sign monitoring regime instigated and second, that the child is identified as showing signs of deterioration.

Despite widespread use the evidence on TTT effectiveness in predicting adverse outcomes in hospitalised children is weak (3). Many TTT have only been validated retrospectively and post predictive value were generally low. Studies reporting significant decreases in cardiac arrest calls or mortality had methodological concerns. The literature does suggest that TTTs have value in supporting process mechanisms in the detection sub-system. Vital signs monitoring is undertaken on all hospital in-patients and, like other high-volume routine activity, is often delegated to junior staff (19-38) who may not have sufficient skills to interpret results (21,22,37). TTTs have value in mitigating these risks: by specifying physiological thresholds that indicate deterioration they take knowledge to the bedside and act as prompts to action (19,39) which can lead to a more systematic and frequent approach to monitoring and improved detection of deterioration (40,41).

TTT's effectiveness in fulfilling these functions depends on certain preconditions. The review highlighted that TTT use was impacted by the availability of appropriate and functioning equipment (22,27,29,34,39,42–46), (in)adequate staffing and night time pressures (22,26,29,30,37,40,42-44,47-52) and an appropriately skilled workforce (26,28,36,43,49,50,53-57). On this latter point, while several papers report on education packages to improve the detection of deterioration, the evidence is not robust enough to recommend specific programs (23–25,28,30,35,55,58–60). There were also times whereby nursing staff prioritised sleep over waking a patient to take vital signs (46,61).

TTTs are also used differently depending on the experience of the user. For juniors, they provide a methodology and structure for monitoring clinical instability and identifying deterioration, whereas more experienced staff reportedly use TTTs as

For peer review only - http://bmjopen.bmj.com/site/about/guidelines.xhtml

confirmatory technologies (19-32). The importance of professional intuition in detecting deterioration is extensively reported across the literature (19-22,26,27,29,31,32,36-40,42,43,45,46,48,50-53,60,62-68) and several authors recommend the inclusion of 'staff concern' in tool criteria (26,48,51,57). This is important; TTTs may be of less value in patients with chronic conditions because of altered normal physiology or where subtle changes are difficult to detect (64). It is also the case that TTTs are implemented in contexts governed by competing organisational logics which impact on their value and use (43,54,58). For example, Iddrisu et al. (57) show TTTs have limited value immediately after surgery because acceptable vital signs parameters are different in the immediate post-operative period.

There is growing interest in the literature in strategies that facilitate patient and relative involvement in the early detection of deterioration (69,70). Healthcare professionals depend on families to explain their child's normal physiological baseline and identify subtle changes in their child's condition but this information is not always systematically obtained (71,72). Some authors propose family involvement in interdisciplinary rounds[1] (73), but this requires parents to have detailed information about the signs and symptoms they should be attending to (72) and as yet there is little evidence on effective strategies for how they might be involved in the detection of deterioration (73).

While much of the literature reports on intermittent manual vital signs monitoring and paper-based recording systems, across the developed world there is a growing use of electronic technologies, which have important implications for the wider detection sub-system (74). We considered a number of evaluations of new technologies which indicated that electronic vital signs recording is associated with a number of positive outcomes, particularly timeliness and accuracy, when compared with paper-based systems (75,76). They can provide prompts or alerts for monitoring (77–79), which facilitates better recognition of deterioration and is associated with a reduction in mortality (78,80). These studies tend to evaluate new technologies in isolation, however, and do not engage with the literature highlighting alarm fatigue which is known to mitigate effectiveness over time or concerns about over burdening staff with alerts (81–83). Moreover, the successful implementation of new technologies is

[1] This is an editorial paper

For peer review only - http://bmjopen.bmj.com/site/about/guidelines.xhtml

conditioned by the local context. For instance, where manual input into an electronic device is required, access to computers is an essential precondition. When computers were not available staff 'batch' the collection of vital signs before data entry, thereby delaying the timely detection of deterioration (27,45,84). In another study where the electronic system was found to be cumbersome and separated the collection and entry of data from the review of vital signs, verbal reports were favoured to ensure timely communication of information (85). See Table 3 for a summary of the evidence reported.

PREPARATION

Detecting and responding to deterioration involves the coordination of action in conditions of uncertainty and competing priorities. The goal of the 'Preparation' sub-system is to ensure the clinical team are ready to act in the event of evidence of deterioration and is reflected in the growing interest in in the literature on structures to facilitate team situational awareness, group decisions and planning (62).

TTTs have been found to support situational awareness. Their use enabled clinicians to have a 'birds-eye' view over all admitted patients on a ward as well as encouraging staff to consider projected acuity levels of the ward (86). A number of studies also report on 'huddles' in facilitating situational awareness (32,65,87,88). A huddle is a multidisciplinary event scheduled at pre-determined times where members discuss specific risk factors around deterioration and develop mitigation plans. One study combined the introduction of huddles with a "watchstander", a role fulfilled by a charge nurse or senior resident, whose primary function is to know patients at high risk for deterioration (88). These initiatives were associated with a near fifty per cent reduction in transfers from acute to intensive care determined to be unrecognized situation awareness events. A further strategy identified by Goldenhar et al. describes the use of the 'watcher' category to designate a patient as at risk where staff have a 'gut feeling' deterioration is likely (87). A recent study used the category of 'watcher' to create a bundle of expectations to standardize communication and contingency planning. Once a patient was labelled 'a watcher' a series of five specific tasks, such as documentation of physician awareness of watcher status and that the family had been notified of the change in the patient's status, needed to completed within two hours (89).

For peer review only - http://bmjopen.bmj.com/site/about/guidelines.xhtml

Handovers are integral to clinical communication and contribute to situational awareness. The extensive literature on handover indicates that information sharing can be of variable quality (47,54,90) and there is growing evidence that structured approaches improve this (30,47,54,63,87,90–94). Ranging from a checklist system (91,93) to a cognitive aid developed through consensus (23,94), most of the published interventions are variations of the SBAR (Situation, Background, Assessment, and Recommendation) tool (54,90–92). While effective handover depends on communicative forms that extend beyond the information transfer that is typically the focus of structured handover tools (90), in the context of early warning systems a lack of standardisation allows greater margin for individualistic practices and difficulties accessing complementary knowledge and establishing shared understandings (47).

There is also a literature on the use of common information spaces – such as white boards – in facilitating situational awareness in the healthcare team (23,33,47,53,55,58,67). These should be in a visible location and colour-coded to correspond with the TTT score, where relevant (47,55,58). Electronic systems automate this information and allow information to be reviewed remotely. However, they disconnect vital signs data from the patient and hence other indicators of clinical status and access to data is contingent upon the availability of computers (27,45,47,84,95).

The literature indicates that situational awareness can be facilitated in different ways in different contexts and it is the relationship between system elements that is important (47). In their study on situational awareness in delivery suites, Mackintosh et al. discuss the three main supports for situational awareness - whiteboard, handover and coordinator role - and illustrate how these interacted in organisations with strong situational awareness compared to those with reduced levels. Crucially this 'interplay' between the different activities was highly context dependent; "the same supports used differently generate different outcomes" pg52 (47). See table 4 for a summary of the preparation evidence.

ACTION

The goal of the "Action" sub-system is to initiate appropriate action in response to evidence of deterioration. The literature suggests that mobilising action across professional boundaries/hierarchies is challenging, with differences in language

For peer review only - http://bmjopen.bmj.com/site/about/guidelines.xhtml

between doctors and nurses and power dynamics contributory factors (27,40,41,50,52,57,60,96). TTTs are in part a response to the challenges of communication in mobilising action in response to deterioration. By transforming a series of discrete observations into a summative indicator of deterioration – such as a score or a trigger – TTTs 'translate' and package the patient's status into a form that can be readily communicated enabling individual level clinical data to be synthesised, made sense of and shared (19-29,33,39,41,42,46,48,50,51,56,62,66,74,86). One study, however, found that TTT were regarded as a nursing tool and were therefore not valued by clinicians. Consequently, nurses encountered difficulties in summoning a response (46).

Several studies also report on the use of SBAR in this context. Like TTTs, SBAR translates information into a form that provides structure, consistency and predictability when presenting patient information. SBAR has been shown to help establish common language and expectations, minimising differences in training, experience and hierarchy and facilitating nurse-clinician communication. While several papers advocate combining SBAR with TTTs (23,25,27,30,35,45,50), none specifically evaluated SBAR use. Mackintosh et al. highlight that audit data suggests resistance to SBAR, with others cautioning that overextending SBAR use carries the risk of SBAR fatigue and attenuation of its effects (27).

Structured communication tools like TTTs and SBAR do not solve all the challenges of acting in response to evidence of deterioration. Barriers to action were widely reported in the literature where these tools were in place. These include: a general disinclination to seek help (19-22,25,27,29,31,36-39,42,48,50,51,56,64,67), concerns about appearing inadequate in front of colleagues (20,22,36,38,50,67), and failure of staff to invest in the escalation or calling criteria (21,22,49). A number of papers also reported negative attitudes to Rapid Response Team (RRT) or Medical Emergency Team (MET) use in the efferent component of safety systems. METs and RRTs operate outside the immediate medical team and create different issues in paediatric warning systems than when the escalation response is managed by the treating team. These include a reluctance to activate because of the perceived busyness of Paediatric Intensive Care Unit or medical staff (20,29,39,48,50,51), because previous expectations about an appropriate response were not met, or a sense that the situation

For peer review only - http://bmjopen.bmj.com/site/about/guidelines.xhtml

For peer review only

was under control (particularly when the physiological instability is in the area of expertise of the treating team) (22,29,31,38,42,50,52,64).

No literature reported on successful interventions to facilitate RRT use, but several propose strategies to support escalation where there was no designated response team in place in the efferent component. These includes informal peer-support, where inexperienced staff team up with more experienced staff (21,29,50,64,67); clear structures to support action and a supportive culture that does not penalise individual decision-making, including the use of a 'no false alarms' policy so staff are not deterred from escalating care (21,29,73, 36). Senior leadership is consistently identified as important (8,20-23,25,27,30,32,33,35,47,52,58,66,67); lack of support from superiors meant that staff are less likely to escalate and more likely to adhere to hierarchies within the current system (25,40,66). There is some evidence to suggest that any escalation policy should be linked to an administrative arm that reinforces the system, measures outcomes and works to ensure an effective system (27,30).

There is a small literature on family involvement in the Action Subsystem. Several studies report on Condition-Help, a program developed in the United States to support families to directly activate a RRT if they have concerns about their child's condition. Families are also becoming increasingly recognised as playing a key role in the activation of RRTs in Australia (97). Research has evaluated the appropriateness of calls that were made by patients or relatives (33,97–101) but has not considered why calls were *not* made (70). Involving family members in escalation demands vigilance, requiring them to take a proactive and interactive role with staff with potentially some degree of confrontation, particularly if challenging the appropriateness of decisions taken (73,97). Families need both cognitive and emotional resources to raise concerns that involve negotiating hierarchies and boundaries (35,70). The literature points to a degree of professional resistance to family involvement in activation, with reports of physician concern that their role would be undermined, that resources would be stretched with an increase in calls and that it might divert attention away from those in need (71,97,99,102,103) although these fears are not supported by the evidence (71,102,104). See Table 5 for a summary of the evidence relating to the action component of the model.

SYNTHESIS & MODEL DEVELOPMENT

For peer review only - http://bmjopen.bmj.com/site/about/guidelines.xhtml

The literature in this field is heterogeneous and stronger on the socio-material barriers to successful afferent component peadiatric early warning systems than it is on solutions. While a number of different single interventions have been proposed and some have been evaluated, there is limited evidence to recommend their use beyond the specific clinical contexts described in the papers. This reflects both the weight and quality of the evidence, the extent to which paediatric systems are conditioned by the local clinical context, and also the need to attend to the relationship between system components and inteventions which work in concert not in isolation. There is also a growing realisation in the quality improvement field that an intervention that has been successful in one context does not necessarily produce the same results elsewhere which cautions against a 'one size fits all' approach (105,106).

While it is not possible to make empirical recommendations for practice, a hermeneutic review methodology enabled the generation of theoretical inferences about the core components of an optimal paediatric early warning system. These model components are logical inferences derived from an overall synthesis of the evidence, informed by our theoretical framework and clinical expertise. These are presented as a propositional model conceptualised as three sub-systems: detection, preparation and action (see Table 1).

DISCUSSION

This paper reports on one of three linked reviews undertaken as part of a wider UK study commissioned to develop and evaluate an evidence-based national paediatric early warning system (3). Drawing on TMT and NPT we have synthesised and analysed the findings from the review to develop a propositional model to specify the core components of optimal afferent component paediatric early warning systems. While there is a growing consensus of the need to think beyond TTTs to consider the whole system, no frameworks exist to support such an approach. Clinical teams wishing to improve rescue trajectories should take a whole systems perspective focused on the constellation of factors necessary to support detection, preparation and action and consider how these relationships can be managed in their local setting. TTTs have value in paediatric early warning systems but they are not the sole solution and depend on certain preconditions for their use. An emerging literature highlights the importance of preparation and indicates that combinations of interventions may

For peer review only - http://bmjopen.bmj.com/site/about/guidelines.xhtml

facilitate situation awareness. Professional judgement is also important in detecting and acting on deterioration and the evidence points to the importance of a wider organisational culture that is supportive of this. Innovative approaches are needed to support family involvement in all aspects of paediatric early warning systems, which are sensitive to the cognitive and emotional resources this requires. System effectiveness requires attention to the socio-material relationships in the local context, senior support and leadership and continuous monitoring and evaluation. New technologies, such as moving from paper based to electronic TTTs, has important implications for all three sub-systems and critical consideration should be given to their wider impacts and the preconditions for their integration into practice.

LIMITATIONS OF THE REVIEW

The literature in this field is heterogeneous and better at identifying system weakness than it is effective improvement interventions. It was only by deploying social theories and a hermeneutic review methodology did it prove possible to develop a propositional model of the core components of an afferent component paediatric early warning system. This model is derived from logical inferences drawing on the overall evidence synthesis, social theories and clinical expertise, rather than strong empirical evidence of single intervention effectiveness. Consequently, there is a growing consensus of the need to take a whole systems approach to improve the detection and response to deterioration in the in-patient paediatric population.

CONCLUSION

Failure to recognise and act upon signs of deterioration is an acknowledged safety concern (1) and TTTs are a common response to this problem. There is, however, a growing recognition of the importance of wider system factors on the effectiveness of responses to deterioration (5) (7). We have reviewed a wide literature and analysed this using social theories to develop a propositional model of an optimal afferent component paediatric early warning system that can be used as a framework for paediatric units to evaluate their current practices and identify areas for improvement. TTT use should be driven by the extent to which teams think that they will help improve the effectiveness of their system as a whole.

For peer review only - http://bmjopen.bmj.com/site/about/guidelines.xhtml

For peer review only

ACKNOWLEDGEMENTS

The authors would like to acknowledge the contribution of Dr Heather Strange to the review. The authors would also like to extend their thanks to the parental advisory group who have helped shape the broader questions of the research study as well as offer guidance on wider contextual factors to consider within the overall paediatric early warning system.

CONTRIBUTORS

NJ: screening and review of papers; led the theoretical synthesis of the literature; contributed to model development; preparation and writing of manuscript (with DA). YM: screening and review of papers; contributed to model development; contributed to the drafting of the manuscript. AL: led the model development (with DA); contributed to the drafting of the manuscript. MM: conceived and led the systematic search strategies; review of manuscript. LT, GS, CP, DR: screening and review of papers; contributed clinical expertise; contributed to model development; contributed to the drafting of the manuscript. RT; screening and review of manuscript. KH: contributed to model development; contributed to the drafting of the manuscript. DA: conceived of and designed the review; led the theoretical framing and analysis; screening and review of papers; led the model development (with AL) and led the writing of the manuscript (with NJ).

COMPETING INTERESTS

None declared.

FUNDING

This study is funded by the National Institute for Health Research (NIHR) Health Services and Delivery Research (HS&DR) programme (12/178/17). The views and opinions expressed in this paper are those of the authors and not necessarily those of the NHS, the NIHR or the Department of Health.

DATA SHARING STATEMENT

For peer review only - http://bmjopen.bmj.com/site/about/guidelines.xhtml

All data relevant to the study are included in the article or uploaded as supplementary information.

For peer review only

For peer review only - http://bmjopen.bmj.com/site/about/guidelines.xhtml

REFERENCES

1. Subbe, C. P. and Welch JR. Failure to rescue: using rapid response systems to improve care of the deteriorating patient in hospital. J Patient Saf Risk Manag. 2013;19(1): 6-11.

2. Chapman SM, Wray J, Oulton K, Peters MJ. Systematic review of paediatric track and trigger systems for hospitalised children. Resuscitation. 2016;109:87–109.

3. Trubey R, Huang C, Lugg-widger F V, Hood K, Allen D, Edwards D, et al. Validity and effectiveness of paediatric early warning systems and track and trigger tools for identifying and reducing clinical deterioration in hospitalised children : a systematic review. BMJ Open. 2019;9:e022105.

4. Parshuram CS, Dryden-Palmer K, Farrell C, Gottesman R, Gray M, Hutchison JS, et al. Effect of a pediatric early warning system on all-cause mortality in Hospitalized pediatric patients: The epoch randomized clinical trial. JAMA - J Am Med Assoc. 2018;319(10):1002–12.

5. The Irish Paediatric Early Warning System (PEWS)(NCEC National Clinical Guideline No. 12). 2015. Department of Health Ireland.

6. Rihari-Thomas J, Newton PJ, Sibbritt D, Davidson PM. Rapid response systems: where we have come from and where we need to go? J Nurs Manag. 2018;26(1):1–2.

7. Lambert V, O'Shea M, Walshe C, Matthews A, Corbally M, O'Mathuna D, et al. A systematic literature review to support the development of a National Clinical Guideline – Paediatric Early Warning System (PEWS). 2014. [last accessed August 2019, http://health.gov.ie/wp-content/uploads/2014/03/PEWS-Sytematic-Literature-Review-Oct-2014.pdf]

8. Almblad AC, Siltberg P, Engvall G, Målqvist M. Implementation of Pediatric Early Warning Score; Adherence to Guidelines and Influence of Context. J Pediatr Nurs. 2018;38:33–9.

9. Lambert V, Matthews A, MacDonell R, Fitzsimons J. Paediatric early warning

For peer review only

For peer review only - http://bmjopen.bmj.com/site/about/guidelines.xhtml

systems for detecting and responding to clinical deterioration in children: A systematic review. BMJ Open. 2017;7:e014497.

10. Boell SK, Cecez-Kecmanovic D. A hermeneutic approach for conducting literature reviews and literature searches. Commun Assoc Inf Syst. 2014;34(12):257–86.

11. Thomas-Jones E, Lloyd A, Roland D, Sefton G, Tume L, Hood K, et al. A prospective, mixed-methods, before and after study to identify the evidence base for the core components of an effective Paediatric Early Warning System and the development of an implementation package containing those core recommendations for use in th. BMC Pediatr. 2018;18:244.

12. Greenhalgh T, A'Court C, Shaw S. Understanding heart failure; explaining telehealth - A hermeneutic systematic review. BMC Cardiovasc Disord. 2017;17(1):1–16.

13. Allen D, May C. Organizing Practice and Practicing Organization: An Outline of Translational Mobilization Theory. SAGE Open. 2017;7(2):35–43.

14. Allen D. Translational Mobilisation Theory: A new paradigm for understanding the organisational elements of nursing work. Int J Nurs Stud. 2018;79:36–42.

15. May C, Finch T. Implementing, embedding, and integrating practices: An outline of normalization process theory. Sociology. 2009;43(3):535–54.

16. May CR, Johnson M, Finch T. Implementation, context and complexity. Implement Sci. 2016;11(1):1–12.

17. Jagt EW van der. Improving Pediatric Survival from Resuscitation Events: The Role and Organization of Hospital-based Rapid Response Systems and Code Teams. Curr Pediatr Rev. 2013;9(2):158–74.

18. Pawson R. Evidence-Based Policy: A Realist Perspective. London: Sage; 2006.

19. Andrews T, Waterman H. Packaging: A grounded theory of how to report physiological deterioration effectively. J Adv Nurs. 2005;52(5):473–81.

20. Azzopardi P, Kinney S, Moulden A, Tibballs J. Attitudes and barriers to a Medical Emergency Team system at a tertiary paediatric hospital.

For peer review only - http://bmjopen.bmj.com/site/about/guidelines.xhtml

Resuscitation. 2011;82(2):167–74.

21. Bonafide CP, Roberts KE, Weirich CM, Paciotti B, Tibbetts KM, Keren R, et al. Beyond statistical prediction: Qualitative evaluation of the mechanisms by which pediatric early warning scores impact patient safety. J Hosp Med. 2013;8(5):248–53.

22. Braaten JS. Hospital System Barriers to Rapid Response Team Activation: A Cognitive Work Analysis. Am J Nurs. 2015;115(2):22–32.

23. Ennis L. Paediatric early warning scores on a children's ward: a quality improvement initiative. Nurs Child Young People. 2014;26(7):25–31.

24. Kaul M, Snethen J, Kelber ST, Zimmanck K, Maletta K, Meyer M. Implementation of the Bedside Paediatric Early Warning System (BedsidePEWS) for nurse identification of deteriorating patients. J Spec Pediatr Nurs. 2014;19(4):339–49.

25. Lobos AT, Fernandes R, Ramsay T MJ. Patient characteristics and disposition after pediatric medical emergency team (MET) activation: disposition depends on who activates the team. 2014;4:99–105.

26. McDonnell A, Tod A, Bray K, Bainbridge D, Adsetts D, Walters S. A before and after study assessing the impact of a new model for recognizing and responding to early signs of deterioration in an acute hospital. J Adv Nurs. 2013;69(1):41–52.

27. Mackintosh N, Rainey H, Sandall J. Understanding how rapid response systems may improve safety for the acutely ill patient: learning from the frontline. BMJ Qual Saf. 2012;21(2):135–44.

28. Monaghan A. Detecting and managing deterioration in children. Paediatr Care. 2005;17(1):32–5.

29. Pattison N, Eastham E. Critical care outreach referrals: A mixed-method investigative study of outcomes and experiences. Nurs Crit Care. 2012;17(2):71–82.

30. Pearson G, Duncan H. Early warning systems for identifying sick children. Paediatr Child Health (Oxford). 2011;21(5):230–3.

For peer review only - http://bmjopen.bmj.com/site/about/guidelines.xhtml

31. Shearer B, Marshall S, Buist MD, Finnigan M, Kitto S, Hore T, et al. What stops hospital clinical staff from following protocols? An analysis of the incidence and factors behind the failure of bedside clinical staff to activate the rapid response system in a multi-campus Australian metropolitan healthcare service. BMJ Qual Saf. 2012;21(7):569–75.

32. Stewart J, Carman M, Spegman A, Sabol VK. Evaluation of the effect of the modified early warning system on the nurse-led activation of the rapid response system. J Nurs Care Qual. 2014;29(3):223–9.

33. Van Voorhis KT, Willis TS. Implementing a Pediatric Rapid Response System to Improve Quality and Patient Safety. Pediatr Clin North Am. 2009;56(4):919–33.

34. Wheatley I. The nursing practice of taking level 1 patient observations. Intensive Crit Care Nurs. 2006;22(2):115–21.

35. Mccabe A, Duncan H, Heward Y. Paediatric early warning systems: where do we go from here? Paediatr Nurs. 2009;21(1):14–7.

36. Chua WL, Mackey S, Ng EKC, Liaw SY. Front line nurses' experiences with deteriorating ward patients: A qualitative study. Int Nurs Rev. 2013;60(4):501–9.

37. James J, Butler-Williams C, Hunt J, Cox H. Vital signs for vital people: An exploratory study into the role of the Healthcare Assistant in recognising, recording and responding to the acutely ill patient in the general ward setting. J Nurs Manag. 2010;18(5):548–55.

38. Mackintosh N, Humphrey C, Sandall J. The habitus of "rescue" and its significance for implementation of rapid response systems in acute health care. Soc Sci Med. 2014;120:233–42.

39. Endacott R, Westley M. Managing patients at risk of deterioration in rural hospitals: A qualitative study. Aust J Rural Health. 2006;14(6):275–9.

40. Lydon S, Byrne D, Offiah G, Gleeson L, O'Connor P. A mixed-methods investigation of health professionals' perceptions of a physiological track and trigger system. BMJ Qual Saf. 2016;25(9):688–95.

41. Sønning K, Nyrud C, Ravn IH. A survey of healthcare professionals '

For peer review only - http://bmjopen.bmj.com/site/about/guidelines.xhtml

experiences with the Paediatric Early Warning Score (PEWS). Nor J Clin Nurs / Sykepl Forsk. 2018;12:e64605.

42. Cioffi J, Salter C, Wilkes L, Vonu-Boriceanu O, Scott J. Clinicians' responses to abnormal vital signs in an emergency department. Aust Crit Care. 2006;19(2):66–72.

43. Endacott R, Kidd T, Chaboyer W, Edington J. Recognition and communication of patient deterioration in a regional hospital: A multi-methods study. Aust Crit Care. 2007;20(3):100–5.

44. Hands C, Reid E, Meredith P, Smith GB, Prytherch DR, Schmidt PE, et al. Patterns in the recording of vital signs and early warning scores: Compliance with a clinical escalation protocol. BMJ Qual Saf. 2013;22(9):719–26.

45. Watson A, Skipper C, Steury R, Walsh H, Levin A. Inpatient nursing care and early warning scores: A workflow mismatch. J Nurs Care Qual. 2014;29(3):215–22.

46. Jensen CS, Nielsen PB, Olesen HV, Kirkegaard H, Aagaard H. Pediatric Early Warning Score Systems, Nurses Perspective - A Focus Group Study. J Pediatr Nurs. 2018;41:e16–22.

47. MacKintosh N, Berridge EJ, Freeth D. Supporting structures for team situation awareness and decision making: Insights from four delivery suites. J Eval Clin Pract. 2009;15(1):46–54.

48. Cioffi J. Nurses' experiences of making decisions to call emergency assistance to their patients. J Adv Nurs. 2000;32(1):108–14.

49. Adelstein B-A, Piza MA, Nayyar V, Mudaliar Y, Klineberg PL, Rubin G. Rapid response systems: A prospective study of response times. J Crit Care. 2011;26(635):e11-8.

50. S Astroth K, M Woith W, Stapleton SJ, Degitz RJ, Jenkins SH. Qualitative exploration of nurses' decisions to activate rapid response teams. J Clin Nurs. 2013;22(19–20):2876–82.

51. Cioffi J. Recognition of patients who require emergency assistance: A descriptive study. Hear Lung J Acute Crit Care. 2000;29(4):262–8.

For peer review only

52. Mackintosh N, Watson K, Rance S, Sandall J. Value of a modified early obstetric warning system (MEOWS) in managing maternal complications in the peripartum period: An ethnographic study. BMJ Qual Saf. 2014;23(1):26–34.

53. Davies O, DeVita MA, Ayinla R, Perez X. Barriers to activation of the rapid response system. Resuscitation. 2014;85(11):1557–61.

54. McCrory MC, Aboumatar H, Custer JW, Yang CP, Hunt EA. "aBC-SBAR" training improves simulated critical patient hand-off by pediatric interns. Pediatr Emerg Care. 2012;28(6):538–43.

55. Randhawa S, Roberts-Turner R, Woronick K, DuVal J. Implementing and sustaining evidence-based nursing practice to reduce pediatric cardiopulmonary arrest. West J Nurs Res. 2011;33(3):443–56.

56. Salamonson Y, Heere B van, Everett B, Davidson P. Voices from the floor: Nurses' perceptions of the medical emergency team. Intensive Crit Care Nurs. 2006;22(3):138–43.

57. Mohammmed Iddrisu S, Hutchinson AF, Sungkar Y, Considine J. Nurses role in recognising and responding to clinical deterioration. J Clin Nurs. 2018;27:1920–30.

58. Demmel KM, Williams L, Flesch L. Implementation of the pediatric early warning scoring system on a pediatric Hematology/Oncology unit. J Pediatr Oncol Nurs. 2010;27(4):229–40.

59. McKay H, Mitchell IA, Sinn K, Mugridge H, Lafferty T, Van Leuvan C, et al. Effect of a multifaceted intervention on documentation of vital signs and staff communication regarding deteriorating paediatric patients. J Paediatr Child Health. 2013;49(1):48–56.

60. Radeschi G, Urso F, Campagna S, Berchialla P, Borga S, Mina A, et al. Factors affecting attitudes and barriers to a medical emergency team among nurses and medical doctors: A multi-centre survey. Resuscitation. 2015;88:92–8.

61. Hope J, Recio-Saucedo A, Fogg C, Griffiths P, Smith GB, Westwood G, et al. A fundamental conflict of care: Nurses' accounts of balancing patients' sleep with taking vital sign observations at night. J Clin Nurs. 2018;27(9–10):1860–

For peer review only - http://bmjopen.bmj.com/site/about/guidelines.xhtml

71.

62. de Groot JF, Damen N, Loos E De, Steeg L Van De, Koopmans L, Rosias P, et al. Implementing paediatric early warning scores systems in the Netherlands : future implications. BMC Pediatr. 2018;18:128.

63. Brady PW, Goldenhar LM. A qualitative study examining the influences on situation awareness and the identification, mitigation and escalation of recognised patient risk. BMJ Qual Saf. 2014;23(2):153–61.

64. Donohue LA, Endacott R. Track, trigger and teamwork: Communication of deterioration in acute medical and surgical wards. Intensive Crit Care Nurs. 2010;26(1):10–7.

65. Claussen D, Garner D, Crow B. Early warning signs and the EHR: At the intersection of technology and care. Nurs Manage. 2013;44(11):14–6.

66. Jones D, Baldwin I, McIntyre T, Story D, Mercer I, Miglic A, et al. Nurses' attitudes to a medical emergency team service in a teaching hospital. Qual Saf Heal Care. 2006;15(6):427–32.

67. Massey D, Chaboyer W, Aitken L. Nurses' perceptions of accessing a Medical Emergency Team: A qualitative study. Aust Crit Care. 2014;27(3):133–8.

68. Burns KA, Reber T, Theodore K, Welch B, Roy D, Siedlecki SL. Enhanced Early Warning System Impact on Nursing Practice: A phenomenological study. J Adv Nurs. 2018;74(5):1150–6.

69. Rainey H, Ehrich K, Mackintosh N, Sandall J. The role of patients and their relatives in "speaking up" about their own safety - a qualitative study of acute illness. Heal Expect. 2015;18(3):392–405.

70. Mackintosh, NJ, Davis, RE, Easter, A, Rayment-Jones, H, Sevdalis, N, Wilson, S, Adams, M, Sandall J. Interventions to increase patient and family involvement in escalation of care for acute life-threatening illness in community health and hospital settings (Protocol). Cochrane Database Syst Rev. 2017;(10):CD012829.

71. Paciotti, B, Roberts, KE, Tibbetts, KM, Weirich Paine, C, Keren, R, Barg, F.K., Holmes, J.H. and Bonafide CP. Physician attitudes toward family-activated medical emergency teams for hospitalized children. Jt Comm J Qual

For peer review only - http://bmjopen.bmj.com/site/about/guidelines.xhtml

Patient Saf. 2014;40(4):187–92.

72. Graedon J, Graedon T. Enlisting Families as Patient Safety Allies. Clin Pediatr Emerg Med. 2006;7(4):265–7.

73. Entwistle V. Nursing shortages and patient safety problems in hospital care: Is clinical monitoring by families part of the solution? Heal Expect. 2004;7(1):1–5.

74. Downey CL, Tahir W, Randell R, Brown JM, Jayne DG. Strengths and limitations of early warning scores : A systematic review and narrative synthesis. Int J Nurs Stud. 2017;76:106–19.

75. Mohammed M, Hayton R, Clements G, Smith G, Prytherch D. Improving accuracy and efficiency of early warning scores in acute care. Br J Nurs. 2009;18(1):18–24.

76. Sefton G, Lane S, Killen R, Black S, Lyon M, Ampah P, et al. Accuracy and Efficiency of Recording Pediatric Early Warning Scores Using an Electronic Physiological Surveillance System Compared With Traditional Paper-Based Documentation. Comput Inf Nurs. 2017;35(5):228–36.

77. Jones S, Mullally M, Ingleby S, Buist M, Bailey M, Eddleston JM. Bedside electronic capture of clinical observations and automated clinical alerts to improve compliance with an Early Warning Score protocol. Crit Care Resusc. 2011;13(2):83–8.

78. Schmidt PE, Meredith P, Prytherch DR, Watson D, Watson V, Killen RM, et al. Impact of introducing an electronic physiological surveillance system on hospital mortality. BMJ Qual Saf. 2015;24(1):10–20.

79. Bellomo R, Ackerman M, Bailey M, Beale R, Clancy G, Danesh V, et al. A controlled trial of electronic automated advisory vital signs monitoring in general hospital wards. Crit Care Med. 2012;40(8):2349–61.

80. Subbe CP, Duller B, Bellomo R. Effect of an automated notification system for deteriorating ward patients on clinical outcomes. Crit Care. 2017;21(1):1–9.

81. Bonafide CP, Zander M, Graham CS, Weirich Paine CM, Rock W, Rich A, et al. Video methods for evaluating physiologic monitor alarms and alarm responses. Biomed Instrum Technol. 2014;48(3):220–30.

For peer review only - http://bmjopen.bmj.com/site/about/guidelines.xhtml

82. Fagan K, Sabel A, Mehler PS, MacKenzie TD. Vital Sign Abnormalities, Rapid Response, and Adverse Outcomes in Hospitalized Patients. Am J Med Qual. 2012;27(6):480–6.

83. Bonafide CP, Localio AR, Holmes JH, Nadkarni VM, Stemler S, Macmurchy M, et al. Video Analysis of Factors Associated With Response Time to Physiologic Monitor Alarms in a Children's Hospital. 2019;171(6):524–31.

84. Wager KA, Schaffner MJ, Foulois B, Swanson Kazley A, Parker C, Walo H. Comparison of the quality and timeliness of vital signs data using three different data-entry devices. CIN - Comput Informatics Nurs. 2010;28(4):205–12.

85. Stevenson JE, Nilsson G. Nurses' perceptions of an electronic patient record from a patient safety perspective: A qualitative study. J Adv Nurs. 2012;68(3):667–76.

86. Vries A De, Draaisma JMT, Fuijkschot J. Clinician Perceptions of an Early Warning System on Patient Safety. Hosp Pediatr. 2017;7(10):579–86.

87. Goldenhar LM, Brady PW, Sutcliffe KM, Muething SE. Huddling for high reliability and situation awareness. BMJ Qual Saf. 2013;22(11):899–906.

88. Brady PW, Muething S, Kotagal U, Ashby M, Gallagher R, Hall D, et al. Improving Situation Awareness to Reduce Unrecognized Clinical Deterioration and Serious Safety Events. Pediatrics. 2013;131(1):e298–308.

89. Parker MW, Carroll M, Bolser B, Ballinger J, Brewington J, Campanella S. Implementation of a Communication Bundle for High-Risk Patients. Hosp Pediatr. 2017;7(9):523–9.

90. Pezzolesi C, Manser T, Schifano F, Kostrzewski A, Pickles J, Harriet N, et al. Human factors in clinical handover: Development and testing of a "handover performance tool" for doctors' shift handovers. Int J Qual Heal Care. 2013;25(1):58–65.

91. Abraham J, Kannampallil T, Patel B, Almoosa K, Patel VL. Ensuring patient safety in care transitions: an empirical evaluation of a Handoff Intervention Tool. AMIA Annu Symp Proc. 2012;2012:17–26.

92. Donahue M, Smith L, Dykes P, Fitzpatrick JJ. Phase 2 of the EMPOWER

For peer review only

project: enhancing communication for paraprofessionals. J Contin Educ Nurs. 2010;41(5):197–8.

93. Mullan PC, Macias CG, Hsu D, Alam S, Patel B. A novel briefing checklist at shift handoff in an emergency department improves situational awareness and safety event identification. Pediatr Emerg Care. 2015;31(4):231–8.

94. Weiss MJ, Bhanji F, Fontela PS, Razack SI. A preliminary study of the impact of a handover cognitive aid on clinical reasoning and information transfer. Med Educ. 2013;47(8):832–41.

95. Wong D, Bonnici T, Knight J, Morgan L, Coombes P, Watkinson P. SEND: A system for electronic notification and documentation of vital sign observations. BMC Med Inform Decis Mak. 2015;15:68.

96. Preece MHW, Hill A, Horswill MS, Karamatic R, Watson MO. Designing observation charts to optimize the detection of patient deterioriation: Reliance on the subjective preferences of healthcare professionals is not enough. Aust Crit Care. 2012;25(4):238–52.

97. Gill, F.N., Leslie, G.D., Marshall AP. Australian Critical Care Family initiated escalation of care for the deteriorating patient in hospital : Family centred care or just " box ticking ." Aust Crit Care. 2016;29(4):195–200.

98. Bogert S, Ferrell C, Rutledge DN. Experience with Family Activation Of Rapid Response Teams. Medsurg Nurs. 2010;19(4):215–22.

99. Dean BS, Decker MJ, Hupp D, Urbach AH, Lewis E, Benes-Stickle J. Condition HELP: a pediatric rapid response team triggered by patients and parents. J Healthc Qual. 2008;30(3):28–31.

100. Greenhouse PK, Kuzminsky B, Martin SC, Merryman T. Calling a condition H(elp). Am J Nurs. 2006;106(11):63–6.

101. Hueckel RM, Mericle JM, Frush K, Martin PL, Champagne MT. Implementation of condition help: Family teaching and evaluation of family understanding. J Nurs Care Qual. 2012;27(2):176–81.

102. Brady PW, Zix J, Brilli R, Wheeler DS, Griffith K, Giaccone MJ, et al. Developing and evaluating the success of a family activated medical emergency team: A quality improvement report. BMJ Qual Saf.

For peer review only - http://bmjopen.bmj.com/site/about/guidelines.xhtml

2015;24(3):203–11.

103. Gerdik C, Vallish RO, Miles K, Godwin SA, Wludyka PS, Panni MK. Successful implementation of a family and patient activated rapid response team in an adult level 1 trauma center. Resuscitation. 2010;81(12):1676–81.

104. Bavare, A.C Thomas, J.K Elliott, E.P Morgan A.C. Graf J. Family-Initiated Pediatric Rapid Response: Characteristics, Impetus, and Outcomes. J Healthc Qual. 2018;40(2):103–9.

105. Bion J, Richardson A, Hibbert P, Beer J, Abrusci T, McCutcheon M, et al. Matching Michigan: A 2-year stepped interventional programme to minimise central venous catheterblood stream infections in intensive care units in England. BMJ Qual Saf. 2013;22(2):110–23.

106. Dixon-Woods M, Leslie M, Tarrant C, Bion J. Explaining Matching Michigan: An ethnographic study of a patient safety program. Implement Sci. 2013; 8:70.

107. National Patient Safety Agency. Recognising and responding appropriately to early signs of deterioration in hospitalised patients London: NPSA; 2008.

108. Gilson RD. Situation awareness — special issue preface. Hum Factors J Hum Factors Ergon Soc. 1995;37(1):3–4.

TABLES

Box 1: Mechanisms of Translational Mobilisation and their Application to Rescue Trajectories (14)

Object formation - how people draw on the interpretative resources available to them within a strategic action field to create the objects of their practice. Enrolment into an escalation trajectory requires multiple examples of object formation beginning with construction of an individual as at risk of deterioration and a regime of vital signs monitoring instigated, through recognition that the patient's

For peer review only - http://bmjopen.bmj.com/site/about/guidelines.xhtml

physiological status is a cause for concern, to the identification of the patient as requiring a specific intervention. How this is achieved is highly dependent on the features of the local strategic action field.

Translation – the processes that enable practice objects to be shared and different understandings accommodated. It points to the actions necessary in order for a patient that is an object of concern for nursing staff to be translated into a clinical priority for the doctor and, if necessary, to be translated into the focus of intervention by the emergency response team.

Articulation - refers to the secondary work processes that align the actions, knowledge and resources necessary for the mobilization of projects of collective action. It is the work that makes the work, work. Responding to deterioration is time critical and articulation work is necessary to ensure the availability of resources and materials to support clinical management. This is not a mundane observation; catastrophic failures in patient safety are often attributed to the lack of functioning equipment (107) and the absence of monitoring equipment has been identified as a factor undermining the implementation of early warning track and trigger tools (48).Attending to articulation in rescue trajectories also underlines the temporal ordering of action and the mechanisms required to achieve this, directing improvement efforts towards the organization's escalation policy, for example.

Reflexive monitoring refers to the processes through which people collectively or individually appraise and review activity. In a distributed field of action, reflexive monitoring is the means through which members accomplish situational awareness (108) of an overall project. The importance of situation awareness in rescue trajectories is well recognised, but achieving this is challenging. Reflexive monitoring is conditioned by the wider institutional context which will include a multiplicity of informal and formal mechanisms designed for this purpose: nursing and medical handovers, the ward round, safety briefings. The form, frequency and effectiveness of these processes in supporting detecting and acting on deterioration would need to be taken into account in any improvement initiative.

For peer review only

Sensemaking - refers to the processes through which agents create order in conditions of complexity. It draws attention to how the material and discursive processes by which members organise their work, account for their actions, and construct the objects of their practice also give meaning and substance to the institutional components of strategic action fields that shape activity and condition future activity.

For peer review only

For peer review only

For peer review only - http://bmjopen.bmj.com/site/about/guidelines.xhtml

TABLE 1: PROPOSITIONAL MODEL

	Proposition	Conceptual requirements	Operationalisation	Tool and activities	Proposed effect of interventions
DETECTION	Detection of deterioration depends on collecting and interpreting information from children, through accurate, timely and appropriate monitoring of vital signs and relevant risk factors.	At a minimum, this requires:<br>• Staff are aware of which core and additional vital signs need to be monitored<br>• Staff are aware of the minimum frequency of observations required for the children in their care<br>• Staff are aware of the need to review the frequency of observations for children in their care<br>• Staff are aware of prior factors that increase children's risk of deterioration (e.g. premature birth)<br>• Staff are aware of additional clinical assessments required for children with prior risk factors<br>• Staff are aware of which additional clinical	At a minimum, this requires:<br>• Tasks are allocated to staff members with appropriate skills to conduct them<br>• Staff receive guidance about:<br>- Which core and additional vital signs need to be monitored<br>- Minimum frequency of observations required for children in their care<br>- Prior factors that increase children's risk of deterioration<br>- Which additional clinical assessments are appropriate for children in their care with prior risk factors<br>- Which members and grades of staff are responsible for monitoring and recording	• Skills training: accurate, timely and appropriate monitoring<br>• Focused education: recognising signs of deterioration<br>• Induction: staff aware of monitoring processes in a specific ward<br>• Observations policy<br>• Track and Trigger Tool<br>• Regular equipment checks	• Staff are aware of which core and additional indicators need to be monitored<br>• Staff are aware of the frequency of observations required for the children in their care<br>• Staff have appropriate skills to regularly monitor and accurately record key physiological parameters<br>• Staff are aware of roles and responsibilities<br>• Observations of key physical parameters are structured, accumulated and displayed so that patterns and trends can be readily detected, and staff are prompted to make observations at the required frequency.

For peer review only - http://bmjopen.bmj.com/site/about/guidelines.xhtml

		assessments are appropriate for children in their care with prior risk factors<br>• Staff have appropriate skills to monitor and accurately record vital signs<br>• Staff have access to appropriate equipment to monitor and accurately record vital signs, and conduct other clinical assessments<br>• Staff are aware of roles and responsibilities<br>• Staff have time to conduct accurate timely and appropriate monitoring of vital signs, alongside other work commitments<br>• Staff use a standardised approach to assessing illness severity	vital signs<br>- How to record and accumulate observations (e.g. use of a device)<br>• Staff discuss and agree appropriate frequency of observations for individual patients<br>• Equipment required to monitor and accurately record vital signs is readily available<br>• Equipment required to monitor and accurately record vital signs is functioning as it should<br>• There are an appropriate number of staff to carry out required tasks<br>• Staff use a device for recording and accumulating key vital signs		

For peer review only

DETECTION	Detection of deterioration depends on supporting and enabling staff to detect other signs of deterioration and express concerns	At a minimum this requires:<br>• Teams have appropriate skills to discern patterns and trends of signs and symptoms<br>• Work is organised to enable staff to recognise patterns and trends<br>• Staff are encouraged and supported in raising concerns<br>• Staff are encouraged and supported to share learning	At a minimum this requires:<br>• Work to be organised to facilitate continuity of care<br>• Work to be organised to facilitate communication if continuity of care is breached<br>• Staff concern formally recognised as a valid indicator of deterioration<br>• Staff concerns are positively received and acted upon, irrespective of the outcome<br>• Staff receive guidance on how to raise concerns about patient deterioration not necessarily related to observations	• Routine opportunities for more experienced staff to coach those less experienced (clinical supervision)<br>• Skills training: working as a team<br>• Track and Trigger Tool: staff concern a core parameter	• Less experienced staff learn pattern recognition from more experienced staff<br>• Staff respect and value each other's opinion<br>• Staff recognise concern as a valid indicator of deterioration<br>• Staff concern included as a key parameter in the detection of deterioration

For peer review only

D E T E C T I O N	Detection of deterioration depends on supporting and enabling families to detect other signs of deterioration and express concerns	At a minimum this requires:<br>• Staff understand the value of family concerns in the detection of deterioration<br>• Families are in a position to discern patterns of signs and symptoms in their child<br>• Families are encouraged and supported in raising concerns<br>• Parents can voice their concerns	At a minimum this requires:<br>• Families are supported to stay with their children<br>• Families are involved with defining normal parameters for their child and tasked with identifying changes Parents are supported to articulate their concerns<br>• Staff keep families informed about developments in care and treatment<br>• Staff receive guidance about how to respond to family concern	• Parent information leaflets and posters<br>• Skills training: family engagement<br>• "Planning care together" forms<br>• "What to expect" script<br>• Track and Trigger Tool: parent concern a core parameter	• Parents are informed that their views matter, and provided guidance about how to share their concerns with staff<br>• Staff respect and value family engagement<br>• Staff recognise family concern as a valid indicator of deterioration<br>• Parents and staff can share, discuss and document parental concerns.<br>• Parents receive guidance about how to articulate their concerns
	Proposition	Conceptual requirements	Operationalisation	Interventions	Proposed effect of interventions
P R E P A R A T I	Preparation depends on collating and sharing indicators of deterioration for each patient.	At a minimum this requires:<br>• Information from children, staff and families is integrated<br>• Staff are in a position to review and interpret information from children, families and staff Identifying children that need to be	At a minimum this requires:<br>• Information from children, staff and families is interpreted together<br>• Regular opportunities for communication between relevant team members	• Skills training: working as a team<br>• "Watcher"<br>• Common information space<br>• Structured and standardised handover<br>• Huddles	• Staff develop the skills and behaviours required for effective communication, coordination and collaboration and understand their purpose and importance.<br>• A formalised process where the bedside nurse,

For peer review only - http://bmjopen.bmj.com/site/about/guidelines.xhtml

O<br>N		watched			and clinician, proactively identify risk, which includes assessment of information from children, families and staff<br>• Information on deterioration from children, staff and families are brought together, displayed together, regularly updated and easily accessible<br>• Key information from children, staff and families is systematically shared in a structured method, during handovers, between shifts or between staff in the same or different clinical areas.<br>• Senior staff allocated responsibility for knowing which patients are at high risk of deterioration meet with those responsible for conducting observations and liaise with families to discuss plans for individual patient management

For peer review only

	Proposition	Conceptual requirements	Operationalisation	Interventions	Proposed effect of interventions
PREPARATION	Preparation depends on staff being aware at ward level of the status of individual patients and the availability of skills and resources and being able to plan appropriate response.	At a minimum this requires:<br>• There is a regular mechanism for reviewing the status of all children in the ward<br>• The is a regular mechanism for reviewing staffing levels and skills mix, workload, acuity and admissions<br>• There is a regular mechanism for communicating the review of all children, staffing levels and other resources to the rest of the team<br>• There is a regular mechanism for planning appropriate response<br>• Staff are aware of at-risk children and response plans<br>• Staff have situational awareness appropriate to their seniority (e.g. individual child or ward level)	At a minimum this requires:<br>• Information from children, staff and families is brought together<br>• Information from individual patient assessments are brought together and kept up to date<br>• Senior staff members are allocated responsibility for assessing staff levels and resources<br>• Senior staff members are allocated responsibility for managing demand and resources<br>• Senior staff members are allocated responsibility for communicating response plans	• Skills training: working as a team<br>• Common information space<br>• Structured and standardised handover<br>• Huddles<br>• Watch-stander	• Staff develop the skills and behaviours required for effective communication, coordination and collaboration and understand their purpose and importance.<br>• Information on deterioration (physiological, staff concern, family concern) are brought together, displayed together, regularly updated and easily accessible<br>• Key information about patients, staffing levels and other resources is systematically recorded and shared<br>• Senior staff allocated responsibility for knowing which patients are at high risk of deterioration meet with those who oversee patient flow and staffing to discuss plans for overall patient management

For peer review only - http://bmjopen.bmj.com/site/about/guidelines.xhtml

ACTION	Escalation depends on effective communication across professional, disciplinary and hierarchical boundaries.	At a minimum this requires:<br>• A trigger or prompt to act<br>• Clearly defined graded escalation and response procedures – agreed at organisational level<br>• Staff aware of roles and responsibilities<br>• Staff understand their part in the escalation procedure as activators and responders	At a minimum this requires:<br>• Staff receive guidance about graded escalation and response procedures<br>• Staff receive guidance on which members of staff are responsible for activating and responding<br>• Staff receive guidance about how to escalate	• Activation policy<br>• Activation criteria displayed throughout hospital<br>• Staff induction<br>• Track and Trigger Tool	• All staff and parents are aware of escalation and response criteria<br>• All staff and parents are aware of escalation and response procedures and roles and responsibilities<br>• Alerts staff to signs of deterioration and prompts the appropriate response
ACTION	Escalation depends on effective communication across professional, disciplinary and hierarchical boundaries	At a minimum this requires:<br>• Staff understand the significance of information<br>• Staff able to communicate information across professional hierarchies<br>• All staff are able to express concerns	At a minimum this requires:<br>• A structured approach to sharing information<br>• Staff concerns are positively received and acted upon, irrespective of the outcome	• Activation criteria displayed throughout hospital<br>• Staff induction<br>• Track and trigger tool<br>• SBAR	• All staff and parents are aware of escalation and response criteria<br>• All staff and parents are aware of escalation and response procedures and roles and responsibilities<br>• Tool packages legitimises, empowers and informs individuals when attempting to signal deterioration<br>• Time critical information about patients, staffing levels and other resources that requires immediate attention and action is

For peer review only - http://bmjopen.bmj.com/site/about/guidelines.xhtml

					shared in a structured method

For peer review only

For peer review only - http://bmjopen.bmj.com/site/about/guidelines.xhtml

TABLE 2 SCREENING BREAKDOWN

	PEWS	EWS	Structured handover	Situational awareness		Electronic systems	Observations & monitoring	Family involvement	Snowball sample
Database searching	3564	1155	3369	302		/	/	/	/
Additional sources	83	7	150	46		/	/	/	/
Records after duplicates removed	2194	751	2156	199		/	/	/	/
Hand searches	431	/	/	/		26	20	15	5
Title screening	90	751	2156	199		26	20	15	5
Abstract screening	62	106	n/a	n/a		26	20	15	5
Full paper screening	39	65	37	26		26	20	15	5
**Included in Syntheses**	**24**	**22**	**4**	**6**		**10**	**2**	**9**	**5**

For peer review only

TABLE 3 SUMMARY OF DETECTION EVIDENCE

Author	Country	Methodology	Analysis	Search Area	Evidence Contribution
Andrews, T. and Waterman, H. (2005) Packaging: a grounded theory of how to report physiological deterioration effectively. *Journal of Advanced Nursing* 52, 5, 473–481	U.K.	Interviews and observations	Grounded theory	EWS	Importance of 'gut feeling' in detecting deterioration<br><br>Vital sign monitoring delegated to junior staff
Astroth, K., Woith, W. M., Stapleton, S. J., Degitz, R. J., & Jenkins, S. H. (2013). Qualitative exploration of nurses' decisions to activate rapid response teams. Journal of Clinical Nursing, 22(19-20), 2876-2882	U.S.A.	Semi-structured interviews with nurses	Coding categories were generated from the data, and consensus on final themes was achieved through an iterative process	EWS	Staff encouraged to use their intuition when activating the RRT
Azzopardi, P.; Kinney, S.; Moulden, A. & Tibbals, J. Attitudes and barriers to a Medical Emergency Team system at a tertiary paediatric hospital. *Resuscitation*, 82, 167-174	Australia	Survey	Statistical analysis	PEWS	Track & Trigger Tools used to confirm or identify deterioration depending on experience. Useful for junior staff<br><br>Vital sign monitoring delegated to junior staff
Bellomo, R., Ackerman, M., Bailey, M., Beale, R. Clancy, G., Danesh, V. et al	Internatio nal: U.S.A; Sweden;U	Before-and-after multi-centred international	Automated vital signs monitoring and early warning	Electronic systems	The electronic health record (HER) provides a prompt to action

For peer review only - http://bmjopen.bmj.com/site/about/guidelines.xhtml

Author	Country	Methodology	Analysis	Search Area	Evidence Contribution
. (2012) The Vital Signs to Identify, Target, and Assess Level of Care Study (VITAL Care Study) Investigators.. Crit Care Med Vol. 40, No. 8; 2349–2361	.K; Netherlands; Australia	controlled trial.	score (EWS) calculated, international study, blinded trial, hospitals retained own EWS and escalation policy		
Bonafide C., Roberts K., Weirich C., Paciotti B., Tibbetts K., Keren R., Barg F. and Holmes J. (2013) Beyond Statistical Prediction: Qualitative Evaluation of the Mechanisms by which Pediatric Early Warning Scores Impact Patient safety. *J Hospital Medicine*, 8, 5, 248-253	U.S.A.	Semi-structured interviews	Grounded theory	PEWS	Vital sign monitoring delegated to junior workers who may not have the knowledge to interpret results. Track & Trigger Tools used to confirm or identify deterioration depending on experience & particularly useful for junior staff<br><br>Professional intuition important for senior staff to detect deterioration
Bonafide, C. P., et al. (2014). Video Methods for Evaluating Physiologic Monitor Alarms and Alarm Responses. Biomédical Instrumentation & Technology May/June 2014, p220-230	U.S.A.	Prospective Feasibility study	Video recording and electronic patient data collected prospectively. Pragmatic observational study of VitalPAC deployment in 2 large hospitals	Electronic systems	Alarm fatigue a barrier to escalation
Bonafide, C.P. et al. (2017) Video Analysis of Factors Associated With Response Time to Physiologic Monitor Alarms in a Children's Hospital. *JAMA*	U.S.A.	Video review and response time outcome	Statistical analysis	PEWS	Alarm fatigue a barrier to escalation

For peer review only - http://bmjopen.bmj.com/site/about/guidelines.xhtml

Author	Country	Methodology	Analysis	Search Area	Evidence Contribution
Pediatrics, 171, 6, 524-531					
Braaten, J.S. (2015). Hospital System Barriers to Rapid Response Team Activation: A Cognitive Work Analysis. *American Journal of Nursing*, 115(2), 22-32.	U.S.A.	Document review and interviews using the principles of cognitive work analysis	Inductive and deductive forms of analysis – cognitive work analysis, framework and directed content analysis	EWS	Vital sign monitoring delegated to junior workers who may not have the knowledge to interpret results.<br><br>Track and Trigger Tools used to confirm or identify deterioration depending on experience; Professional intuition important for more senior staff/when clinical change is abrupt<br>Issues around availability of equipment and staffing
Brady P.W. and Goldenhar L.M. (2013a). A qualitative study examining the influences on situation awareness and the identification, mitigation and escalation of recognised patient risk. *BMJ Quality and SafetyI*, 0, 1-9.	U.S.A.	Focus groups x7 – held in groups of participants with similar roles	Constant comparison	Situational awareness	Paediatric early warning score (PEWS) supplementing gut feeling but these were not standardised
Burns et al. (2018) Enhanced early warning system impact on nursing practice:<br>A phenomenological study. *Journal of Advanced Nursing*, 1-7.	U.S.A.	Semi-structured interviews were used drawing on a descriptive phenomenological methodology	Iterative thematic analysis	Snowball sample	Importance of professional intuition is reported
Chua, W. L., Mackey, S., Ng, E. K. C., & Liaw, S. Y. (2013). Front line nurses' experiences with deteriorating ward patients: a qualitative study. *International Nursing Review*, 60, 4.,	Singapore	A qualitative survey using critical incident technique	Inductive content analysis	EWS	Vital sign monitoring is the responsibility of nurses. The regularity of this activity can lead to it being viewed as a mundane activity<br><br>Importance of 'gut feeling' and intuition in detection of deterioration

For peer review only

Author	Country	Methodology	Analysis	Search Area	Evidence Contribution
501-509.					
Cioffi, J. (2000). Nurses' experiences of making decisions to call emergency assistance to their patients. Journal of Advanced Nursing, 32(1), 108-114.	Australia	Unstructured interviews with nurses who had activated the medical emergency team (MET)	Simple code and retrieve	EWS	Importance of gut feeling and 'knowing' your patient in the detection of deterioration<br><br>Importance of having staff concern in the MET criterion
Cioffi, J. (2000). Recognition of patients who require emergency assistance: a descriptive study. Heart & Lung, 29(4), 262-268.	Australia	Unstructured interviews	Simple code and retrieve	EWS	Importance of gut feeling and intuition in recognising deterioration<br><br>Importance of having staff concern in a calling criteria
Cioffi, J., Salter, C., Wilkes, L., Vonu-Boriceanu, O., & Scott, J. (2006). Clinicians' responses to abnormal vital signs in an emergency department. Australian Critical Care, 19(2), 66-72.	Australia	Focus groups with clinicians and nurses exploring their responses to abnormal vital signs	Constant comparison	EWS	Intuition important, particularly for more senior staff to detect AVS. Knowing your patient reported as key; knowledge and experience seen as essential.
Claussen, D., Garner, D. and Crow, B. (2013) Early warning signs and the EHR: At the intersection of technology and care. *Nursing Management*, 44, 11, 14-16	U.S.A.	Retrospective review of calls to the rapid response team (RRT) and cardiac arrest calls to evaluate impact of evidence based guidelines (pre-intervention). EWS and huddles implemented. EHR available throughout to compare pre and	Descriptive statistics	Electronic systems	Professional intuition used in conjunction with MEWS

For peer review only - http://bmjopen.bmj.com/site/about/guidelines.xhtml

Author	Country	Methodology	Analysis	Search Area	Evidence Contribution
		post			
Davies, O., DeVita, M.A., Ayinla, A. and Perez, X.. (2014). Barriers to activation of the rapid response system. *Resuscitation*, 85(11), 1557-1561.	U.S.A.	Survey looking at barriers to rapid response system (RRS) activation	Statistical analysis	EWS	Professional intuition used over RRS activation criteria – barrier to activation
de Groot, J F. et al (2018) "Implementing Paediatric Early Warning Scores Systems in the Netherlands : Future Implications.. *BMC Pediatrics,* 18: 128	Netherlands	Retrospective patient review & semi structured interviews with professionals	Descriptive statistics and grounded theory	PEWS	Importance of professional intuition is reported
Donohue, L. and Endacott, R. (2010) Track, trigger and teamwork: Communication of deterioration in acute medical and surgical wards. *Intensive and Critical Care Nursing*, 26, 10-17	U.K.	Qualitative design with critical incident technique. Semi-structured interviews with nurses and the outreach team	Thematic analysis	EWS	Importance of gut feeling and intuition in detecting deterioration
Downey, C.L., Tahir, W., Randell, R., Brown, J.M. and Jayne, D.G. (2017) Strengths and limitations of early warning scores: A systematic review and narrative synthesis. *International Journal of Nursing Studies*, 76, 106-119	U.K.	Narrative review	"Patterns were identified and translated to themes, which were further refined using an iterative process".	PEWS	Drive towards automated alerts
Endacott et al (2007). Recognition and	U.K.	Mixed methods case study – semi	Qualitative content analysis &	Obs and monitoring	Gut feeling important – interestingly particularly for nurses whereas doctors sought additional empirical evidence to back up intuition

For peer review only

Author	Country	Methodology	Analysis	Search Area	Evidence Contribution
communication of patient deterioration in a regional hospital: A multi-methods study. Australian Critical care 20, 100—105		structured interviews and audit of charts	descriptive statistics		
Endacott, R., & Westley, M. (2006). Managing patients at risk of deterioration in rural hospitals: a qualitative study. Australian Journal of Rural Health, 14(6), 275-279	Australia	Questionnaire, in-depth interviews and observations	Content analysis and constant comparison	EWS	Intuition and anticipatory skills important in detecting deterioration
Entwistle, V. (2004). Nursing shortages and patient safety problems in hospital care: is clinical monitoring by families part of the solution? Health Expectations, 7, pp.1–5 (editorial)	U.S.A.	Editorial	N/a	Family involvement	Little evidence/no evaluations of policies or practices that encourage and support family involvement in clinical monitoring<br><br>Propose the innovative practice of interdisciplinary rounds where families are invited, and communication is directed to the patient and family
Fagan, K.,Sabel, A., Mehler, P. S. and MacKenzie, T. D. (2012). Vital Sign Abnormalities, Rapid Response, and Adverse Outcomes in Hospitalized Patients. American Journal of Medical Quality 27(6) 480– 486	U.S.A.	Observational Cohort comparison study	Descriptive statistics	Electronic systems	Concerns about over-burdening staff with automated alerts.<br><br>When considering the patient's baseline, nursing staff are expected to notify the patient's care provider immediately responsible for the patient when the vital signs meet or exceed the clinical trigger
Graedon, J. and Graedon, T. (2006) Enlisting Families as Patient Safety Allies. Clinical Paediatric *Emergency Medicine*, 265-	U.S.A focus	Opinion piece	N/a	Family involvement	Parents to explain child's baseline<br><br>Need to have detailed information about signs and symptoms that they should look for that would require alerting the medical team

For peer review only - http://bmjopen.bmj.com/site/about/guidelines.xhtml

Author	Country	Methodology	Analysis	Search Area	Evidence Contribution
267					Propose the need for structured inclusion of family concern during ward rounds (safety walkrounds)
Hands, C. et al. (2013) Patterns in the recording of vital signs and early warning scores: compliance with a clinical escalation protocol. *Quality and Safety in Health Care*, 1-8.	U.K.	The vital signs (V.S.) and early warning data for all in-patients for one year to investigate patterns of V.S observations collected	Statistical	Obs and Monitoring	Staffing levels and availability of equipment impede the collection of vital signs and early warning data.<br><br>Night-time factors also play a role with a decrease in observations noted
Hope et al. (2017) A fundamental conflict of care: Nurses' accounts of balancing patients' sleep with taking vital sign observations at night. *Journal of Clinical Medicine*	U.K.	Semi-structured interviews with 17 registered nurses	Constant comparative method informed by grounded theory	Snowball sample	Wider context of night-time care considered; there is some indication that staff are making a choice and prioritising sleep over monitoring
James, J., Butler-Williams, C., Hunt, J. and Cox, H. (2010) Vital signs for vital people: an exploratory study into the role of the Healthcare Assistant in recognising, recording and responding to the acutely ill patient in the general ward setting. *Journal of Nursing Management*, 18, 548-555	U.K.	Postal survey with Healthcare Assistants (HCAs) utilising closed and open questions	Descriptive statistics & content analysis of qualitative data	Observations and monitoring	Factors other than the score used to detect deterioration - intuition/patient's own descriptions<br><br>Vital sign monitoring delegated to junior staff who may not have the skills to interpret results; many HCAs did not "fully understand this neurological assessment and it is implications for the acutely unwell patient thus resulting in the risk of an inaccurate EWS score" p 552.
Jensen, C. et al. (2018) Pediatric Early Warning Score Systems, Nurses Perspective – A Focus Group Study. *Journal of*	Denmark	Focus group exploring nurse' experiences with PEWS	Qualitative meaning condensation analysis	PEWS	Sleeping as a part of care identified<br><br>Professional intuition<br><br>Night-time pressures

For peer review only - http://bmjopen.bmj.com/site/about/guidelines.xhtml

Author	Country	Methodology	Analysis	Search Area	Evidence Contribution
Pediatric Nursing					
Jones, D. et al. (2006). Nurses' attitudes to a medical emergency team service in a teaching hospital. Quality & Safety in Health Care, 15(6), 427-432.	Australia	Questionnaire about understandings and barriers to activating a medical emergency team (MET)	Simple descriptive statistics	EWS	Intuition important when detecting deterioration
Jones, S., Mullally, M., Ingleby, S., Buist, M., Bailey, M. and Eddleston, J. M. (2011). Bedside electronic capture of clinical observations and automated clinical alerts to improve compliance with an Early Warning Score protocol. Crit Care Resusc; 13: 83–88	U.K.	Electronic capture of physiological data to see if automated clinical alerts increase compliance with an early warning score (EWS) and improve patient outcomes	Statistical	Electronic systems	Electronic systems can provide prompts or alerts for monitoring vital signs<br><br>This system demonstrated significant improvement in clinician attendance to acutely ill patients with an early warning score of 3 and above
Kaul, M., Snethen, J., Kelber, S., Zimmanck, K., Maletta, K. and Meyer, M. (2014) Implementation of the bedside paediatric early warning system (BedsidePEWS) for nurse identification of deteriorating patients'. *Journal for specialists in pediatirc nursing*, 19, 339-349	U.S.A.	Descriptive cross sectional study; nurse and medical staff survey	Descriptive statistics	PEWS	Benefits of BedsidePEWS – nurses more likely to identify early signs of deterioration and a greater ability to escalate care. Delegation to junior staff justified.
Lobos AT, Fernandes R, Ramsay T, McNally JD. (2014) Patient	Canada	Implementation report	Simple descriptive statistics	PEWS	Delegation to junior staff<br><br>Education package developed around the RRS with a variety of informal lectures

For peer review only

Author	Country	Methodology	Analysis	Search Area	Evidence Contribution
characteristics and disposition after pediatric medical emergency team (MET) activation: disposition depends on who activates the team. Hospital Pediatrics. 4(2):99-105					and interactive sessions; “lunch and learns”, “education coffee carts” and simulation programs for instance.
Lydon et al. (2016) A mixed-methods investigation of health professionals’ perceptions of a physiological track and trigger system. *BMJ Quality and Safety*, 25, 688-695		30 semi-structured interviews based on the theory of planned behaviour were conducted.<br><br>Questionnaire with Likert scale developed from the interview data	Deductive content analysis	PEWS	Professional intuition
Mackintosh, N., Humphrey, C., & Sandall, J. (2014a). The habitus of 'rescue' and its significance for implementation of rapid response systems in acute health care. Social Science & Medicine, 120, 233-242	U.K.	Ethnographic perspective ; observations, semi-structured interviews	Data were inductively and deductively coded using NVivo v8 and organised thematically.	EWS	Delegation of routine observation and vital sign monitoring to junior staff<br><br>Professional intuition important; ‘knowing the patient’ important for detecting subtle changes in a patient’s condition
Mackintosh, N., Rainey, H. and Sandall, J. (2012a) Understanding how rapid response systems may improve safety for the acutely ill patient: learning from the frontline. *BMJ Qual Saf*, 21, 135-144	U.K.	Comparative case study a rapid response system (RRS) using ethnographic methods including observations, interviews and documentary	Inductive and deductive coding facilitated by NVivo. Also utilised theme building and structuring methods from framework analysis whilst also	EWS	Observations and monitoring delegated to junior staff/health care assistants (HCAs) and nurses – early warning system (EWS) legitimised delegation of these tasks. Importance of pattern recognition and intuition<br><br>The TTT used to confirm or identify deterioration and are particularly useful for junior staff.

For peer review only - http://bmjopen.bmj.com/site/about/guidelines.xhtml

Author	Country	Methodology	Analysis	Search Area	Evidence Contribution
		review.	informed by other theoretical frameworks such as 'technology-in-practice'.		
Mackintosh, N., Watson, K., Rance, S., & Sandall, J. (2014b). Value of a modified early obstetric warning system (MEOWS) in managing maternal complications in the peripartum period: an ethnographic study. BMJ Quality & Safety, 23(1), 26-34	U.K.	Ethnographic study using observations (>120 h), semi-structured interviews (n=45) and documentary review	Thematic analysis with data analysed iteratively in addition to a more strategic and policy-focused coding framework	EWS	Professional intuition important when detecting deterioration
Massey, D., Chaboyer, W., & Aitken, L. (2014). Nurses' perceptions of accessing a Medical Emergency Team: a qualitative study. Australian Critical Care, 27(3), 133-138	Australia	In depth semi structured interviews	Inductive approach – thematic analysis	EWS	Importance of intuition or 'sensing clinical deterioration'
McDonnell, A., Tod, A., Bray, K., Bainbridge, D., Adsetts, D., & Walters, S. (2013). A before and after study assessing the impact of a new model for recognizing and responding to early signs of deterioration in an acute hospital. *Journal of Advanced Nursing,* 69(1), 41-52.	U.K.	Single centre, mixed method before and after study including a survey to measure changes in nurses' knowledge after implementation of a track and trigger system (T&Ts). Also, qualitative interviews	Statistical analysis and thematic framework analysis	EWS	T&Ts used to confirm or identify deterioration depending on the experience of the user. Specific objective information was seen as helping the response arm prioritise work<br><br>Importance of professional intuition or 'gut feeling' in detecting deterioration<br><br>Need for staff concern in T&Ts

For peer review only - http://bmjopen.bmj.com/site/about/guidelines.xhtml

Author	Country	Methodology	Analysis	Search Area	Evidence Contribution
McKay, H., Mitchell, I., Sinn, K., Mugridge, H., Lafferty, T., Leuvan,, C. Mamootil, S. and Abdel-Latif, M. (2013) "Effect of a multifaceted intervention on documentation of vital signs and staff communication regarding deteriorating paediatric patients", *Journal of Paediatrics and Child Health,* Volume 49, Issue 1, pages 48–56	Australia	Prospective, controlled before and after intervention trial.	Statistical analysis	PEWS	Specific education package developed around the Paediatric Early Warning Score (PEWS) which assists in the recognition of deterioration
Mohammed, Hayton, R., Clements, G., Smith, G. and Prytherch, D. (2009) Improving accuracy and efficiency of early warning scores in acute care. *British Journal of Nursing*, 18, 1, 18-24	U.K.	Three phases; the first two were based in a classroom and asked nurses to calculate an early warning score (EWS) from vignettes using pen and paper followed by a hand-held computer. The third phase followed the previous approach but was based on the ward after nurses had been using the device for 4 weeks	Statistical	Electronic systems	Timeliness of electronic vital sign recording when compared with paper systems
Mohammed Iddrisu, S. et	Australia	To explore nurse'	Thematic analysis	Snowball	Staff concern in tool criteria useful

For peer review only - http://bmjopen.bmj.com/site/about/guidelines.xhtml

Author	Country	Methodology	Analysis	Search Area	Evidence Contribution
al (2018) Nurses role in recognising and responding to clinical deterioration. *Journal of Clinical Nursing,* 27, 9-10, 1920-1930		role in recognising and responding to deteriorating postoperative patients through focus groups.		sample	
Paciotti B, Roberts KE, Tibbets KM, Weirich Paine C, Keren R, Barg FK, Holmes J H and Bonafide CP (2014). Physician attitudes toward family-activated medical emergency teams for hospitalized children. The Joint Commission Journal on Quality and Patient Safety, 40 (4)	U.S.A.	Semi-structured interviews with clinicians	Grounded theory and constant comparison	Family involvement	Physicians depend on families to explain a child's base-line
Pattison, N., & Eastham, E. (2012). Critical care outreach referrals: a mixed-method investigative study of outcomes and experiences. *Nursing in Critical Care,* 17(2), 71-82.	U.K.	Mixed method study looking at the impact of a critical care outreach team (CCOT)	Statistical analysis and grounded theory	EWS	Track and trigger tool is used to either confirm or identify deterioration depending on the experience of the user<br><br>'Gut feeling'/intuition important – patient appearance often an important sign in detecting deterioration
Radeschi, G., Urso, F., Campagna, S., Berchialla, P., Borga, S., Mina, A., . . . Sandroni, C. (2015). Factors affecting attitudes and barriers to a medical emergency team among nurses and medical doctors: A multi-centre	Italy	Multicentre survey to identify the attitudes and barriers to medical emergency team (MET) utilisation	Statistical analysis	EWS	Intuition (physical appearance important) in detecting deterioration

For peer review only - http://bmjopen.bmj.com/site/about/guidelines.xhtml

Author	Country	Methodology	Analysis	Search Area	Evidence Contribution
survey. *Resuscitation*, 88, 92-98.					
Schmidt, P. E., et al (2014). Impact of introducing an electronic physiological surveillance system on hospital mortality. BMJ Quality and safety.	U.K.	Retrospective analysis of data collected routinely. Pragmatic observational study of VitalPAC deployment in 2 large hospitals	Statistical analyses	Electronic systems	Electronic systems provide prompts for alerts facilitates better recognition of deterioration and is associated with reduced mortality.
Sefton, G., Lane, S., Killen, R. et al. (2017) Accuracy and Efficiency of Recording Pediatric Early Warning Scores Using an Electronic Physiological Surveillance System Compared With Traditional Paper-Based Documentation. *Computers, Informatics, Nursing,* 35, 5, 228 – 236	U.K.	Controlled study of vital sign documentation & PEWS calculation and a survey of acceptability	Descriptive statistics	PEWS	Errors in paper-based documentation were found; incorrect age-specific chart used; inaccurate documentation of values and plotting of trends; incorrect PEWS calculation. In comparison the error rate using the electronic PEWS was low
Shearer, B., Marshall, S., Buist, M. D., Finnigan, M., Kitto, S., . . . Ramsay, W. (2012). What stops hospital clinical staff from following protocols? An analysis of the incidence and factors behind the failure of bedside clinical staff to activate the rapid response system in a multi-campus Australian	Australia	A mixed-method study	Iterative coding	EWS	Importance of professional intuition

For peer review only - http://bmjopen.bmj.com/site/about/guidelines.xhtml

Author	Country	Methodology	Analysis	Search Area	Evidence Contribution
metropolitan healthcare service. BMJ Quality & Safety, 21(7), 569-575					
Sonning, K, et al. (2018) "A Survey of Healthcare Professionals' Experiences with the Paediatric Early Warning Score (PEWS)." *Norwegian Journal of Clinical Nursing* 12(64605).	Norway	Questionnaire of a sample of staff who use PEWS	Descriptive statistics	PEWS	Appreciate that PEWS encourages a systematic approach to monitoring.
Stevenson, J. E. & Nilsson G. (2011). Nurses' perceptions of an electronic patient record from a patient safety perspective: a qualitative study. Journal of Advanced Nursing 68(3), 667–676	Sweden	Qualitative; focus groups with 21 registered nurses	Content analysis of interviews	Electronic systems	Verbal reports were favoured over the electronic system
Stewart, J., Carman, M., Spegman, A., & Sabol, V. (2014). Evaluation of the effect of the modified early warning system on the nurse-led activation of the rapid response system. *Journal of Nursing Care Quality*, 29(3), 223-229.	Sweden	Mixed method. Retrospective review of records and nurse led focus groups	Statistical analysis and content analysis	EWS	Intuition still plays a part – the 'score' was rarely the single determining factor in activation but rather prompted nurses to gather additional information
Subbe et al. (2018) Effect of an automated notification system for deteriorating ward patients on clinical outcomes. *Critical Care*, 21, 52	U.K.	A before and after study of an electronic automated advisory V.S. monitoring and notification system. Elevated	Statistical analysis	Snowball sample	Automated VS monitoring associated with a decrease in mortality

For peer review only - http://bmjopen.bmj.com/site/about/guidelines.xhtml

Author	Country	Methodology	Analysis	Search Area	Evidence Contribution
		scores were relayed to a RRT.			
Wager, K. A., Schaffner, M. J., Foulois, B., Swanson Kazley, A., Parker, C. Walo, H. (2010). Comparison of the Quality and Timeliness of Vital Signs Data Using Three Different Data-Entry Devices. Computers, Informatics, Nursing & Vol. 28, No. 4, 205–212	U.S.A.	Observational study.	Descriptive statistics	Electronic systems	Batching of patient data whereby the care provider handwrites the patient's vital signs and uploads it to the computer at a later time is common, especially as the computers are often busy. Individual tablet PCs seems to eliminate this from occurring.
Watson, A., Skipper, C., Steury, R., Walsh, H. and Levin, A. (2014) Inpatient nursing care and early warning scores: a workflow mismatch. *J Nurs Care Qual*, 29, 3, 215-222	U.S.A.	Mixed method, retrospective medical record observations and observations of nurse interactions in one minute blocks.	Observation analysis, although this is not described, and statistical analysis	PEWS	Information relating to transfer to electronic systems – distance the 'eyes' of the nurses from the patient; batching.<br><br>Intuition
Wheatley (2006). The nursing practice of taking level 1 patient observations. I&CCNurs 22: 115-121	U.K.	Ethnographic approach; participant observation & semi-structured interviews	Thematic & content analysis	Observations and monitoring	The regularity of vital signs monitoring may also lead to it becoming viewed as mundane practice that can be delegated to health care assistants.<br><br>Workplace pressure/equipment failures affect quality of observations

For peer review only

TABLE 4 SUMMARY OF PREPARATION EVIDENCE

Author	Country	Methodology	Analysis	Search Area	Evidence Contribution
Abraham J, Kannampallil T, Patel B, Almoosa K, Patel V. (2012) Ensuring patient safety in care transitions: an empirical evaluation of a Handoff *Intervention Tool. AMIA Annual Symposium Proceedings/AMIA Symposium*, 17-26	U.S.A.	Pre and post prospective study.	The quality and completeness of the handoff note – both tools – was assessed by a multi-professional round	Structured handover	Value of a checklist tool for handover
Brady P.W. and Goldenhar L.M. (2013a). A qualitative study examining the influences on situation awareness and the identification, mitigation and escalation of recognised patient risk. *BMJ Quality and SafetyI*, 0, 1-9.	U.S.A.	Focus groups x7 – held in groups of participants with similar roles	Constant comparison	Situational awareness	Huddle useful to proactively identify and plan for risk<br><br>Structure to support handover important
Brady P.W., Muething S., Kotagal et al. (2013b). Improving situation awareness to reduce unrecognized clinical deterioration and serious safety events. *Pediatrics*, 131, 1.	U.S.A.		Statistical process control charts	Situational awareness	Huddles led by a Watchstander charge nurse. When risk is identified the team discussed this and developed a plan to mitigate risk
Claussen, D., Garner, D. and	U.S.A.	Retrospective	Descriptive statistics	Electronic	The huddle was seen as useful and was called as a patient's status

For peer review only - http://bmjopen.bmj.com/site/about/guidelines.xhtml

Author	Country	Methodology	Analysis	Search Area	Evidence Contribution
Crow, B. (2013) Early warning signs and the EHR: At the intersection of technology and care. *Nursing Management*, 44, 11, 14-16		review of calls to the rapid response team (RRT) and cardiac arrest calls to evaluate impact of evidence-based guidelines		systems	was changing to 'red' so that all team members were informed
Davies, O., DeVita, M.A., Ayinla, A. and Perez, X.. (2014). Barriers to activation of the rapid response system. *Resuscitation*, 85(11), 1557-1561.	U.S.A.	Survey looking at barriers to rapid response system (RRS) activation	Statistical analysis	EWS	Activation criteria displayed around the hospital
Demmel KM, Williams L, Flesch L. (2010) Implementation of the Pediatric Early Warning Scoring System on a Pediatric Hematology/Oncology Unit. *Journal of Pediatric Oncology Nursing*, 27(4):229-240	U.S.A.	Discussion of the set-up and implementation of a paediatric early warning scoring tool and an associated algorithm	Rapid Plan-Do-Study-Act (PDSA) cycles were implemented using small tests of change. The data from the PDSA cycles were continuously collected, analysed, and reviewed with the multidisciplinary staff and planning team and used to give ongoing direction to the implementation plan.	PEWS	Importance of common information spaces & display of activation criteria throughout the hospital
Donahue M, Smith L, Dykes P. (2010) Phase 2 of the EMPOWER project: enhancing communication for paraprofessionals. *Journal of Continuing Education in Nursing*. 0;41(5).	U.S.A.	Focus groups evaluation of a training programme which was developed to teach paraprofessionals situation, background, assessment, recommendation	Not clear	Structured handover	Value of a structured approach to handover<br><br>Adaptation of the SBAR tool for handover

For peer review only - http://bmjopen.bmj.com/site/about/guidelines.xhtml

Author	Country	Methodology	Analysis	Search Area	Evidence Contribution
		(SBAR) communication tool			
Ennis, L (2014) Paediatric early warning scores on a children's ward: a quality improvement initiative. *Nursing children and young people*, 26, 7, 25-31	Ireland	Description of implementation of paediatric early warning score (PEWS) and subsequent audit (prospective cohort observational study)	Simple descriptive statistics of numbers children triggering PEWS and compliance with escalation protocol	PEWS	Common information spaces important & display of activation criteria throughout the hospital<br><br>Usefulness of ISBAR as a communication tool
Goldenhar LM, Brady PW, Sutcliffe KM, Muething SE (2013). Huddling for high reliability and situation awareness. *BMJ Quality & Safety*, 22, 899-906	U.S.A.	Semi structured interviews and focus groups to develop a deeper understanding of a newly implemented huddle systems	Constant comparison	Situational awareness	Importance of the huddle - Empowerment and sense of community; Facilitated greater and better information sharing<br><br>Each huddle participant was asked to systematically report on patients in their units who they thought would deteriorate in the near future and to label them as 'watchers', asking senior nurses and physician leads to coach charge nurses on how to integrate their perceptions into a informal severity of illness assessment (comprehension) and training the clinicians on how to use the information to facilitate prediction and planning for at risk patients (projection).
Mackintosh N, Berridge E-J, Freeth D. (2009) Supporting structures for team situation awareness and decision making: insights from four delivery suites. *Journal of Evaluation in Clinical Practice.*15(1):46-54.	U.K.	Ethnographic<br>2 stage process of lightly structured observations followed by a more focused period of data generation to test and elaborate the outcomes from stage 1	Initial thematic analysis and search for negative cases.	Situational awareness	For teams with a strong level of situation awareness (SA), key supports were all used in a balanced and supportive manner to gather and disseminate information which served to promote a culture of shared ownership and a proactive model of workload management, with temporary disruptions to the model easily accommodated.<br><br>Whiteboard – when used effectively was a vital means for supporting SA. It provides teams with a snapshot of a constantly changing workload, the team's activity levels and resource availability. Workload at times compromised its effectiveness. And irrespective of workloads, location and local traditions had a bearing on its use

For peer review only - http://bmjopen.bmj.com/site/about/guidelines.xhtml

Author	Country	Methodology	Analysis	Search Area	Evidence Contribution
					and usefulness.<br><br>White boards need to be in a visible location<br><br>Importantly, the interplay between these key support for SA will vary depending on the context; the same supports used differently will naturally produce different outcomes<br><br>Handover took many forms; structured and informal; profession specific and inter-professional; participatory or a one-way transmission of information. The effectiveness of SA was dependent upon the form that handover took – who was present, contributions made, information relayed. SA was more likely to be compromised when key people were absent.<br><br>Senior staff coordinator important for SA and became compromised if they were largely focussed on providing patient care.
Mackintosh, N., Rainey, H. and Sandall, J. (2012a) Understanding how rapid response systems may improve safety for the acutely ill patient: learning from the frontline. *BMJ Qual Saf*, 21, 135-144	U.K.	Comparative case study a rapid response system (RRS) using ethnographic methods including observations, interviews and documentary review.	Inductive and deductive coding facilitated by NVivo. Also utilised theme building and structuring methods from framework analysis whilst also informed by other theoretical frameworks such as 'technology-in-practice'.	EWS	Availability of equipment an issue where the track and trigger tool (TTT) was electronic<br><br>Electronic systems helped HCAs and nursing staff to share understandings, planning and manage their workload
Massey, D., Chaboyer, W., & Aitken, L. (2014). Nurses' perceptions of accessing a Medical Emergency Team: a qualitative study. Australian Critical Care, 27(3), 133-138	Australia	In depth semi structured interviews	Inductive approach – thematic analysis	EWS	Common information spaces useful. Display of activation criteria throughout hospital
McCrory et al. (2012) ABC-SBAR Training improves	U.S.A.	Prospective, pre-interventional and	Two blinded reviewers assessed 52 video-recorded	Structured handover	Information sharing for handovers are of variable quality

For peer review only - http://bmjopen.bmj.com/site/about/guidelines.xhtml

Author	Country	Methodology	Analysis	Search Area	Evidence Contribution
simulated critical patient hand-off by pediatric interns. *Pediatric Emergency Care*. 0;28(6).		post-interventional study to evaluate the educational intervention of teaching ABC-SBAR	hand-offs for inclusion, order, and elapsed time to essential hand-off information using a scoring tool		A more structured approach will improve information sharing and therefore situational awareness – "without a structured hand-of tool, paediatric interns overemphasize background information and leave the reason for the call delayed".<br><br>Adaptation of the SBAR tool to include ABC – usefulness of this
Mullan PCM. A novel briefing checklist at shift handoff in an emergency department improves situational awareness and safety event identification. *Pediatric Emergency Care*. 2015;31(4):231-8.	U.S.A.	Descriptive observational study	Checklists were evaluated for rates of use, completion and identification of potential safety events	Situational awareness	The value of more structured approach to information sharing and situational awareness.<br><br>Utilises a checklist handover system for physicians. Checklist items focussed on the status of the patient, ED providers and hospital resources. A "Read-Do" format was designed.
Parker, M.W, Carroll, M., Bolser, B. et al. (2017) Implementation of a Communication Bundle for High-Risk Patients. *Hospital Pediatrics*, 7, 9, 523-529	U.S.A.	Manual review of all eligible patient records	Descriptive statistics	PEWS	Example of a bundle around the "watcher" category with 5 component that needed to be completed within two hours of a patient being designated as such.
Pearson G & Duncan H. (2011) Early warning systems for identifying sick children Paediatrics and Child Health. 21(5): 232-233	U.K.	Brief review of the evidence base surrounding PEWS	N/a	PEWS	Value of a more structured approach to communication - Advocate the use of a shared communication model such as SBAR (Situation, Background,<br>Assessment and Recommendation) to communicate findings to superiors.
Pezzolesi C, Manser T, Schifano F, Kostrzewski A, Pickles J, Harriet N, et al. (2013) Human factors in clinical handover: Development and testing of a 'handover performance	U.K.	Delphi Study for tool development	Descriptive statistics<br><br>Handovers were analysed and rated according to a measure of essential skills	Situational awareness	Information on handover – can be of variable quality<br><br>Most handover tools are adaptations of Situation Background Assessment Recommendation (SBAR) communication tool<br><br>Important to remember that handover facilitates teamwork and this hinges on communicative forms that extend beyond the information

For peer review only - http://bmjopen.bmj.com/site/about/guidelines.xhtml

Author	Country	Methodology	Analysis	Search Area	Evidence Contribution
tool' for doctors' shift handovers. *International Journal for Quality in Health Care*. 25(1):58-65.					transfer that is typically the focus of structured handover tools
Randhawa S, Roberts-Turner R, Woronick K, DuVal J. (2011) Implementing and Sustaining Evidence-Based Nursing Practice to Reduce Pediatric Cardiopulmonary Arrest. Western Journal of Nursing Research. 33(3):443-456	U.S.A.	Description of the implementation process with cardiopulmonary arrest (CPA) statistics pre and post implementation	Once a cycle from the implementation has been completed this is evaluated and then another cycle begins.	PEWS	White boards placed in a central location displayed scores of all patients so that staff could quickly assess which patients were at high risk for deterioration.<br><br>Activation criteria displayed throughout the hospital
Stewart, J., Carman, M., Spegman, A., & Sabol, V. (2014). Evaluation of the effect of the modified early warning system on the nurse-led activation of the rapid response system. *Journal of Nursing Care Quality*, 29(3), 223-229.	Sweden	Mixed method. Retrospective review of records and nurse led focus groups	Statistical analysis and content analysis	EWS	Huddle –the score was used during the daily bed 'huddle' with the nursing administrators, to evaluate current unit acuity, determine staffing need & prepare for any transfers
Van Voorhis, K.T. and Scahde Willis, T. (2009) Implementing a pediatric rapid response system to improve quality and patient safety. *Pediatr Clin N Am*, 56, 919-933	U.S.A.	Discussion paper highlighting the process of developing a paediatric rapid response system (RRS).	N/a	PEWS	Display of activation criteria throughout hospital on lanyards and use of white boards useful
Vries, A., Draaisma, J. and Fuijkschot, J. (2017) Clinician Perceptions of an Early	Netherland s	Semi-structured interview	Qualitative content analysis	PEWS	PEWS/TTT is used to support situational awareness. Use of PEWS enables clinicians to have a 'birds-eye' view over admitted patients

For peer review only - http://bmjopen.bmj.com/site/about/guidelines.xhtml

Author	Country	Methodology	Analysis	Search Area	Evidence Contribution
Warning System on Patient Safety. *Hospital Pediatrics*, 7, 10, 579-586					
Wager, K. A., Schaffner, M. J., Foulois, B., Swanson Kazley, A., Parker, C. Walo, H. (2010). Comparison of the Quality and Timeliness of Vital Signs Data Using Three Different Data-Entry Devices. Computers, Informatics, Nursing & Vol. 28, No. 4, 205–212	U.S.A.	Observational study.<br><br>Explored the timeliness and quality of vital signs data entered by 3 different recording methods	Descriptive statistics	Electronic systems	Batching of patient data whereby the care provider handwrites the patient's vital signs and uploads it to the computer at a later time is common, especially as the computers are often busy.
Watson, A., Skipper, C., Steury, R., Walsh, H. and Levin, A. (2014) Inpatient nursing care and early warning scores: a workflow mismatch. *J Nurs Care Qual*, 29, 3, 215-222	U.S.A.	Mixed method, retrospective medical record observations and observations of nurse interactions	Observation analysis, although this is not described, and statistical analysis	PEWS	Availability of equipment a factor
Weiss M.J.B., Bhanji, F., Fontela, PS. And Razack, SI.. (2013) A preliminary study of the impact of a handover cognitive aid on clinical reasoning and information transfer. Medical Education. 0;47(8)	Canada	A randomised controlled trial in an academic paediatric intensive care unit (PICU) of 20 handovers events	Differences between intervention and control groups were assessed using the Mann-Whitney test and multivariate linear regression.	Structured handover	Value of a more structured approach to support information sharing - a cognitive aid to facilitate handover that prompted residents to transmit this information. The handover aid was not linked into hospital information systems – so this had to be completed by hand before handover.<br><br>Handover is an opportunity for learning and professional socialisation
Wong, D., Bonnici, T., Knight, J., Morgan, L., Coombes, P. and Watkinson, P. (2015) SEND: a system for electronic notification and documentation of vital sign observations. *BMC Medical*	U.K.	Description of user-focussed design process for use of electronic monitoring & numbers of observations taken	Descriptive statistics on the number of observations recorded using the SEND system & the number of active users	Electronic systems	Development of a flexible electronic system which enabled staff to have an overview of patients – reflections on disconnection

For peer review only - http://bmjopen.bmj.com/site/about/guidelines.xhtml

Author	Country	Methodology	Analysis	Search Area	Evidence Contribution
Informatics and Decision Making, 15, 68.		using the system. Acceptability questionnaire			

For peer review only

TABLE 5 SUMMARY OF ACTION EVIDENCE

Author	Country	Methodology	Analysis	Search Area	Evidence Contribution
Adelstein, B. A., Piza, M. A., Nayyar, V., Mudaliar, Y., Klineberg, P. L., & Rubin, G. (2011). Rapid response systems: A prospective study of response times. Journal of Critical Care, 26, 635	Australia	Prospective comparison of rapid response team (RRT) criteria breaches	Statistical	EWS	Day/night differences in activation identified<br><br>Nurses may not understand what is required for activation.<br><br>Staff not investing in calling criteria
Almblad, A.C., Siltberg, P., Engvall, G. and Malqvist, M. (2018) Implementation of Pediatric Early Warning Score; Adherence toGuidelines and Influence of Context. *Journal of Pediatric Nursing,* 38, 33-39	Sweden	retrospective review of EPR and a context assessment of the work environment using the Alberta Context Tool.	Statistical	Snowball sample	Senior leadership consistently identified as important
Andrews, T. and Waterman, H. (2005) Packaging: a grounded theory of how to report physiological deterioration effectively. *Journal of Advanced Nursing* 52, 5,473–481	U.K.	Interviews and observations	Grounded theory	EWS	Track & Trigger Tools (TTT) act as prompts to action<br><br>TTT used to overcome challenges in communication & particularly valuable for junior staff<br><br>Negative attitude towards calling for help
Astroth, K., Woith, W. M., Stapleton, S. J., Degitz, R.	U.S.A.	Semi-structured interviews with	Coding categories were generated	EWS	Situation under control – no need to escalate or perceived business of medical staff discouraged staff from RRT activation

For peer review only - http://bmjopen.bmj.com/site/about/guidelines.xhtml

Author	Country	Methodology	Analysis	Search Area	Evidence Contribution
J., & Jenkins, S. H. (2013). Qualitative exploration of nurses' decisions to activate rapid response teams. Journal of Clinical Nursing, 22(19-20), 2876-2882		nurses	from the data, and consensus on final themes was achieved through an iterative process		Staff encouraged to use their intuition when activating the RRT<br><br>Concern about feeling inadequate in front of colleagues a barrier to RRT activation<br>Inexperienced staff teaming up – led to staff trusting their own judgement<br><br>Traditional hierarchies a barrier to RRT activation – nurses more likely to call the attending physician rather than activate the RRT
Azzopardi, P.; Kinney, S.; Moulden, A. & Tibbals, J. Attitudes and barriers to a Medical Emergency Team system at a tertiary paediatric hospital. *Resuscitation*, 82, 167-174	Australia	Survey	Statistical analysis	PEWS	Score rarely the determining factor in escalation - would not escalate for a patient who looked well but would escalate for a patient they were worried about even if not triggering<br><br>Negative attitude towards calling for help – feeling inadequate/perceived business of Paediatric Intensive Care Unit had an impact on doctors escalating but not nurses<br><br>Senior leadership is important when implementing a MET
Bavare, A.C., Thomas, J.K., Elliott, E.P., Morgan, A.C. and Graf, J. (2017) Family-initiated pediatric rapid response: characteristics, impetus and outcomes, *Journal for healthcare Quality*, 40, 2, 103 – 109	U.S.A.	Retrospective observational study of rapid response events	Descriptive statistics	PEWS	All family activated RRT had appropriate clinical triggers with the most common being uncontrolled pain. More than half of FIRRs had a vital sign change that should have qualified Clinician-RRT activation. Seventy-six percent FIRRs needed at least one or more interventions. Twenty-seven percent of family initiated RRTs needed transfer to intensive care unit compared with 60% transfer rate for Clinician RRTs.
Bogert, S., Ferrell, C. and Rutledge, D. N. (2010). Experience with Family Activation Of Rapid Response Teams. MEDSURG Nursing—July/August 2010—Vol. 19/No. 4	U.S.A	Implementation of Condition Help (ConditionH)	Descriptive statistics	Family involvement	Implementation of ConditionH.<br><br>ConditionH being addressed during daily rounds
Bonafide C., Roberts K., Weirich C., Paciotti B., Tibbetts K., Keren R., Barg	U.S.A.	Semi-structured interviews	Grounded theory	PEWS	Disinclination to seek help and concerns about appearing inadequate in front of colleagues

For peer review only - http://bmjopen.bmj.com/site/about/guidelines.xhtml

Author	Country	Methodology	Analysis	Search Area	Evidence Contribution
F. and Holmes J. (2013) Beyond Statistical Prediction: Qualitative Evaluation of the Mechanisms by which Pediatric Early Warning Scores Impact Patient safety. *J Hospital Medicine*, 8, 5, 248-253					Informal peer support<br><br>Senior leadership important
Braaten, J.S. (2015). Hospital System Barriers to Rapid Response Team Activation: A Cognitive Work Analysis. *American Journal of Nursing*, 115(2), 22-32.	U.S.A.	Document review and interviews using the principles of cognitive work analysis	Inductive and deductive forms of analysis – cognitive work analysis, framework and directed content analysis	EWS	Issues around availability of equipment and staffing<br><br>Negative attitude/delays around calling for help with staff needing to justify escalation. Other factors impact upon this including the perception that the situation is under control/perceived business of physicians/not wanting to appear inadequate
Brady P.W., Muething S., Kotagal et al. (2013b). Improving situation awareness to reduce unrecognized clinical deterioration and serious safety events. *Pediatrics,* 131, 1.	U.S.A.		Statistical process control charts	Situational awareness	Concerns about resources reported
Brady, P.W. et al. (2014) Developing and evaluating the success of a family activated medical emergency team: a quality improvement report. *BMJ Qual Saf* 2014; 0: 1–9.	U.S.A.	A retrospective cohort study looking at the association between family and clinician activations and transfer to the intensive care unit following a MET call	Quality improvement methods and statistical process control charts were used to assess the rate of family activation of METs	Family involvement	Direct mechanism for families to activate a MET<br><br>Concerns from clinicians about a family activated MET over-burdening the system are unfounded

For peer review only - http://bmjopen.bmj.com/site/about/guidelines.xhtml

Author	Country	Methodology	Analysis	Search Area	Evidence Contribution
Chua, W. L., Mackey, S., Ng, E. K. C., & Liaw, S. Y. (2013). Front line nurses' experiences with deteriorating ward patients: a qualitative study. *International Nursing Review*, 60, 4., 501-509.	Singapore	A qualitative survey using critical incident technique	Inductive content analysis	EWS	Staff felt that they had not been educated to an adequate level – training lacking<br><br>Negative attitude towards calling for help – fears of appearing inadequate
Cioffi, J. (2000). Nurses' experiences of making decisions to call emergency assistance to their patients. Journal of Advanced Nursing, 32(1), 108-114.	Australia	Unstructured interviews with nurses who had activated the medical emergency team (MET)	Simple code and retrieve	EWS	Reluctance to activate – doubting ability; fears of appearing inadequate; decisions made based on the perceived availability of resources/business of medical staff/time of day all had an impact on decisions to activate the MET<br><br>Importance of having staff concern in the MET criterion
Cioffi, J. (2000). Recognition of patients who require emergency assistance: a descriptive study. Heart & Lung, 29(4), 262-268.	Australia	Unstructured interviews	Simple code and retrieve	EWS	Importance of having staff concern in a calling criteria<br><br>Reluctance to activate – business of ward a factor
Cioffi, J., Salter, C., Wilkes, L., Vonu-Boriceanu, O., & Scott, J. (2006). Clinicians' responses to abnormal vital signs in an emergency department. Australian Critical Care, 19(2), 66-72.	Australia	Focus groups with clinicians and nurses exploring their responses to abnormal vital signs	Constant comparison	EWS	Availability of equipment an issue/staffing pressures; staff unable to carry out routine monitoring that would enable the detection of abnormal vital signs (AVS)/escalation hampered because of difficulty finding the appropriate senior person<br><br>.<br><br>Medical emergency team (MET) criteria used to confirm or identify deterioration depending on experience<br><br>Negative attitude towards asking for help - lack of confidence questioning peers/fear of being reprimanded /feeling the situation was under control
de Groot, J F. et al (2018) “Implementing Paediatric	Netherlands	Retrospective patient review &	Descriptive statistics and	PEWS	Easily approachable nurses and physicians, as well as good communication, were considered to be vital for timely intervention in cases of clinical deterioration in

For peer review only - http://bmjopen.bmj.com/site/about/guidelines.xhtml

Author	Country	Methodology	Analysis	Search Area	Evidence Contribution
Early Warning Scores Systems in the Netherlands : Future Implications.. *BMC Pediatrics*, 18: 128		semi structured interviews with professionals	grounded theory		paediatric patients.<br><br>Facilitators for the implementation of registration of PEWS included the integration of PEWS scores into the electronic patient records
Dean, BS, Decker MJ, Hupp D, Urbach AH, Lewis E, Benes-Stickle J (2008) Condition HELP: a pediatric rapid response team triggered by patients and parents. *J Health Qual*, 30, 3, 28-31	U.S.A.	Two-year reflection following implementation of Condition Help (ConditionH)	Descriptive statistics	Family involvement	ConditionH criteria for activation<br><br>Concern that family activated RRS could divert attention away from resources<br><br>Clinician involvement important<br><br>Daily 'patient rounds' involving patients and families is useful<br><br>Patients and families have access to relevant information and understand the medical information and care plans.
Demmel KM, Williams L, Flesch L. (2010) Implementation of the Pediatric Early Warning Scoring System on a Pediatric Hematology/Oncology Unit. *Journal of Pediatric Oncology Nursing*, 27(4):229-240	U.S.A.	Discussion of the set-up and implementation of a paediatric early warning scoring tool and an associated algorithm	Rapid Plan-Do-Study-Act (PDSA) cycles were implemented using small tests of change.	PEWS	Education package developed around the history and development of PEWS along with the rationale for and the goals of the initiative. The scoring process was explained and how it would be integrated into routine nursing assessments; normal vital sign parameters were reviewed.<br><br>Importance of common information spaces & display of activation criteria throughout the hospital<br><br>Senior lead commitment & importance of champions integral for implementation
Donohue, L. and Endacott, R. (2010) Track, trigger and teamwork: Communication of deterioration in acute medical and surgical wards. *Intensive and Critical Care Nursing*, 26, 10-17	U.K.	Qualitative design with critical incident technique. Semi-structured interviews with nurses and the outreach team	Thematic analysis	EWS	Some resistance to escalation – clinicians preferring to deal with patient problems within their own team<br><br>Inexperienced staff teaming up with more experienced staff once patient deterioration was recognised
Downey, C.L., Tahir, W., Randell, R., Brown, J.M.	U.K.	Narrative review	"Patterns were identified and	PEWS	Impact on communication – packaging information. Facilitates communication across hierarchies.

For peer review only - http://bmjopen.bmj.com/site/about/guidelines.xhtml

Author	Country	Methodology	Analysis	Search Area	Evidence Contribution
and Jayne, D.G. (2017) Strengths and limitations of early warning scores: A systematic review and narrative synthesis. *International Journal of Nursing Studies*, 76, 106-119			translated to themes, which were further refined using an iterative process".		
Endacott, R., & Westley, M. (2006). Managing patients at risk of deterioration in rural hospitals: a qualitative study. Australian Journal of Rural Health, 14(6), 275-279	Australia	Questionnaire, in-depth interviews and observations	Content analysis and constant comparison	EWS	Art of referral important – using the right language and suggesting actions that would be acceptable to the doctor<br><br>Availability of equipment a factor<br><br>Negative attitude towards calling for help; Escalation dependent upon perceived capability of medical staff
Ennis, L (2014) Paediatric early warning scores on a children's ward: a quality improvement initiative. *Nursing children and young people*, 26, 7, 25-31	Ireland	Description of implementation of paediatric early warning score (PEWS) and subsequent audit (prospective cohort observational study)	Simple descriptive statistics of numbers children triggering PEWS and compliance with escalation protocol	PEWS	Structured education and training programme on the use of ISBAR (Identify, Situation, Background,<br><br>Assessment and Recommendation) and PEWS was provided and nurse manager/staff nurse in charge should review any educational requirements in completing PEWS, particularly for relief staff<br><br>Common information spaces important & display of activation criteria throughout the hospital<br><br>Usefulness of ISBAR as a communication tool<br><br>Senior lead commitment – PEWS management policy developed/senior staff promote and reinforce use of PEWS
Entwistle, V. (2004). Nursing shortages and patient safety problems in hospital care: is clinical monitoring by families part of the solution? Health	U.S.A.	Editorial	N/a	Family involvement	Little evidence/no evaluations of policies or practices that encourage and support family involvement in clinical monitoring<br><br>Propose the innovative practice of interdisciplinary rounds where families are invited, and communication is directed to the patient and family

For peer review only - http://bmjopen.bmj.com/site/about/guidelines.xhtml

Author	Country	Methodology	Analysis	Search Area	Evidence Contribution
Expectations, 7, pp.1–5 (editorial)					
Gerdick, C., Vallish, R., Miles, K., Godwin, S., Wludyka, P. and Panni, M. (2010) Successful implementation of a family and patient activated rapid response team in an adult level 1 trauma center. *Resuscitation*, 81, 1676-1681	U.S.A.	Routine data collection for number of rapid response team (RRT) calls and the result of these activations and patient/family survey relating to RRT activation	Statistical analysis	Family involvement	Direct mechanism for families to activate the RRT<br><br>Barriers to family activation highlighted, specifically professional resistance<br><br>Physician and leadership support important to overcome barriers
Gill, F.J., Leslie, G.D. and Marshall, A.P. (2016) Family initiated escalation of care for the deteriorating patient in hospital: Family centred care or just "box ticking". *Australian Critical Care*, 29, 195-200	Australia	Commentary drawing together family centred care concepts, the NSQHS Standards and the development of family-initiated care in Australia	N/a	PEWS	Family activated RRTs now increasingly common in Australia. In the first instance, families need to be aware of the policy.<br><br>Stress the importance of understanding the number and nature of the call.<br><br>Reports on health professional's resistance to it.<br><br>Families need vigilance to escalate care. Need resources in order to negotiate hierarchies and boundaries
Greenhouse, P., Kuzminsky, B., Martin, S., and Merryman, T. (2006) Calling a Condition H(elp). *AJN*, 106, 11	U.S.A focus	Discussion about the implementation of Condition Help (ConditionH)	Descriptive statistics	Family involvement	Appropriateness of calls is reported rather that why they are made<br><br>Note some scepticism and wariness among nurses and physicians
Hueckel, R. M., Mericle, J. M., Frush, K., Martin, P. L. and Champagne M. T. (2012). Implementation of Condition Help. Family Teaching and Evaluation of Family Understanding. J Nurs Care Qual Vol. 27, No. 2, pp. 176–181	U.S.A.	Scripted family teaching about RRT activation at the time of patient admission from Condition Help (ConditionH).	Descriptive statistics about delivery of educational programme and rapid response team (RRT) call-out; survey testing family	Family involvement	Description of ConditionHelp<br>Appropriateness of calls is reported rather that why they are made

For peer review only - http://bmjopen.bmj.com/site/about/guidelines.xhtml

Author	Country	Methodology	Analysis	Search Area	Evidence Contribution
			understanding		
James, J., Butler-Williams, C., Hunt, J. and Cox, H. (2010) Vital signs for vital people: an exploratory study into the role of the Healthcare Assistant in recognising, recording and responding to the acutely ill patient in the general ward setting. *Journal of Nursing Management*, 18, 548-555	U.K.	Postal survey with Healthcare Assistants (HCAs) utilising closed and open questions	Descriptive statistics & content analysis of qualitative data	Observations and monitoring	Workload and ward distractions a barrier to activation, such as time spent locating equipment.<br><br>Disinclination to seek help from senior staff/clinicians
Jensen, C. et al. (2018) Pediatric Early Warning Score Systems, Nurses Perspective – A Focus Group Study. *Journal of Pediatric Nursing*, 14, e16–e22	Denmark	Focus group exploring nurse' experiences with PEWS	Qualitative meaning condensation analysis	PEWS	PEWS as a nursing tool and therefore not valued by medic - no universal language because of this; "when you call and saythat they have a PEWS score of 5, then they don't know what 5 means" (FG2 P1).
Kaul, M., Snethen, J., Kelber, S., Zimmanck, K., Maletta, K. and Meyer, M. (2014) Implementation of the bedside paediatric early warning system (BedsidePEWS) for nurse identification of deteriorating patients. *Journal for specialists in pediatirc nursing*, 19, 339-349	U.S.A	Descriptive cross-sectional study; nurse and medical staff survey	Descriptive statistics	PEWS	Noted that the score provides a 'universal language' and interdisciplinary communication
Lobos AT, Fernandes R, Ramsay T, McNally JD. (2014) Patient	Canada	Implementation discussion	Simple descriptive statistics	PEWS	Situation Background Assessment Recommendation (SBAR) helps to establish a common language and guide escalated events

For peer review only - http://bmjopen.bmj.com/site/about/guidelines.xhtml

Author	Country	Methodology	Analysis	Search Area	Evidence Contribution
characteristics and disposition after pediatric medical emergency team (MET) activation: disposition depends on who activates the team. Hospital Pediatrics. 4(2):99-105					Negative attitude towards calling for help – traditional hierarchies a barrier to activation/concerns about communication between primary and responding team<br><br>No false alarms and de-brief useful<br><br>Importance of champions (utilising a social marketing approach) to encourage 'inter-professional collaboration & advisory group to help establish a sense of ownership<br><br>Lack of support from superiors means less likely to escalate
Mackintosh, N., Rainey, H. and Sandall, J. (2012a) Understanding how rapid response systems may improve safety for the acutely ill patient: learning from the frontline. *BMJ Qual Saf*, 21, 135-144	U.K.	Comparative case study a rapid response system (RRS) using ethnographic methods including observations, interviews and documentary review.	Inductive and deductive coding facilitated by NVivo. Also utilised theme building and structuring methods from framework analysis whilst also informed by other theoretical frameworks such as 'technology-in-practice'.	EWS	Availability of equipment an issue where the track and trigger tool (TTT) was electronic<br><br>Gave junior staff license to escalate care.<br>Additionally, 'while standardisation of practice clearly has its benefits, it also comes at a cost that these tools attenuate lower level staff's authority and ability to persuade staff higher up in the organisation of the credibility of their knowledge' p 143.<br><br>Efforts to develop junior staff's communication and clinical understanding need to acknowledge power dynamics at play.<br><br>Usefulness of situation-background-assessment-recommendation' (SBAR) communication tool as part of the escalation policy as reported by staff (not seen in action).<br><br>Negative attitude towards escalation – difficulty in summoning a response<br><br>Senior lead commitment to patient safety was important. Zero tolerance for cardiac arrest was championed by senior staff<br><br>Night-time/out of hours pressures identified
Mackintosh, N., Humphrey, C., & Sandall, J. (2014a). The habitus of 'rescue' and its significance for implementation of rapid response systems in acute	U.K.	Ethnographic perspective; observations, semi-structured interviews	Data were inductively and deductively coded and organised thematically.	EWS	Negative attitude towards seeking help. Escalating care outside the parameters marked by a track and trigger tool proved difficult; Power struggles identified – junior staff have difficulty persuading more senior staff of the credibility of their knowledge<br><br>Difficulties in activation across professional boundaries

For peer review only - http://bmjopen.bmj.com/site/about/guidelines.xhtml

Author	Country	Methodology	Analysis	Search Area	Evidence Contribution
health care. Social Science & Medicine, 120, 233-242					
Massey, D., Chaboyer, W., & Aitken, L. (2014). Nurses' perceptions of accessing a Medical Emergency Team: a qualitative study. Australian Critical Care, 27(3), 133-138	Australia	In depth semi structured interviews	Inductive approach – thematic analysis	EWS	Common information spaces useful. Display of activation criteria throughout hospital<br><br>General negative attitude towards calling for help – appearing inadequate in front of others<br><br>Importance of leadership support<br><br>Peers support – would often consult their colleagues
McCabe, A. and Duncan, H. (2009) “Paediatric early warning systems: where do we go from here?” *Paediatric Nursing*, 21, 1, 14-17	U.K.	Opinion piece about lessons to be learnt from the adult experience of implementing early warning systems	N/a	PEWS	Specific education package needed on how to use an early warning system (EWS) and on basic clinical assessment, guidance and standardisation of observation and monitoring. Advocate situational simulated scenario education and e-learning<br><br>Highlight the usefulness of communication tools such as situation, background, assessment, recommendation (SBAR) for establishing roles and responsibilities, engaging them in making an appropriate management plan that can, if necessary, be escalated<br><br>Senior lead commitment key - reflected in resources and education - to improve the safety and quality of care of hospitalised patients<br><br>Families need to be empowered to request a patient review
McDonnell, A., Tod, A., Bray, K., Bainbridge, D., Adsetts, D., & Walters, S. (2013). A before and after study assessing the impact of a new model for recognizing and responding to early signs of deterioration in an acute hospital. *Journal of Advanced Nursing*, 69(1), 41-52.	U.K.	Single centre, mixed method before and after study including a survey to measure changes in nurses' knowledge after implementation of a track and trigger system (T&Ts). Also, qualitative interviews	Statistical analysis and thematic framework analysis	EWS	Rolling education programme for all nurses on the recognition and response to deteriorating patients & an overview of the T&Ts<br>Workplace pressures; nurses concerned that they couldn't always summon a timely response from doctors /night time pressures also identified<br><br>Need for staff concern in T&Ts

For peer review only - http://bmjopen.bmj.com/site/about/guidelines.xhtml

Author	Country	Methodology	Analysis	Search Area	Evidence Contribution
Monaghan A. (2005) Detecting and managing deterioration in children. *Paediatric Nursing.* 17(1):32-35	U.K. focus	Commentary on the development of the Brighton paediatric early warning score and setting up a paediatric critical care outreach team.	Simple descriptive statistics of all activations, actions and outcomes during the first three months of implementation	PEWS	Education based model was developed to assist in recognising deterioration<br><br>Temporary staff/workplace pressures impact upon staffs' ability to detect deterioration
Paciotti B, Roberts KE, Tibbets KM, Weirich Paine C, Keren R, Barg FK, Holmes J H and Bonafide CP (2014). Physician attitudes toward family-activated medical emergency teams for hospitalized children. The Joint Commission Journal on Quality and Patient Safety, 40 (4)	U.S.A.	Semi-structured interviews with clinicians to explore physicians viewpoints on families facilitating the identification of children with a deteriorating condition.	Grounded theory and constant comparison	Family involvement	Concerns that resources would be diverted away with an increase in calls – not supported
Pattison, N., & Eastham, E. (2012). Critical care outreach referrals: a mixed-method investigative study of outcomes and experiences. *Nursing in Critical Care,* 17(2), 71-82.	U.K.	Mixed method study looking at the impact of a critical care outreach team (CCOT)	Statistical analysis and grounded theory	EWS	Availability of equipment an issue/workload<br><br>Negative attitude towards calling for help – situation under control/ward business<br><br>Inexperienced staff teaming up/checking with peers before calling the CCOT
Pearson G & Duncan H. (2011) Early warning systems for identifying sick children Paediatrics and	U.K.	Brief review of the evidence base surrounding PEWS together with	N/a	PEWS	Team training & education is important increasing confidence in the use of medical language and empowering bedside carers. 'Although doing observations is fundamental to nursing practice many … have not been taught a structured approach to assessment'. Advocate a simulated environment

For peer review only - http://bmjopen.bmj.com/site/about/guidelines.xhtml

Author	Country	Methodology	Analysis	Search Area	Evidence Contribution
Child Health. 21(5): 232-233		reflections from their own experiences from the Birmingham Children's Hospital			Value of a more structured approach to communication - Advocate the use of a shared communication model such as SBAR (Situation, Background, Assessment and Recommendation) to communicate findings to superiors.<br><br>Need for senior commitment – cultural change may be required to ensure management support (reflected in resources and education) /Importance of champions
Salamonson, Y., van Heere, B., Everett, B., & Davidson, P. (2006). Voices from the floor: Nurses' perceptions of the medical emergency team. *Intensive & Critical Care Nursing*, 22(3), 138-143.	Australia	Survey with closed and open questions to examine perceptions of and satisfaction with the medical emergency team (MET)	Descriptive statistics and content analysis	EWS	Need for more education on deterioration identified<br><br>Negative attitude towards asking for help; attitude of MET team a barrier to activation
Shearer, B., Marshall, S., Buist, M. D., Finnigan, M., Kitto, S., . . . Ramsay, W. (2012). What stops hospital clinical staff from following protocols? An analysis of the incidence and factors behind the failure of bedside clinical staff to activate the rapid response system in a multi-campus Australian metropolitan healthcare service. BMJ Quality & Safety, 21(7), 569-575	Australia	A multi-method study;<br>A point prevalence survey;<br>A prospective audit of all patients experiencing a cardiac arrest, unplanned intensive care (ICU) unit admission or death over an 8-week period.<br>Structured interviews with	Iterative coding	EWS	Adequate staffing and a lack of beds on critical care leads to a failure to activate the RRS<br><br>Score rarely the single determining factor in activation despite the fact that staff recognised patients met activation criteria. Data from the point prevalence study confirm this as only one patient had a serious adverse event<br><br>Negative attitude towards calling for help – situation under control; treating team had expertise to treat (particularly when the physiological instability was in the area of expertise of the treating team)<br><br>Traditional (intra-professional clinical) hierarchies a barrier to activation

For peer review only - http://bmjopen.bmj.com/site/about/guidelines.xhtml

Author	Country	Methodology	Analysis	Search Area	Evidence Contribution
		staff to explore cognitive and sociocultural barriers to activation			
Sonning, K, et al. (2018) "A Survey of Healthcare Professionals' Experiences with the Paediatric Early Warning Score (PEWS)." *Norwegian Journal of Clinical Nursing* 12(64605).	Norway	Questionnaire of a sample of staff who use PEWS	Descriptive statistics	PEWS	Nurses gain self-confidence. More effective communication
Stewart, J., Carman, M., Spegman, A., & Sabol, V. (2014). Evaluation of the effect of the modified early warning system on the nurse-led activation of the rapid response system. *Journal of Nursing Care Quality*, 29(3), 223-229.	Sweden	Mixed method. Retrospective review of records and nurse led focus groups	Statistical analysis and content analysis	EWS	The RRS was valuable for junior staff escalating care across hierarchical and professional boundaries<br><br>Senior lead commitment – culture of support promoted by nursing administrators
Van Voorhis, K.T. and Scahde Willis, T. (2009) Implementing a pediatric rapid response system to improve quality and patient safety. *Pediatr Clin N Am*, 56, 919-933	U.S.A.	Discussion paper highlighting the process of developing a paediatric rapid response system (RRS).<br><br>The system was evaluated by prospectively collected data recorded on RRS activation forms	N/a	PEWS	Display of activation criteria throughout hospital on lanyards and use of white boards useful<br><br>De-briefing following activation and a commitment to no false alarms is encouraged<br><br>Senior lead commitment – administrative arm of the RRS vital<br><br>Utilises ConditionHelp. The appropriateness of calls was facilitated by the 'no false alarms' culture.

For peer review only - http://bmjopen.bmj.com/site/about/guidelines.xhtml

Author	Country	Methodology	Analysis	Search Area	Evidence Contribution
		and existing performance improvement database information.			
Vries, A., Draaisma, J. and Fuijkschot, J. (2017) Clinician Perceptions of an Early Warning System on Patient Safety. *Hospital Pediatrics*, 7, 10, 579-586	Netherlands	Semi-structured interview	Qualitative content analysis	PEWS	PEWS facilitated communication across hierarchies
Watson, A., Skipper, C., Steury, R., Walsh, H. and Levin, A. (2014) Inpatient nursing care and early warning scores: a workflow mismatch. *J Nurs Care Qual*, 29, 3, 215-222	U.S.A.	Mixed method, retrospective medical record observations and observations of nurse interactions in one-minute blocks.	Observation analysis, although this is not described, and statistical analysis	PEWS	Availability of equipment a factor<br><br>Score rarely the determining factor in escalation<br><br>SBAR

FIGURE 1 PRISMA DIAGRAM

For peer review only - http://bmjopen.bmj.com/site/about/guidelines.xhtml

Figure 1: PRISMA Diagram

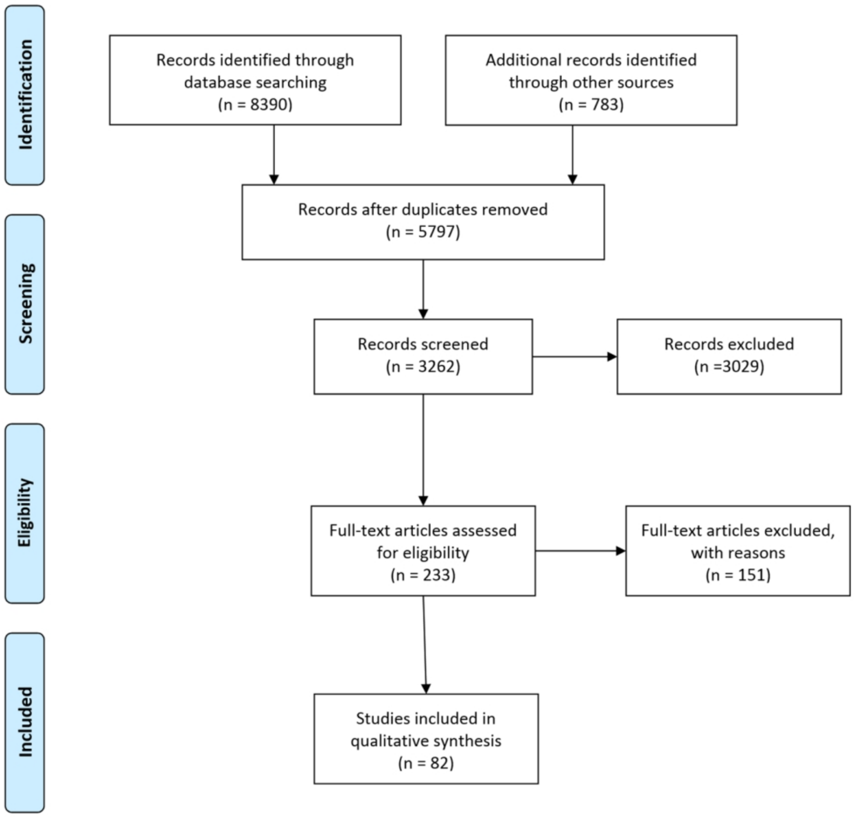

From: Moher D, Liberati A, Tetzlaff J, Altman DG, The PRISMA Group (2009). *P*referred *R*eporting *I*tems for *S*ystematic Reviews and *M*eta-*A*nalyses: The PRISMA Statement. PLoS Med 6(7): e1000097. doi:10.1371/journal.pmed1000097

For more information, visit www.prisma-statement.org.

Figure 1: PRISMA Diagram

Appendix 1: Data Extraction Template 1

PUMA literature review template

Study/Paper	
Authors, title, date, source	
Purpose	
Contribution to review	
Design/Methods	
Participants/Setting	
Data type	
Theoretical framework	
Quality Appraisal Tool	
Quality Assessment	
Intervention type	
Formal study finding	
Recommendations	

For peer review only

Context	
History of intervention	
Country	
Organisation type (i.e. DGH, tertiary hospital, primary care)	
PEWS (efferent) wider activity system elements and inter-relationships with TTT [decision algorithms, CCOT, MET, ICU liaison, PICU, HDU]	
Infra-structural context [other related artefacts]	
Related activity systems [QI, audit, governance, referral processes and inter-relationships with TTT]	
Socio-cultural context [including experience of QI, stability of team and division of labour, teaching-non-teaching etc]	
Wider policy/organisational context	
**Intervention (TIDieR)**	
Name	
Rationale, theory or goal of the elements – active ingredients - identified as essential to the intervention [DA: this could be explicit or implicit in the paper]	
What (materials): Describe any physical or informational	

For peer review only

materials used in the intervention, including those provided to participants or used in intervention delivery or in training of intervention providers.

**TTT Affordances (including but not limited to indicative list)**	
Paper/electronic	
What observations/information are recorded?	
How is data displayed?	
Single parameter/ Aggregated weighting	
Response algorithm(s) (including details)	
Parental/patient involvement	
Manual or automated monitoring	
Proactive intermittent/continuous monitoring	
Linked artefacts (e.g SBAR)	
Audit/performance surveillance	

For peer review only

What (procedures): describe each of the procedures, activities, and/or processes used in the intervention, including any enabling or supportive activities.	
Who provided: For each category of intervention provider (for example, nursing assistant, nurse, junior doctor) describe their expertise, background and any specific training given and also the roles and relationships within the overall division of labour.	
How: Describe the mechanisms (implicit or explicit) about how the intervention, or intervention components have their effects	
How: Describe the modes of TTT delivery (such as face to face or some other mechanism such as phone or internet), or whether the intervention was provided individually or in a group.	
When and how much: Describe the number of times the intervention was delivered and over what period of time,	

For peer review only

including the number of sessions, schedule and intensity of dose (universal or selective application)				
Tailoring: If the intervention was planned to be personalised or adapted, then describe what, why, when and how.				
Modifications: If the intervention was modified during the course of the study, describe the changes (What, why, when, and how)				
Assessment of intervention adherence or fidelity, describe how and by whom, and if any strategies were used to maintain or improve fidelity, describe them				
If intervention adherence or fidelity was assessed, describe the extent to which the intervention was delivered as planned				
**Implementation (NPT) (questions modified from Murray et al. 2010 and kennedy et al 2013)**				
Coherence [i.e. the meaning attributed to PEWS and whether it makes sense for users e.g. cultural and scientific legitimacy, feasibility]<br>• Evidence that the	Policy	Organisational	Professional	Patient/family

For peer review only

intervention was easy to describe<br>• Evidence that it was recognised as different from existing ways of working by stakeholders<br>• Evidence that it has a clear purpose for all relevant actors<br>• Evidence that stakeholders recognise the benefits the intervention will bring and to whom<br>• Evidence that the intervention fitted with the overall goals and activity of the organisation				
Cognitive Participation [i.e. commitment and engagement with the intervention]<br>• Evidence that the target group see the intervention was a good idea<br>• Evidence that they were prepared to invest time and energy and work in it?<br>• Evidence that the stakeholders thought				

For peer review only

their engagement with the intervention was appropriate<br>• Evidence that the stakeholders have taken steps to sustain the use of the intervention				
Collective Action [what are the skills and resources required to enact the intervention?]<br>• How did the intervention affect the user groups?<br>• What work was undertaken to operationalise the intervention?<br>• Did it promote or impede their work?<br>• Did staff require extensive training before they can use it?<br>• How compatible was the intervention with existing work practices?<br>• What impact did it have on the division of labour, resources, power and				

For peer review only

responsibility between different professional groups?<br>• Did it fit with the overall goals and activity of the organisation?				
Reflexive Monitoring [i.e methods for formal and informal evaluation of the effects of the intervention]<br>• Did stakeholders take practical steps to measure the influence of the intervention?<br>• How did users perceive the intervention once it has been in use for a while?<br>• Was the intervention seen as advantageous for patients and staff?<br>• Was it clear what effects the intervention has had (including unintended consequences)?<br>• Did users' contribute feedback on the intervention once it is in use?				

For peer review only

• Was the intervention be adapted/improved on the basis of this experience?				
**Findings (positive, negative and unintended consequences) and useful content**				
**Emergent theories about key ingredients and mechanisms (including summary of supporting evidence)**				
**Emergent theories of the factors necessary for paediatric EWS to be normalised (including summary of supporting evidence)**				
**Additional Insights of note (including references to follow up)**				

For peer review only

Appendix 2: Search Methodology

Search Methods

Database Search

A comprehensive search was conducted across a wide-ranging set *of databases* from 1995 September 2016, which was then extended to May 2018 to identify relevant evidence/studies in English language on

- Paediatric Early Warning Systems (all study types).

A preliminary search strategy was developed using a set of key papers known to the group for Ovid Medline using both text words and Medical subject headings. A further three systematic searches was conducted across a range of databases from 1995 to September 2016 to identify relevant studies in the English language papers reporting on:

- Adult Early Warning Systems (qualitative studies only)
- Interventions to improve situational awareness (all study types)
- Structured communication tools for handover and handoff (all study types)

The Medline search strategy was translated to use across the rest of the databases.

The focus of the search strategy was to achieve high sensitivity and specificity for retrieving studies relevant to the review question. The Medline search strategy was modified according to the indexing systems of the other databases.

Table 1.

Databases
British Nursing Index
CINAHL (Cumulative Index of Nursing and Allied Health Literature)
Cochrane Central Register of Controlled Trials
Database of Abstracts of Reviews of Effectiveness
EMBASE
HMIC (Health Management Information Centre)
Medline
Medline in Process
Scopus
Web of Knowledge (Science Citation Indexes)

Additional searches

In addition to the above databases, we searched both published and unpublished literature. To identify supplementary papers, information on studies in progress, unpublished research or research reported in the grey literature was identified through searching a range of relevant websites and trial registers including Clinical Trials.gov. To identify published resources that had not yet been catalogued in the electronic databases, recent editions of key journals were hand-searched.

Trial Registers
ClinicalTrials.gov<br>https://clinicaltrials.gov/

For peer review only

UK Clinical Trials Gateway<br>http://www.ukctg.nihr.ac.uk/default.aspx
The WHO trial search portal for studies worldwide:<br>http://apps.who.int/trialsearch
**Journal site**
BMJ<br>http://www.bmj.com/theBMJ
BMJ Quality and safety<br>http://qualitysafety.bmj.com/
Websites and organisations
The Health Foundation

Identify relevant studies

The search results would be imported into reference management database *Endnote.* Duplicate references and clearly irrelevant citations will be removed. All remaining studies will then be downloaded into Dropbox for reviewers to screen for relevance.

Medline Search Strategy: Adult Early Warning Systems

1. ("early warning" adj5 scor*).ab,ti.
2. ("early warning" adj5 system* adj5 (deteriorat* or mortality or death or outcome* or harm* or safety)).ab,ti.
3. "acute illness severity".mp.
4. "early medical intervention"/ and ((prevent* or reduc* or improv*) adj5 (deteriorat* or mortality or death or outcome* or harm* or safety)).ab,ti.
5. ("early medical intervention" adj5 (tool* or scor* or index* or indicator* or indice* or assessment* or guide* or instrument* or criteria or parameter* or deteriorat* or mortality or death or monitor* or outcome* or harm* or safety)).ab,ti.
6. exp Health Status Indicators/ and ((tool* or scor* or index* or indicator* or indice* or assessment* or instrument* or criteria or parameter*) adj3 ((prevent* or reduc* or improv*) adj3 (deteriorat* or mortality or death or outcome* or harm* or safety))).ab,ti.
7. "Severity of Illness Index"/ and ((tool* or scor* or index* or indicator* or indice* or assessment* or instrument* or criteria or parameter*) adj5 ((prevent* or reduc* or improv*) adj5 (deteriorat* or mortality or death or outcome* or harm* or safety))).ab,ti.
8. exp Hospitals/ and ((Detecting or managing) adj3 deterioration).tw.
9. ("medical device" adj3 (prevent* or reduc* or improv*)).mp.
10. ("alert criteria" or "activation criteria" or "calling criteria").tw.
11. Hospital Rapid Response Team/
12. Clinical Alarms/
13. (outreach adj3 emergency).tw.
14. VitalPAC Early Warning Score.tw.
15. medical emergency team.tw.
16. Rapid Response Systems.mp.
17. Rapid Response Team.tw.
18. (((Detecting or managing) adj3 deterioration) and warning).tw.

For peer review only

19. track-and-trigger system.tw.
20. (Track adj trigger).tw.
21. (Track and trigger).tw.
22. trigger tools.tw.
23. SBAR technique*.mp.
24. ("rapid response" adj5 (prevent* or reduc* or improv*)).tw.
25. (score adj3 severity of illness).tw.
26. or/1-25
27. (qualitative or ethnograph* or thematic analysis or grounded theory or audio-recorded or transcribed or verbatim or ethnograph* or content analysis technique).ti,ab.
28. (("semi-structured" or semistructured or unstructured or informal or "in-depth" or indepth or "face-to-face" or structured or guide) adj3 (interview* or discussion* or questionnaire*)).ti,ab.
29. ((field or case) adj (stud* or research)).ti,ab.
30. Focus groups/ or Qualitative research/ or Interviews as topic/ or Interview, Psychological/ or ((focus or discussion) adj group*1).ti,ab.
31. (Questionnaires/ or interviews as topic/ or interview, psychological/) and (experience* or predictor* or determinant* or barrier* or facilitator* or enabler* or factor* associat* or perception* or perceive* or attitude* or view*1 or viewpoint* or standpoint* or encounter* or experience* or story or stories or narrative*1 or theme*1 or opinion* or concerns or motivat* or need*1).ti,ab.
32. (cross-sectional studies/ or cross-sectional survey.ti,ab. or correlation study.ti,ab.) and (predictor* or determinant* or barrier* or facilitator* or enabler* or factor* associat* or perception* or perceive* or attitude* or view*1 or viewpoint* or standpoint* or encounter* or experience* or story or stories or narrative*1 or theme*1 or opinion* or concerns or motivat* or need*).ti,ab.
33. process evaluation/ or process evaluation.ti,ab.
34. mixed method*1.ti,ab.
35. ((assoc* factor*1 or predictor* or determinant* or barrier* or facilitator* or enabler*) adj3 (interview* or survey* or questionnaire* or study)).ti,ab.
36. *motivation/
37. ((perception* or perceive* or attitude* or view*1 or viewpoint* or standpoint* or encounter* or experience* or story or stories or narrative*1 or description* or theme* or opinion* or need*1 or concerns or motivat*) adj3 (interview* or survey* or questionnaire* or study or explor* or evaluate or investigate* or analys* or collect*)).ti,ab.
38. (themes adj3 (identif* or analy* or review or explor* or investigat*)).ti,ab.
39. "attitude of health personnel"/ or *attitude to health/
40. exp emotions/
41. consumer satisfaction/
42. personal satisfaction/
43. exp professional-patient relations/
44. exp interprofessional relations/
45. "Health Services Needs and Demand"/
46. or/27-45
47. 26 and 46
48. limit 47 to (english language and humans and yr="1995 -Current")

For peer review only

Medline Search Strategy: Structured communication tools for handover and handoff

1. Situation Background Assessment Recommendation.tw.
2. SBAR.mp.
3. (ABC and "collaborative care").tw.
4. Patient Handoff/
5. Patient Discharge/ and ABC.mp.
6. exp Patient Transfer/ and checklist.mp.
7. (handoff adj3 communication).tw.
8. (handoff adj5 (tool or approach or technique or method)).tw.
9. ("information transfer" and "emergency care").tw.
10. Patient Discharge/ and information transfer.tw.
11. ("information transfer" and "critical care").tw.
12. ("information transfer" and handoff).tw.
13. written checklist.tw.
14. ("Rapid Syndrome Validation Project" or RSVP).tw.
15. "Communication tools".tw.
16. "Escalation of care".tw.
17. or/1-16
18. limit 17 to (english language and humans and yr="1995 -Current")

Medline Search Strategy: Paediatric Early Warning Systems to include observation and training

1. ("early warning" adj5 scor*).ab,ti.
2. ("early warning" adj5 system* adj5 (deteriorat* or mortality or death or outcome* or harm* or safety)).ab,ti.
3. "acute illness severity".mp.
4. "early medical intervention"/ and ((prevent* or reduc* or improv*) adj5 (deteriorat* or mortality or death or outcome* or harm* or safety)).ab,ti.
5. ("early medical intervention" adj5 (tool* or scor* or index* or indicator* or indice* or assessment* or guide* or instrument* or criteria or parameter* or deteriorat* or mortality or death or monitor* or outcome* or harm* or safety)).ab,ti.
6. exp Health Status Indicators/ and ((tool* or scor* or index* or indicator* or indice* or assessment* or instrument* or criteria or parameter*) adj3 ((prevent* or reduc* or improv*) adj3 (deteriorat* or mortality or death or outcome* or harm* or safety))).ab,ti.
7. "Severity of Illness Index"/ and ((tool* or scor* or index* or indicator* or indice* or assessment* or instrument* or criteria or parameter*) adj5 ((prevent* or reduc* or improv*) adj5 (deteriorat* or mortality or death or outcome* or harm* or safety))).ab,ti.
8. exp Hospitals/ and ((Detecting or managing) adj3 deterioration).tw.
9. ("medical device" adj3 (prevent* or reduc* or improv*)).mp.
10. ("alert criteria" or "activation criteria" or "calling criteria").tw.
11. Hospital Rapid Response Team/
12. Clinical Alarms/
13. (outreach adj3 emergency).tw.
14. VitalPAC Early Warning Score.tw.

For peer review only

15. medical emergency team.tw.
16. Rapid Response Systems.mp.
17. Rapid Response Team.tw.
18. (((Detecting or managing) adj3 deterioration) and warning).tw.
19. track-and-trigger system.tw.
20. (Track adj trigger).tw.
21. (Track and trigger).tw.
22. trigger tools.tw.
23. SBAR technique*.mp.
24. ("Situation Background Assessment Recommendation" or SBAR).tw.
25. (patient* adj3 deteriorat*).tw.
26. (deterioration adj3 hospital).tw.
27. (patient deterioration adj4 hospital).tw.
28. (Patients adj4 adverse event).tw.
29. clinical deterioration.tw.
30. ("rapid response" adj5 (prevent* or reduc* or improv*)).tw.
31. (score adj3 severity of illness).tw.
32. Vital signs.tw.
33. or/1-26
34. P?ediatric Early Warning.mp.
35. p?ediatric alert.tw.
36. p?ediatric early warning systems.mp.
37. p?ediatric risk of mortality.tw.
38. P?ediatric Rapid Response Team.tw.
39. Point-of-Care Systems/ and ((paediatric or pediatric) adj3 (improve or identify or detect* or outcome or early or critical or emergency)).tw.
40. P?ediatric Advanced Warning Score.tw.
41. neonatal early warning.tw.
42. infant early warning.tw.
43. p?ediatric rapid response.tw.
44. Bedside paediatric early warning.tw.
45. Bedside PEWS.tw.
46. or/34-45
47. 33 or 46
48. Health Plan Implementation/
49. (implement* or applicat* or execute).tw.
50. (observ* or monitoring or monitor or education).tw.
51. Risk Assessment/
52. Education/
53. Education, Continuing/
54. "Hospitals, Teaching"/
55. Decision Making/
56. Safety Management/
57. Patient Simulation/
58. Awareness/
59. Knowledge/
60. *"Attitude of Health Personnel"/

For peer review only

61. *"Education, Medical, Continuing"/
62. *"Interdisciplinary Communication"/
63. Communication/
64. Monitoring, Physiologic/
65. Decision Making/
66. Judgment/
67. Needs Assessment/
68. Interprofessional Relations/
69. Interdisciplinary Communication/
70. ((organi#ation* adj2 (structur* or form* or function* or determinant* or factors or environme nt* or process* or culture*)) and (outcome? or perform* or satisf* or efficien* or effective* or equ* or growth or develop* or justice or quality or culture* or manage* or leader*)).tw.
71. exp *psychology, industrial/ or *absenteeism/ or *efficiency/ or *job satisfaction/ or *"task performance and analysis"/ or *"time and motion studies"/ or *work simplification/ or *time management/ or *vocational guidance/
72. Decision Support Systems, Clinical/
73. Workflow/
74. or/48-73
75. Nurses/
76. Physicians/
77. (nurse* or physician* or doctor*).tw.
78. Medical Staff/
79. Medical Staff, Hospital/ or Nursing Staff, Hospital/ or Intensive Care Units/
80. or/75-79
81. 47 and 74 and 80
82. limit 81 to (english language and humans and yr="1995 -Current")

Medline Search Strategy: Interventions to improve situational awareness

1 Patient Safety/ (6742)

2 patient safety.tw. (13735)

3 1 or 2 (18307)

4 "Situation* Awareness".tw. (488)

5 3 and 4 (69)

6 limit 5 to (english language and humans and yr="1995 -Current")

For peer review only

Appendix 3: Data extraction template 2

Study/Paper	
Authors, title, date, source	
Purpose	
Design/Methods	
Participants/Setting	
Data type	
Theoretical framework	
Quality Assessment	
Formal study finding	

**Theory Area 1 – Properties of the monitoring system in EWS**
**What are the technical characteristics of the monitoring system?**
**What are the policies/processes associated with the monitoring system?**
**What are roles and responsibilities of the monitoring system?**
**What are the skills and resources needed to enact the monitoring system?**
**How often is the monitoring system applied?**
**How effective is the monitoring system?**
**What is the mechanisms (implicit or explicit) by which the monitoring system has its effects?**
**What actions are necessary to ensure the effectiveness of the monitoring system?**
**What is the relationship of the monitoring system and the context?**
**What is the relationship of the monitoring system to the recording system?**
Is the evidence provided in this theory area good and relevant enough to be included in the synthesis?

**Theory Area 2 – Properties of the recording system in EWS**
**What are the technical characteristics of the recording system?**

For peer review only

**What are the policies/processes associated with the recording system?**
**What are the roles and responsibilities of the recording system?**
**How effective is the recording system?**
**What are the skills and resources needed to enact the recording system?**
**What is the mechanism (implicit or explicit) by which the recording system has its effects?**
**What actions are necessary to ensure the effectiveness of the recording system?**
**What is the relationship between the recording system and the context?**
**What is the relationship between the recording system, the monitoring and detection system?**
Is the evidence provided in this theory area good and relevant enough to be included in the synthesis?

**Theory Area 3 – Properties of the detection/trigger system in EWS**
**What are the technical characteristics of the detection/trigger system?**
**What are the policies/processes associated with the detection/trigger system?**
**What are the roles and responsibilities involved in the detection/trigger system?**
**What are the skills and resources needed to enact the detection system?**
**How effective is the trigger system?**
**What is the mechanism (implicit or explicit) by which the detection/trigger system has its effects?**
**What actions are necessary to ensure the effectiveness of the detection/trigger system?**
**What is the relationship between the trigger system and the context?**
**What is the relationship between the trigger system and the activation system?**
Is the evidence provided in this theory area good and relevant enough to be included in the synthesis?

**Theory Area 4 – Properties of the activation system in EWS**
**What are the technical characteristics of the activation system?**

For peer review only

**What are the policies/processes associated with the activation system?**
**What are the roles and responsibilities involved in the activation system?**
**What are the skills and resources needed to enact the activation system?**
**How effective is the activation system?**
**What is the mechanism (implicit or explicit) by which the activation system has its effects?**
**What actions are necessary to ensure the effectiveness of the activation system?**
**What is the relationship of the activation system and the context?**
**What is the relationship between the activation system and the monitoring, recording and trigger system?**
Is the evidence provided in this theory area good and relevant enough to be included in the synthesis?

**Theory Area 5 – Properties of family involvement in EWS**
**At what stage in the PEWS process are families involved?**
**What are the roles and responsibilities involved in ensuring family involvement?**
**How effective is family involvement in the EWS process?**
**What are the mechanisms by which families are involved in EWS?**
**How effective are mechanisms for involving families in EWS?**
**What are the benefits and dis-benefits of family involvement?**
Is the evidence provided in this theory area good and relevant enough to be included in the synthesis?

**Theory Area 6 – Properties of chart design in EWS**
**What are the optimal properties of chart design to ensure effective monitoring?**
**What are the optimal properties of chart design to ensure effective recording?**
**What are the optimal properties of chart design to ensure effective detection?**

For peer review only

Is the evidence provided in this theory area good and relevant enough to be included in the synthesis?

**Theory Area 7– Properties of implementation and normalisation in EWS**
**What are the barriers to implementation and normalisation of EWS?**
**What actions are necessary to overcome barriers to the implementation and normalisation of EWS?**
**What actions are effective in overcoming barriers to the implementation and normalisation of EWS?**
**What are the mechanisms by which effective actions in overcoming barriers to implementation and normalisation of EWS have their effects?**
**What are the key pillars of an effective reflexive monitoring system?**
Is the evidence provided in this theory area good and relevant enough to be included in the synthesis?

For peer review only

For peer review only

PRISMA 2009 Checklist

Section/topic	#	Checklist item	Reported on page #
**TITLE**			
Title	1	Identify the report as a systematic review, meta-analysis, or both.	1
**ABSTRACT**			
Structured summary	2	Provide a structured summary including, as applicable: background; objectives; data sources; study eligibility criteria, participants, and interventions; study appraisal and synthesis methods; results; limitations; conclusions and implications of key findings; systematic review registration number.	2-3
**INTRODUCTION**			
Rationale	3	Describe the rationale for the review in the context of what is already known.	3-4
Objectives	4	Provide an explicit statement of questions being addressed with reference to participants, interventions, comparisons, outcomes, and study design (PICOS).	n/a
**METHODS**			
Protocol and registration	5	Indicate if a review protocol exists, if and where it can be accessed (e.g., Web address), and, if available, provide registration information including registration number.	2
Eligibility criteria	6	Specify study characteristics (e.g., PICOS, length of follow-up) and report characteristics (e.g., years considered, language, publication status) used as criteria for eligibility, giving rationale.	n/a
Information sources	7	Describe all information sources (e.g., databases with dates of coverage, contact with study authors to identify additional studies) in the search and date last searched.	6
Search	8	Present full electronic search strategy for at least one database, including any limits used, such that it could be repeated.	7, appendix 2
Study selection	9	State the process for selecting studies (i.e., screening, eligibility, included in systematic review, and, if applicable, included in the meta-analysis).	8
Data collection process	10	Describe method of data extraction from reports (e.g., piloted forms, independently, in duplicate) and any processes for obtaining and confirming data from investigators.	8, appendix 1& 3
Data items	11	List and define all variables for which data were sought (e.g., PICOS, funding sources) and any assumptions and simplifications made.	n/a
Risk of bias in individual studies	12	Describe methods used for assessing risk of bias of individual studies (including specification of whether this was done at the study or outcome level), and how this information is to be used in any data synthesis.	n/a
Summary measures	13	State the principal summary measures (e.g., risk ratio, difference in means).	n/a
Synthesis of results	14	Describe the methods of handling data and combining results of studies, if done, including measures of consistency (e.g., I^2) for each meta-analysis.	5-6, 8

For peer review only

For peer review only - http://bmjopen.bmj.com/site/about/guidelines.xhtml

PRISMA 2009 Checklist

Section/topic	#	Checklist item	Reported on page #
Risk of bias across studies	15	Specify any assessment of risk of bias that may affect the cumulative evidence (e.g., publication bias, selective reporting within studies).	n/a
Additional analyses	16	Describe methods of additional analyses (e.g., sensitivity or subgroup analyses, meta-regression), if done, indicating which were pre-specified.	8-9, table 1
**RESULTS**			
Study selection	17	Give numbers of studies screened, assessed for eligibility, and included in the review, with reasons for exclusions at each stage, ideally with a flow diagram.	8, table 2
Study characteristics	18	For each study, present characteristics for which data were extracted (e.g., study size, PICOS, follow-up period) and provide the citations.	Table 3, 4 and 5
Risk of bias within studies	19	Present data on risk of bias of each study and, if available, any outcome level assessment (see item 12).	n/a
Results of individual studies	20	For all outcomes considered (benefits or harms), present, for each study: (a) simple summary data for each intervention group (b) effect estimates and confidence intervals, ideally with a forest plot.	n/a
Synthesis of results	21	Present results of each meta-analysis done, including confidence intervals and measures of consistency.	n/a
Risk of bias across studies	22	Present results of any assessment of risk of bias across studies (see Item 15).	n/a
Additional analysis	23	Give results of additional analyses, if done (e.g., sensitivity or subgroup analyses, meta-regression [see Item 16]).	8-9, box 1
**DISCUSSION**			
Summary of evidence	24	Summarize the main findings including the strength of evidence for each main outcome; consider their relevance to key groups (e.g., healthcare providers, users, and policy makers).	15-16
Limitations	25	Discuss limitations at study and outcome level (e.g., risk of bias), and at review-level (e.g., incomplete retrieval of identified research, reporting bias).	3
Conclusions	26	Provide a general interpretation of the results in the context of other evidence, and implications for future research.	15-16
**FUNDING**			
Funding	27	Describe sources of funding for the systematic review and other support (e.g., supply of data); role of funders for the systematic review.	3

From: Moher D, Liberati A, Tetzlaff J, Altman DG, The PRISMA Group (2009). Preferred Reporting Items for Systematic Reviews and Meta-Analyses: The PRISMA Statement. PLoS Med 6(7): e1000097. doi:10.1371/journal.pmed1000097

For more information, visit: **www.prisma-statement.org**.

For peer review only - http://bmjopen.bmj.com/site/about/guidelines.xhtml

For peer review only
